# De Novo Design of Specific Heterotrimeric Collagen‐Like Peptides via Genetic Algorithm

**DOI:** 10.1002/advs.202502377

**Published:** 2025-08-18

**Authors:** Thi H. Bui, Oluwakamisi Adetunji, Carson C. Cole, Le Tracy Yu, Caroline M. Peterson, Jeffrey D. Hartgerink

**Affiliations:** ^1^ Departments of Chemistry and Bioengineering Rice University Houston TX 77005 USA

**Keywords:** collagen, de novo design, genetic algorithm, peptides, self‐assembly

## Abstract

Collagen, the most abundant protein by mass in mammals, features a triple‐helical structure composed of three intertwined peptide strands. Nonequivalent strands can assemble, forming heterotrimeric structures, which are more prevalent among natural collagens. However, the design and application of heterotrimeric collagen mimetic peptides (CMPs) are constrained by the potential formation of competing compositions and registers, which impede the formation of target assemblies. Herein, a computational protocol is described, GRACE, utilizing a genetic algorithm to reproducibly generate peptide sequences having a strong tendency to self‐assemble into heterotrimeric triple helices with high specificity. The approach leverages SCEPTTr1.2, a refined scoring function tailored to estimate CMP stability and structure, as the basis for fitness evaluation. Four sets of peptide sequences generated by the algorithm, including two with bio‐relevant integrin‐binding motifs, are experimentally synthesized and characterized. All computationally designed peptides are experimentally shown to self‐assemble into heterotrimeric triple helices with expected registers having a minimum specificity of 13.5 °C. The study represents a robust method to overcome barriers in heterotrimeric CMP design providing a versatile framework for the fundamental study of collagen as well as engineering collagen‐like materials.

## Graphical abstract

GRACE is a computational algorithm producing peptide sequences that will reliably fold into composition and registration‐controlled collagen‐like triple helices with user‐specified thermal stability. Additionally, GRACE allows users to specify the inclusion of biologically relevant (or other) sequences to automatically create tailored triple helices for further study.

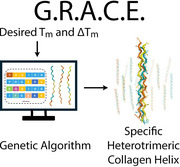

## 1 Introduction

Collagen, known for its distinct polyproline type II (PPII) triple helical structure, is the most abundant protein by mass in mammals.^[^ ^1^ ^]^ Being the major component of the extracellular matrix (ECM), collagens provide structural support and influence cell activity via cell adhesion.^[^ ^2^, ^3^, ^4^ ^]^More than fifty different types of collagen and proteins with collagen‐like domains have been identified in humans, many of which are involved in either fatal diseases such as Ehlers‐Danlos Syndrome^[^ ^5^ ^]^ or critical functionality such as innate immune response.^[^ ^6^, ^7^, ^8^, ^9^, ^10^ ^]^ Due to their biocompatibility and biodegradability, collagen‐based biomaterials have shown extensive applications.^[^ ^11^ ^]^ Therefore, understanding collagen's structure and stability is essential, both for deciphering natural collagen and for advancing the design and engineering of collagen‐based materials.

**Figure 1:**
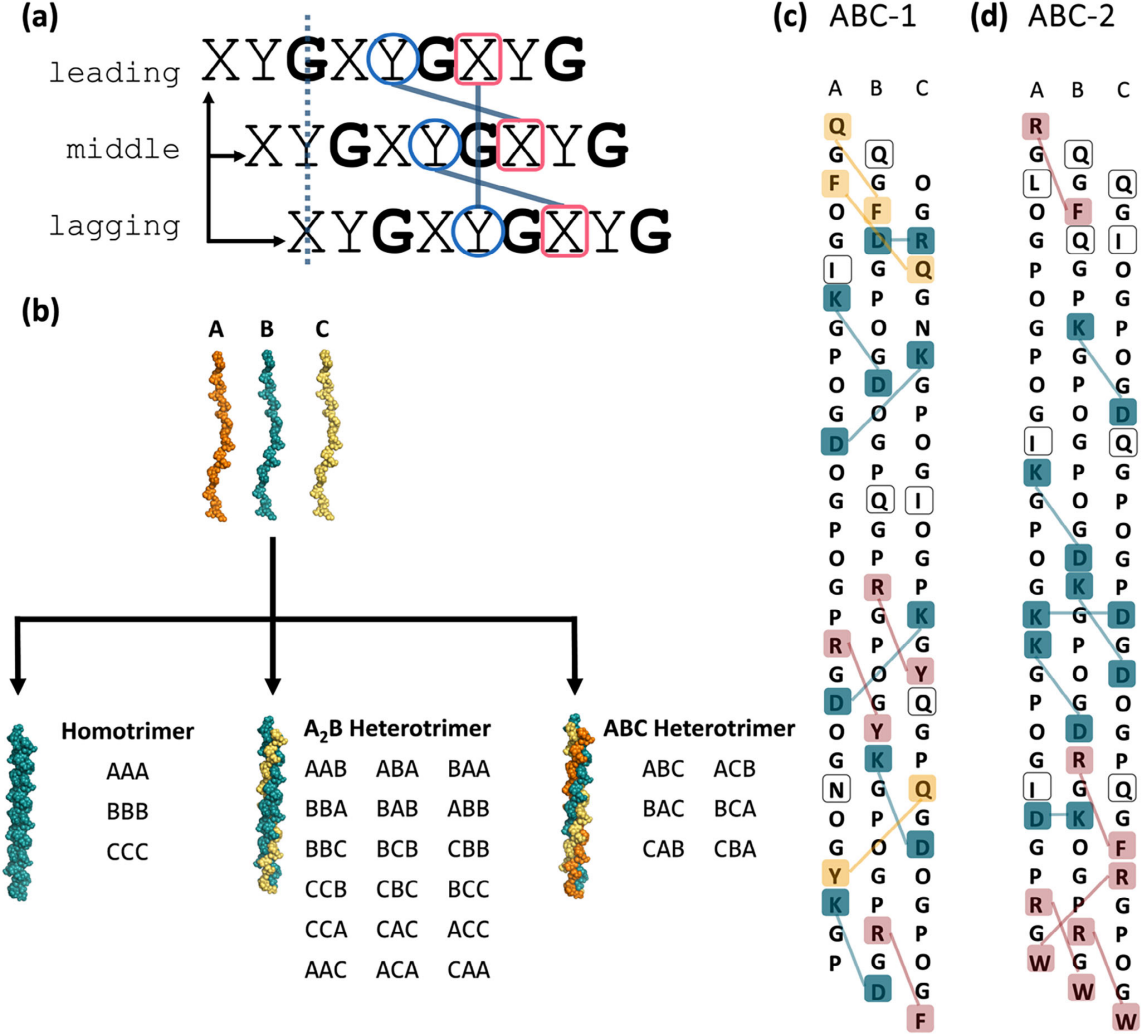
Heterotrimeric assembly directed by pairwise interactions a) Representation of the Xaa‐Yaa‐Gly motif, canonical staggering, and potential axial and lateral pairwise interactions in a triple helix; b) Representation of potential triple helical assemblies in a mixture of peptide A, B, and C; c, d) Sequences of GRACE‐generated peptide ABC‐1 and ABC‐2 highlighting potential axial and lateral pairwise interactions, including charge pairs (teal), cation‐π (pink), amide–π (yellow) interactions, and non‐interacting residues (transparent); peptide strands are presented in the order of predicted registers.

The difficulties in the experimental study of collagen originate from several challenges, including collagen's large size, poor solubility, high cross‐linking, heterogeneous assembly environment, and colocalization of multiple types in the same tissue.^[^ ^3^ ^]^ To overcome these issues, small synthetic collagen mimetic peptides (CMPs) have been utilized to study the structure and self‐assembly mechanism of collagen under controlled conditions.^[^ ^12^ ^]^ CMPs mimic the fundamental structure of collagen with three left‐handed PPII strands coiled together to form a right‐handed superhelix. Each peptide strand features a repeating Xaa‐Yaa‐Gly motif where Xaa is often proline (P) and Yaa is typically (4R)‐4‐hydroxyproline (O) for structural stabilization. A triple helix can be formed by identical (homotrimer) or distinct (heterotrimer) peptide strands. The three peptide strands are offset by a single amino acid residue with respect to one another in the canonical registry, which ensures glycine is present at every helix cross‐section to accommodate a very restrictive steric environment and provide a hydrogen bonding network (**Figure** **1**a).^[^ ^2^, ^3^, ^12^ ^]^

Designing a self‐assembling CMP homotrimer is straightforward. Numerous examples of (POG)_n_ sequences and conservative modifications of this basis sequence have demonstrated their ability to form triple helices.^[^ ^13^ ^]^ Heterotrimer design, which must take peptide composition and register into consideration, has proven to be a more challenging process. In a heterogeneous solution of peptides, different chemical identities of the leading, middle, and lagging strands can lead to the formation of triple helices with various compositions and registers. For example, when mixing two peptides (A and B), eight unique triple helices can be expected to be formed: two homotrimers (A_3_ and B_3_), and two compositions of A_2_B and AB_2_, each of which gives rise to three different heterotrimeric registers. An ABC mixture compounds the possible triple helix formations up to 27 helices (Figure 1b). When designing sequences of peptides to self‐assemble into a heterotrimer, it is important for the final heterotrimer to not only have good thermal stability but also to be the sole existing species at the desired temperature range. Due to the potential for multiple compositions and registers, optimizing sequence design to stabilize the target assembly while destabilizing all the competing species is a non‐trivial task.

It has been shown that self‐assembly of well‐controlled heterotrimeric CMPs can be directed by pairwise interactions between amino acids on adjacent strands.^[^ ^12^ ^]^ These interactions are known to occur in two geometries, axial or lateral, and can either stabilize or destabilize the helical structure depending on the amino acids involved. Axial pairwise interaction occurs approximately parallel to the long axis of the helix, between the Yaa of the n^th^ triplet on one strand and the Xaa of the (n+1)^th^ triplet on the adjacent strand (Figure 1). In the lateral geometry, the interaction between Yaa on the n^th^ triplet and Xaa on the n^th^ triplet of the neighboring strand occurs approximately perpendicular to the helix (Figure 1). As imino acids pre‐organize individual strands into the PPII conformation, all substitutions by natural amino acids result in lower melting temperatures compared to the canonical (POG)_n_.^[^ ^13^, ^14^ ^]^ However, the net stability of a triple helix can still be maintained by simultaneously modifying both Xaa and Yaa positions to amino acids that can form stabilizing pairwise interactions.^[^ ^15^, ^16^, ^17^, ^18^, ^19^, ^20^ ^]^

Considering the distinctive characteristics and structural complications of the triple helix, computational tools tailored specifically to collagen can be an important aid in the design of CMPs. Thanks to the availability of hundreds of published CMPs, several models have been developed to enhance the efficiency in the design process. The Buehler group utilized deep learning (ColGen) and natural language processing (CollagenTransformer) approaches to develop models for predicting the thermal stability of collagen triple helices.^[^ ^21^, ^22^, ^23^ ^]^ They were able to demonstrate that machine learning can be applied to predict triple‐helical stability exclusively through sequence information, without considering other physical and chemical properties. However, their models only evaluate homotrimers. Efforts have also been made to develop several physics‐based models that achieve a great degree of predictive power in a broader scope. The first algorithm to predict the stability of CMPs, Collagen Stability Calculator (CSC), was published by Brodsky and co‐workers.^[^ ^14^ ^]^ This algorithm considers the propensity of all canonical amino acids in Xaa and Yaa positions and pairwise interactions to evaluate the stability of homotrimers. Although CSC established a standard for predicting collagen stability, the model is also limited to homotrimer evaluation and results in predictions with low precision. Subsequently, many computational models optimized for the pairwise interactions between charged amino acid residues have been published.^[^ ^24^, ^25^, ^26^, ^27^, ^28^, ^29^ ^]^ These models achieved success in predicting both homotrimers and heterotrimers with enhanced accuracy. However, their successes were only applicable to a subset of interactions and amino acid substitutions. In 2021, our group introduced the first version of the “Scoring function for Collagen Emulating‐Peptides’ Temperature of Transition” (SCEPTTr 1.0).^[^ ^30^ ^]^ This physics‐based scoring function considers the effect of amino substitution, pairwise interactions, and other features to predict the melting temperature of triple helices. It can analyze both homotrimers and heterotrimers with both canonical and non‐canonical registration to estimate the specificity of the most stable assembly. SCEPTTr 1.0 was tested against the most comprehensive library to date (431 CMPs), resulting in the highest precision and accuracy compared to previously published models. Subsequently, experimental data from twelve peptides having amide‐π interactions were added to the library, and the scoring parameters of SCEPTTr 1.0 were re‐tuned to produce SCEPTTr 1.1.^[^ ^19^ ^]^ The updated scoring function achieved significantly improved predictions for Gln–Phe and Asn–Phe interactions.

Despite the advantages of SCEPTTr in accelerating the design process, the preliminary analysis to achieve the initial sequence input (including evaluating competing species, amino acid propensity at different positions, pairwise interactions, along with other factors related to CMP folding) remains a complex and time‐consuming task. This task is made considerably more difficult if sequences are further constrained by requirements for biologically relevant motifs such as the incorporation of a protein binding site. A robust method for generating sequences that can self‐assemble into highly stable and specific triple helices, similar to those developed for α‐helical coiled coils,^[^ ^31^, ^32^ ^]^ would serve as a critical tool to help overcome these challenges. The Nanda group leveraged Monte Carlo Simulated Annealing (MCSA) to design sequences of ABC‐type heterotrimers.^[^ ^24^, ^25^ ^]^ However, their approaches suffered in terms of optimization scope, which considered only charged residues. Previously, our lab used a genetic algorithm, an exploration technique that is inspired by the theory of natural evolution, to generate CMP heterotrimers.^[^ ^26^ ^]^ A fitness function utilizing electrostatic lysine‐aspartic acid charge pairs resulted in the generation of A_2_B and ABC‐type heterotrimers. However, that algorithm was limited to only axial pairwise interaction and cannot estimate a value for the melting temperature. Khare and co‐workers also implemented a deep learning model to evaluate fitness scores for their genetic algorithm, published as ColGen‐GA, but their algorithm's capability is limited to homotrimer generation.^[^ ^23^ ^]^ An in‐depth discussion of algorithms preceding this study is reported in the Supporting Information (see Section S2 and Table S4, Supporting Information).

Herein, we describe GRACE (Genetically Refined Algorithm for Collagen Engineering), a new generation of genetic algorithm designed to discover chemically diverse heterotrimeric triple helices with high specificity. Additionally, GRACE can incorporate user inputs of biologically relevant sequences, such as binding motifs, and preserve these inputs throughout the optimization process. To validate the algorithm's efficacy, four GRACE‐generated triple helices, comprising both A_2_B and ABC compositions, as well as two that feature collagen‐specific α2β1 integrin‐binding motifs, were experimentally characterized. Circular dichroism (CD) and nuclear magnetic resonance (NMR) data confirmed that all peptides indeed self‐assembled into the predicted heterotrimeric helices with high specificity of composition and register.

## 2 Results and Discussion

### 2.1 Construction of GRACE

**Figure 2:**
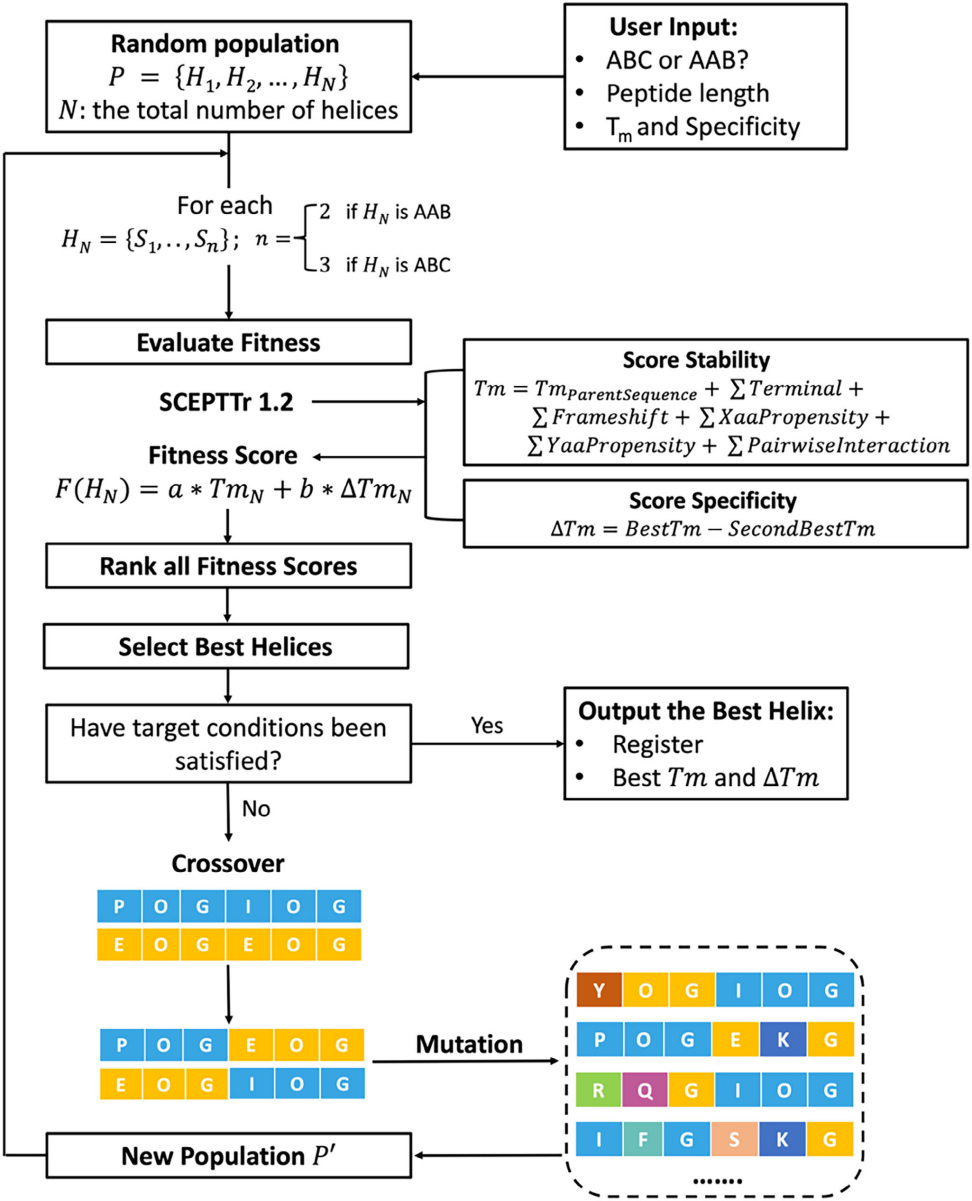
Flowchart describing the logical flow of GRACE to generate sequences of heterotrimeric triple helices. GRACE begins by accepting user inputs as the target conditions for termination. Then, a random population P of collagen‐like peptide sequences is generated. The stability and specificity of each triple helix *H_N,_* the initial population, is estimated by SCEPTTr 1.2. These values are then input into the fitness function *F*(*H_N_*) to compute fitness scores. The helices are ranked, and the two with the highest scores are selected as parents for the next generation. If the best helix meets the target conditions, GRACE outputs the best triple helix along with estimated values for melting temperature and specificity. If not, the parent helices undergo crossover and further mutation to produce a new generation of population P’. This iterative evolution continues until a helix satisfying all user‐defined criteria is found.

The genetic algorithm, inspired by natural selection, mimics the process wherein the fittest individuals are selected to reproduce. Offspring that inherit better traits from their parents stand a better chance of survival. This selection process iterates until an individual who can satisfy all given conditions is found. Implementing this concept in the context of collagen triple helix design, we developed GRACE as an algorithm that searches for novel sequences capable of self‐assembling into heterotrimeric triple helices that satisfy specific criteria defined by users. These criteria include types of compositions (A_2_B or ABC), length of the peptide strands, target melting temperature, and specificity. The general workflow of GRACE is demonstrated in **Figure** **2**.

Built on the success of the two previous versions of SCEPTTr,^[^ ^19^, ^30^ ^]^ we further refined the scoring function to serve as the basis for fitness evaluation in our genetic algorithm. The most up‐to‐date version, SCEPTTr 1.2 described here, considers additional effects of frameshift and terminal amino acids on the stability of the triple helix. 113 new peptides from our own work and the literature were curated and added to the training library (Table S1, Supporting Information) to improve the scope of our scoring function. Experimental characterizations of previously unpublished peptides are reported in Section S1.2 (Supporting Information) “CD melting curve, derivatives, mass spectra, and UPLC of new peptides in the library”, Table S2, and Figures S2–S22 (Supporting Information). Improvements were observed for the predictions of peptides with prefixes and suffixes, peptides with various frameshifts and capping conditions, and peptides with cation‐π interactions (Table S1 and Figure S23, Supporting Information). An extensive description of the scoring function refinements and library updates can be found in the Supporting Information (see Section S1 “Refinement of SCEPTTr”).

The genetic algorithm begins with user‐defined inputs: desired composition, peptide length, minimum melting temperature, and specificity. A random initial population size of 500 triple helix sets was chosen because it allows enough diversity while not compromising computational efficiency (Tables S17–S21, Supporting Information). Each triple helix set consists of two or three collagen‐like sequences, as defined by the user. Sequences are encoded as character vectors using the 20 canonical amino acid abbreviations and “O” for 4‐hydroxyproline. To ensure the initial population has the representation of all possible frames (Gly‐Xaa‐Yaa, Yaa‐Gly‐Xaa, or Xaa‐Yaa‐Gly), the position of glycine in the first triplet is randomly chosen. Subsequently, glycine is inserted at every third position after the first one until the end of the sequence, maintaining the collagen‐like motif. All peptides are assumed to be acetylated and amidated to prevent destabilization from uncapped termini. Each triple helix is scored using SCEPTTr 1.2 to estimate melting temperature (Tm) and specificity (∆Tm). The fitness score is calculated as F(H_n_) = 0.5 Tm_n_ + 0.5 ∆Tm_n_. The two highest‐scoring helices are selected as parents for the next generation.

New populations are generated through operating crossover and mutation on the chosen parent helices. Crossover is the process by which segments of two parent triple helices are combined to produce a new offspring triple helix. The crossover point is randomly chosen at any Gly position along the length of the helix. The mutation process introduces random changes at Xaa and Yaa positions. To maintain a high‐quality pool, parent helices are carried over to the next generation. Crossover and mutation rates are set at 0.6 and 0.2, respectively. Based on our observations after testing a variety of different values, the chosen rates converged on solutions the most effectively (Tables S7–S21, Supporting Information).

The convergence time of our genetic algorithm varies depending on the given targets. For realistic targets, such as a stability range of 30–40 °C and specificity of 5–10 °C, the algorithm typically produces results within minutes using a standard laptop computer. However, for more challenging or unusual targets, very high melting temperature, and/or specificity, GRACE may take hours to converge. The long run time can be explained by the stochastic nature of the genetic algorithm and the large exploratory space involving 20 possibilities of amino acids for each Xaa and Yaa position, as well as multiple pairwise interactions. To mitigate these challenges, we allow user customization of amino acids in the Xaa and Yaa positions. When users choose to exclude specific amino acids at Xaa and/or Yaa positions, the mutation rate of these amino acids at these positions will be changed to 0. This approach can accelerate the process by narrowing down the search to only include, for example, amino acids with well‐studied pairwise interactions (Figure S25, Supporting Information). With limited amino acid substitutions, the algorithm finds solutions significantly faster given the same target Tm and ∆Tm. The details of runtime comparison are reported in Tables S5 and S6 (Supporting Information). Despite this, the remaining difficulty in achieving exceptionally high melting temperatures or specificity may be due to the limitations in currently known parameters of amino acid propensity and pairwise interactions that facilitate heterotrimeric assemblies. Achieving such targets may require exploring beyond canonical amino acids and supramolecular assemblies.^[^ ^33^, ^34^, ^35^, ^36^ ^]^

We observed instances when the algorithm was trapped in local minima, where offspring generated by crossover or mutation show little improvement over their parents. In such cases, a simple approach of re‐initializing the population by starting the algorithm over can help escape the trap by setting the search at a different location. In addition, it is worth noting that the searching process was terminated upon a set of peptide sequences satisfying all the predefined criteria being found. This does not necessarily imply that this output is the most optimal solution that can be achieved, as the search is not exhaustive. Further judgment by users with physics‐based intuition may produce better heterotrimers.

During the development and refinement of GRACE, numerous chemically diverse triple helices were generated. Two of these, generated without any restrictions in amino acid substitution, were analyzed and experimentally characterized here as proof of principle. The two generated sets of sequences were chosen because they exhibit a variety of pairwise interactions stabilizing a targeted ABC register without any manual refinement or editing. Each heterotrimer has multiple potential electrostatic interactions in both axial and lateral geometries, while cation‐ π and amide‐ π interactions are suggested in axial geometries (Figure 1c,d). Non‐interacting residues are also found in GRACE‐generated sequences, presumably as a negative design to destabilize competing species. The melting temperature and specificity of heterotrimer ABC‐1 are predicted to be 39.7 and 16.0 °C, respectively, while those of heterotrimer ABC‐2 are 63.9 and 26.3 °C, respectively (**Table** **1**).

**Table 1:** Predicted melting temperatures, specificities, and registers of GRACE‐generated heterotrimers. Comprehensive Tm predictions of all possible registers are reported in Table S23 (Supporting Information).

Heterotrimer	Melting Temperature [°C]							Specificity [°C]
	A	B	C	AB	AC	BC	ABC	
ABC‐1	< 10.0	<10.0	<10.0	23.6 {ABB}	14.0 {AAC}	23.7 (BCC}	39.7 {ABC}	16.0
ABC‐2	14.3	15.1	17.7	33.7 {AAB}	28.3 {ACC}	37.6 {CBC}	63.9 {ABC}	26.3
AAB‐FOGER	28.4	<10.0	N/A	45.8 {AAB}	N/A	N/A	N/A	17.4
ABC‐FOGER	<10.0	<10.0	14.0	22.8 {ABB}	22.9 {ACC}	24.9 {CBC}	47.5 {ABC}	22.6

Letters inside the curly brackets indicate the predicted registers; Specificity = Tm_Most stable assembly_ – Tm_Second most stable assembly_

### 2.2 Experimental Characterization Validates Heterotrimeric Triple Helical Formations

**Figure 3:**
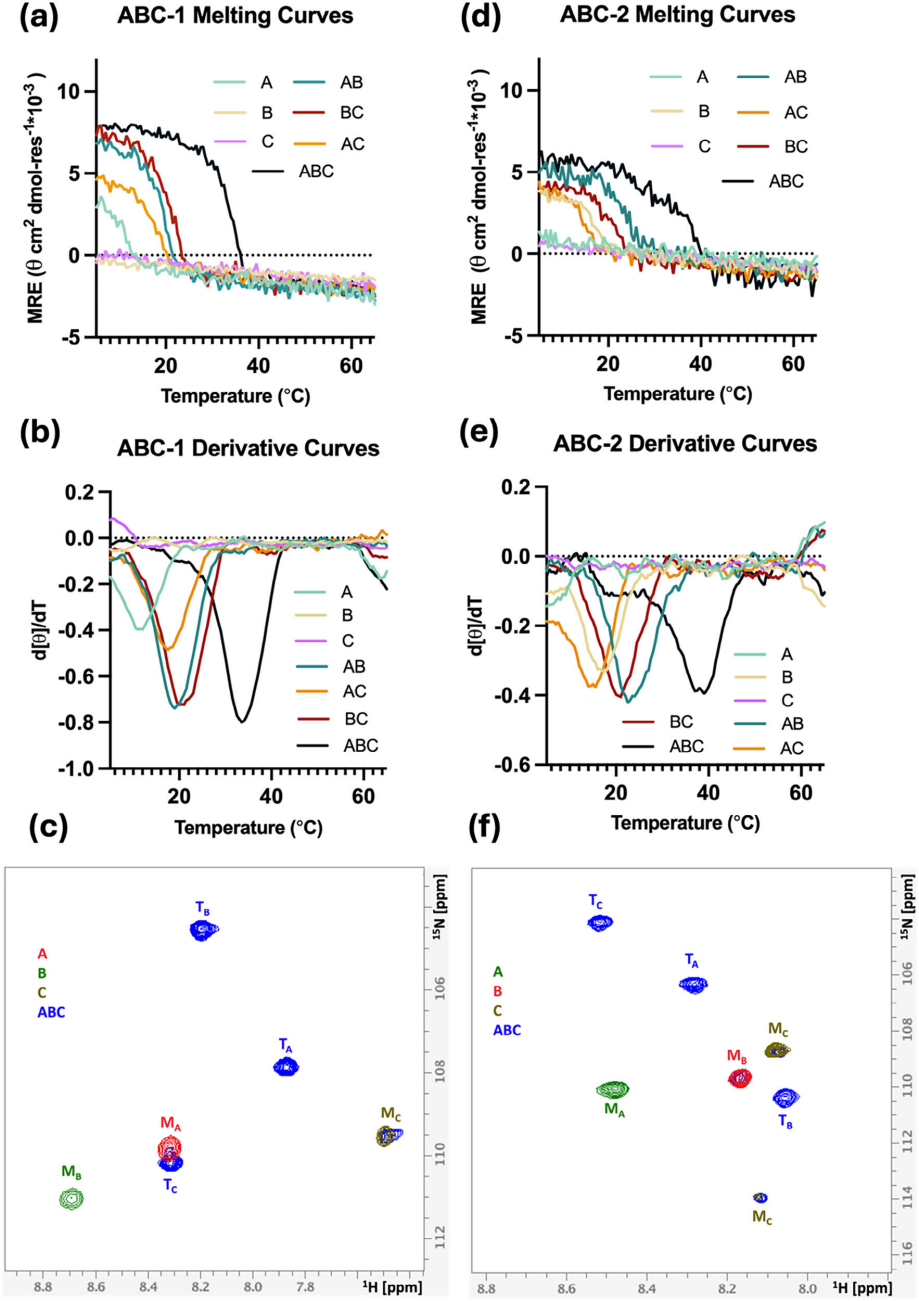
CD and NMR of ABC‐1 and ABC‐2 heterotrimers. a) Melting curves of heterotrimer ABC‐1 determined by CD. b) First‐order derivative of ABC‐1 melting curves. d [θ] is the change in mean residue ellipticity (MRE), and dT is the change in the temperature. The minimum of each curve indicates the melting temperature (Tm) of the peptide. c) ^1^H‐^15^N HSQC of ABC‐1 unary and ternary peptide solutions at 25 °C. d) CD melting curves of heterotrimer ABC‐2. e) First‐order derivative of ABC‐2 melting curves. f) ^1^H‐^15^N HSQC of ABC‐2 unary and ternary peptide solutions at 25 °C.

To confirm the heterotrimeric assemblies, all peptides were synthesized via solid‐phase synthesis (Table S24, Figure S26‐S36, Supporting Information). To verify the predicted results from GRACE, melting temperature analysis using CD was conducted on seven peptide solutions: three unary solutions of A, B, and C peptides, three binary mixtures at 1:1 ratio of AB, AC, and BC, and one mixture at 1:1:1 ratio of ABC. Spectra measurements were performed to confirm the secondary structures in each sample solution (Figure S37a,b, Supporting Information). The experimental melting temperatures of different peptide combinations of each heterotrimer are presented in **Table** **2** and **Figure** **3**. The specificity is determined as the difference between the melting temperature of the most stable and the second most stable assemblies. In both ABC‐1 and ABC‐2, the highest melting temperatures were observed in the ternary mixtures as predicted. The experimental specificity of ABC‐1 and ABC‐2 is 13.5 and 16.0 °C, respectively. These observations indicate a preference for ABC assemblies in both systems.

**Table 2:** Experimental melting temperatures and specificities of different peptide combinations of GRACE‐generated heterotrimers.

Heterotrimer	Melting Temperature [°C]							Specificity [°C]
	A	B	C	AB	AC	BC	ABC	
ABC‐1	11.0	<10.0	<10.0	19.5	17.5	20.0	33.5	13.5
ABC‐2	<10.0	17.0	<10.0	22.5	15.5	21.0	38.5	16.0
AAB‐FOGER	18.5	14.5	N/A	34.5	N/A	N/A	N/A	16.0
ABC‐FOGER	<10.0	<10.0	13.0	20.0	26.0	21.5	40.5	14.5

All peptides were characterized at 0.3 mM in 1 mM phosphate buffer; Specificity = Tm_Most stable assembly_ – Tm_Second most stable assembly_.

The CD analysis provides valuable insights into thermal stability; however, it cannot fully resolve the ambiguity in compositions and registers of assemblies in mixed peptide samples due to overlapping melting temperatures. The CD melting curve of ABC‐1 demonstrates a sharp, distinct transition (Figure 3a), whereas the melting curve of ABC‐2 appears broader (Figure 3d). The derivative curve of ABC‐2 shows a local minimum ≈19.0 °C in addition to a global minimum of 38.5 °C, indicating the potential formation of multiple compositions and registers. To gain a more detailed understanding of the assembly of these systems, 2D ^1^H–^15^N heteronuclear single quantum coherence (HSQC) experiments were performed at 25 °C for all peptide mixtures to confirm the compositions. Each peptide strand of a heterotrimer was synthesized with a ^15^N‐labeled glycine. In the 2D ^1^H–^15^N HSQC spectra, each unique conformation of a peptide strand in a unique chemical environment is represented by a single cross‐peak. This strategy allows the simplification of the highly overlapping ^1^H NMR shifts of a peptide. In both ABC‐1 and ABC‐2 systems, the overlaid spectra of unary and ternary solutions show three different peaks corresponding to the heterotrimers, distinctively separated from the monomer peaks (Figure 3c,f). The absence of additional trimer peaks in the spectra suggests that a mixture of A, B, and C peptides forms only a single triple helix at 25 °C. This supports the conclusion that at 25 °C, to the limit of detection by NMR, both the ABC‐1 and ABC‐2 mixtures contain only ABC‐type heterotrimers and monomers with no other triple helical species.

**Figure 4:**
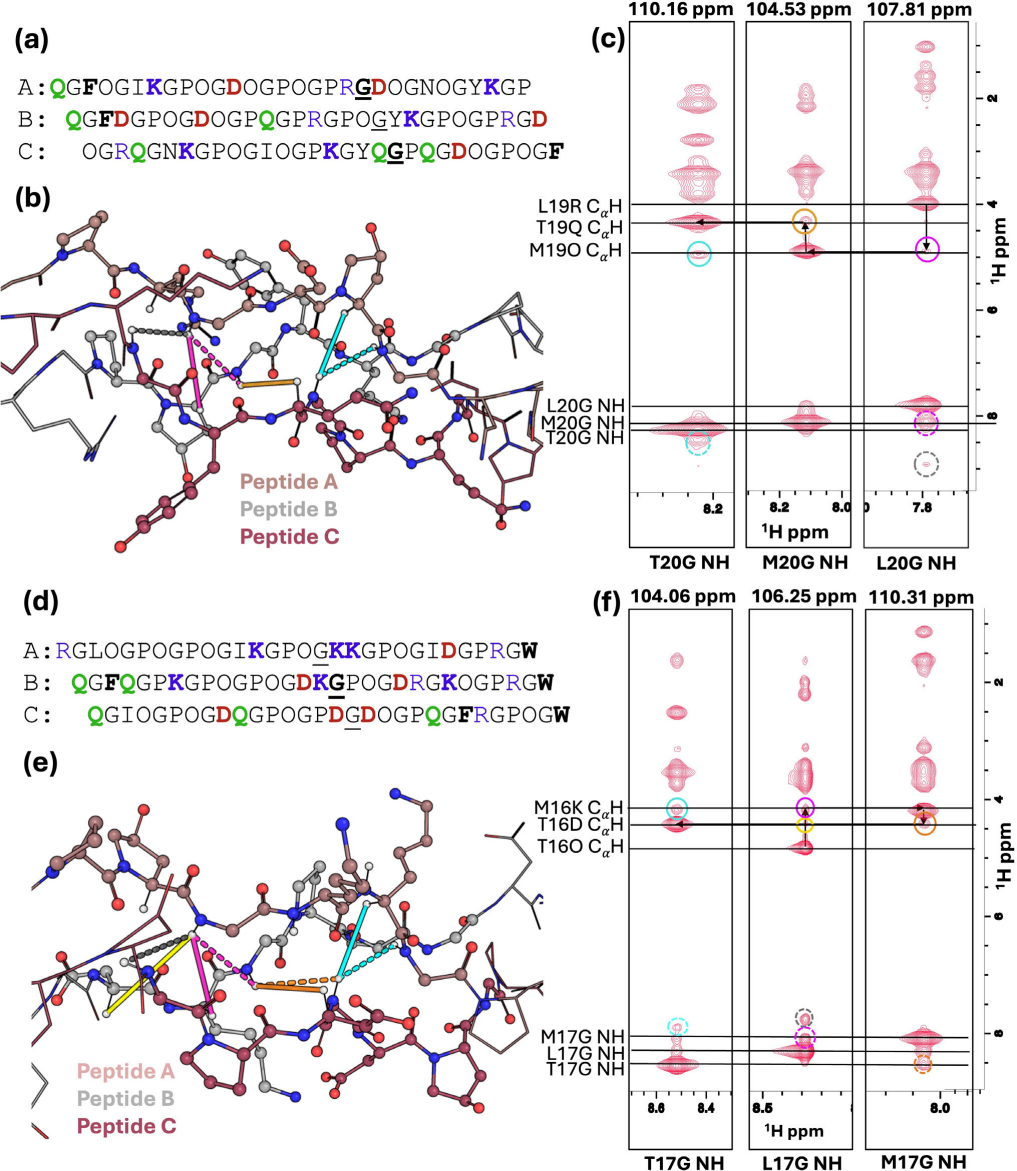
3D ^1^H‐^1^H‐^15^N NOESY HSQC analysis of ABC‐1 and ABC‐2 heterotrimers. a) Sequence and registration of the expected ABC‐1 triple helix with ^15^N‐labeled glycine underlined, and ^15^N‐^13^C‐labeled glycine bolded and underlined. b) Chemical structures of the residues surrounding the isotopically labeled glycine of ABC‐1. Solid lines represent NOEs to Cα‐H; dashed lines represent NOEs to NH; colors of the lines correlate with colors of circles in spectra. c) Three planes of the 3‐D NOESY HSQC taken at ^15^N chemical shifts of 110.16, 104.53, and 107.81 ppm of ABC‐1 at 25 °C. d) Sequence and registration of the expected ABC‐2 triple helix with ^15^N‐labeled glycine underlined, and ^15^N‐^13^C‐labeled glycine bolded and underlined. e) Chemical structures of the residues surrounding the isotopically labeled glycine of ABC‐2. Solid lines represent NOEs to Cα‐H; dashed lines represent NOEs to NH; colors of the lines correlate with colors of circles in spectra. f) Three planes of the 3‐D NOESY HSQC taken at ^15^N chemical shifts of 104.06, 106.25, and 110.31 ppm of ABC‐2 at 25 °C.

Validation of the registration was conducted by 3D ^1^H‐^1^H‐^15^N nuclear overhauser effect spectroscopy (NOESY)‐ HSQC NMR experiments. Each strand of peptide ABC‐1 has one ^15^N‐labeled glycine at position 20 (**Figure** **4**a), and each strand of peptide ABC‐2 has one ^15^N‐labeled glycine at position 17 (Figure 4d). Figure 4c,f each display three ^1^H‐^1^H NOESY planes taken at ^15^N chemical shifts corresponding to the heterotrimer peaks observed in the ^1^H‐^15^N spectra slice. The identification of each plane, as well as helical registration, can be resolved by analyzing the Cα‐H proton region. The cross‐peaks in each plane represent NOEs between the labeled amide proton and nearby protons within ≈5 Å. Each labeled glycine amide proton is expected to show NOEs with its own Cα‐H (≈3.50 ppm) and with two other intra‐ and inter‐strand Cα‐H protons (≈4.00–5.00 ppm). The larger peak in the 4.00–5.00 ppm region corresponds to the Cα‐H of the preceding Yaa residue, while the smaller one represents the Cα‐H of the Yaa residue on the adjacent strand. The triple helical registration, or the correlation from leading to middle to trailing strand, can be determined by sequentially following a path from a large Cα‐H peak vertically to a small Cα‐H peak, then moving horizontally to another large Cα‐H peak, as illustrated in Figure 4c,f.

The arrows in Figure 4c represent the pattern that allows the determination of the triple helical register. This begins from the right plane at ^15^N shift of 107.81 ppm at the large cross peak at 3.99 ppm, which represents the NOEs to the Cα‐H on the preceding Yaa residue on the same strand of the labeled glycine. Moving downward in the same plane to the smaller cross peak at 4.89 ppm, which has its ^1^H chemical shift aligned to the large cross peak on the middle plane. From this large cross peak, progressing upward in the middle plane brings us to the small cross peak at 4.31 ppm, which matches the large cross peak on the left‐hand plane. Considering all these observations, the NOESY plane at ^15^N shift of 107.81 ppm corresponds to the leading strand, the plane at 104.53 ppm to the middle strand, and the plane at 110.16 ppm to the trailing strand. Previous studies on the NOEs in the context of collagen have shown that 4‐hydroxyproline results in a downfield Cα‐H shift.^[^ ^30^, ^37^ ^]^ Therefore, the larger peak at 4.89 ppm in the middle strand plane is assigned to the Cα‐H of Hyp20 on peptide B. In the trailing strand plane, a small peak correlating to the chemical shift of hydroxyproline Cα, marked with cyan lasso in Figure 4c, implies the NOE of the glycine amide proton from the trailing strand to a hydroxyproline Cα‐H. This hydroxyproline can be resolved as Hyp22 on peptide A, which is indicative of the assignment of peptide A to be the leading strand and peptide C to be the trailing strand (Figure 4a,b). From this, the peak at 3.99 ppm in the leading strand plane can be assigned to the Cα‐H on Arg19 of peptide A, and the peak at 4.31 ppm in the trailing strand plane belongs to the Cα‐H of Gln19 in peptide C.

Figure 4f shows three NOESY planes at 104.06, 106.25, and 110.31 ppm, corresponding to the ^15^N shifts of ABC‐2 heterotrimer peaks. Sequential walking reveals that the 106.25 ppm plane corresponds to the leading strand, the 110.31 ppm plane to the middle strand, and the 104.06 ppm plane to the trailing strand. This pattern is illustrated by arrows in Figure 4f. The peak at 4.81 ppm on the leading plane represents a NOE to a hydroxyproline Cα‐H, so this shift is diagnostic of peptide A's labeled glycine. The larger peak at 4.16 ppm on the middle strand plane can be assigned to the labeled glycine's NOE to the Cα‐H of Lys16 on peptide B, while the peak at 4.39 ppm on the trailing strand plane represents the NOE to the Cα‐H of Asp16 on peptide C. The smaller peak at 4.16 ppm in the trailing plane, marked with a cyan circle in Figure 4f, has a chemical shift aligned with the Cα‐H of Lys16 on the peptide B plane. Assigning this peak to the NOE of peptide C's labeled glycine to the Cα‐H of Lys19 on peptide A supports the assignment of peptides B and C as the middle and trailing strands, respectively (Figure 4d,e). Additionally, the small peak at 4.381 ppm, marked with a yellow circle in Figure 4f, corresponds to a minor NOE between the labeled glycine on the leading strand and the more distant Cα‐H of Asp15 on peptide B (middle strand), further confirming our prediction of an ABC register.

The complete peak assignments, including peaks in the upfield and amide regions, for both heterotrimer ABC‐1 and ABC‐2 can be found in the Supporting Information (see section “Structural characterization by NMR”).

### 2.3 GRACE can Accept and Conserve Biologically Relevant Binding Motifs Throughout Optimization

Collagen interacts with other proteins in vivo by presenting bioactive amino acid sequences in a triple helical geometry. For example, integrins interact differently with collagens depending on whether the assembly is heterotrimeric or homotrimeric.^[^ ^38^ ^]^ Previous studies on collagen and collagen‐binding proteins were mostly conducted utilizing homotrimeric CMPs due to the complications of rational heterotrimer design.^[^ ^39^ ^]^ Different amino acids exhibit varying propensities for helical stability; thus, inserting a binding motif in one or more sequences can significantly alter the Tm of the target assembly. Moreover, all changes in competing species need to be considered, as the inserted binding motif might impact the Tm of the target assembly but stabilize competing species, thereby reducing specificity. Control over the register is another critical aspect, as changes in specificity can complicate the prediction of whether the recognition motif will occur on the leading, middle, or trailing strand. The heuristic search by the genetic algorithm can ensure that the generated sequences can experimentally fold into a heterotrimer with desired stability, specificity, and register while keeping the recognition motif intact.

**Figure 5:**
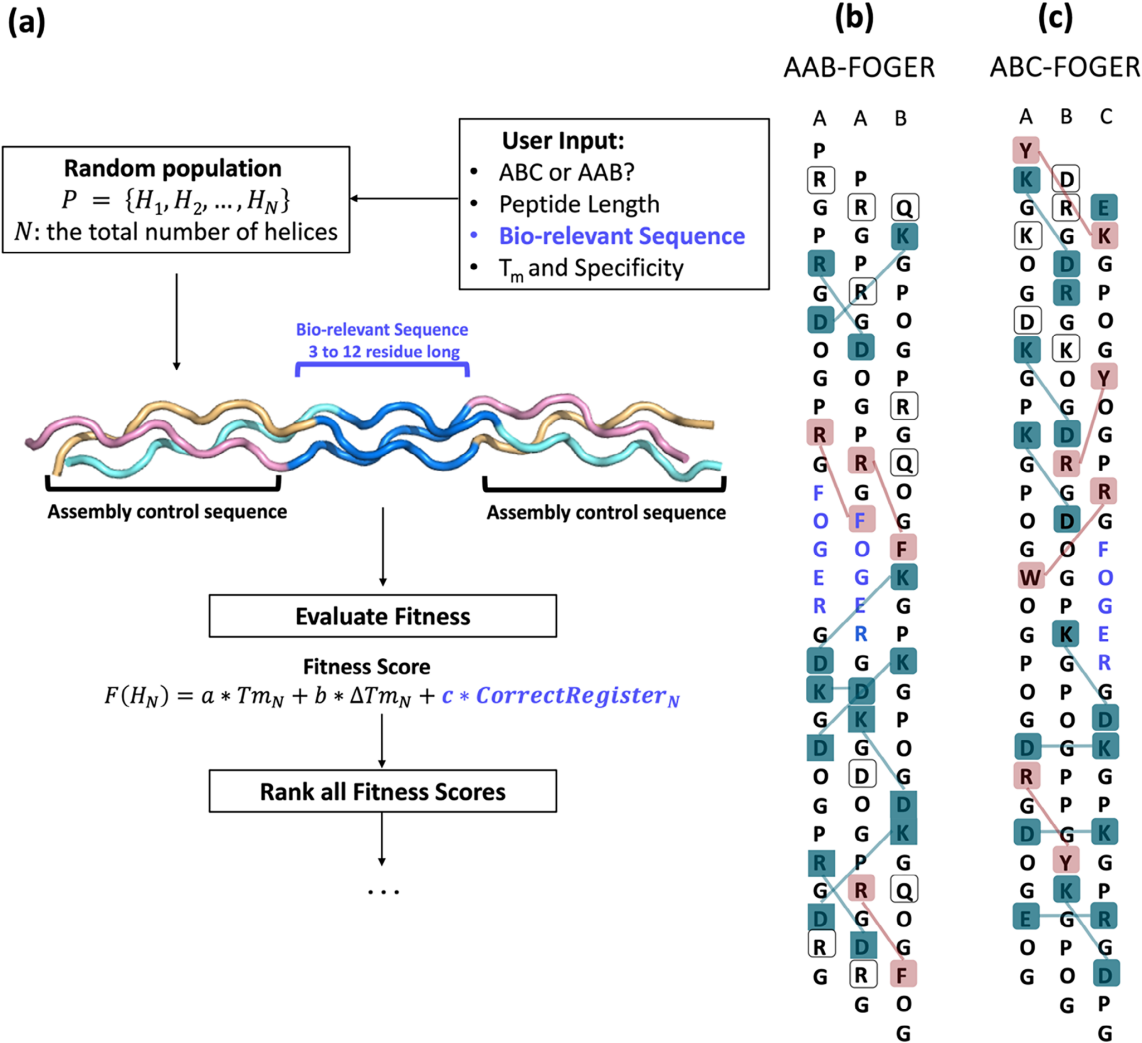
Features of GRACE to design heterotrimers with integrin‐binding motifs. a) A flow chart represents how the algorithm processes user input of a Bio‐relevant Sequence. b, c) Sequences of GRACE‐generated peptides having the GFOGER integrin‐binding motif (colored in blue). Assembly‐control regions show multiple potential axial and lateral pairwise interactions, including charge pairs (teal), cation‐π (pink) interactions, and non‐interacting residues (transparent); peptide strands are presented in the order of predicted registers with one residue staggered.

A feature of GRACE is that the algorithm allows for the incorporation of bio‐relevant motifs by accepting user input of specific sequences. To preserve these motifs throughout the optimization process, the mutation and crossover rates for the inserted sequences are set to zero, ensuring that the bio‐relevant regions remain conserved across generations. To ensure that the conserved sequence adopts the correct helical register as in nature, another parameter*, CorrectRegister*, was incorporated to guide the optimization toward the desired registers. The coefficients a, b, and c are set at 0.4, 0.4, and 0.2, respectively (**Figure** **5**a).

To prove the functionality of this feature, we generated triple‐helical sequences containing the GFOGER motif, an α2β1 integrin‐binding motif found in the α1 strand of collagen I. The algorithm successfully produced an AAB heterotrimer with the GFOGER motif positioned on peptide A, effectively mimicking the structure of natural collagen (Figure 5b). Additionally, the algorithm's capability to control register placement was demonstrated by generating a separate ABC heterotrimer with the binding motif positioned on the trailing strand (Figure 5c). As before, once the desired parameters were set, no further human optimization or editing of the sequences generated was performed. This highlights the versatility of this computational approach in designing bio‐relevant heterotrimers with precise structural properties. Both sets of sequences were generated using limited amino acid substitutions at Xaa and Yaa positions, as reported in Figure S25 (Supporting Information), to improve the search efficiency. The predicted melting temperature and specificity of AAB‐FOGER are 45.8 and 17.4 °C, respectively, while those of ABC‐FOGER are 47.5 and 22.6 °C, respectively (Table 1).

### 2.4 Experimental Validation of Generated Peptides Featuring Integrin Binding Motif

**Figure 6:**
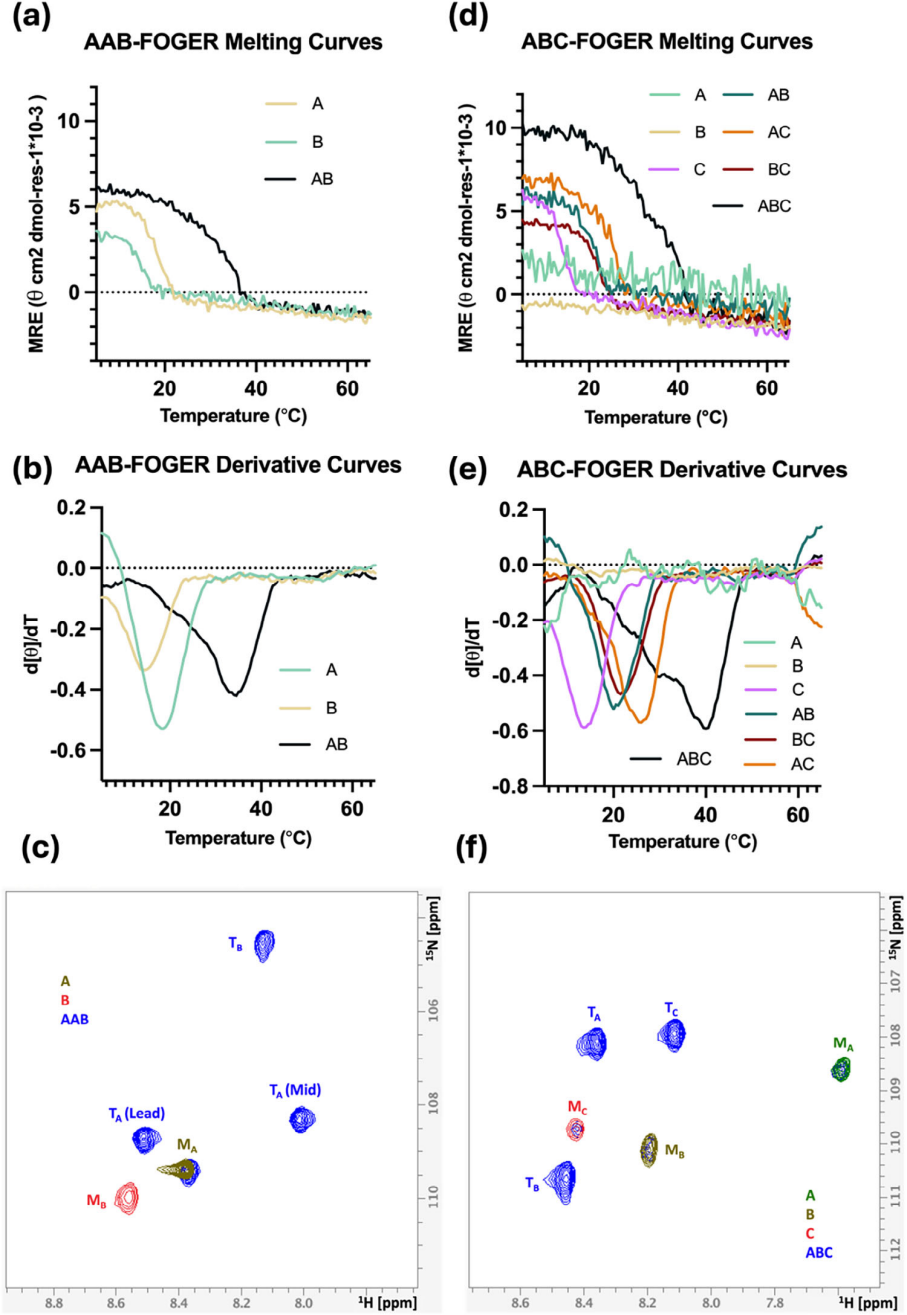
CD and NMR of AAB‐FOGER and ABC‐FOGER heterotrimers. a) Melting curves of heterotrimer AAB‐FOGER determined by CD. b) First‐order derivative of melting curves of AAB‐FOGER. d[θ] is the change in mean residue ellipticity (MRE), and dT is the change in the temperature. The minimum of each curve indicates the melting temperature (Tm) of the peptide. c) ^1^H‐^15^N HSQC of the unary and ternary peptide solutions of AAB‐FOGER at 25 °C. d) CD melting curves of heterotrimer ABC‐FOGER. e) First‐order derivative of the melting curves of ABC‐FOGER. f) ^1^H‐^15^N HSQC of the unary and ternary peptide solutions of ABC‐FOGER at 25 °C.

CD analysis of AAB‐FOGER and ABC‐FOGER was performed on multiple mixtures of peptides in a fashion analogous to ABC‐1 and ABC‐2 as discussed previously. Wavelength scans of the binary mixture of AAB‐FOGER were performed on samples with different mixing ratios, 2:1 A:B and 1:2 A:B. CD spectra of 2:1 A:B sample show a higher MRE value of the maximum at 224 nm (Figure S38, Supporting Information), suggesting this is the preferred ratio for the formation of the triple helix. The melting analysis of AAB‐FOGER and ABC‐FOGER confirms the assembly of heterotrimeric triple helices with specificity of 16.0 and 18.5 °C, respectively (**Figure** **6** and Table 2). ^15^N‐^1^H HSQC NMR of both systems showed only three distinct trimer peaks in addition to the monomer peaks, validating the formation of a single heterotrimeric register in each peptide mixture (Figure 6c,f).

**Figure 7:**
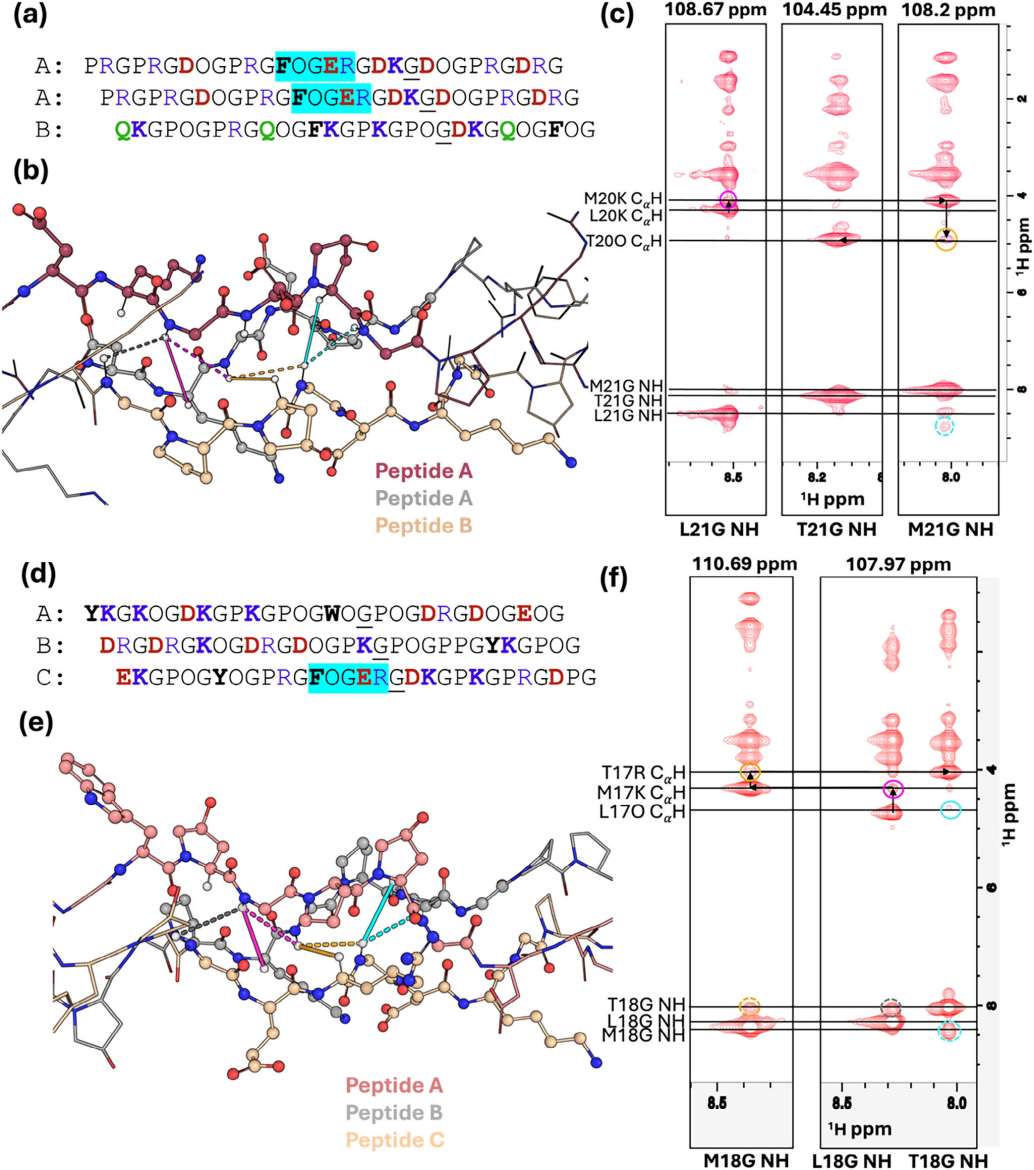
3D ^1^H‐^1^H‐^15^N NOESY HSQC analysis of AAB‐FOGER and ABC‐FOGER heterotrimers. a) Sequence and registration of the expected AAB‐FOGER triple helix with ^15^N‐labeled glycine underlined. b) Chemical structures of the residues surrounding the isotopically labeled glycine of AAB‐FOGER. Solid lines represent NOEs to Cα‐H; dashed lines represent NOEs to NH; colors of the lines correlate with colors of circles in spectra. c) Three planes of the 3‐D NOESY HSQC taken at ^15^N chemical shifts of 108.67, 104.45, and 108.2 ppm of AAB‐FOGER at 25 °C. d) Sequence and registration of the expected ABC‐FOGER triple helix with ^15^N‐labeled glycine underlined. e) Chemical structures of the residues surrounding the isotopically labeled glycine of ABC‐FOGER. Solid lines represent NOEs to Cα‐H; dashed lines represent NOEs to NH; colors of the lines correlate with colors of circles in spectra. f) Two planes of the 3‐D NOESY HSQC taken at ^15^N chemical shifts of 110.69 and 107.69 ppm of ABC‐FOGER at 25 °C.

Data from ^1^H‐^1^H‐^15^N‐NOESY‐HSQC experiments confirmed that the order of peptide strands is consistent with the algorithm predictions. **Figure** **7**c shows NOESY planes of the AAB‐FOGER peptide at ^15^N chemical shifts of 104.45, 108.2, and 108.67 ppm. From the Cα‐H correlation as shown by arrows in Figure 7c, labeled glycine on the leading strand can be assigned to the cross peak at ^15^N chemical shift of 108.67 ppm, on the middle strand to the peak at 108.2 ppm, and on the trailing strand to the peak at 104.45 ppm. In the trailing strand plane, the cross peak with higher ^1^H chemical shift at 4.94 ppm is indicative of the NOE to the hydroxyproline Cα‐H on peptide B. Cross peaks at ≈4.3 and 4.2 ppm in the leading and middle strand planes are NOEs to lysine Cα‐H of peptide A. Figure 7f shows NOESY planes taken at ^15^N chemical shifts corresponding to trimer peaks of ABC‐FOGER heterotrimer. The plane taken at ^15^N chemical shift of 110.69 ppm represents the middle strand. At ^15^N chemical shift of 107.97 ppm, the left side is assigned to the leading strand and the right side to the trailing strand (Figure 7f). The downfield peak at 4.80 on the leading strand plane is NOE to a hydroxyproline Cα, which is indicative of peptide A being the leading strand (Figure 7d,e). In the middle strand plane, a large peak at 4.3 ppm corresponds to the NOE to a lysine Cα‐H, inferring that this plane represents peptide B. In the trailing strand plane, a large peak at 4.03 ppm corresponds to the NOE to an arginine Cα‐H, resolving that peptide C is the trailing strand. The complete peak assignments can be found in the Supporting Information (see section “Structural characterization by NMR”).

### 2.5 3D Models of GRACE‐Generated Heterotrimers by AlphaFold3

While AlphaFold2 underperforms on predicting collagen triple helical structures, the more recently released version, AlphaFold3, which allows prediction of post‐translational modification amino acids such as hydroxyproline, has shown significant improvements in predictive accuracy, especially for multimeric proteins.^[^ ^40^ ^]^ We used AlphaFold3 to model the 3D structures of all heterotrimers generated by GRACE, varying the peptide input order to evaluate the registration. Due to the stochastic nature of AlphaFold3, each input order was replicated four times. Details can be found in Table S29 (Supporting Information). AlphaFold3's predictions on four GRACE‐generated heterotrimers showed reasonable packing of triple helical backbone and side chains (Figures S63–S67, Supporting Information). However, inconsistencies were observed in the order of peptide strands (Figure S62 and Table S29, Supporting Information). While altering the order of peptide strands may appear to be minor in sequence representation, the 3D structure and biological functionality of the triple helix are unique for each register. It should be further noted that AlphaFold3 will only generate a trimer consisting of exactly the number of peptides given to it, so there is no chance for it to explore or compare alternate compositions. For example, when given only two different collagen‐like peptides as inputs, AlphaFold3 predicted two left‐handed PPII helices coiled together into a right‐handed dimer (Figure S67, Supporting Information). In contrast, experimental results discussed in the previous sections showed that the mixture of these peptides formed a triple helix.

Given that the primary aim of AlphaFold3 is to serve as a universal model for accurately predicting 3D structures of proteins, critical aspects in experimental collagen heterotrimer synthesis may be overlooked if relying solely on this tool. While knowing that the peptides can correctly assemble into a triple helical structure is beneficial, having enough information to achieve a high specificity in experimental characterization is critical.

### 2.6 Current Limitations and Future Perspectives

Although all generated heterotrimers successfully folded into triple helices with predicted registers and a specificity of at least 13.5 °C, a discrepancy between predicted and experimental melting temperatures remains a limitation of the algorithm. In all cases, GRACE overestimated the melting temperatures of the target assemblies with deviations ranging from 6.2  to 25.4 °C (**Table** **3**). One contributing factor is the absence of resolved experimental values for all pairwise interactions. This is due in part to previous studies, which have been primarily focused on elucidating stabilizing interactions for rational design, while overlooking destabilizing interactions. Currently, many of our parameter values for pairwise interactions are assumed to be zero, although some of these interactions may destabilize the triple helix and are not accounted for. Additionally, the effects of pairwise interaction are quantified using experimentally characterized single and doubly substituted (POG)_n_ peptides’ thermal stabilities by CD. The influence of multiple, co‐existing pairwise interactions within a CMP remains unverified, as CD data does not inform the register. This limitation may be addressed as a more diverse experimental dataset is developed over time. Despite this, the consistent success of register prediction confirms that the algorithm workflow effectively captures the key features that direct triple helix self‐assembly.

**Table 3:** Comparison of predicted and experimental Tm for target assemblies.

Heterotrimer	Predicted Tm	Experimental Tm	Deviation^a)^
ABC‐1	39.7	33.5	6.2
ABC‐2	63.9	38.5	25.4
AAB‐FOGER	45.8	34.5	11.3
ABC‐FOGER	47.5	40.7	6.8

^a)^ Deviation = Predicted Tm – Experimental Tm.

GRACE is developed to be a tool for the design of de novo heterotrimeric collagen‐like peptides, helping to bridge current gaps in knowledge about collagen structure and function. One area of exploration can be understanding the effects of pairwise interactions within a chemically diverse environment, where multiple interactions co‐exist in one region of the triple helix. Additionally, the precise average helical pitch for natural collagen is not well‐defined, with fiber diffraction and crystallographic studies suggesting varying pitches.^[^ ^41^, ^42^ ^]^ Experimentally characterizing multiple chemically diverse collagen mimetic heterotrimers could shed light on the true helical packing of natural collagen, potentially revealing variations across different regions and types of collagens. These insights could possibly allow the development of a set of discrete rules for heterotrimeric CMP design, similar to those for assemblies by well‐studied peptide structures like α‐helices and β‐sheets. Additionally, the feature of GRACE, which can generate sequences with specific binding motifs, could enable controlled studies of binding interactions between heterotrimeric collagens and collagen‐binding proteins, providing insights into structure and function.

## 3 Conclusion

We have presented GRACE as the first computational method that can reproducibly generate chemically diverse heterotrimeric CMPs with high specificity. By enabling user‐defined inputs such as CMP length, composition, and thresholds for stability and specificity, GRACE offers flexibility in tailoring target assemblies. Notably, its ability to generate heterotrimers with biologically relevant sequences from user input addresses the longstanding challenge of registration control while maintaining functional constraints of the biological sequence. GRACE not only provides triple helical sequences but also predicts the specificity and stability of the target heterotrimeric assemblies, as well as all competing species. Experimental validation of four GRACE‐generated sequences, without further modifications, confirmed their successful self‐assembly into heterotrimeric triple helices. While deviations were observed between predicted and experimental melting temperatures, all computationally designed heterotrimers achieved specificity exceeding 10 °C. Our computational framework represents a step forward in advancing collagen studies and enabling the design of heterotrimeric collagen‐like peptides for diverse biomedical applications.

## 4 Experimental Section

### Genetic Algorithm Pseudocode

**Table:** 

**1. User input setup**
0 = novel heterotrimer, 1 = heterotrimer + Biological motif
If Motif:
Input motifLength (3‐15)
Input sequences for Seq1 (on leading strand), Seq2 (on middle strand), Seq3 (on trailing strand)
Validate Gly at every 3rd position; ensure positions align
Else:
Input AAB or ABC
Input sequence length (21‐40)
Ask for target Tm (30‐70 °C) and Specificity (10‐35)
**2. Initialize Genetic Algorithm Parameters with user inputs**
**3. Genetic Algorithm**
FUCTION InitialPopulationGeneration(GA parameters):
For each individual in population (populationSize):
Create empty helix with numPep peptides, each with numAA residues
If user provided a motif:
Set numAA = 2 * randomSeqLength + motifLength
For each peptide:
– Fill first randomSeqLength positions with random amino acids
– Insert user motif in the middle
– Fill last randomSeqLength positions with random amino acids
– Insert Glycine (“G”) at every 3rd position based on user motif
– Replace any excluded amino acids at Xaa/Yaa positions if applicable
Save helix to GAparameters.Helices
Else (no user motif):
For each peptide:
– Fill entire sequence randomly
– Randomly assigned GlyPos (0,1,2 positions)
– Insert Glycine at every 3rd position starting from GlyPos
– Replace any excluded amino acids at X and Y positions if applicable
Save helix to GAparameters.Helices
Return updated GAparameters
FUCTION FitnessScore(parameters, library, helixParameters):
Initialize empty lists: FitnessScore, Tm, specificity, bestRegister
Call SCEPTTr12 to compute:
– Tm (melting temp)
– specificity
– bestRegister alignment
For each sequence:
If bestRegister is [0, 1, 2]:
Assign CCRegisterScore = +50
Else:
Assign CCRegisterScore = ‐50
Set weights:
– If motif exists: a = 0.4, b = 0.4, c = 0.2
– Else: a = 0.5, b = 0.5, c = 0.0
For each sequence:
Fitness = a * specificity + b * Tm + c * CCRegisterScore

**Table:** 

Output:
– Fitness scores
– Melting temp (Tm)
– Specificity
FUNCTION Selection(Parents, fitnessScores):
Find indices of top 2 helices with highest fitness:
bestHelicesIndex = findIndicesOfTwoHighest(Parents, fitnessScores)
Save bestHelicesIndex to bestHelixID
Create new GA_Parameters bestParents:
Set population size = 2
Clear previous helices
For each peptide in numPep:
Extract sequences from:
– Best helix (index 0)
– Second‐best helix (index 1)
Save these sequences as BestHelix and secBestHelix
Add both BestHelix and secBestHelix to bestParents.Helices
Return bestParents
// EVOLUTION LOOP
Flag done = false
WHILE (not done):
IF (rounds % 100 = = 0):
Display generation, current best Tm, specificity, register
// Crossover
IF motif exists:
Parents = CrossOver_withMotif(Parents)
ELSE:
Parents = CrossOver(Parents)
// Mutation
IF motif exists:
Parents = Mutation_withMotif(Parents)
ELSE:
Parents = Mutation(Parents)
// Fitness Evaluation
Recompute fitness scores, Tm, specificity
Select best Parents for next generation
Record stats
// Stopping Criteria
IF (HighTm ≥ targetTm AND Specificity ≥ targetSpec AND registers differ)
OR (rounds ≥ 500000):
done = true
Increment rounds
END WHILE
**4. Output and Export Results**
Display:
Final best helices, sequences, Tm, specificity, registers
If motif exists, display original motif sequences
Export FitnessLandscape.csv with:
Generation, TimeElapsed, FitnessScore, Tm, Spec

### Peptide Synthesis

Peptides belonging to heterotrimers were synthesized with one ^15^N‐labeled glycine or ^13^C‐^15^N‐labeled glycine. Isotopically labeled Fmoc‐Gly‐OH was purchased from Cambridge Isotope Laboratories (CIL). Amino acid reagents Fmoc‐Asp(OtBu)‐OH, Fmoc‐Phe‐OH, Fmoc‐Gly‐OH, Fmoc‐Ile‐OH, Fmoc‐Leu‐OH, Fmoc‐Lys(Boc)‐OH, Fmoc‐Asn(Trt)‐OH, Fmoc‐Hyp(tBu)‐OH, Fmoc‐Pro‐OH, Fmoc‐Gln(Trt)‐OH, Fmoc‐Arg(Pbf)‐OH, Fmoc‐Trp(Boc)‐OH, Fmoc‐Tyr(But)‐OH and other reagents were purchased from Sigma‐Aldrich. Peptides were synthesized using standard fluorenylmethyloxycarbonyl (Fmoc)‐protected amino acids on a low‐loading Rink amide MBHA resin, resulting in the final formation of C‐terminal amidated peptides. Deprotection was achieved using a mixture of 25% v/v piperidine in dimethylformamide (DMF). The coupling reactions were done in DMF using 2‐(7‐Aza‐1H‐benzotriazole‐1‐yl)‐1,1,3,3‐tetramethyluronium hexafluorophosphate (HATU) and diisopropylethylamine (DiEA) at a ratio of 1:4:4:6 (resin: amino acids: HATU: DiEA). After the deprotection of the final residue, the N‐terminus was acetylated using acetic anhydride and DiEA in dichloromethane (DCM). The cleavage of peptides from the resin was performed in the presence of excess trifluoroacetic acid (TFA), with the addition of Milli‐Q water, triisopropylsilane (TIPS), anisole, and ethanedithiol (EDT) as scavengers. TFA was evaporated under nitrogen, and cold diethyl ether was used to triturate the peptide. The triturated peptide was then centrifuged and washed with excess ether.

### Peptide Purification

Crude peptides were dissolved in Milli‐Q water to a concentration of 15 mg mL^−1^, followed by filtration with a 0.3 µm syringe filter. The purification of peptides was performed using a Waters reversed‐phase semi‐preparative high‐performance liquid chromatography (HPLC) with a C‐18 column. The mobile phase was a gradient of Milli‐Q water with 0.05% w./v. TFA (mobile phase A) and acetonitrile with 0.05% w./v. TFA (mobile phase B). A gradient of 5%–50% B, 1.5% B/min was used for all peptide purification. The samples were roto‐evaporated to remove acetonitrile before being frozen and lyophilized with a FreeZone 4.5L Freeze Dry System. The correct mass of peptides was confirmed using a Bruker AutoFlex Speed MALDI ToF or an Agilent MSD XT Single‐Q LC‐MS. Ultra‐Performance Liquid Chromatography (UPLC) with Water Acquity was performed on a 1 mM sample to confirm the purity of the expected peptides. Characterization of all peptides can be found in the supporting information.

### Sample Preparation

The concentrations of peptides in SCEPTTr Library 1.2 and peptides in the AAB‐FOGER heterotrimer were determined by mass. The concentrations of the other GRACE‐generated peptides were determined by quantifying absorbance at 280 nm with a NanoDrop 2000c spectrophotometer. The concentration was calculated using a molar extinction coefficient of 1480 M^−1 ^cm^−1^ for tyrosine and 5540 M^−1 ^cm^−1^ for tryptophan.^[^ ^43^ ^]^ To prepare 3 mM samples, each peptide was dissolved in a 10 mM phosphate buffer and then adjusted to pH 7.0±0.2. These solutions were annealed at 85 °C for 15 min, then allowed to cool to room temperature and equilibrated at 4 °C. The equilibrating period is a minimum of 24 h for unary solutions and seven days for mixtures of peptides.

### Circular Dichroism

The circular dichroism (CD) data were collected on a Jasco J‐810 spectropolarimeter (Tokyo, Japan) equipped with a Peltier temperature controller. The samples were diluted with Milli‐Q water to concentrations of 0.03 mM for wavelength scans and 0.3 mM for melt experiments. 200 µL of peptide samples were transferred to a quartz cuvette with a path length of 1 mm. Wavelength scans were conducted between 200 and 250 nm at 5 °C. The melting curves were recorded from 5 to 65 °C, with a heating rate of 10 °C per hour, using the wavelength that corresponds to the maximum molar residue ellipticity (MRE) value of each sample. The first derivative curve was calculated using the Savitzky‐Golay smoothing algorithm. The temperature corresponding to the minimum point of the first derivatives is defined as the melting temperature (Tm). The MRE value was calculated using the equation, MRE = (θ × M) / (c × l × nr × 10), where θ is the experimental ellipticity (millidegrees), *M* is the molecular weight of the peptide (g/mol), l is the path length of the cuvette (cm), and nr is the number of amino acid residues in the peptide.

### NMR

The isotopically labeled peptides were prepared at a concentration of 2.7 mM with 9 mM phosphate buffer and 10 mM trimethylsilyl propanoic acid (TSP) in 90%H2O/10%D2O. All experiments were performed on a Bruker NEO 600 MHz High‐Performance digital NMR with a helium‐cooled inverse TCI probe. ^1^H‐^15^N HSQC characterizations were acquired using the same set of parameters, including 1024 × 128 complex points, 8 scans, and 16 dummy scans. The spectral window was set to 35 ppm for the ^15^N dimension and 16 ppm for the 1H dimension. The 3D ^1^H–^1^H–^15^N NOESY HSQC experiments were collected with 8 scans and 1024 × 32 × 128 complex points with 32 dummy scans. The spectral window for 3D experiments was 12 ppm in the ^1^H dimension and 20 ppm for the ^15^N dimension. For all experiments, the nitrogen carrier frequency was centered at 117 ppm, while the proton carrier frequency was set to match the water peak. Calibration of the 90‐degree pulse was carried out separately for each individual sample. Each dimension for each experiment was Fourier‐transformed and phase‐corrected. Raw NMR data were processed using TopSpin 4.3.0 and TopSpin 4.4.0 software (Bruker, Mass., USA).

### Homology Modelling

3D models of heterotrimers used in register determination were constructed using the crystal structure of a collagen mimetic peptide (PDB: 1K6F) as a template.^[^ ^44^ ^]^ Residue mutations and energy minimization of side chain conformations were prepared using DLPacker under the “score” option.^[^ ^45^ ^]^ Triple helical structures were rendered using PyMOL.

## Conflict of Interest

The authors declare no conflict of interest.

## Supporting information



Supporting Information

## Data Availability

Source code and parameter files are available at https://github.com/htb2m/GRACE.git.
