# Supplementary material for: De Novo Design of Specific Heterotrimeric Collagen‐Like Peptides via Genetic Algorithm
Source: Adv Sci (Weinh). 2025 Aug 18;12(39):e02377. doi: 10.1002/advs.202502377 (PMC12533406; doi:10.1002/advs.202502377)
Supplement: Supplementary file 1 — Supporting Information [file ADVS-12-e02377-s001.docx]

Supporting Information

De Novo Design of Specific Heterotrimeric Collagen-like Peptides via Genetic Algorithm

Thi H. Bui, Oluwakamisi Adetunji, Carson C. Cole, Le Tracy Yu, Caroline M. Peterson, and Jeffrey D. Hartgerink*

Departments of Chemistry and Bioengineering, Rice University, Houston, TX, USA 77005

E-mail: jdh@rice.edu

Table of Contents

[1. Refinement of SCEPTTr 35](#_Toc199348712)

[1.1. Expansion of the peptide library to make Library 1.2 36](#_Toc199348713)

[1.2. CD melting curve, derivatives, mass spectra and UPLC of new peptides in the library 51](#_Toc199348714)

[1.3. Incorporation of new parameters considering frameshifts and terminal amino acids 62](#_Toc199348715)

[1.4. SCEPTTr 1.2 optimization by a genetic algorithm 64](#_Toc199348716)

[2. Algorithms preceding GRACE 65](#_Toc199348717)

[3. GRACE performance at varying target conditions, mutation rate, crossover rate, and initial population size 68](#_Toc199348718)

[3.1. Comparison of GRACE performance at varying target melting temperatures and specificity 68](#_Toc199348719)

[3.2. Comparison of GRACE performance at varying mutation rates 71](#_Toc199348720)

[3.3. Comparison of GRACE performance at varying crossover rates 75](#_Toc199348721)

[2.4. Comparison of GRACE performance at varying initial population size 77](#_Toc199348722)

[4. Comprehensive Tm predictions of all possible assemblies from GRACE-generated peptides 81](#_Toc199348723)

[5. Mass spectra and UPLC of GRACE-generated peptides 82](#_Toc199348724)

[6. CD characterization of GRACE-generated heterotrimers 88](#_Toc199348725)

[7. Structural characterization by NMR 89](#_Toc199348726)

[7.1. Structural analysis of competing assemblies at 10 °C 89](#_Toc199348727)

[7.2. ABC-1 91](#_Toc199348728)

[7.3. ABC-2 101](#_Toc199348729)

[7.4. AAB-FOGER 106](#_Toc199348730)

[7.5. ABC-FOGER 107](#_Toc199348731)

[8. Three-dimensional models of GRACE-generated heterotrimers by AlphaFold3 118](#_Toc199348732)

[9. GRACE example runs 123](#_Toc199348733)

# **1. Refinement of SCEPTTr**


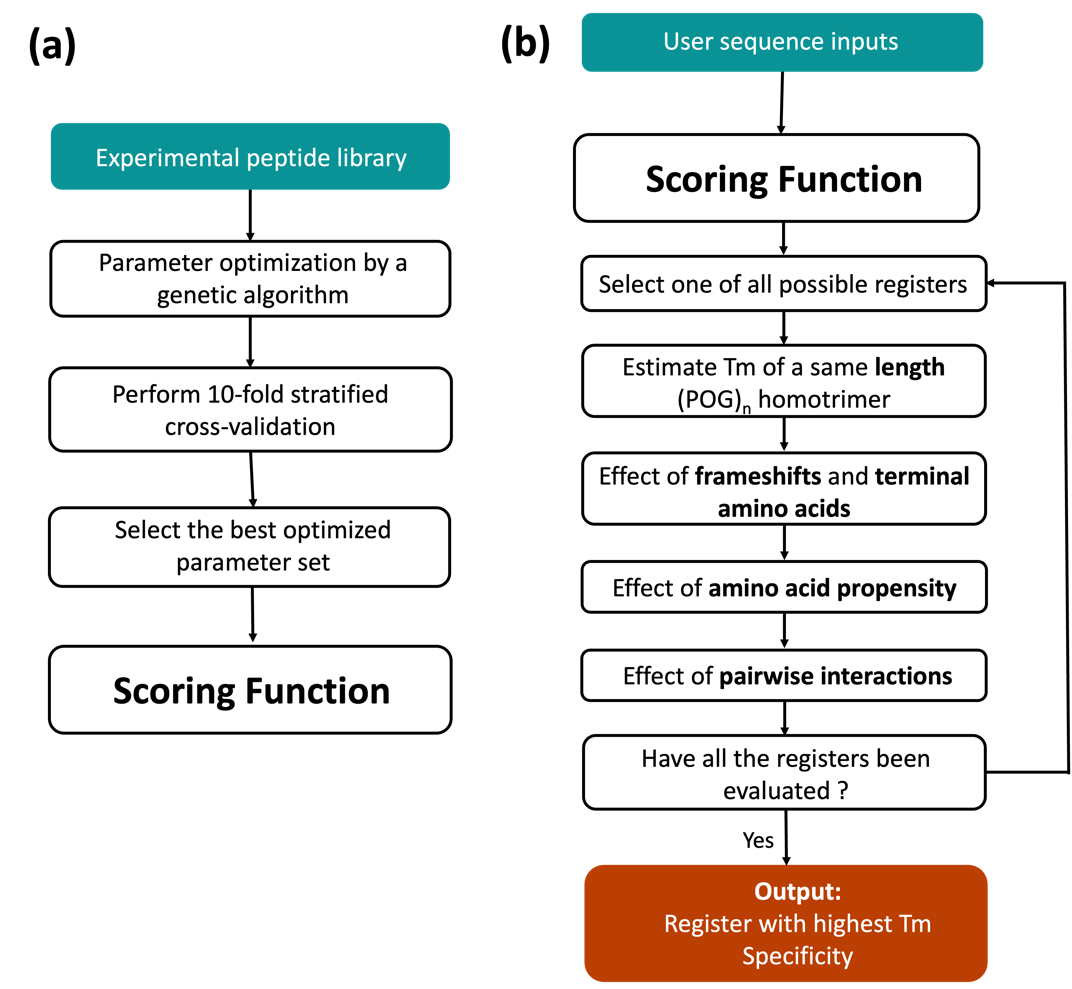


**Figure S1.** Flow chart summarizing the development and functionality of SCEPTTr 1.2, (a) Scoring parameter optimization process, (b) Triple helix scoring process.

## **1.1. Expansion of the peptide library to make Library 1.2**

Following the last publication on SCEPTTr in 2021, there have been several peptides with diverse features synthesized by our lab and other research groups. Thus, it is necessary to expand the SCEPTTr library to encompass the characteristics of these new collagen-like peptides​. To achieve a comprehensive and reliable dataset, all selected peptides were characterized using a uniform set of methods and conditions, including a pH of approximately 7 and a heating rate of 10 °C per hour for melting experiments. This standardization aims to mitigate potential experimental variations that could influence the final melting temperature, thereby minimizing bias in our scoring function and ensuring a more accurate assessment. Additionally, any duplications were either removed from the database or replaced with their averaging values to allow the optimal performance of our scoring function. As a result, 113 new peptides were curated and added to the peptide library to make Library 1.2.

**Table S1.** Library 1.2.

Nterm: ‘ac’ for acetylated N-terminal, and ‘n’ for free N-terminal; Cterm: ‘am’ for amidated C-terminal and ‘c’ for free C-terminal.

† Experimental melting temperature is the average of values reported in mentioned references.

* Peptides were characterized for this publication. See experimental data in the next section.

| Id | num Pep | num AA | N term | C term | Exp. Temp | Peptide sequence | SCEPTTr 1.0 | SCEPTTr 1.1 | SCEPTTr 1.2 |
| --- | --- | --- | --- | --- | --- | --- | --- | --- | --- |
| 1 | 1 | 21 | ac | am | 43 | POGPOGPOGPOGPOGPOGPOG^[46]^ | 40.7 | 39.5 | 37.0 |
| 2 | 1 | 21 | ac | c | 40 | POGPOGPOGPOGPOGPOGPOG^[46]^ | 36.4 | 39.5 | 36.4 |
| 3 | 1 | 21 | n | am | 35 | POGPOGPOGPOGPOGPOGPOG^[46]^ | 33.8 | 29.5 | 37.1 |
| 4 | 1 | 21 | n | c | 32 | POGPOGPOGPOGPOGPOGPOG^[46]^ | 29.0 | 29.5 | 32.2 |
| 5 | 1 | 24 | ac | am | 50.5 | POGPOGPOGPOGPOGPOGPOGPOG^[47]^ | 51.1 | 50.5 | 47.6 |
| 6 | 1 | 30 | n | c | 29.5 | PPGPPGPPGPPGPPGPPGPPGPPGPPGPPG^[48]^ | 32.7 | 34.5 | 31.4 |
| 7 | 1 | 30 | ac | am | 67.5 | POGPOGPOGPOGPOGPOGPOGPOGPOGPOG^[49]^ | 66.9 | 67.1 | 65.1 |
| 9 | 1 | 24 | ac | am | 40.5 | POGPOGPOGPKGPOGPOGPOGPOG^[19]^ | 41.9 | 41.5 | 39.2 |
| 10 | 1 | 24 | ac | am | 47 | POGPOGPOGPRGPOGPOGPOGPOG^[19]^ | 47.5 | 46.8 | 45.2 |
| 11 | 1 | 24 | ac | am | 38 | POGPOGPOGPHGPOGPOGPOGPOG^[19]^ | 37.9 | 37.4 | 37.5 |
| 12 | 1 | 24 | ac | am | 47 | POGPOGPOGPOGPPGPOGPOGPOG^[19]^ | 48.0 | 47.3 | 44.2 |
| 13 | 1 | 24 | ac | am | 45 | POGPOGPOGPQGPOGPOGPOGPOG^[19]^ | 46.6 | 45.1 | 44.6 |
| 14 | 1 | 24 | ac | am | 34 | POGPOGPOGPNGPOGPOGPOGPOG^[19]^ | 35.6 | 34.9 | 32.8 |
| 15 | 1 | 24 | ac | am | 44 | POGPOGPOGPAGPOGPOGPOGPOG^[19]^ | 44.8 | 43.4 | 39.9 |
| 16 | 1 | 24 | ac | am | 31 | POGPOGPOGPFGPOGPOGPOGPOG^[19]^ | 32.7 | 32.7 | 29.2 |
| 17 | 1 | 24 | ac | am | 43.5 | POGPOGPOGPVGPOGPOGPOGPOG^[19]^ | 43.5 | 43.9 | 41.5 |
| 18 | 1 | 24 | ac | am | 36 | POGPOGPOGPLGPOGPOGPOGPOG^[19]^ | 37.8 | 37.1 | 32.6 |
| 19 | 1 | 24 | ac | am | 44 | POGPOGPOGPOGSOGPOGPOGPOG^[47]^ | 43.3 | 43.0 | 41.9 |
| 20 | 1 | 24 | ac | am | 39.5 | POGPOGPOGPOGTOGPOGPOGPOG^[47]^ | 40.2 | 40.3 | 38.5 |
| 21 | 1 | 24 | ac | am | 45.5 | POGPOGPOGPOGZOGPOGPOGPOG^[47]^ | 44.7 | 44.0 | 42.9 |
| 22 | 1 | 24 | ac | am | 46.5 | POGPOGPOGPOGJOGPOGPOGPOG^[47]^ | 46.8 | 45.0 | 45.7 |
| 23 | 1 | 24 | ac | am | 41.5 | POGPOGPOGPOGDOGPOGPOGPOG^[19]^ | 43.5 | 42.4 | 42.2 |
| 24 | 1 | 24 | ac | am | 44.5 | POGPOGPOGPOGEOGPOGPOGPOG^[19]^ | 45.9 | 45.1 | 43.1 |
| 25 | 1 | 24 | ac | am | 37 | POGPOGPOGPOGFOGPOGPOGPOG^[19]^ | 37.9 | 38.5 | 36.7 |
| 26 | 1 | 24 | ac | am | 33.5 | POGPOGPOGPOGWOGPOGPOGPOG^[19]^ | 33.8 | 33.0 | 31.9 |
| 27 | 1 | 24 | ac | am | 45 | POGPOGPOGPOGAOGPOGPOGPOG^[19]^ | 45.9 | 44.2 | 41.1 |
| 28 | 1 | 24 | ac | am | 43.5 | POGPOGPOGPOGLOGPOGPOGPOG^[19]^ | 45.2 | 44.5 | 42.2 |
| 29 | 1 | 24 | ac | am | 41.5 | POGPOGPOGPOGVOGPOGPOGPOG^[19]^ | 41.3 | 40.7 | 37.5 |
| 30 | 1 | 24 | ac | am | 32.5 | POGPOGPOGPQGFOGPOGPOGPOG^[19]^ | 36.7 | 33.2 | 35.7 |
| 31 | 1 | 24 | ac | am | 21.5 | POGPOGPOGPNGFOGPOGPOGPOG^[19]^ | 30.6 | 22.5 | 23.2 |
| 32 | 1 | 24 | ac | am | 30 | POGPOGPOGPKGTOGPOGPOGPOG^[47]^ | 30.9 | 30.4 | 29.3 |
| 33 | 1 | 24 | ac | am | 44 | POGPOGPOGPKGEOGPOGPOGPOG^[19]^ | 45.0 | 44.3 | 40.2 |
| 34 | 1 | 24 | ac | am | 47 | POGPOGPOGPKGDOGPOGPOGPOG^[19]^ | 46.5 | 45.7 | 41.8 |
| 35 | 1 | 24 | ac | am | 36.5 | POGPOGPOGPKGSOGPOGPOGPOG^[47]^ | 35.8 | 37.4 | 33.0 |
| 36 | 1 | 24 | ac | am | 22.5 | POGPOGPOGPHGWOGPOGPOGPOG^[19]^ | 22.0 | 21.7 | 20.7 |
| 39 | 1 | 24 | ac | am | 42 | POGPOGPOGPRGDOGPOGPOGPOG^[19]^ | 42.8 | 42.7 | 41.2 |
| 40 | 1 | 24 | ac | am | 42.5 | POGPOGPOGPRGEOGPOGPOGPOG^[19]^ | 44.1 | 42.4 | 42.0 |
| 41 | 1 | 24 | ac | am | 49.5 | POGPOGPOGPKGZOGPOGPOGPOG^[47]^ | 50.1 | 49.8 | 48.1 |
| 42 | 1 | 24 | ac | am | 38.5 | POGPOGPOGPKGJOGPOGPOGPOG^[47]^ | 38.2 | 39.0 | 35.8 |
| 43 | 1 | 24 | ac | am | 36.5 | POGPOGPOGPAGAOGPOGPOGPOG^[19]^ | 35.0 | 36.0 | 34.2 |
| 44 | 1 | 24 | ac | am | 29 | POGPOGPOGPAGFOGPOGPOGPOG^[19]^ | 28.1 | 26.8 | 28.0 |
| 45 | 1 | 24 | ac | am | 33.5 | POGPOGPOGPAGVOGPOGPOGPOG^[19]^ | 32.7 | 34.0 | 32.3 |
| 46 | 1 | 24 | ac | am | 34.4 | POGPOGPOGPAGLOGPOGPOGPOG^[19]^ | 36.1 | 35.2 | 35.5 |
| 47 | 1 | 24 | ac | am | 25.6 | POGPOGPOGPFGAOGPOGPOGPOG^[19]^ | 26.5 | 25.6 | 23.9 |
| 48 | 1 | 24 | ac | am | 19.5 | POGPOGPOGPFGFOGPOGPOGPOG^[19]^ | 20.3 | 19.5 | 18.3 |
| 49 | 1 | 24 | ac | am | 23.5 | POGPOGPOGPFGLOGPOGPOGPOG^[19]^ | 23.4 | 24.0 | 24.1 |
| 50 | 1 | 24 | ac | am | 18 | POGPOGPOGPFGVOGPOGPOGPOG^[19]^ | 18.1 | 18.0 | 19.0 |
| 51 | 1 | 24 | ac | am | 29 | POGPOGPOGPLGAOGPOGPOGPOG^[19]^ | 28.8 | 28.4 | 28.8 |
| 52 | 1 | 24 | ac | am | 24 | POGPOGPOGPLGFOGPOGPOGPOG^[19]^ | 24.1 | 24.7 | 22.9 |
| 53 | 1 | 24 | ac | am | 28.5 | POGPOGPOGPLGLOGPOGPOGPOG^[19]^ | 30.3 | 29.9 | 29.5 |
| 54 | 1 | 24 | ac | am | 19.5 | POGPOGPOGPLGVOGPOGPOGPOG^[19]^ | 18.8 | 18.9 | 23.0 |
| 55 | 1 | 24 | ac | am | 37.5 | POGPOGPOGPVGAOGPOGPOGPOG^[19]^ | 36.7 | 35.6 | 36.6 |
| 56 | 1 | 24 | ac | am | 30.1 | POGPOGPOGPVGFOGPOGPOGPOG^[19]^ | 29.8 | 29.1 | 30.9 |
| 57 | 1 | 24 | ac | am | 34.5 | POGPOGPOGPVGLOGPOGPOGPOG^[19]^ | 33.5 | 33.8 | 37.3 |
| 58 | 1 | 24 | ac | am | 30.6 | POGPOGPOGPVGVOGPOGPOGPOG^[19]^ | 32.7 | 32.0 | 32.1 |
| 59 | 1 | 24 | ac | am | 24.5 | POGPOGPOGPOGFQGPOGPOGPOG^[19]^ | 39.5 | 30.2 | 24.7 |
| 60 | 1 | 24 | ac | am | 19.5 | POGPOGPOGPOGFNGPOGPOGPOG^[19]^ | 22.4 | 20.5 | 21.5 |
| 61 | 1 | 24 | ac | am | 22.5 | POGPOGPOGPOGFAGPOGPOGPOG^[19]^ | 28.6 | 26.8 | 20.9 |
| 63 | 1 | 24 | ac | am | 28.4 | POGPOGPOGPKGYOGPOGPOGPOG^[17]^ | 27.4 | 28.9 | 29.0 |
| 64 | 1 | 24 | ac | am | 26 | POGPOGPOGPKGWOGPOGPOGPOG^[17]^ | 25.4 | 26.1 | 23.7 |
| 66 | 1 | 24 | ac | am | 41.1 | POGPOGPOGPRGYOGPOGPOGPOG^[17]^ | 40.4 | 41.9 | 35.2 |
| 67 | 1 | 24 | ac | am | 39.2 | POGPOGPOGPRGWOGPOGPOGPOG^[17]^ | 40.1 | 38.4 | 34.6 |
| 68 | 1 | 24 | ac | am | 21.5 | POGPOGPOGPFGROGPOGPOGPOG^[17]^ | 22.4 | 21.2 | 21.8 |
| 69 | 1 | 24 | ac | am | 14.5 | POGPOGPOGWHGPOGPOGPOGPOG^[19]^ | 17.8 | 17.5 | 15.1 |
| 70 | 1 | 24 | ac | am | 31 | POGPOGPOGPOGFPGPOGPOGPOG^[19]^ | 30.3 | 31.3 | 30.1 |
| 71 | 1 | 24 | ac | am | 39.5 | POGPOGPOGPOGEPGPOGPOGPOG^[19]^ | 39.1 | 41.0 | 37.8 |
| 72 | 1 | 24 | ac | am | 36.5 | POGPOGPOGPOGDPGPOGPOGPOG^[19]^ | 38.2 | 36.0 | 38.2 |
| 73 | 1 | 24 | ac | am | 29.5 | POGPOGPOGPOGFRGPOGPOGPOG^[19]^ | 34.0 | 34.0 | 29.1 |
| 74 | 1 | 24 | ac | am | 42.5 | POGPOGPOGPOGERGPOGPOGPOG^[19]^ | 44.1 | 43.5 | 42.2 |
| 75 | 1 | 24 | ac | am | 32 | POGPOGPOGPOGDKGPOGPOGPOG^[19]^ | 36.4 | 36.0 | 34.0 |
| 76 | 1 | 24 | ac | am | 37 | POGPOGPOGPOGEKGPOGPOGPOG^[19]^ | 37.7 | 37.8 | 36.7 |
| 77 | 1 | 24 | ac | am | 22 | POGPOGPOGPOGFKGPOGPOGPOG^[19]^ | 27.6 | 28.2 | 23.6 |
| 78 | 1 | 24 | ac | am | 38 | POGPOGPOGPOGDRGPOGPOGPOG^[19]^ | 38.8 | 38.9 | 39.3 |
| 79 | 1 | 30 | ac | am | 16 | PRGEPGPRGERGPPGPPGERGPPGEPGEPG^[19]^ | 16.9 | 17.1 | 16.1 |
| 80 | 1 | 30 | ac | am | 43 | POGEOGPOGEOGPOGEOGPOGEOGPOGEOG^[51]^ | 42.4 | 42.9 | 42.2 |
| 81 | 1 | 30 | ac | am | 35.5 | POGDOGPOGDOGPOGDOGPOGDOGPOGDOG^[51]^ | 30.9 | 30.9 | 37.3 |
| 82 | 1 | 27 | ac | am | 49 | POGPRGPOGPRGPOGPRGPOGPRGPOG^[52]^ | 45.4 | 45.2 | 47.1 |
| 83 | 1 | 30 | n | c | 46 | POGPOGPOGPOGEKGPOGPOGPOGPOGPOG^[53]^ | 46.9 | 48.7 | 49.5 |
| 85 | 1 | 30 | n | c | 41.3 | GPOGPOGPOGQOGLOGLOGPOGPOGPOGPO^[54]^ | 8.0 | 44.1 | 42.8 |
| 86 | 1 | 33 | n | c | 48 | FGPOGPOGPOGQOGLOGLOGPOGPOGPOGPOGY^[54]^ | 37.9 | 40.5 | 44.5 |
| 87 | 1 | 30 | ac | am | 51.5 | POGPOGEOGPOGEOGPOGEOGPOGPOGPOG^[49]^ | 51.3 | 50.9 | 51.7 |
| 88 | 1 | 30 | ac | am | 58.5 | POGPOGPRGPOGPRGPOGPRGPOGPOGPOG^[49]^ | 56.1 | 56.2 | 58.0 |
| 89 | 1 | 30 | ac | am | 46.5 | EOGPOGEOGPOGEOGPOGEOGPOGEOGPOG^[49]^ | 46.0 | 44.6 | 45.2 |
| 90 | 1 | 30 | ac | am | 55.5 | PRGPOGPRGPOGPRGPOGPRGPOGPRGPOG^[49]^ | 52.4 | 52.0 | 54.4 |
| 91 | 1 | 30 | n | c | 38 | PKGDOGPKGDOGPKGDOGPKGDOGPKGDOG^[19]^ | 40.8 | 41.0 | 36.1 |
| 92 | 1 | 30 | ac | am | 23.6 | GPPGPPGPKGDPGPKGDPGPKGDPGPPGGY^[33]^ | 24.6 | 23.4 | 29.6 |
| 93 | 1 | 30 | ac | am | 50 | POGPOGPOGPOGPOGLIGPOGPOGPOGPOG^[55]^ | 50.6 | 48.0 | 49.9 |
| 94 | 1 | 30 | ac | am | 41 | POGPOGPOGPOGPIGLIGPOGPOGPOGPOG^[55]^ | 42.2 | 38.6 | 40.7 |
| 95 | 1 | 30 | ac | am | 33 | POGPOGPOGPOGLIGLIGPOGPOGPOGPOG^[55]^ | 32.8 | 32.2 | 33.3 |
| 96 | 1 | 30 | ac | am | 24 | POGPOGPOGPIGLIGLIGPOGPOGPOGPOG^[55]^ | 24.4 | 23.1 | 23.9 |
| 97 | 1 | 30 | ac | am | 15 | POGPOGPOGLIGLIGLIGPOGPOGPOGPOG^[55]^ | 15.0 | 16.8 | 16.5 |
| 98 | 1 | 30 | ac | am | 24 | POGPOGPOGQQGLIGLIGPOGPOGPOGPOG^[55]^ | 24.1 | 23.3 | 24.5 |
| 99 | 2 | 30 | ac | am | 41 | PRGPRGPRGPRGPRGPRGPRGPRGPRGPRG^[49]^ EOGEOGEOGEOGEOGEOGEOGEOGEOGEOG | 40.1 | 40.0 | 43.9 |
| 100 | 2 | 30 | ac | am | 36 | PKGPKGPKGPKGPKGPKGPKGPKGPKGPKG^[56]^ DOGDOGDOGDOGDOGDOGDOGDOGDOGDOG | 41.2 | 39.7 | 42.4 |
| 101 | 2 | 30 | ac | am | 38 | PKGPKGPKGPKGPKGPKGPKGPKGPKGPKG^[56]^ EOGEOGEOGEOGEOGEOGEOGEOGEOGEOG | 41.0 | 39.9 | 39.7 |
| 102 | 2 | 30 | ac | am | 42 | PRGPRGPRGPRGPRGPRGPRGPRGPRGPRG^[56]^ DOGDOGDOGDOGDOGDOGDOGDOGDOGDOG | 37.0 | 38.2 | 38.6 |
| 103 | 2 | 30 | ac | am | 56 | PRGPRGPRGPRGPRGPRGPRGPRGPRGPRG^[57]^ EOGPOGEOGPOGEOGPOGEOGPOGEOGPOG | 47.9 | 47.5 | 48.5 |
| 104 | 2 | 30 | ac | am | 45 | PKGPOGPKGPOGPKGPOGPKGPOGPKGPOG^[51]^ EOGEOGEOGEOGEOGEOGEOGEOGEOGEOG | 44.5 | 43.4 | 44.2 |
| 105 | 2 | 30 | ac | am | 46 | PKGPOGPKGPOGPKGPOGPKGPOGPKGPOG^[51]^ DOGDOGDOGDOGDOGDOGDOGDOGDOGDOG | 44.8 | 43.6 | 44.5 |
| 106 | 2 | 30 | ac | am | 47 | POGEOGPOGEOGPOGEOGPOGEOGPOGEOG^[51]^ PRGPRGPRGPRGPRGPRGPRGPRGPRGPRG | 46.3 | 46.8 | 46.6 |
| 107 | 2 | 30 | ac | am | 52.5 | PRGPOGPRGPOGPRGPOGPRGPOGPRGPOG^[51]^ EOGEOGEOGEOGEOGEOGEOGEOGEOGEOG | 52.4 | 47.5 | 49.6 |
| 108 | 2 | 27 | ac | am | 47 | POGPRGPOGPRGPOGPRGPOGPRGPOG^[52]^ POGFOGPOGFOGPOGFOGPOGFOGPOG | 45.4 | 43.8 | 47.4 |
| 109 | 2 | 27 | ac | am | 47 | POGPRGPOGPRGPOGPRGPOGPRGPOG^[52]^ POGYOGPOGYOGPOGYOGPOGYOGPOG | 46.3 | 46.1 | 39.2 |
| 113 | 2 | 30 | ac | am | 56.5 | POGPOGPRGPOGPRGPOGPRGPOGPOGPOG^[49]^ POGPOGEOGPOGEOGPOGEOGPOGPOGPOG | 58.2 | 58.2 | 58.1 |
| 114 | 2 | 30 | ac | am | 54 | PRGPOGPRGPOGPRGPOGPRGPOGPRGPOG^[49]^ EOGPOGEOGPOGEOGPOGEOGPOGEOGPOG | 55.2 | 54.8 | 55.6 |
| 115 | 2 | 30 | n | c | 50 | PKGDOGPKGDOGPKGDOGPKGDOGPKGDOG^[19]^ POGDKGPOGDKGPOGDKGPOGDKGPOGDKG | 53.5 | 54.2 | 51.8 |
| 116 | 2 | 30 | n | c | 52 | PKGDOGPKGDOGPKGDOGPKGDOGPKGDOG^[19]^ DKGPOGDKGPOGDKGPOGDKGPOGDKGPOG | 55.5 | 55.1 | 53.1 |
| 117 | 2 | 30 | ac | am | 44 | POGDOGPOGDOGPOGDOGPOGDOGPOGDOG^[51]^ PKGPKGPKGPKGPKGPKGPKGPKGPKGPKG | 41.2 | 42.0 | 42.8 |
| 118 | 2 | 30 | ac | am | 40 | POGEOGPOGEOGPOGEOGPOGEOGPOGEOG^[51]^ PKGPKGPKGPKGPKGPKGPKGPKGPKGPKG | 42.4 | 42.0 | 40.0 |
| 119 | 2 | 30 | ac | am | 43 | EOGPOGEOGPOGEOGPOGEOGPOGEOGPOG^[19]^ PKGPKGPKGPKGPKGPKGPKGPKGPKGPKG | 46.0 | 42.0 | 40.2 |
| 120 | 2 | 30 | ac | am | 39.5 | POGDOGPOGDOGPOGDOGPOGDOGPOGDOG^[51]^ PRGPRGPRGPRGPRGPRGPRGPRGPRGPRG | 40.6 | 41.8 | 44.8 |
| 121 | 2 | 30 | ac | am | 44 | PRGPOGPRGPOGPRGPOGPRGPOGPRGPOG^[51]^ DOGDOGDOGDOGDOGDOGDOGDOGDOGDOG | 52.4 | 43.0 | 43.9 |
| 122 | 2 | 30 | ac | am | 33.5 | KOGKOGKOGKOGKOGKOGKOGKOGKOGKOG^[58]^ PEGPEGPEGPEGPEGPEGPEGPEGPEGPEG | 31.6 | 31.1 | 29.8 |
| 123 | 2 | 30 | ac | am | 21 | KOGKOGKOGKOGKOGKOGKOGKOGKOGKOG^[58]^ PDGPDGPDGPDGPDGPDGPDGPDGPDGPDG | 27.6 | 27.2 | 32.0 |
| 124 | 2 | 30 | ac | am | 36 | GPPGPPGPKGDPGPKGDPGPKGDPGPPGGY^[33]^ GPPGPKGPPGDKGPPGDKGPPGDPGPPGGY | 36.6 | 35.3 | 33.1 |
| 125 | 2 | 30 | n | c | 13 | PKGLKGPKGPKGPKGPKGPKGPKGPKGLIG^[19]^ DOGDOGDOGLOGDIGDIGDOGDOGDOGDOG | 13.9 | 13.9 | 16.7 |
| 126 | 3 | 30 | ac | am | 39.5 | PKGPKGFOGPOGFKGFKGPKGPOGFKGPOG^[19]^ PKGDOGDKGPOGPPGDKGDOGDKGPKGDOG PRGEPGPRGERGPPGPPGERGPPGEPGEPG | 45.5 | 44.7 | 41.4 |
| 127 | 3 | 30 | ac | am | 33.5 | PKGPKGFOGPOGFKGFKGPKGPOGFKGPOG^[19]^ PKGDOGDKGPOGPPGDKGDOGDKGPKGDOG PQGEPGPQGEQGPPGPPGEQGPPGEPGEPG | 27.8 | 35.4 | 36.5 |
| 128 | 3 | 30 | ac | am | 24 | PKGPKGFOGPOGFKGFKGPKGPOGFKGPOG^[19]^ PKGDOGDKGPOGPPGDKGDOGDKGPKGDOG PAGEPGPAGEAGPPGPPGEAGPPGEPGEPG | 25.6 | 25.2 | 24.6 |
| 131 | 3 | 30 | n | c | 24 | DOGDOGDOGPKGPKGPKGPKGPKGKOGPDG^[59]^ KOGPDGPDGPDGDOGPDGDOGDOGDOGPDG PKGKOGKOGKOGKOGKOGPDGPDGPKGKOG | 25.9 | 26.3 | 28.5 |
| 132 | 3 | 30 | n | c | 15 | KOGPDGPDGPKGKOGPKGKOGKOGKOGKOG^[59]^ KOGPDGPDGPDGDOGPDGDOGDOGDOGPDG PKGKOGKOGKOGKOGKOGPDGPDGPKGKOG | 16.4 | 14.9 | 19.3 |
| 133 | 3 | 30 | n | c | 11 | KOGPDGPDGPKGKOGPKGKOGKOGKOGKOG^[59]^ PKGKOGKOGKOGKOGKOGPDGPDGPKGKOG PDGDOGDOGDOGPDGKOGPDGPDGPDGDOG | 16.5 | 15.8 | 18.0 |
| 134 | 3 | 30 | n | c | 24 | PKGPKGPKGPKGKOGPDGDOGDOGDOGPKG^[59]^ PDGDOGDOGDOGPDGKOGPDGPDGPDGDOG KOGPDGPDGPKGKOGPKGKOGKOGKOGKOG | 24.0 | 20.4 | 24.1 |
| 136 | 3 | 30 | n | c | 17.6 | KOGPDGPDGPDGDOGPDGDOGDOGDOGPDG^[59]^ PKGKOGKOGKOGKOGKOGPDGPDGPKGKOG DOGPKGPKGPKGPKGPKGKOGPDGDOGDOG | 21.8 | 21.2 | 21.6 |
| 137 | 3 | 30 | n | c | 24 | KOGPDGPDGPDGDOGPDGDOGDOGDOGPDG^[59]^ PKGKOGKOGKOGKOGKOGPDGPDGPKGKOG DOGDOGPKGPKGPKGPKGPKGKOGPDGDOG | 29.9 | 29.0 | 30.6 |
| 138 | 3 | 30 | n | c | 19 | PDGPDGKOGDOGDOGDOGDOGPKGPKGKOG^[60]^ PKGKOGPOGPDGPDGPDGPDGDOGDOGDOG DOGDOGPKGPKGKOGKOGPDGPDGPDGKOG | 20.8 | 17.6 | 19.0 |
| 139 | 3 | 30 | n | c | 18.5 | PDGPDGPDGDOGDOGDOGPKGPKGPKGKOG^[60]^ PDGDOGDOGPKGKOGKOGPDGPDGKOGPDG PKGKOGPDGPDGPDGDOGDOGDOGDOGDOG | 17.3 | 13.9 | 19.3 |
| 140 | 3 | 30 | n | c | 36 | PKGLKGPKGPKGPKGPKGPKGPKGPKGLIG^[19]^ DOGDOGDOGLOGDIGDIGDOGDOGDOGDOG PIGPOGPOGPOGPOGLOGLOGPIGPOGPOG | 41.2 | 34.5 | 32.2 |
| 143 | 1 | 24 | ac | am | 29.4 | GPOGPOGPOGKDGKDGPOGPOGPO^[61]^ | 26.0 | 25.9 | 28.0 |
| 144 | 1 | 30 | n | c | 26 | GKOGEOGPKGDAGAOGAOGPOGPOGPOGPO^[53]^ | 23.8 | 29.2 | 28.3 |
| 145 | 1 | 24 | ac | am | 42.9 | GPOGPOGPOGEOGPOGPOGPOGPO^[62]^ | 42.2 | 42.4 | 43.0 |
| 148 | 1 | 24 | ac | am | 41.5 | GPOGPOGPOGKOGPOGPOGPOGPO^[62]^ | 42.8 | 43.2 | 43.1 |
| 149 | 1 | 24 | ac | am | 40.6 | GPOGPOGPOGROGPOGPOGPOGPO^[62]^ | 41.8 | 41.2 | 39.7 |
| 150 | 1 | 24 | ac | am | 40.4 | GPOGPOGPOGQOGPOGPOGPOGPO^[13]^ | 42.0 | 42.4 | 40.0 |
| 151 | 1 | 24 | ac | am | 40.1 | GPOGPOGPOGDOGPOGPOGPOGPO^[62]^ | 39.8 | 39.7 | 42.0 |
| 152 | 1 | 24 | ac | am | 39 | GPOGPOGPOGLOGPOGPOGPOGPO^[63]^ | 41.5 | 41.9 | 42.1 |
| 153 | 1 | 24 | ac | am | 38.9 | GPOGPOGPOGVOGPOGPOGPOGPO^[13]^ | 37.6 | 38.1 | 37.4 |
| 154 | 1 | 24 | ac | am | 38.6 | GPOGPOGPOGMOGPOGPOGPOGPO^[13]^ | 27.8 | 40.6 | 40.2 |
| 155 | 1 | 24 | ac | am | 38.4 | GPOGPOGPOGIOGPOGPOGPOGPO^[13]^ | 39.6 | 40.4 | 40.1 |
| 156 | 1 | 24 | ac | am | 38.3 | GPOGPOGPOGNOGPOGPOGPOGPO^[13]^ | 37.7 | 37.7 | 40.2 |
| 157 | 1 | 24 | ac | am | 38 | GPOGPOGPOGSOGPOGPOGPOGPO^[64]^ | 39.6 | 40.4 | 41.8 |
| 158 | 1 | 24 | ac | am | 36.5 | GPOGPOGPOGHOGPOGPOGPOGPO^[13]^ | 35.6 | 35.7 | 36.6 |
| 159 | 1 | 24 | ac | am | 36.2 | GPOGPOGPOGTOGPOGPOGPOGPO^[13]^ | 36.6 | 37.7 | 38.4 |
| 160 | 1 | 24 | ac | am | 36.1 | GPOGPOGPOGCOGPOGPOGPOGPO^[13]^ | 36.9 | 35.7 | 35.8 |
| 161 | 1 | 24 | ac | am | 34.3 | GPOGPOGPOGYOGPOGPOGPOGPO^[13]^ | 35.1 | 36.0 | 31.0 |
| 162 | 1 | 24 | ac | am | 33.5 | GPOGPOGPOGFOGPOGPOGPOGPO^[63]^ | 34.2 | 35.9 | 36.5 |
| 163 | 1 | 24 | ac | am | 33.2 | GPOGPOGPOGGOGPOGPOGPOGPO^[50]^ | 34.9 | 35.2 | 35.8 |
| 164 | 1 | 24 | ac | am | 31.9 | GPOGPOGPOGWOGPOGPOGPOGPO^[13]^ | 30.1 | 30.4 | 31.8 |
| 167 | 1 | 24 | ac | am | 42.6 | GPOGPOGPOGPMGPOGPOGPOGPO^[13]^ | 42.7 | 42.3 | 45.2 |
| 168 | 1 | 24 | ac | am | 41.5 | GPOGPOGPOGPIGPOGPOGPOGPO^[13]^ | 40.4 | 40.6 | 39.7 |
| 169 | 1 | 24 | ac | am | 41.3 | GPOGPOGPOGPQGPOGPOGPOGPO^[13]^ | 42.9 | 42.5 | 44.5 |
| 172 | 1 | 24 | ac | am | 40 | GPOGPOGPOGPVGPOGPOGPOGPO^[13]^ | 39.9 | 41.3 | 41.4 |
| 173 | 1 | 24 | ac | am | 39.7 | GPOGPOGPOGPEGPOGPOGPOGPO^[62]^ | 40.7 | 40.9 | 41.3 |
| 174 | 1 | 24 | ac | am | 39.7 | GPOGPOGPOGPTGPOGPOGPOGPO^[13]^ | 38.9 | 41.7 | 39.7 |
| 175 | 1 | 24 | ac | am | 37.7 | GPOGPOGPOGPCGPOGPOGPOGPO^[13]^ | 38.2 | 38.5 | 37.3 |
| 176 | 1 | 24 | ac | am | 36.8 | GPOGPOGPOGPKGPOGPOGPOGPO^[62]^ | 38.2 | 38.9 | 39.0 |
| 177 | 1 | 24 | ac | am | 35.7 | GPOGPOGPOGPHGPOGPOGPOGPO^[13]^ | 34.2 | 34.8 | 37.4 |
| 178 | 1 | 24 | ac | am | 35 | GPOGPOGPOGPSGPOGPOGPOGPO^[64]^ | 36.0 | 37.0 | 41.7 |
| 181 | 1 | 24 | ac | am | 32.7 | GPOGPOGPOGPGGPOGPOGPOGPO^[13]^ | 34.3 | 34.7 | 36.4 |
| 182 | 1 | 24 | ac | am | 32.7 | GPOGPOGPOGPLGPOGPOGPOGPO^[63]^ | 34.2 | 34.5 | 32.5 |
| 183 | 1 | 24 | ac | am | 30.3 | GPOGPOGPOGPNGPOGPOGPOGPO^[13]^ | 31.9 | 32.3 | 32.6 |
| 184 | 1 | 24 | ac | am | 30.2 | GPOGPOGPOGPYGPOGPOGPOGPO^[13]^ | 31.6 | 32.2 | 35.2 |
| 185 | 1 | 24 | ac | am | 28.3 | GPOGPOGPOGPFGPOGPOGPOGPO^[63]^ | 29.0 | 30.1 | 29.1 |
| 186 | 1 | 24 | ac | am | 26.1 | GPOGPOGPOGPWGPOGPOGPOGPO^[13]^ | 27.1 | 28.1 | 27.0 |
| 187 | 1 | 24 | ac | am | 40.4 | GPOGPOGPOGERGPOGPOGPOGPO^[62]^ | 40.4 | 40.9 | 42.0 |
| 188 | 1 | 24 | ac | am | 39.5 | GPOGPOGPOGQRGPOGPOGPOGPO^[16]^ | 39.2 | 40.8 | 39.2 |
| 191 | 1 | 24 | ac | am | 38.9 | GPOGPOGPOGKQGPOGPOGPOGPO^[16]^ | 34.7 | 40.1 | 38.5 |
| 192 | 1 | 24 | ac | am | 38.2 | GPOGPOGPOGARGPOGPOGPOGPO^[65]^ | 36.9 | 36.8 | 38.3 |
| 193 | 1 | 24 | ac | am | 37.7 | GPOGPOGPOGEQGPOGPOGPOGPO^[16]^ | 38.2 | 38.8 | 36.8 |
| 194 | 1 | 24 | ac | am | 37.1 | GPOGPOGPOGDRGPOGPOGPOGPO^[62]^ | 35.1 | 36.3 | 39.2 |
| 195 | 1 | 24 | ac | am | 35.9 | GPOGPOGPOGETGPOGPOGPOGPO^[16]^ | 35.1 | 36.1 | 36.3 |
| 196 | 1 | 24 | ac | am | 35.7 | GPOGPOGPOGLQGPOGPOGPOGPO^[16]^ | 36.4 | 36.5 | 36.4 |
| 197 | 1 | 24 | ac | am | 35.8 | GPOGPOGPOGKDGPOGPOGPOGPO^[62]^ | 35.4 | 35.3 | 36.1 |
| 198 | 1 | 24 | ac | am | 35.3 | GPOGPOGPOGEVGPOGPOGPOGPO^[16]^ | 35.2 | 35.4 | 36.9 |
| 199 | 1 | 24 | ac | am | 35.3 | GPOGPOGPOGKEGPOGPOGPOGPO^[62]^ | 36.1 | 38.7 | 36.8 |
| 200 | 1 | 24 | ac | am | 35 | GPOGPOGPOGEKGPOGPOGPOGPO^[62]^ | 34.1 | 35.2 | 36.6 |
| 201 | 1 | 24 | ac | am | 34.6 | GPOGPOGPOGEAGPOGPOGPOGPO^[16]^ | 33.8 | 34.9 | 35.1 |
| 202 | 1 | 24 | ac | am | 34.5 | GPOGPOGPOGRDGPOGPOGPOGPO^[16]^ | 33.4 | 34.4 | 32.9 |
| 203 | 1 | 24 | ac | am | 33.9 | GPOGPOGPOGIAGPOGPOGPOGPO^[16]^ | 34.2 | 32.9 | 34.0 |
| 204 | 1 | 24 | ac | am | 33.8 | GPOGPOGPOGREGPOGPOGPOGPO^[62]^ | 34.7 | 34.6 | 33.9 |
| 205 | 1 | 24 | ac | am | 33 | GPOGPOGPOGADGPOGPOGPOGPO^[16]^ | 33.6 | 33.9 | 29.6 |
| 206 | 1 | 24 | ac | am | 33 | GPOGPOGPOGASGPOGPOGPOGPO^[16]^ | 32.8 | 33.0 | 35.3 |
| 207 | 1 | 24 | ac | am | 32.9 | GPOGPOGPOGAAGPOGPOGPOGPO^[16]^ | 34.1 | 34.1 | 33.4 |
| 208 | 1 | 24 | ac | am | 32.6 | GPOGPOGPOGQKGPOGPOGPOGPO^[16]^ | 33.1 | 32.7 | 29.8 |
| 209 | 1 | 24 | ac | am | 32.5 | GPOGPOGPOGVKGPOGPOGPOGPO^[16]^ | 33.3 | 33.0 | 29.2 |
| 210 | 1 | 24 | ac | am | 31.7 | GPOGPOGPOGMKGPOGPOGPOGPO^[16]^ | 32.1 | 32.3 | 32.1 |
| 211 | 1 | 24 | ac | am | 31.7 | GPOGPOGPOGKNGPOGPOGPOGPO^[16]^ | 35.5 | 36.1 | 29.1 |
| 212 | 1 | 24 | ac | am | 31.6 | GPOGPOGPOGDAGPOGPOGPOGPO^[16]^ | 30.9 | 32.2 | 34.0 |
| 213 | 1 | 24 | ac | am | 31.2 | GPOGPOGPOGLAGPOGPOGPOGPO^[16]^ | 33.7 | 33.1 | 32.7 |
| 214 | 1 | 24 | ac | am | 31.1 | GPOGPOGPOGLKGPOGPOGPOGPO^[16]^ | 29.7 | 31.0 | 32.6 |
| 215 | 1 | 24 | ac | am | 30.9 | GPOGPOGPOGDKGPOGPOGPOGPO^[62]^ | 32.8 | 33.3 | 33.9 |
| 216 | 1 | 24 | ac | am | 30.8 | GPOGPOGPOGAKGPOGPOGPOGPO^[65]^ | 30.8 | 31.7 | 34.3 |
| 217 | 1 | 24 | ac | am | 30.5 | GPOGPOGPOGRSGPOGPOGPOGPO^[16]^ | 30.4 | 31.9 | 33.2 |
| 218 | 1 | 24 | ac | am | 29.7 | GPOGPOGPOGEDGPOGPOGPOGPO^[16]^ | 29.0 | 28.5 | 28.6 |
| 219 | 1 | 24 | ac | am | 29.5 | GPOGPOGPOGRKGPOGPOGPOGPO^[16]^ | 29.3 | 30.8 | 31.3 |
| 220 | 1 | 24 | ac | am | 29.5 | GPOGPOGPOGENGPOGPOGPOGPO^[16]^ | 27.2 | 30.4 | 28.9 |
| 221 | 1 | 24 | ac | am | 27.8 | GPOGPOGPOGALGPOGPOGPOGPO^[63]^ | 27.9 | 28.9 | 28.2 |
| 222 | 1 | 24 | ac | am | 26.9 | GPOGPOGPOGGKGPOGPOGPOGPO^[16]^ | 26.0 | 26.5 | 27.8 |
| 223 | 1 | 24 | ac | am | 26.9 | GPOGPOGPOGLLGPOGPOGPOGPO^[16]^ | 29.4 | 27.2 | 29.0 |
| 224 | 1 | 24 | ac | am | 26 | GPOGPOGPOGGAGPOGPOGPOGPO^[16]^ | 25.4 | 26.9 | 28.4 |
| 225 | 1 | 24 | ac | am | 25.3 | GPOGPOGPOGGLGPOGPOGPOGPO^[16]^ | 26.1 | 23.5 | 22.1 |
| 226 | 1 | 24 | ac | am | 24.1 | GPOGPOGPOGFAGPOGPOGPOGPO^[16]^ | 24.9 | 24.2 | 20.8 |
| 227 | 1 | 24 | ac | am | 21.9 | GPOGPOGPOGAFGPOGPOGPOGPO^[16]^ | 20.4 | 24.0 | 22.5 |
| 228 | 1 | 24 | ac | am | 19.7 | GPOGPOGPOGGFGPOGPOGPOGPO^[50]^ | 19.6 | 19.4 | 16.5 |
| 232 | 1 | 24 | ac | am | 33.3 | GPOGPOGPOGPOGAAGPOGPOGPO^[15]^ | 34.1 | 34.1 | 33.4 |
| 233 | 1 | 24 | ac | am | 20 | GPOGPOGPOGAAGAAGPOGPOGPO^[15]^ | 20.2 | 20.3 | 20.1 |
| 234 | 1 | 24 | ac | am | 36.9 | GPOGPOGPOGAOGAOGPOGPOGPO^[15]^ | 37.0 | 35.3 | 34.5 |
| 235 | 1 | 24 | ac | am | 35.8 | GPOGPOGPOGPAGPAGPOGPOGPO^[15]^ | 34.9 | 33.7 | 32.1 |
| 236 | 1 | 24 | ac | am | 28.2 | GPOGPOGPOGPLGLOGPOGPOGPO^[15]^ | 26.6 | 27.3 | 29.3 |
| 237 | 1 | 24 | ac | am | 38.1 | GPOGPOGPOGLOGLOGPOGPOGPO^[15]^ | 35.6 | 36.0 | 36.7 |
| 239 | 1 | 24 | ac | am | 38 | GPOGPOGPOGEOGPKGPOGPOGPO^[15]^ | 33.3 | 33.9 | 34.6 |
| 240 | 1 | 24 | ac | am | 37.9 | GPOGPOGPOGKOGEOGPOGPOGPO^[15]^ | 38.1 | 38.4 | 38.7 |
| 241 | 1 | 24 | ac | am | 36.5 | GPOGPOGPOGPKGPEGPOGPOGPO^[15]^ | 32.0 | 32.6 | 32.5 |
| 243 | 1 | 24 | ac | am | 39.6 | GPOGPOGPOGPRGDOGPOGPOGPO^[15]^ | 39.2 | 40.1 | 41.1 |
| 244 | 1 | 24 | ac | am | 36.2 | GPOGPOGPOGDOGPRGPOGPOGPO^[15]^ | 36.5 | 36.5 | 39.7 |
| 245 | 1 | 24 | ac | am | 42.8 | GPOGPOGPOGPRGEOGPOGPOGPO^[15]^ | 40.4 | 39.8 | 41.9 |
| 246 | 1 | 24 | ac | am | 45.5 | GPOGPOGPOGPPGPOGPOGPOGPO^[14]^ | 44.3 | 44.7 | 44.1 |
| 247 | 1 | 24 | ac | am | 37.7 | GPOGPOGPOGAPGPOGPOGPOGPO^[14]^ | 38.4 | 37.3 | 41.6 |
| 248 | 1 | 32 | ac | am | 42.8 | GPOGPOGPOGAKGDAGPOGPAGPOGPOGPOGY^[14]^ | 40.8 | 39.3 | 41.4 |
| 249 | 1 | 32 | ac | am | 27.5 | GPAGPAGPVGPAGARGPAGPOGPOGPOGPOGV^[66]^ | 28.3 | 27.5 | 24.6 |
| 250 | 1 | 32 | ac | am | 22.9 | GPAGPAGPVGPAGARGPAGPOGPOGPOGPOGY^[67]^ | 28.3 | 27.5 | 27.1 |
| 252 | 1 | 32 | ac | am | 26 | GPOGPOGPOGPOGPAGPAGPVGPAGARGPAGV^[68]^ | 23.8 | 24.6 | 24.3 |
| 257 | 1 | 32 | n | c | 20.6 | GPAGPAGPVGPAGARGPAGPOGPOGPOGPOGY^[14]^ | 21.1 | 21.5 | 25.1 |
| 258 | 1 | 32 | ac | am | 30.8 | GARGPAGPQGPRGDKGETGPOGPOGPOGPOGV^[66]^ | 32.3 | 30.7 | 31.0 |
| 259 | 1 | 32 | ac | c | 25 | GSOGAQGLQGPRGLOGTOGPOGPOGPOGPOGV^[14]^ | 29.2 | 25.8 | 26.9 |
| 260 | 1 | 32 | ac | c | 20.9 | GGKGDAGAOGERGPOGLAGPOGPOGPOGPOGV^[50]^ | 24.6 | 21.8 | 23.4 |
| 261 | 1 | 32 | ac | c | 23.2 | GDAGAOGERGPOGLAGAOGPOGPOGPOGPOGV^[50]^ | 29.4 | 24.1 | 23.8 |
| 262 | 1 | 32 | ac | c | 25.9 | GAOGERGPOGLAGAOGLRGPOGPOGPOGPOGV^[50]^ | 30.4 | 26.5 | 25.5 |
| 263 | 1 | 32 | ac | c | 16.5 | GERGPOGLAGAOGLRGGAGPOGPOGPOGPOGV^[50]^ | 19.6 | 14.1 | 14.1 |
| 264 | 1 | 32 | ac | c | 15.8 | GPOGLAGAOGLRGGAGPOGPOGPOGPOGPOGV^[50]^ | 19.3 | 15.2 | 16.1 |
| 265 | 1 | 32 | ac | c | 17.5 | GLAGAOGLRGGAGPOGPEGPOGPOGPOGPOGV^[50]^ | 25.9 | 19.5 | 18.5 |
| 266 | 1 | 30 | ac | am | 8.9 | GAOGLRGGAGPOGPEGGKGPOGPOGPOGPO^[50]^ | 15.6 | 9.4 | 8.8 |
| 267 | 1 | 32 | ac | am | 25.4 | GLAGEOGKOGIOGLOGRAGPOGPOGPOGPOGV^[14]^ | 27.6 | 26.1 | 23.8 |
| 268 | 1 | 32 | ac | am | 23.2 | GERGERGEKGERGEQGRDGPOGPOGPOGPOGV^[14]^ | 23.3 | 25.8 | 26.6 |
| 269 | 1 | 26 | ac | am | 32.3 | GPOGPOGPOGKRGKOGPOGPOGPOGG^[69]^ | 32.8 | 32.6 | 34.1 |
| 272 | 1 | 26 | ac | am | 20.1 | GPOGPOGRQGKRGKOGPOGPOGPOGG^[69]^ | 19.6 | 19.2 | 21.4 |
| 276 | 1 | 30 | n | am | 18.6 | POGROGAAGAOGVRGPRGPOGPOGPOGPOG^[70]^ | 15.4 | 18.5 | 22.7 |
| 279 | 1 | 30 | n | am | 7 | POGLOGMLGQKGEMGPKGPOGPOGPOGPOG^[70]^ | 6.6 | 7.7 | 11.0 |
| 280 | 1 | 30 | n | am | 19.7 | POGROGKRGKOGVRGPRGPOGPOGPOGPOG^[88]^ | 17.2 | 21.8 | 25.4 |
| 281 | 1 | 31 | ac | am | 30 | GPOGPOGROGRKGROGPOGPOGPOGPOGPOG^[70]^ | 31.6 | 32.0 | 35.7 |
| 282 | 1 | 31 | ac | am | 23.9 | GPOGPOGROGKRGKQGQKGPOGPOGPOGPOG^[70]^ | 24.3 | 24.8 | 23.6 |
| 283 | 1 | 30 | n | c | 30 | POGPOGPOGPKGQKGEKGPOGPOGPOGPOG^[71]^ | 30.7 | 28.7 | 28.7 |
| 284 | 1 | 35 | ac | am | 23 | GINGFOGKDGRDGTKGEKGEOGPOGPOGPOGPOGG^[72]^ | 22.3 | 21.2 | 16.5 |
| 285 | 1 | 32 | n | c | 17.9 | GFOGKDGRDGTKGEKGEOGPOGPOGPOGPOGY^[72]^ | 15.9 | 18.4 | 20.5 |
| 286 | 1 | 21 | ac | c | 18.5 | GTKGEKGEOGPOGPOGPOGPO^[72]^ | 24.7 | 16.3 | 26.2 |
| 287 | 1 | 32 | ac | am | 23.3 | GPOGROGRRGROGLKGEQGPOGPOGPOGPOGY^[14]^ | 25.0 | 23.5 | 21.6 |
| 288 | 1 | 32 | ac | am | 28.4 | GPOGIRGPKGQKGEOGLOGPOGPOGPOGPOGY^[14]^ | 28.5 | 29.1 | 28.4 |
| 289 | 1 | 32 | n | c | 44 | GPOGPOGPOGQOGLOGLOGPOGPOGPOGPOGY^[54]^ | 39.9 | 42.1 | 47.4 |
| 293 | 1 | 24 | ac | am | 37 | GPOGPOGPOGPEGKOGPOGPOGPO^[73]^ | 39.1 | 35.5 | 39.1 |
| 294 | 1 | 24 | ac | am | 33 | GPOGPOGPOGPDGKOGPOGPOGPO^[73]^ | 32.6 | 32.1 | 32.3 |
| 299 | 1 | 30 | ac | am | 32 | POGPOGPOGPOGPOGEOGEOGEOGEOGEOG^[29]^ | 39.6 | 39.8 | 42.7 |
| 306 | 3 | 30 | ac | am | 50 | PRGPRGPRGPRGPRGPOGPOGPOGPOGPOG^[29]^ POGPOGPOGPOGPOGEOGEOGEOGEOGEOG EOGEOGEOGEOGEOGPRGPRGPRGPRGPRG | 51.0 | 50.9 | 51.1 |
| 308 | 3 | 30 | n | c | 30.8 | PKGPKGPKGKOGPDGDOGDOGDOGPKGPKG^[27]^ PDGDOGDOGDOGPDGKOGPDGPDGPDGDOG KOGPDGPDGPKGAOGPKGKOGKOGKOGKOG | 30.2 | 31.7 | 28.6 |
| 309 | 3 | 30 | n | c | 31.3 | PKGPKGPKGKOGPDGDOGDOGDOGPKGPKG^[27]^ PDGDOGDOGDOGPDGKOGPDGPDGPDGDOG KOGPDGPAGPKGKOGPKGKOGKOGKOGKOG | 27.4 | 31.8 | 30.3 |
| 310 | 3 | 30 | n | c | 34.4 | PKGPKGPKGKOGPDGDOGAOGDOGPKGPKG^[27]^ PDGDOGDOGDOGPDGKOGPDGPDGPDGDOG KOGPDGPDGPKGKOGPKGKOGKOGKOGKOG | 28.8 | 32.5 | 31.9 |
| 311 | 3 | 30 | n | c | 33.5 | PKGPKGPKGKOGPDGDOGDOGDOGPKGPKG^[27]^ PDGDOGDOGDOGPDGAOGPDGPDGPDGDOG KOGPDGPDGPKGKOGPKGKOGKOGKOGKOG | 29.6 | 33.7 | 32.1 |
| 312 | 3 | 30 | n | c | 35 | PKGPKGPKGKOGPDGDOGDOGDOGPKGPKG^[27]^ PDGDOGDOGDOGPDGKOGPDGPDGPDGDOG KOGPAGPDGPKGKOGPKGKOGKOGKOGKOG | 30.1 | 34.3 | 34.2 |
| 313 | 3 | 30 | n | c | 31.2 | PKGPKGPKGKOGPDGDOGDOGAOGPKGPKG^[27]^ PDGDOGDOGDOGPDGKOGPDGPDGPDGDOG KOGPDGPDGPKGKOGPKGKOGKOGKOGKOG | 25.3 | 30.2 | 26.8 |
| 314 | 3 | 30 | n | c | 28.6 | PKGPKGPKGKOGPDGAOGDOGDOGPKGPKG^[27]^ PDGDOGDOGDOGPDGKOGPDGPDGPDGDOG KOGPDGPDGPKGKOGPKGKOGKOGKOGKOG | 25.3 | 30.2 | 26.8 |
| 315 | 3 | 30 | n | c | 28.2 | PKGPKGPKGKOGPDGDOGDOGDOGPKGPKG^[27]^ PDGDOGDOGDOGPDGKOGPDGPDGPDGDOG KOGPDGPDGPKGDOGPKGKOGKOGKOGKOG | 24.9 | 30.0 | 27.1 |
| 316 | 3 | 30 | n | c | 30 | PKGPKGPKGKOGPDGDOGDOGDOGPKGPKG^[27]^ PDGDOGDOGDOGPDGKOGPDGPDGPDGDOG KOGPDGPKGPKGKOGPKGKOGKOGKOGKOG | 25.1 | 29.1 | 30.2 |
| 317 | 3 | 30 | n | c | 33.4 | PKGPKGPKGKOGPDGDOGKOGDOGPKGPKG^[27]^ PDGDOGDOGDOGPDGKOGPDGPDGPDGDOG KOGPDGPDGPKGKOGPKGKOGKOGKOGKOG | 29.1 | 33.1 | 32.1 |
| 318 | 3 | 30 | n | c | 29 | PKGPKGPKGKOGPDGDOGDOGDOGPKGPKG^[27]^ PDGDOGDOGDOGPDGDOGPDGPDGPDGDOG KOGPDGPDGPKGKOGPKGKOGKOGKOGKOG | 26.1 | 30.6 | 30.2 |
| 319 | 3 | 30 | n | c | 34.4 | PKGPKGPKGKOGPDGDOGDOGDOGPKGPKG^[27]^ PDGDOGDOGDOGPDGKOGPDGPDGPDGDOG KOGPKGPDGPKGKOGPKGKOGKOGKOGKOG | 29.2 | 33.8 | 32.1 |
| 321 | 2 | 30 | ac | am | 64 | POGPOGPOGPOGPOGPOGPOGPOGPOGPOG^[49]^ EOGPOGEOGPOGEOGPOGEOGPOGEOGPOG | 66.9 | 60.1 | 58.6 |
| 322 | 2 | 30 | ac | am | 65 | POGPOGPOGPOGPOGPOGPOGPOGPOGPOG^[49]^ PRGPOGPRGPOGPRGPOGPRGPOGPRGPOG | 66.9 | 62.6 | 61.8 |
| 323 | 3 | 30 | ac | am | 54 | PRGPRGPRGPRGPRGPRGPRGPRGPRGPRG^[49]^ EOGEOGEOGEOGEOGEOGEOGEOGEOGEOG POGPOGPOGPOGPOGPOGPOGPOGPOGPOG | 66.9 | 50.9 | 51.1 |
| 324 | 3 | 30 | ac | am | 65 | PKGPKGPKGPKGPKGPKGPKGPKGPKGPKG^[56]^ DOGDOGDOGDOGDOGDOGDOGDOGDOGDOG POGPOGPOGPOGPOGPOGPOGPOGPOGPOG | 60.3 | 63.5 | 59.8 |
| 325 | 3 | 30 | ac | am | 57.5 | PKGPKGPKGPKGPKGPKGPKGPKGPKGPKG^[56]^ EOGEOGEOGEOGEOGEOGEOGEOGEOGEOG POGPOGPOGPOGPOGPOGPOGPOGPOGPOG | 60.3 | 55.9 | 53.9 |
| 326 | 3 | 30 | ac | am | 44.5 | PRGPRGPRGPRGPRGPRGPRGPRGPRGPRG^[56]^ DOGDOGDOGDOGDOGDOGDOGDOGDOGDOG POGPOGPOGPOGPOGPOGPOGPOGPOGPOG | 66.9 | 49.1 | 45.2 |
| 327 | 1 | 39 | ac | am | 68 | POGPOGPOGPOGPOGPQGPGGPPGPOGPOGPOGPOGPOG^[74]^ | 61.1 | 61.3 | 65.1 |
| 328 | 1 | 39 | ac | am | 54 | POGPOGPOGPOGPOGPVGAAGATGPOGPOGPOGPOGPOG^[74]^ | 53.0 | 53.3 | 52.5 |
| 329 | 3 | 39 | ac | am | 60 | POGPOGPOGPOGPOGPQGPGGPPGPOGPOGPOGPOGPOG^[74]^ PKGPKGPKGPKGPKGPQGPGGPPGPKGPKGPKGPKGPKG DOGDOGDOGDOGDOGPVGAAGATGDOGDOGDOGDOGDOG | 61.1 | 58.3 | 56.2 |
| 331 | 3 | 30 | n | am | 60 | PKGPKGPKGDKGPKGPKGPKGPKGDKGPKG^[75]^ DOGDOGDOGDOGDOGDOGDOGDOGDOGDOG POGPKGPOGPOGPOGPOGPKGPOGPOGPOG | 59.2 | 57.1 | 58.6 |
| 332 | 1 | 30 | n | am | 51 | POGPKGPOGPOGPOGPOGPKGPOGPOGPOG^[75]^ | 45.0 | 43.9 | 48.4 |
| 333 | 3 | 30 | n | am | 52 | PKGPKGPKGDKGPKGPKGPKGPKGDKGPKG^[75]^ EOGEOGEOGEOGEOGEOGEOGEOGEOGEOG POGPKGPOGPOGPOGPOGPKGPOGPOGPOG | 52.0 | 49.5 | 52.6 |
| 334 | 3 | 30 | ac | am | 58 | PKGPKGDOGPOGDKGDKGPKGPOGDKGPOG^[26]^ POGDOGDKGPOGPOGDKGDOGDKGPKGDOG PKGPOGPKGDKGPOGPOGDKGPOGDOGDOG | 64.0 | 63.4 | 61.0 |
| 335 | 1 | 30 | ac | am | 32 | PKGPOGPKGDKGPOGPOGDKGPOGDOGDOG^[26]^ | 33.8 | 33.0 | 29.0 |
| 336 | 2 | 30 | ac | am | 43 | POGDOGDKGPOGPOGDKGDOGDKGPKGDOG^[26]^ PKGPOGPKGDKGPOGPOGDKGPOGDOGDOG | 43.8 | 42.3 | 38.8 |
| 337 | 2 | 30 | ac | am | 43 | PKGPKGDOGPOGDKGDKGPKGPOGDKGPOG^[26]^ PKGPOGPKGDKGPOGPOGDKGPOGDOGDOG | 46.6 | 46.4 | 39.2 |
| 338 | 2 | 30 | ac | am | 33 | PKGPKGDOGPOGDKGDKGPKGPOGDKGPOG^[26]^ POGDOGDKGPOGPOGDKGDOGDKGPKGDOG | 39.0 | 37.7 | 34.9 |
| 339 | 1 | 30 | ac | am | 30.5 | KOGKOGKOGKOGKOGKOGKOGKOGKOGKOG^[58]^ | 20.8 | 20.2 | 24.3 |
| 340 | 3 | 30 | ac | am | 43.5 | KOGKOGKOGKOGKOGKOGKOGKOGKOGKOG^[58]^ PEGPEGPEGPEGPEGPEGPEGPEGPEGPEG POGPOGPOGPOGPOGPOGPOGPOGPOGPOG | 66.9 | 45.0 | 45.4 |
| 341 | 3 | 30 | ac | am | 53 | KOGKOGKOGKOGKOGKOGKOGKOGKOGKOG^[58]^ PDGPDGPDGPDGPDGPDGPDGPDGPDGPDG POGPOGPOGPOGPOGPOGPOGPOGPOGPOG | 66.9 | 41.2 | 45.5 |
| 344 | 3 | 30 | n | am | 43 | PKGPKGPKGPKGPKGPKGPKGPKGPKGPKG^[76]^ DKGDKGDKGDKGDKGDKGDKGDKGDKGDKG EOGEOGEOGEOGEOGEOGEOGEOGEOGEOG | 47.7 | 45.3 | 48.6 |
| 345 | 3 | 30 | n | am | 37 | PKGPKGPKGPKGPKGPKGPKGPKGPKGPKG^[76]^ DKGDKGDKGDKGDKGDKGDKGDKGDKGDKG EPGEPGEPGEPGEPGEPGEPGEPGEPGEPG | 43.0 | 36.4 | 39.5 |
| 346 | 2 | 30 | n | am | 39 | PKGPKGPKGPKGPKGPKGPKGPKGPKGPKG^[76]^ EOGEOGEOGEOGEOGEOGEOGEOGEOGEOG | 37.1 | 34.1 | 39.8 |
| 347 | 2 | 30 | n | am | 52 | PKGDOGPKGDOGPKGDOGPKGDOGPKGDOG^[77]^ POGDOGPOGDOGPOGDOGPOGDOGPOGDOG | 50.7 | 48.1 | 53.2 |
| 348 | 1 | 30 | n | am | 43 | PKGDOGPKGDOGPKGDOGPKGDOGPKGDOG^[77]^ | 43.5 | 41.0 | 40.9 |
| 349 | 2 | 30 | n | am | 51 | PKGPOGPKGPOGPKGPOGPKGPOGPKGPOG^[77]^ PKGDOGPKGDOGPKGDOGPKGDOGPKGDOG | 50.3 | 47.8 | 50.2 |
| 350 | 2 | 30 | n | am | 51 | PKGEOGPKGEOGPKGEOGPKGEOGPKGEOG^[77]^ POGDOGPOGDOGPOGDOGPOGDOGPOGDOG | 50.9 | 48.2 | 48.3 |
| 351 | 2 | 30 | n | am | 40 | PKGPOGPKGPOGPKGPOGPKGPOGPKGPOG^[77]^ PKGEOGPKGEOGPKGEOGPKGEOGPKGEOG | 43.0 | 39.8 | 36.6 |
| 352 | 1 | 30 | n | am | 32 | PKGEOGPKGEOGPKGEOGPKGEOGPKGEOG^[77]^ | 37.6 | 35.3 | 34.1 |
| 353 | 1 | 30 | n | am | 45 | EOGPOGEOGPOGEOGPOGEOGPOGEOGPOG^[78]^ | 42.0 | 38.9 | 45.3 |
| 354 | 1 | 30 | n | am | 42 | POGEOGPOGEOGPOGEOGPOGEOGPOGEOG^[78]^ | 38.4 | 37.2 | 42.3 |
| 355 | 2 | 30 | n | am | 36 | PKGEOGPKGEOGPKGEOGPKGEOGPKGEOG^[78]^ POGPKGPOGPKGPOGPKGPOGPKGPOGPKG | 39.2 | 37.0 | 34.4 |
| 356 | 2 | 30 | n | am | 44 | PKGEOGPKGEOGPKGEOGPKGEOGPKGEOG^[78]^ EOGPOGEOGPOGEOGPOGEOGPOGEOGPOG | 45.4 | 42.2 | 40.7 |
| 357 | 2 | 30 | n | am | 42 | POGPKGPOGPKGPOGPKGPOGPKGPOGPKG^[78]^ EOGPOGEOGPOGEOGPOGEOGPOGEOGPOG | 50.1 | 48.0 | 48.2 |
| 358 | 3 | 30 | n | am | 47 | POGPKGPOGPKGPOGPKGPOGPKGPOGPKG^[78]^ EOGPOGEOGPOGEOGPOGEOGPOGEOGPOG PKGEOGPKGEOGPKGEOGPKGEOGPKGEOG | 50.1 | 45.8 | 41.1 |
| 359 | 2 | 30 | n | am | 39 | PKGPOGPKGPOGPKGPOGPKGPOGPKGPOG^[78]^ PKGEOGPKGEOGPKGEOGPKGEOGPKGEOG | 43.0 | 39.8 | 36.6 |
| 360 | 2 | 30 | n | am | 41 | POGEOGPOGEOGPOGEOGPOGEOGPOGEOG^[78]^ PKGEOGPKGEOGPKGEOGPKGEOGPKGEOG | 47.2 | 44.2 | 41.9 |
| 361 | 2 | 30 | n | am | 49 | PKGPOGPKGPOGPKGPOGPKGPOGPKGPOG^[78]^ POGEOGPOGEOGPOGEOGPOGEOGPOGEOG | 51.3 | 49.0 | 49.9 |
| 362 | 3 | 30 | n | am | 52 | PKGPOGPKGPOGPKGPOGPKGPOGPKGPOG^[78]^ PKGEOGPKGEOGPKGEOGPKGEOGPKGEOG POGEOGPOGEOGPOGEOGPOGEOGPOGEOG | 54.9 | 51.7 | 49.4 |
| 363 | 1 | 30 | ac | am | 39 | POGPKGDKGDOGFOGERGPOGPKGDKGDOG^[19]^ | 35.5 | 36.2 | 35.3 |
| 364 | 1 | 30 | ac | am | 28 | PKGPKGEOGEOGPOGESGPKGPKGEOGEOG^[19]^ | 26.8 | 26.2 | 28.6 |
| 365 | 2 | 30 | ac | am | 46 | POGPKGDKGDOGFOGERGPOGPKGDKGDOG^[19]^ PKGPKGEOGEOGPOGESGPKGPKGEOGEOG | 41.4 | 41.5 | 42.0 |
| 366 | 1 | 36 | ac | am | 47 | POGPKGDOGPKGDOGFOGERGPOGPKGDOGPKGDOG^[19]^ | 41.6 | 43.2 | 39.8 |
| 367 | 1 | 36 | n | am | 52 | PKGPOGDKGPOGDOGPOGESGPKGPOGDKGPOGDOG^[79]^ | 48.0 | 51.6 | 34.8 |
| 368 | 2 | 36 | ac | am | 59 | POGPKGDOGPKGDOGFOGERGPOGPKGDOGPKGDOG^[79]^ PKGPOGDKGPOGDOGPOGESGPKGPOGDKGPOGDOG | 57.4 | 58.3 | 58.6 |
| 369 | 1 | 30 | ac | am | 37 | POGPKGDOGPKGDOGPKGDOGFOGERGPOG^[79]^ | 34.9 | 35.5 | 33.5 |
| 370 | 1 | 30 | n | am | 44 | PKGPOGDKGPOGDKGPOGDOGPOGESGPOG^[79]^ | 41.0 | 44.7 | 31.0 |
| 371 | 2 | 30 | ac | am | 47 | POGPKGDOGPKGDOGPKGDOGFOGERGPOG^[79]^ PKGPOGDKGPOGDKGPOGDOGPOGESGPOG | 47.1 | 47.6 | 46.9 |
| 372 | 2 | 30 | ac | am | 39.5 | POGPKGDPGPKGDPGPKGDPGFOGERGPOG^[79]^ PKGPOGDKGPOGDKGPOGDOGPOGESGPOG | 44.9 | 39.4 | 39.3 |
| 373 | 1 | 30 | n | am | 32 | PKGPPGDKGPPGDKGPPGDOGPOGESGPOG^[79]^ | 31.7 | 35.6 | 34.4 |
| 374 | 2 | 30 | ac | am | 34 | POGPKGDPGPKGDPGPKGDPGFOGERGPOG^[79]^ PKGPPGDKGPPGDKGPPGDOGPOGESGPOG | 35.7 | 34.2 | 35.9 |
| 375 | 1 | 30 | n | am | 26 | PPGDKGPPGPPGDKGDKGPOGPOGESGPOG^[79]^ | 22.0 | 24.9 | 24.5 |
| 376 | 2 | 30 | ac | am | 36 | PKGPPGDPGPKGPKGDPGDOGFOGERGPOG^[79]^ PPGDKGPPGPPGDKGDKGPOGPOGESGPOG | 33.9 | 33.0 | 38.2 |
| 377 | 2 | 30 | ac | am | 33 | PKGDPGPKGDPGPKGDPGPOGFOGERGPOG^[79]^ PKGPPGDKGPPGDKGPPGDOGPOGESGPOG | 35.6 | 35.0 | 38.1 |
| 378 | 1 | 42 | ac | am | 40 | PPGPKGDPGPKGDPGPKGDPGPPGPOGDQGPOGIOGPPGPPG^[79]^ | 39.6 | 38.7 | 43.7 |
| 380 | 2 | 42 | ac | am | 45.5 | PPGPKGDPGPKGDPGPKGDPGPPGPOGDQGPOGIOGPPGPPG^[79]^ PKGPPGDKGPPGDKGPPGDOGPPGAKGRAGFOGLOGPPGPPG | 46.0 | 45.1 | 44.2 |
| 381 | 1 | 45 | n | am | 29.5 | PPGPKGDPGPKGDPGPKGDPGPOGPQGIAGQRGVVGLOGPPGPPG^[79]^ | 26.8 | 28.5 | 25.6 |
| 382 | 1 | 45 | n | am | 27.5 | PKGPPGDKGPPGDKGPPGDPGPOGPQGLLGAPGILGLOGPPGPPG^[79]^ | 25.1 | 26.9 | 26.6 |
| 383 | 2 | 45 | n | am | 33 | PPGPKGDPGPKGDPGPKGDPGPOGPQGIAGQRGVVGLOGPPGPPG^[79]^ PKGPPGDKGPPGDKGPPGDPGPOGPQGLLGAPGILGLOGPPGPPG | 31.7 | 34.5 | 28.5 |
| 384 | 1 | 45 | n | am | 27.5 | PKGDPGPKGDPGPKGDPGPPGPOGPQGIAGQRGVVGLOGPPGPPG^[79]^ | 26.8 | 28.4 | 28.3 |
| 385 | 2 | 45 | n | am | 31.5 | PKGDPGPKGDPGPKGDPGPPGPOGPQGIAGQRGVVGLOGPPGPPG^[79]^ PKGPPGDKGPPGDKGPPGDPGPOGPQGLLGAPGILGLOGPPGPPG | 31.1 | 30.6 | 30.4 |
| 386 | 1 | 45 | n | am | 26 | PKGPPGDPGPKGPKGDPGDPGPOGPQGIAGQRGVVGLOGPPGPPG^[79]^ | 24.4 | 26.7 | 29.5 |
| 387 | 1 | 45 | n | am | 16 | PPGDKGPPGPPGDKGDKGPPGPOGPQGLLGAPGILGLOGPPGPPG^[79]^ | 15.0 | 16.7 | 17.0 |
| 388 | 2 | 45 | n | am | 33 | PKGPPGDPGPKGPKGDPGDPGPOGPQGIAGQRGVVGLOGPPGPPG^[79]^ PPGDKGPPGPPGDKGDKGPPGPOGPQGLLGAPGILGLOGPPGPPG | 30.8 | 32.8 | 29.1 |
| 389 | 1 | 39 | n | am | 32 | PKGPPGDKGPPGDKGPPGDPGARGEOGNIGFOGPOGPOG^[79]^ | 29.8 | 32.6 | 33.3 |
| 390 | 2 | 39 | ac | am | 35 | POGPKGDPGPKGDPGPKGDPGARGQAGVMGFOGPOGPOG^[79]^ PKGPPGDKGPPGDKGPPGDPGARGEOGNIGFOGPOGPOG | 35.9 | 35.1 | 33.5 |
| 391 | 1 | 39 | n | am | 21 | PKGPPGDPGPKGPKGDPGDOGARGQAGVMGFOGPOGPOG^[79]^ | 18.7 | 20.0 | 23.9 |
| 392 | 1 | 39 | n | am | 21.5 | PPGDKGPPGPPGDKGDKGPPGARGEOGNIGFOGPOGPOG^[79]^ | 20.0 | 20.5 | 21.7 |
| 393 | 2 | 39 | ac | am | 35 | PKGPPGDPGPKGPKGDPGDOGARGQAGVMGFOGPOGPOG^[79]^ PPGDKGPPGPPGDKGDKGPPGARGEOGNIGFOGPOGPOG | 34.7 | 36.2 | 32.7 |
| 398 | 1 | 30 | ac | am | 30.2 | GPPGPKGPPGDKGPPGDKGPPGDPGPPGGY^[33]^ | 25.7 | 26.3 | 25.4 |
| 399 | 1 | 30 | n | c | 41 | PIGPOGPOGPOGPOGLOGLOGPIGPOGPOG^[80]^ | 41.2 | 41.0 | 38.9 |
| 400 | 1 | 30 | n | c | 37 | LOGPOGLOGPOGLOGPOGLOGPOGLOGPOG^[80]^ | 36.4 | 36.7 | 38.0 |
| 401 | 1 | 30 | n | c | 28 | POGPIGPOGPIGPOGPIGPOGPIGPOGPIG^[80]^ | 27.3 | 28.8 | 27.1 |
| 402 | 3 | 30 | n | c | 25.1 | PKGPKGPKGKOGPDGDOGDOGDOGPKGPKG^[81]^ PDGDOGDFGDOGPDGKOGPDGPDGPDGDOG KOGPDGPDGPKGKOGPKGKOGKOGKOGKOG | 21.7 | 25.9 | 25.4 |
| 403 | 3 | 30 | n | c | 27.3 | PKGPKGPKGKOGPDGDOGDOGDOGPKGPKG^[81]^ PDGDOGDYGDOGPDGKOGPDGPDGPDGDOG KOGPDGPDGPKGKOGPKGKOGKOGKOGKOG | 22.6 | 26.6 | 27.4 |
| 404 | 3 | 30 | n | c | 26.6 | PKGPKGPKGKOGPDGDOGDOGDOGPKGPKG^[81]^ PDGDOGDWGDOGPDGKOGPDGPDGPDGDOG KOGPDGPDGPKGKOGPKGKOGKOGKOGKOG | 21.1 | 25.3 | 24.7 |
| 405 | 3 | 30 | n | c | 29 | PKGPKGPKGKOGPDGDOGDOGDOGPKGPKG^[81]^ PDGDOGDRGDOGPDGKOGPDGPDGPDGDOG KOGPDGPDGPKGKOGPKGKOGKOGKOGKOG | 26.8 | 30.8 | 30.7 |
| 406 | 3 | 30 | n | c | 27.6 | PKGPKGPKGKOGPDGDOGDOGDOGPKGPKG^[81]^ PDGDOGDKGDOGPDGKOGPDGPDGPDGDOG KOGPDGPDGPKGKOGPKGKOGKOGKOGKOG | 25.0 | 29.0 | 28.7 |
| 407 | 3 | 30 | n | c | 27 | PKGPKGPKGKOGPDGDOGDOGDOGPKGPKG^[81]^ PDGDOGDOGDOGPDGKOGPDGPDGPDGDOG KOGPDGPDGRKGKOGPKGKOGKOGKOGKOG | 26.1 | 29.7 | 28.9 |
| 408 | 3 | 30 | n | c | 28.9 | PKGPKGPKGKOGPDGDOGDOGDOGPKGPKG^[81]^ PDGDOGDOGDOGPDGKOGPDGPDGPDGDOG KOGPDGPDGKKGKOGPKGKOGKOGKOGKOG | 26.4 | 30.4 | 30.0 |
| 409 | 3 | 30 | n | c | 27 | PKGPKGPKGKOGPDGDOGDOGDOGPKGPKG^[81]^ PDGDOGDOGDOGPDGKOGPDGPDGPDGDOG KOGPDGPDGFKGKOGPKGKOGKOGKOGKOG | 23.5 | 27.8 | 27.8 |
| 410 | 3 | 30 | n | c | 24.7 | PKGPKGPKGKOGPDGDOGDOGDOGPKGPKG^[81]^ PDGDOGDOGDOGPDGKOGPDGPDGPDGDOG KOGPDGPDGWKGKOGPKGKOGKOGKOGKOG | 22.1 | 26.0 | 26.3 |
| 411 | 3 | 30 | n | c | 27.4 | PKGPKGPKGKOGPDGDOGDOGDOGPKGPKG^[81]^ PDGDOGDOGDOGPDGKOGPDGPDGPDGDOG KOGPDGRDGPKGKOGPKGKOGKOGKOGKOG | 26.2 | 29.8 | 28.9 |
| 412 | 3 | 30 | n | c | 28.3 | PKGPKGPKGKOGPDGDOGDOGDOGPKGPKG^[81]^ PDGDOGDOGDOGPDGKOGPDGPDGPDGDOG KOGPDGKDGPKGKOGPKGKOGKOGKOGKOG | 26.5 | 30.5 | 30.0 |
| 413 | 3 | 30 | n | c | 29.4 | PKGPKGPKGKOGPDGDOGDOGDOGPKGPKG^[81]^ PDGDOGDOGDOGPDGKOGPDGPDGPDGDOG KOGPDGFDGPKGKOGPKGKOGKOGKOGKOG | 23.5 | 27.8 | 27.8 |
| 414 | 3 | 30 | n | c | 28 | PKGPKGPKGKOGPDGDOGDOGDOGPKGPKG^[81]^ PDGDOGDOGDOGPDGKOGPDGPDGPDGDOG KOGPDGYDGPKGKOGPKGKOGKOGKOGKOG | 23.8 | 27.9 | 26.0 |
| 415 | 3 | 30 | n | c | 27.5 | PKGPKGPKGKOGPDGDOGDOGDOGPKGPKG^[81]^ PDGDOGDOGDOGPDGKOGPDGPDGPDGDOG KOGPDGWDGPKGKOGPKGKOGKOGKOGKOG | 22.1 | 26.0 | 26.3 |
| 416 | 3 | 30 | n | c | 23.6 | PKGPKGPKGKOGPDGDOGDOGDOGPKGPKG^[81]^ PDGDOGDFGDOGPDGKOGPDGPDGPDGDOG KOGPDGPDGKKGKOGPKGKOGKOGKOGKOG | 20.1 | 24.3 | 23.7 |
| 417 | 3 | 30 | n | c | 25 | PKGPKGPKGKOGPDGDOGDOGDOGPKGPKG^[81]^ PDGDOGDYGDOGPDGKOGPDGPDGPDGDOG KOGPDGPDGKKGKOGPKGKOGKOGKOGKOG | 21.5 | 24.0 | 24.4 |
| 418 | 3 | 30 | n | c | 24.1 | PKGPKGPKGKOGPDGDOGDOGDOGPKGPKG^[81]^ PDGDOGDWGDOGPDGKOGPDGPDGPDGDOG KOGPDGPDGKKGKOGPKGKOGKOGKOGKOG | 20.7 | 23.7 | 23.5 |
| 419 | 3 | 30 | n | c | 26.8 | PKGPKGPKGKOGPDGDOGDOGDOGPKGPKG^[81]^ PDGDOGDRGDOGPDGKOGPDGPDGPDGDOG KOGPDGPDGFKGKOGPKGKOGKOGKOGKOG | 25.9 | 29.7 | 30.9 |
| 420 | 3 | 30 | n | c | 25.1 | PKGPKGPKGKOGPDGDOGDOGDOGPKGPKG^[81]^ PDGDOGDRGDOGPDGKOGPDGPDGPDGDOG KOGPDGPDGWKGKOGPKGKOGKOGKOGKOG | 23.6 | 27.1 | 30.3 |
| 421 | 3 | 30 | n | c | 22.8 | PKGPKGPKGKOGPDGDOGDOGDOGPKGPKG^[81]^ PDGDOGDFGDOGPDGKOGPDGPDGPDGDOG KOGPDGRDGPKGKOGPKGKOGKOGKOGKOG | 16.1 | 19.8 | 22.4 |
| 422 | 3 | 30 | n | c | 36.4 | PKGPKGPKGKOGPEGDOGDOGDOGPKGPKG^[82]^ PDGDOGDOGDOGPDGKOGPDGPDGPDGDOG KOGPDGPDGPKGKOGPKGKOGKOGKOGKOG | 32.9 | 36.0 | 35.3 |
| 423 | 3 | 30 | n | c | 33.1 | PKGPKGPKGKOGPEGDOGDOGDOGPKGPKG^[82]^ PDGDOGDOGDOGPDGAOGPDGPDGPDGDOG KOGPDGPDGPKGKOGPKGKOGKOGKOGKOG | 28.7 | 33.9 | 33.8 |
| 424 | 3 | 30 | n | c | 34.6 | PKGPKGPKGKOGPEGDOGDOGDOGPKGPKG^[82]^ PDGDOGDOGDOGPDGROGPDGPDGPDGDOG KOGPDGPDGPKGKOGPKGKOGKOGKOGKOG | 32.1 | 37.8 | 33.0 |
| 425 | 3 | 30 | n | c | 32.2 | PKGPKGPKGKOGPDGDOGDOGDOGPKGPKG^[82]^ PDGDOGDOGDOGPDGROGPDGPDGPDGDOG KOGPDGPDGPKGKOGPKGKOGKOGKOGKOG | 28.2 | 31.3 | 31.3 |
| 426 | 3 | 30 | n | c | 32 | PKGPKGPKGKOGPDGEOGDOGDOGPKGPKG^[82]^ PDGDOGDOGDOGPDGKOGPDGPDGPDGDOG KOGPDGPDGPKGKOGPKGKOGKOGKOGKOG | 27.2 | 31.0 | 30.1 |
| 427 | 3 | 30 | n | c | 31.2 | PKGPKGPKGKOGPDGEOGDOGDOGPKGPKG^[82]^ PDGDOGDOGDOGPDGKOGPDGPDGPDGDOG KOGPDGPDGPRGKOGPKGKOGKOGKOGKOG | 25.6 | 29.1 | 30.1 |
| 428 | 3 | 30 | n | c | 31.5 | PKGPKGPKGKOGPDGAOGDOGDOGPKGPKG^[82]^ PDGDOGDOGDOGPDGKOGPDGPDGPDGDOG KOGPDGPDGPRGKOGPKGKOGKOGKOGKOG | 27.1 | 31.8 | 30.1 |
| 429 | 3 | 30 | n | c | 30.9 | PKGPKGPKGKOGPDGDOGDOGDOGPKGPKG^[82]^ PDGDOGDOGDOGPDGKOGPDGPDGPDGDOG KOGPDGPDGPRGKOGPKGKOGKOGKOGKOG | 26.1 | 30.2 | 30.4 |
| 430 | 3 | 30 | n | c | 32.4 | PKGPKGPKGKOGPDGDOGDOGDOGPKGPKG^[82]^ PDGDOGDOGDOGPEGKOGPDGPDGPDGDOG KOGPDGPDGPKGKOGPKGKOGKOGKOGKOG | 27.4 | 32.4 | 30.5 |
| 431 | 3 | 30 | n | c | 30.8 | PKGPKGPKGKOGPDGDOGDOGDOGPKGPKG^[82]^ PDGDOGDOGDOGPEGKOGPDGPDGPDGDOG KOGPDGPDGPKGAOGPKGKOGKOGKOGKOG | 26.8 | 30.8 | 30.8 |
| 432 | 3 | 30 | n | c | 32 | PKGPKGPKGKOGPDGDOGDOGDOGPKGPKG^[82]^ PDGDOGDOGDOGPEGKOGPDGPDGPDGDOG KOGPDGPDGPKGROGPKGKOGKOGKOGKOG | 26.9 | 30.7 | 29.6 |
| 433 | 3 | 30 | n | c | 34 | PKGPKGPKGKOGPDGDOGDOGDOGPKGPKG^[82]^ PDGDOGDOGDOGPDGKOGPDGPDGPDGDOG KOGPDGPDGPKGROGPKGKOGKOGKOGKOG | 27.2 | 31.9 | 30.5 |
| 434 | 3 | 30 | n | c | 33.4 | PKGPKGPKGKOGPDGDOGEOGDOGPKGPKG^[82]^ PDGDOGDOGDOGPDGKOGPDGPDGPDGDOG KOGPDGPDGPKGKOGPKGKOGKOGKOGKOG | 28.9 | 32.9 | 32.7 |
| 435 | 3 | 30 | n | c | 31.9 | PKGPKGPKGKOGPDGDOGEOGDOGPKGPKG^[82]^ PDGDOGDOGDOGPDGKOGPDGPDGPDGDOG KOGPDGPDGPKGKOGPRGKOGKOGKOGKOG | 26.9 | 31.1 | 31.3 |
| 436 | 3 | 30 | n | c | 31.9 | PKGPKGPKGKOGPDGDOGAOGDOGPKGPKG^[82]^ PDGDOGDOGDOGPDGKOGPDGPDGPDGDOG KOGPDGPDGPKGKOGPRGKOGKOGKOGKOG | 26.8 | 30.6 | 29.8 |
| 437 | 3 | 30 | n | c | 31.8 | PKGPKGPKGKOGPDGDOGDOGDOGPKGPKG^[82]^ PDGDOGDOGDOGPDGKOGPDGPDGPDGDOG KOGPDGPDGPKGKOGPRGKOGKOGKOGKOG | 26.1 | 30.2 | 30.0 |
| 438 | 2 | 30 | ac | am | 20.5 | PKGPKGFOGPOGFKGFKGPKGPOGFKGPOG^[19]^ PKGDOGDKGPOGPPGDKGDOGDKGPKGDOG | 20.4 | 20.4 | 26.9 |
| 439 | 2 | 30 | ac | am | 22.5 | PKGPKGFOGPOGFKGFKGPKGPOGFKGPOG^[19]^ PRGEPGPRGERGPPGPPGERGPPGEPGEPG | 21.0 | 21.1 | 21.6 |
| 440 | 2 | 30 | ac | am | 12.5 | PKGPKGFOGPOGFKGFKGPKGPOGFKGPOG^[19]^ PQGEPGPQGEQGPPGPPGEQGPPGEPGEPG | 27.8 | 10.1 | 9.9 |
| 441 | 1 | 24 | ac | am | 19 | POGPOGPOGPOGFLGPOGPOGPOG^*^ | 27.0 | 22.5 | 22.9 |
| 443 | 1 | 24 | ac | am | 12.5 | POGPOGPOGPOGFFGPOGPOGPOG^*^ | 23.2 | 18.0 | 17.3 |
| 444 | 1 | 24 | ac | am | 32.5 | POGPOGPOGPDGKOGPOGPOGPOG^[20]^ | 36.3 | 34.7 | 32.4 |
| 445 | 1 | 24 | ac | am | 37.8 | POGPOGPOGPEGKOGPOGPOGPOG^[20]^ | 42.8 | 38.2 | 39.2 |
| 446 | 1 | 30 | ac | am | 28 | POGROGPOGPOGPDGPOGKOGKOGPOGPDG^[20]^ | 27.6 | 24.6 | 30.1 |
| 447 | 1 | 30 | ac | am | 31 | POGPOGPDGPOGROGPOGPOGPDGPOGKOG^[20]^ | 31.6 | 29.3 | 25.5 |
| 448 | 1 | 30 | ac | am | 21.5 | PDGPOGKOGPOGPOGPDGPEGROGPOGPOG^[20]^ | 37.5 | 32.5 | 29.0 |
| 452 | 2 | 30 | ac | am | 43 | POGROGPOGPOGPDGPOGKOGKOGPOGPDG^[20]^ POGPOGPDGPOGROGPOGPOGPDGPOGKOG | 40.2 | 41.8 | 39.1 |
| 453 | 2 | 30 | ac | am | 29.5 | POGROGPOGPOGPDGPOGKOGKOGPOGPDG^[20]^ PDGPOGKOGPOGPOGPDGPEGROGPOGPOG | 40.1 | 39.6 | 36.9 |
| 454 | 2 | 30 | ac | am | 39 | POGPOGPDGPOGROGPOGPOGPDGPOGKOG^[20]^ PDGPOGKOGPOGPOGPDGPEGROGPOGPOG | 41.7 | 41.5 | 35.3 |
| 456 | 2 | 30 | ac | am | 17 | POGPDGPRGKOGPDGYOGPKGYRGPOGDDG^[20]^ KOGPPGYDGPRGKOGPRGKOGPKGYOGPOG | 15.8 | 10.5 | 11.0 |
| 458 | 3 | 30 | ac | am | 43 | POGROGPOGPOGPDGPOGKOGKOGPOGPDG^[20]^ POGPOGPDGPOGROGPOGPOGPDGPOGKOG PDGPOGKOGPOGPOGPDGPEGROGPOGPOG | 47.2 | 50.0 | 49.7 |
| 459 | 3 | 30 | ac | am | 28.5 | POGPDGPRGKOGPDGYOGPKGYRGPOGDDG^[20]^ PDGKRGPOGYOGKDGPOGPDGDRGYOGKOG KOGPPGYDGPRGKOGPRGKOGPKGYOGPOG | 39.2 | 39.6 | 37.9 |
| 460 | 1 | 24 | ac | am | 24 | POGPOGPOGPHGYOGPOGPOGPOG^[18]^ | 25.6 | 25.6 | 23.4 |
| 461 | 1 | 24 | ac | am | 17.5 | POGPOGPOGPOGYHGPOGPOGPOG^[18]^ | 25.0 | 25.2 | 19.5 |
| 462 | 1 | 24 | ac | am | 13.5 | POGPOGPOGPOGWKGPOGPOGPOG^[18]^ | 21.8 | 24.4 | 13.5 |
| 463 | 1 | 24 | ac | am | 21 | POGPOGPOGPOGWRGPOGPOGPOG^[18]^ | 38.5 | 37.9 | 20.3 |
| 464 | 1 | 24 | ac | am | 26 | POGPOGPOGPOGYRGPOGPOGPOG^[18]^ | 35.1 | 34.9 | 26.4 |
| 465 | 2 | 30 | ac | am | 19.5 | PKGPKGYOGPOGYKGYKGPKGPOGYKGPOG^[18]^ PRGEPGPRGERGPPGPPGERGPPGEPGEPG | 22.9 | 23.8 | 21.5 |
| 466 | 3 | 30 | ac | am | 40 | PKGPKGYOGPOGYKGYKGPKGPOGYKGPOG^[18]^ PKGDOGDKGPOGPPGDKGDOGDKGPKGDOG PRGEPGPRGERGPPGPPGERGPPGEPGEPG | 47.4 | 47.3 | 41.3 |
| 467 | 3 | 30 | ac | am | 21 | PKGPKGYOGPOGYKGYKGPKGPOGYKGPOG^[18]^ PKGDOGDKGPOGPPGDKGDOGDKGPKGDOG PAGEPGPAGEAGPPGPPGEAGPPGEPGEPG | 30.8 | 30.0 | 21.4 |
| 470 | 2 | 30 | ac | am | 15.5 | PKGPKGWOGPOGWKGWKGPKGPOGWKGPOG^[18]^ PRGEPGPRGERGPPGPPGERGPPGEPGEPG | 16.9 | 17.1 | 19.1 |
| 471 | 3 | 30 | ac | am | 37 | PKGPKGWOGPOGWKGWKGPKGPOGWKGPOG^[18]^ PKGDOGDKGPOGPPGDKGDOGDKGPKGDOG PRGEPGPRGERGPPGPPGERGPPGEPGEPG | 35.7 | 34.5 | 39.0 |
| 472 | 3 | 30 | ac | am | 19.5 | PKGPKGWOGPOGWKGWKGPKGPOGWKGPOG^[18]^ PKGDOGDKGPOGPPGDKGDOGDKGPKGDOG PAGEPGPAGEAGPPGPPGEAGPPGEPGEPG | 24.2 | 22.5 | 18.2 |
| 483 | 1 | 24 | ac | am | 44 | POGPOGPOGPIGPOGPOGPOGPOG^*^ | 44.1 | 43.2 | 39.8 |
| 484 | 1 | 24 | ac | am | 30 | POGPOGPOGIIGPOGPOGPOGPOG^*^ | 35.7 | 35.3 | 33.3 |
| 486 | 1 | 24 | ac | am | 31 | POGPOGPOGPIGIOGPOGPOGPOG^*^ | 36.3 | 35.7 | 33.9 |
| 487 | 1 | 24 | ac | am | 23 | POGPOGPOGPNGTOGPOGPOGPOG^*^ | 24.7 | 24.7 | 22.6 |
| 488 | 1 | 24 | ac | am | 25.5 | POGPOGPOGPOGTNGPOGPOGPOG^*^ | 24.1 | 24.3 | 24.3 |
| 489 | 1 | 30 | ac | am | 45.5 | PKGEOGPOGPOGPKGEOGPOGPOGPKGEOG^[83]^ | 53.8 | 42.2 | 48.6 |
| 492 | 1 | 30 | ac | am | 39.5 | PKGEOGPOGPOGFOGERGPOGPOGPKGEOG^[84]^ | 39.7 | 40.6 | 39.6 |
| 493 | 1 | 30 | ac | am | 34.5 | PKGEOGPOGPOGVOGEAGPOGPOGPKGEOG^*^ | 36.4 | 36.9 | 33.6 |
| 494 | 1 | 24 | ac | am | 42 | PKGEOGPOGPOGPOGPOGPOGPOG^*^ | 50.0 | 48.4 | 46.8 |
| 495 | 1 | 24 | ac | am | 36 | PKGEOGPOGPOGPOGPOGPKGEOG^*^ | 44.1 | 42.9 | 38.4 |
| 496 | 1 | 24 | ac | am | 43.5 | POGPOGPOGPOGPOGPOGPKGEOG^*^ | 45.3 | 44.9 | 39.2 |
| 497 | 1 | 24 | ac | am | 29.5 | PKGPOGPOGPOGPOGPOGPKGPOG^*^ | 41.7 | 40.0 | 36.3 |
| 500 | 1 | 36 | n | am | 36 | PKGPKGPKGPKGPOGPOGPOGPOGDOGDOGDOGDOG^[85]^ | 21.6 | 14.1 | 29.5 |
| 501 | 1 | 24 | ac | am | 40 | GPOGPOGPOGPQGFOGPOGPOGPO^[86]^ | 33.0 | 30.5 | 35.6 |
| 502 | 1 | 25 | n | c | 40.7 | GGPOGPOGPOGPOGPOGPOGPOGGG^[87]^ | 26.9 | 30.1 | 38.9 |
| 503 | 1 | 25 | n | c | 38.4 | KGPOGPOGPOGPOGPOGPOGPOGGG^[87]^ | 26.9 | 30.8 | 40.7 |
| 504 | 1 | 25 | n | c | 40.7 | EGPOGPOGPOGPOGPOGPOGPOGGG^[87]^ | 27.0 | 31.1 | 39.6 |
| 505 | 1 | 25 | ac | c | 41.6 | KGPOGPOGPOGPOGPOGPOGPOGGG^[87]^ | 33.5 | 39.5 | 39.7 |
| 506 | 1 | 25 | ac | c | 40.8 | EGPOGPOGPOGPOGPOGPOGPOGGG^[87]^ | 33.6 | 39.9 | 38.6 |
| 507 | 1 | 25 | n | c | 42 | GGPOGPOGPOGPOGPOGPOGPOGGK^[87]^ | 27.2 | 30.3 | 40.0 |
| 508 | 1 | 25 | n | c | 38.7 | GGPOGPOGPOGPOGPOGPOGPOGGE^[87]^ | 26.0 | 29.0 | 40.9 |
| 509 | 1 | 25 | n | am | 39.7 | GGPOGPOGPOGPOGPOGPOGPOGGK^[87]^ | 31.5 | 30.3 | 41.2 |
| 510 | 1 | 25 | n | am | 40.1 | GGPOGPOGPOGPOGPOGPOGPOGGE^[87]^ | 30.3 | 29.0 | 42.1 |
| 511 | 1 | 25 | n | c | 41.6 | EGPOGPOGPOGPOGPOGPOGPOGGK^[87]^ | 27.4 | 31.3 | 40.7 |
| 512 | 1 | 25 | n | c | 34.2 | KGPOGPOGPOGPOGPOGPOGPOGGE^[87]^ | 26.1 | 29.7 | 42.7 |
| 513 | 1 | 25 | n | c | 41.4 | DGPOGPOGPOGPOGPOGPOGPOGGG^[87]^ | 26.9 | 29.9 | 36.9 |
| 514 | 1 | 25 | n | c | 35.8 | GGPOGPOGPOGPOGPOGPOGPOGGD^[87]^ | 26.9 | 30.1 | 38.3 |
| 515 | 3 | 32 | n | c | 35.5 | YGPKGPKGPKGKOGPDGDOGDOGDOGPKGPKG^[28]^ YGPDGDOGDOGDOGPDGKOGPDGPDGPDGDOG YGKOGPDGPDGPKGKOGPKGKOGKOGKOGKOG | 24.6 | 21.8 | 35.1 |
| 516 | 3 | 32 | n | c | 34.2 | YGPKGPKGPKGKOGPDGDOGDOGEOGPKGPKG^[28]^ YGPDGDOGDOGDOGPDGKOGPDGPDGPDGDOG YGKOGPDGPDGPKGKOGPKGKOGKOGKOGKOG | 23.7 | 20.8 | 33.8 |
| 517 | 3 | 32 | n | c | 36 | YGPKGPKGPKGKOGPDGDOGEOGDOGPKGPKG^[28]^ YGPDGDOGDOGDOGPDGKOGPDGPDGPDGDOG YGKOGPDGPDGPKGKOGPKGKOGKOGKOGKOG | 25.5 | 22.7 | 36.3 |
| 518 | 3 | 32 | n | c | 35 | YGPKGPKGPKGKOGPDGDOGEOGEOGPKGPKG^[28]^ YGPDGDOGDOGDOGPDGKOGPDGPDGPDGDOG YGKOGPDGPDGPKGKOGPKGKOGKOGKOGKOG | 24.6 | 21.7 | 35.0 |
| 519 | 3 | 32 | n | c | 33.6 | YGPKGPKGPKGKOGPDGDOGDOGDOGPKGPKG^[28]^ YGPDGDOGDOGDOGPDGKOGPDGPDGPDGDOG YGKOGPDGPDGPKGKOGPRGKOGKOGKOGKOG | 22.7 | 20.0 | 33.7 |
| 520 | 3 | 32 | n | c | 33.8 | YGPKGPKGPKGKOGPDGDOGDOGEOGPKGPKG^[28]^ YGPDGDOGDOGDOGPDGKOGPDGPDGPDGDOG YGKOGPDGPDGPKGKOGPRGKOGKOGKOGKOG | 22.2 | 19.2 | 33.4 |
| 521 | 3 | 32 | n | c | 33.9 | YGPKGPKGPKGKOGPDGDOGEOGDOGPKGPKG^[28]^ YGPDGDOGDOGDOGPDGKOGPDGPDGPDGDOG YGKOGPDGPDGPKGKOGPRGKOGKOGKOGKOG | 23.5 | 20.9 | 35.0 |
| 522 | 3 | 32 | n | c | 34.2 | YGPKGPKGPKGKOGPDGDOGEOGEOGPKGPKG^[28]^ YGPDGDOGDOGDOGPDGKOGPDGPDGPDGDOG YGKOGPDGPDGPKGKOGPRGKOGKOGKOGKOG | 23.8 | 21.0 | 34.7 |
| 523 | 3 | 32 | n | c | 32.4 | YGPKGPKGPKGKOGPDGDOGAOGAOGPKGPKG^[28]^ YGPDGDOGDOGDOGPDGKOGPDGPDGPDGDOG YGKOGPDGPDGPKGKOGPAGKOGKOGKOGKOG | 21.0 | 18.0 | 31.0 |
| 524 | 3 | 32 | n | c | 35.8 | YGPKGPKGPKGKOGPDGDOGDOGAOGPKGPKG^[28]^ YGPDGDOGDOGDOGPDGKOGPDGPDGPDGDOG YGKOGPDGPDGPKGKOGPKGKOGKOGKOGKOG | 21.8 | 20.0 | 30.4 |
| 525 | 3 | 32 | n | c | 35.8 | YGPKGPKGPKGKOGPDGDOGAOGDOGPKGPKG^[28]^ YGPDGDOGDOGDOGPDGKOGPDGPDGPDGDOG YGKOGPDGPDGPKGKOGPKGKOGKOGKOGKOG | 25.3 | 22.3 | 35.5 |
| 526 | 3 | 32 | n | c | 33.8 | YGPKGPKGPKGKOGPDGDOGEOGAOGPKGPKG^[28]^ YGPDGDOGDOGDOGPDGKOGPDGPDGPDGDOG YGKOGPDGPDGPKGKOGPKGKOGKOGKOGKOG | 22.6 | 20.9 | 31.6 |
| 527 | 3 | 32 | n | c | 34.2 | YGPKGPKGPKGKOGPDGDOGAOGEOGPKGPKG^[28]^ YGPDGDOGDOGDOGPDGKOGPDGPDGPDGDOG YGKOGPDGPDGPKGKOGPKGKOGKOGKOGKOG | 24.4 | 21.3 | 34.2 |
| 528 | 3 | 32 | n | c | 33.1 | YGPKGPKGPKGKOGPDGDOGDOGAOGPKGPKG^[28]^ YGPDGDOGDOGDOGPDGKOGPDGPDGPDGDOG YGKOGPDGPDGPKGKOGPRGKOGKOGKOGKOG | 23.7 | 21.6 | 33.4 |
| 529 | 3 | 32 | n | c | 33.6 | YGPKGPKGPKGKOGPDGDOGAOGDOGPKGPKG^[28]^ YGPDGDOGDOGDOGPDGKOGPDGPDGPDGDOG YGKOGPDGPDGPKGKOGPRGKOGKOGKOGKOG | 23.4 | 20.5 | 33.4 |
| 530 | 3 | 32 | n | c | 33.8 | YGPKGPKGPKGKOGPDGDOGEOGAOGPKGPKG^[28]^ YGPDGDOGDOGDOGPDGKOGPDGPDGPDGDOG YGKOGPDGPDGPKGKOGPRGKOGKOGKOGKOG | 24.5 | 22.5 | 34.7 |
| 531 | 3 | 32 | n | c | 34.1 | YGPKGPKGPKGKOGPDGDOGAOGEOGPKGPKG^[28]^ YGPDGDOGDOGDOGPDGKOGPDGPDGPDGDOG YGKOGPDGPDGPKGKOGPRGKOGKOGKOGKOG | 22.9 | 19.4 | 33.2 |
| 532 | 1 | 24 | ac | am | 40.8^†^ | GPOGPOGPOGAOGPOGPOGPOGPO^[13][63]^ | 42.2 | 41.6 | 41.0 |
| 533 | 1 | 24 | ac | am | 39^†^ | GPOGPOGPOGKRGPOGPOGPOGPO^[16][69]^ | 38.4 | 38.7 | 39.6 |
| 534 | 1 | 24 | ac | am | 39.6^†^ | GPOGPOGPOGPAGPOGPOGPOGPO^[13][63]^ | 41.2 | 40.8 | 39.8 |
| 535 | 1 | 24 | ac | am | 32^†^ | GPOGPOGPOGPDGPOGPOGPOGPO^[13][62]^ | 34.6 | 33.3 | 33.3 |
| 536 | 1 | 24 | ac | am | 46^†^ | GPOGPOGPOGPKGDOGPOGPOGPO^[15][73]^ | 42.9 | 43.1 | 41.6 |
| 537 | 1 | 24 | ac | am | 47.4^†^ | GPOGPOGPOGPKGEOGPOGPOGPO^[15][73]^ | 41.3 | 41.7 | 40.1 |
| 538 | 1 | 24 | ac | am | 46.3^†^ | GPOGPOGPOGPOGPOGPOGPOGPO^[13][63][69]^ | 47.5 | 47.8 | 47.4 |
| 539 | 1 | 24 | ac | am | 45.9^†^ | GPOGPOGPOGPRGPOGPOGPOGPO^[13][62]^ | 43.8 | 44.2 | 45.1 |
| 540 | 1 | 24 | ac | am | 29.1^†^ | POGPOGPOGPKGFOGPOGPOGPOG^[17][19]^ | 30.4 | 30.9 | 30.6 |
| 541 | 1 | 24 | ac | am | 41.2^†^ | POGPOGPOGPRGFOGPOGPOGPOG^[17][19]^ | 40.9 | 40.6 | 39.4 |
| 542 | 1 | 24 | ac | am | 46.4 | POGPOGPOGPMGPOGPOGPOGPOG^*^ | 46.4 | 44.9 | 45.3 |
| 543 | 1 | 24 | ac | am | 39.8 | POGPOGPOGLMGPOGPOGPOGPOG^*^ | 39.9 | 38.5 | 36.3 |
| 544 | 1 | 24 | ac | am | 40.5 | POGPOGPOGPMGLOGPOGPOGPOG^*^ | 40.5 | 39.0 | 39.6 |
| 545 | 1 | 24 | ac | am | 38 | POGPOGPOGIMGPOGPOGPOGPOG^*^ | 38.0 | 38.0 | 38.3 |
| 546 | 1 | 24 | ac | am | 38.6 | POGPOGPOGPMGIOGPOGPOGPOG^*^ | 38.6 | 37.4 | 38.1 |
| 547 | 1 | 24 | ac | am | 37 | POGPOGPOGMMGPOGPOGPOGPOG^*^ | 37.0 | 37.2 | 33.0 |
| 548 | 1 | 24 | ac | am | 37.4 | POGPOGPOGPMGMOGPOGPOGPOG^*^ | 37.4 | 37.7 | 35.4 |
| 549 | 1 | 24 | ac | am | 41.8 | POGPOGPOGMOGPOGPOGPOGPOG^*^ | 41.9 | 43.2 | 40.3 |
| 551 | 1 | 24 | ac | am | 43.3 | POGPOGPOGIOGPOGPOGPOGPOG^*^ | 43.3 | 43.0 | 40.2 |
| 552 | 1 | 30 | n | c | 17.5 | POGPOGPOGITGARGLAGPOGPOGPOGPOG^[50]^ | 27.1 | 28.6 | 22.0 |
| 553 | 1 | 30 | n | c | 12 | GPSGPRGLOGPOGAOGPQGEOGEOGASGPM^[24]^ | 27.7 | 12.8 | 15.6 |
| 554 | 1 | 24 | ac | am | 43.5 | POGPOGPOGPOGKOGPOGPOGPOG^[20]^ | 46.5 | 45.8 | 43.3 |
| 555 | 1 | 24 | ac | am | 42.5 | POGPOGPOGPOGROGPOGPOGPOG^[20]^ | 45.5 | 43.8 | 39.8 |
| 556 | 1 | 24 | ac | am | 34.5 | POGPOGPOGPDGPOGPOGPOGPOG^[20]^ | 38.3 | 35.9 | 33.4 |
| 557 | 1 | 24 | ac | am | 41.4 | POGPOGPOGPEGPOGPOGPOGPOG^[20]^ | 44.3 | 43.5 | 41.4 |
| 558 | 1 | 24 | ac | am | 30.5 | POGPOGPOGPDGROGPOGPOGPOG^[20]^ | 34.3 | 33.8 | 31.1 |
| 559 | 1 | 24 | ac | am | 35 | POGPOGPOGPEGROGPOGPOGPOG^[20]^ | 40.7 | 39.5 | 33.5 |
| 560 | 1 | 24 | ac | am | 42.5 | POGPOGPOGPOGEOGPOGPOGPOG^[20]^ | 45.1 | 45.9 | 43.1 |
| 561 | 1 | 24 | ac | am | 37.5 | POGPOGPOGPOGKDGPOGPOGPOG^[20]^ | 39.1 | 37.9 | 36.2 |
| 562 | 1 | 24 | ac | am | 37 | POGPOGPOGPOGRDGPOGPOGPOG^[20]^ | 37.1 | 37.0 | 33.0 |
| 563 | 1 | 24 | ac | am | 36 | POGPOGPOGPOGKEGPOGPOGPOG^[20]^ | 39.8 | 41.3 | 36.9 |
| 564 | 1 | 24 | ac | am | 35 | POGPOGPOGPOGREGPOGPOGPOG^[20]^ | 38.4 | 37.2 | 34.0 |
| 565 | 1 | 24 | ac | am | 37.5 | POGPOGPOGEKGEOGPOGPOGPOG^[20]^ | 39.1 | 38.4 | 37.8 |
| 566 | 1 | 24 | ac | am | 31 | POGPOGPOGKEGKOGPOGPOGPOG^[20]^ | 37.4 | 37.6 | 34.8 |
| 567 | 1 | 24 | ac | am | 38.5 | POGPOGPOGERGEOGPOGPOGPOG^[20]^ | 39.8 | 38.9 | 39.0 |
| 568 | 1 | 24 | ac | am | 28.5 | POGPOGPOGREGROGPOGPOGPOG^[20]^ | 34.4 | 32.4 | 26.1 |
| 569 | 1 | 24 | ac | am | 34 | POGPOGPOGRDGROGPOGPOGPOG^[20]^ | 33.6 | 33.9 | 30.8 |
| 570 | 1 | 24 | ac | am | 37 | POGPOGPOGDRGDOGPOGPOGPOG^[20]^ | 34.5 | 34.1 | 35.4 |
| 571 | 1 | 24 | ac | am | 35.5 | POGPOGPOGKDGKOGPOGPOGPOG^[20]^ | 37.1 | 36.3 | 35.2 |
| 572 | 1 | 24 | ac | am | 58.5 | GPOGPOGPOGPOGPOGPOGPOGPO^*^ | 47.8 | 47.5 | 47.4 |
| 573 | 3 | 30 | ac | am | 58.5 | PKGPKGPOGPOGPKGDKGPKGPOGDOGPOG^[37]^ PKGDKGDOGPKGPOGDKGDOGDKGPKGPOG POGDOGDOGPKGDOGPOGDKGPOGDOGDKG | 62.4 | 62.4 | 60.5 |
| 574 | 2 | 30 | ac | am | 31 | PKGPKGPOGPOGPKGDKGPKGPOGDOGPOG^[37]^ PKGDKGDOGPKGPOGDKGDOGDKGPKGPOG | 41.4 | 40.8 | 39.1 |
| 575 | 2 | 30 | ac | am | 37 | PKGPKGPOGPOGPKGDKGPKGPOGDOGPOG^[37]^ POGDOGDOGPKGDOGPOGDKGPOGDOGDKG | 39.9 | 40.6 | 41.0 |
| 576 | 2 | 30 | ac | am | 29.5 | PKGDKGDOGPKGPOGDKGDOGDKGPKGPOG^[37]^ POGDOGDOGPKGDOGPOGDKGPOGDOGDKG | 37.5 | 37.5 | 39.7 |
| 577 | 1 | 30 | ac | am | 20 | PKGPKGPOGPOGPKGDKGPKGPOGDOGPOG^[37]^ | 31.2 | 30.2 | 31.1 |
| 578 | 1 | 30 | ac | am | 21 | PKGDKGDOGPKGPOGDKGDOGDKGPKGPOG^[37]^ | 24.6 | 24.3 | 29.0 |
| 579 | 1 | 30 | ac | am | 20.5 | POGDOGDOGPKGDOGPOGDKGPOGDOGDKG^[37]^ | 19.9 | 20.8 | 23.1 |
| 580 | 3 | 30 | ac | am | 23.5 | KPGYPGDRGPOGQRGKRGPPGDRGFPGFOG^[37]^ PRGLKGLOGAQGPOGAKGLKGPRGYKGLOG PKGYOGDOGIAGPDGPKGDQGDRGAOGDOG | 41.8 | 49.9 | 21.6 |
| 581 | 2 | 30 | ac | am | 12.5 | PRGLKGLOGAQGPOGAKGLKGPRGYKGLOG^[37]^ PKGYOGDOGIAGPDGPKGDQGDRGAOGDOG | 19.2 | 13.7 | 9.4 |
| 582 | 3 | 30 | ac | am | 44 | PKGROGPKGFOGYOGPRGROGKKGPRGPOG^[37]^ SKGDOGPOGDRGPKGPOGYKGPOGDKGFRG PDGDRGPRGPOGYOGDDGPEGDOGPPGDOG | 48.0 | 49.7 | 45.7 |
| 583 | 2 | 30 | ac | am | 17.5 | PKGROGPKGFOGYOGPRGROGKKGPRGPOG^[37]^ SKGDOGPOGDRGPKGPOGYKGPOGDKGFRG | 18.2 | 18.6 | 14.3 |
| 584 | 2 | 30 | ac | am | 17 | PKGROGPKGFOGYOGPRGROGKKGPRGPOG^[37]^ PDGDRGPRGPOGYOGDDGPEGDOGPPGDOG | 14.5 | 15.9 | 14.5 |
| 585 | 2 | 31 | ac | am | 14 | SKGDOGPOGDRGPKGPOGYKGPOGDKGFRG^[37]^ PDGDRGPRGPOGYOGDDGPEGDOGPPGDOG | 15.6 | 20.6 | 19.1 |

## **1.2. CD melting curve, derivatives, mass spectra and UPLC of new peptides in the library**

**Table S2.** Sequences, expected and observed m/z of previously unpublished peptides in Library 1.2

| Peptide | Sequence | Expected | Observed |
| --- | --- | --- | --- |
| 441 | POGPOGPOGPOGFLGPOGPOGPOG | [M+2H]^2+^ 1124.7 | [M+2H]^2+^ 1124.7 |
| 443 | POGPOGPOGPOGFFGPOGPOGPOG | [M+Na]^+^  2304.4 | [M+Na]^+^  2304.8 |
| 483 | POGPOGPOGPIGPOGPOGPOGPOG | [M+Na]^+^  2220.4 | [M+Na]^+^  2220.3 |
| 484 | POGPOGPOGIIGPOGPOGPOGPOG | [M+Na]^+^  2236.4 | [M+Na]^+^  2236.3 |
| 486 | POGPOGPOGPIGIOGPOGPOGPOG | [M+Na]^+^  2236.4 | [M+Na]^+^  2236.2 |
| 487 | POGPOGPOGPNGTOGPOGPOGPOG | [M+Na]^+^  2225.3 | [M+Na]^+^  2225.1 |
| 488 | POGPOGPOGPOGTNGPOGPOGPOG | [M+Na]^+^  2225.3 | [M+Na]^+^  2225.1 |
| 493 | PKGEOGPOGPOGVOGEAGPOGPOGPKGEOG | [M]  2817.9 | [M]  2817.1 |
| 494 | PKGEOGPOGPOGPOGPOGPOGPOG | [M+Na]^+^  2267.4 | [M+Na]^+^  2266.9 |
| 495 | PKGEOGPOGPOGPOGPOGPKGEOG | [M+H]^+^  2292.4 | [M+H]^+^  2292.3 |
| 496 | POGPOGPOGPOGPOGPOGPKGEOG | [M+Na]^+^  2267.4 | [M+Na]^+^  2266.9 |
| 542 | POGPOGPOGPMGPOGPOGPOGPOG | [M+Na]^+^  2238.4 | [M+Na]^+^  2238.4 |
| 543 | POGPOGPOGLMGPOGPOGPOGPOG | [M+Na]^+^  2254.4 | [M+Na]^+^  2254.5 |
| 544 | POGPOGPOGPMGLOGPOGPOGPOG | [M+Na]^+^  2254.4 | [M+Na]^+^  2254.5 |
| 545 | POGPOGPOGIMGPOGPOGPOGPOG | [M+Na]^+^  2254.4 | [M+Na]^+^ 2254.2 |
| 546 | POGPOGPOGPMGIOGPOGPOGPOG | [M+Na]^+^ 2254.4 | [M+Na]^+^ 2254.2 |
| 547 | POGPOGPOGMMGPOGPOGPOGPOG | [M+Na]^+^  2272.5 | [M+Na]^+^  2272.4 |
| 548 | POGPOGPOGPMGMOGPOGPOGPOG | [M+Na]^+^  2272.5 | [M+Na]^+^  2272.4 |
| 549 | POGPOGPOGMOGPOGPOGPOGPOG | [M+Na]^+^  2231.4 | [M+Na]^+^  2254.4 |
| 551 | POGPOGPOGIOGPOGPOGPOGPOG | [M+Na]^+^ 2236.4 | [M+Na]^+^ 2235.8 |
| 572 | GPOGPOGPOGPOGPOGPOGPOGPO | [M+2Na]^2+^  1121.6  [M+H+2Na]^3+^  748.1 | [M+2Na]^2+^  1122.8  [M+H+2Na]^3+^  748.8 |

Sequences and mass spectra data for peptides synthesized and characterized for the first time in this publication.

**
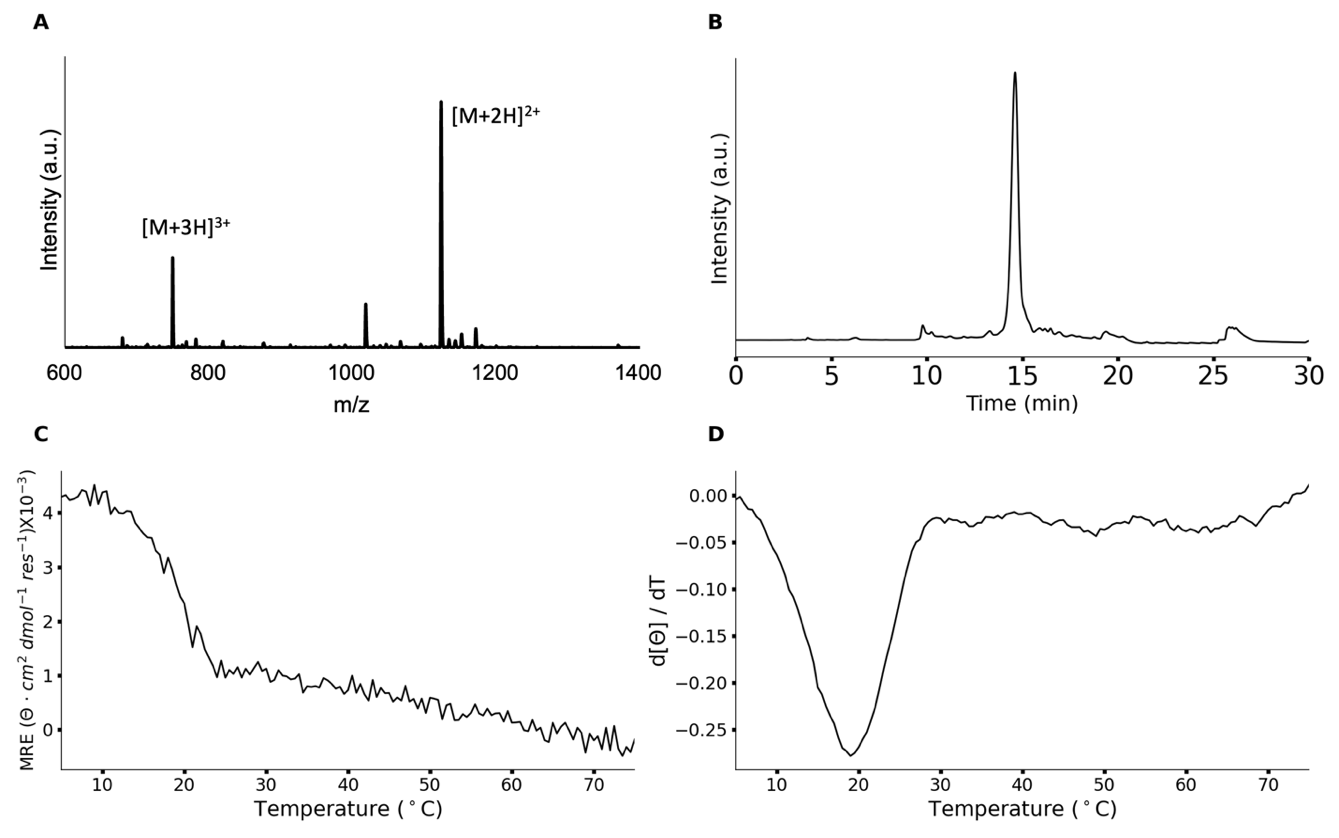
**

**Figure S2.** Characterization of peptide 441: A) ESI mass spectrum B) HPLC trace, CD C) Melting curve D) First derivative of the melt


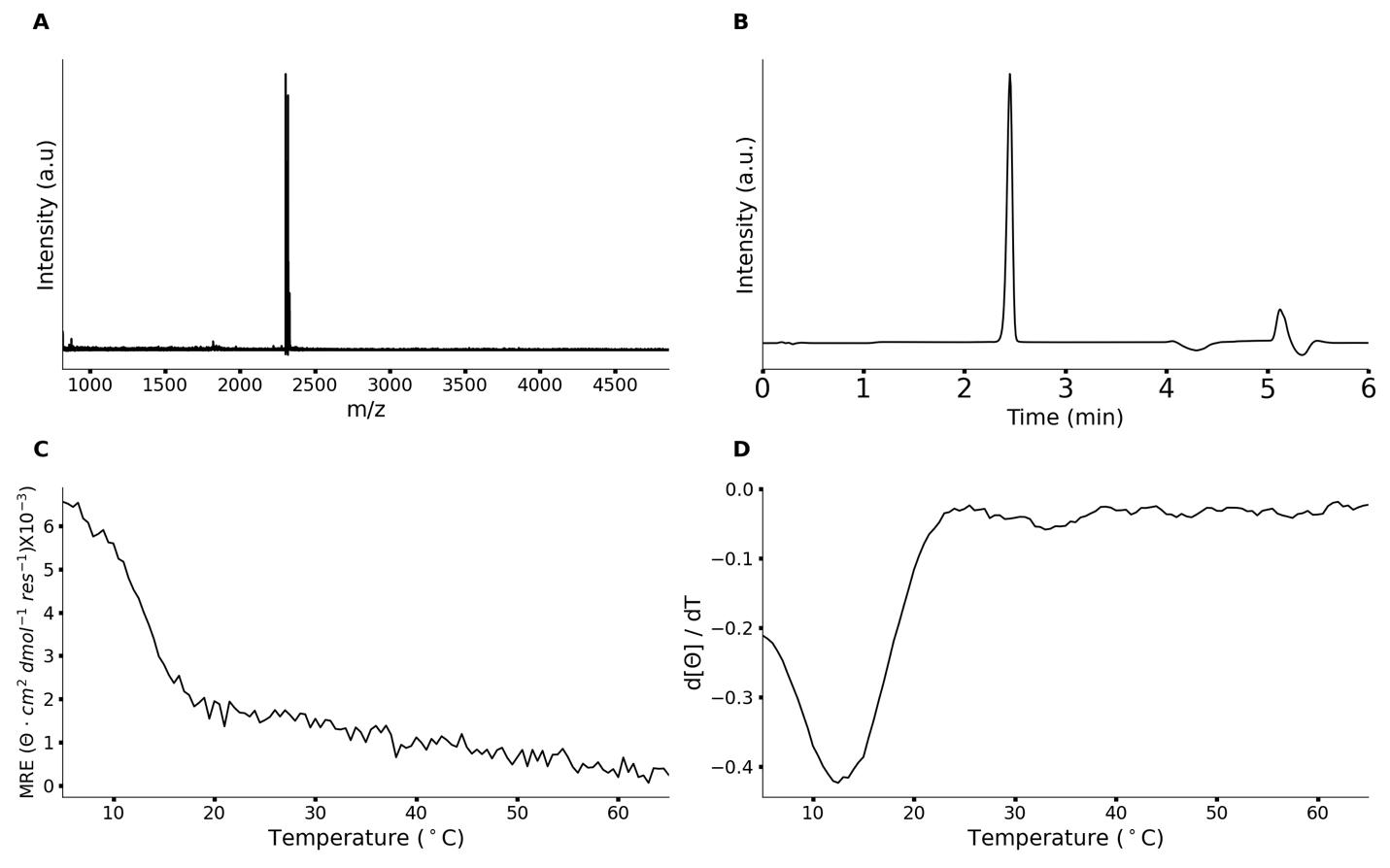


**Figure S3.** Characterization of peptide 443: (A) MALDI-ToF mass spectrum, (B) UPLC trace, CD C) Melting curve (D) First derivative of the melt


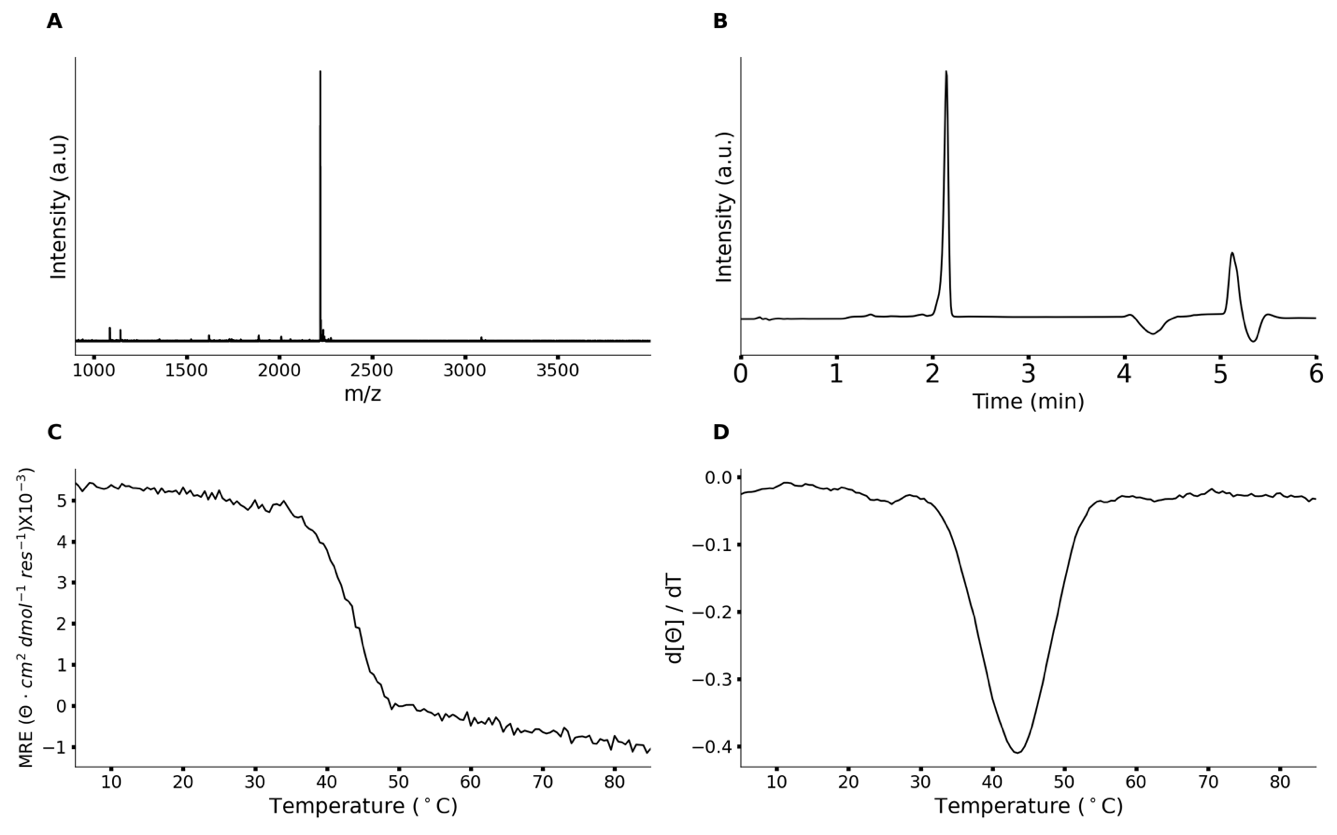


**Figure S4.** Characterization of peptide 483: (A) MALDI-ToF mass spectrum, (B) UPLC trace, CD C) Melting curve (D) First derivative of the melt


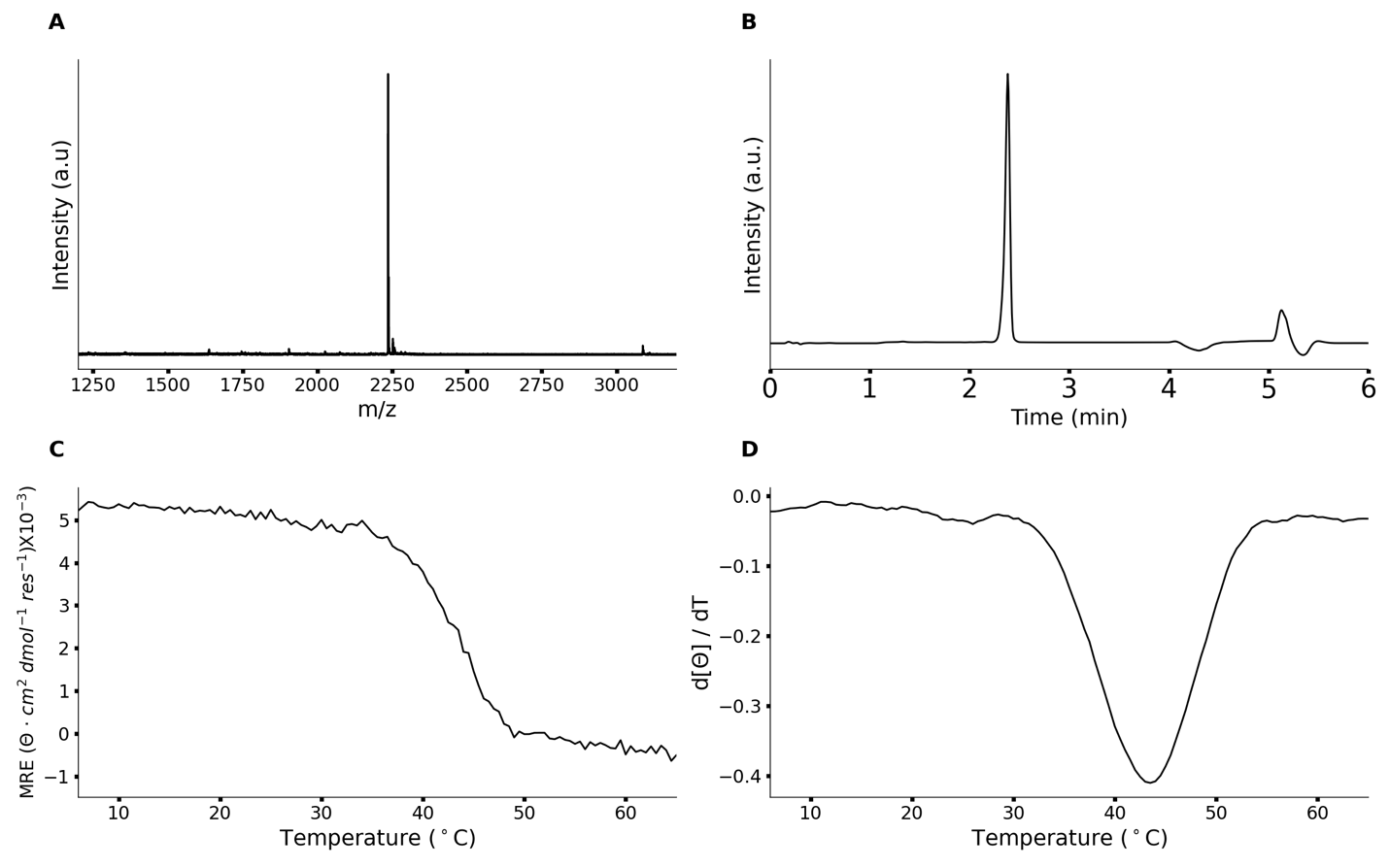


**Figure S5.** Characterization of peptide 484: (A) MALDI-ToF mass spectrum, (B) UPLC trace, CD C) Melting curve (D) First derivative of the melt


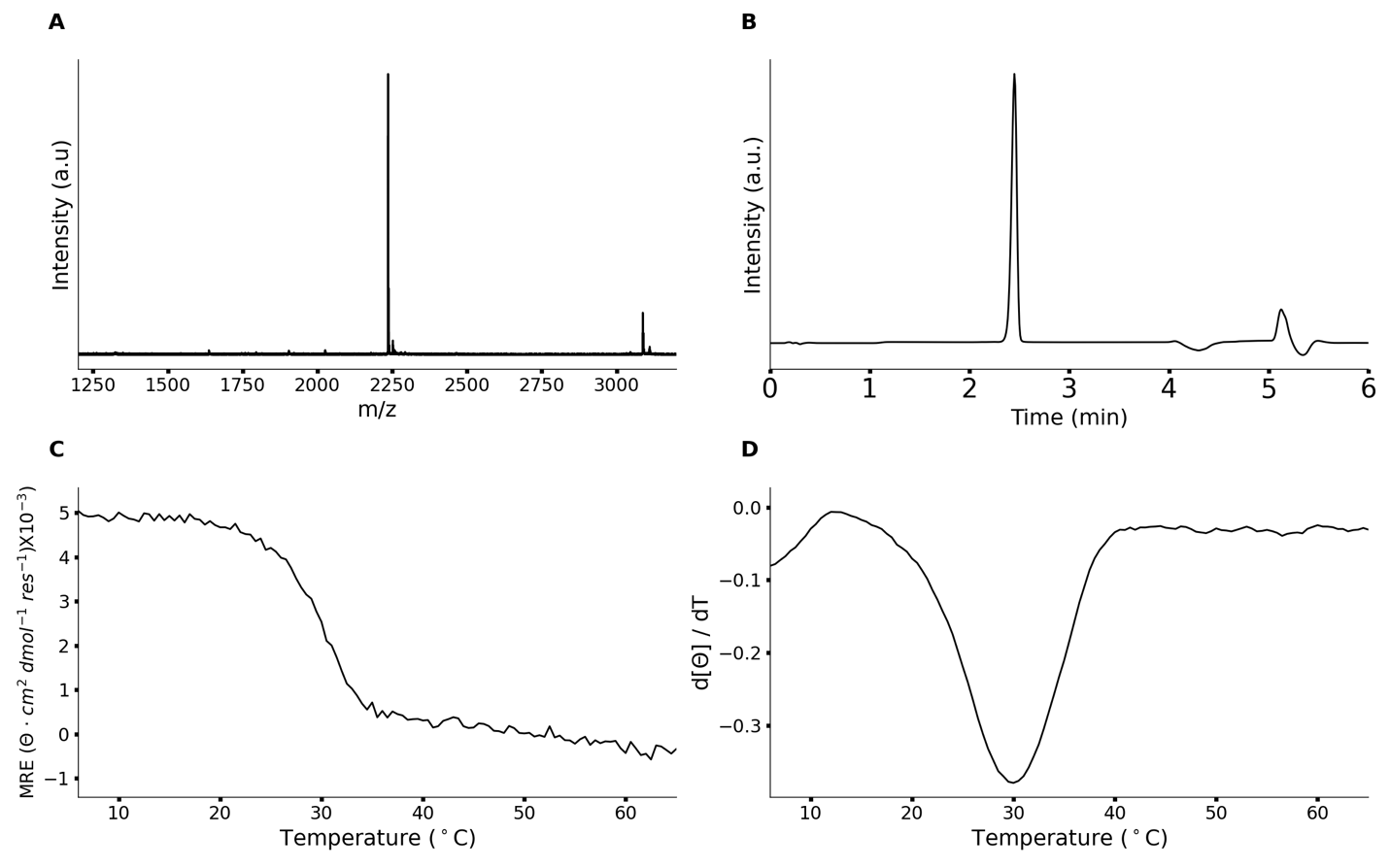


**Figure S6.** Characterization of peptide 486: (A) MALDI-ToF mass spectrum, (B) UPLC trace, CD C) Melting curve (D) First derivative of the melt


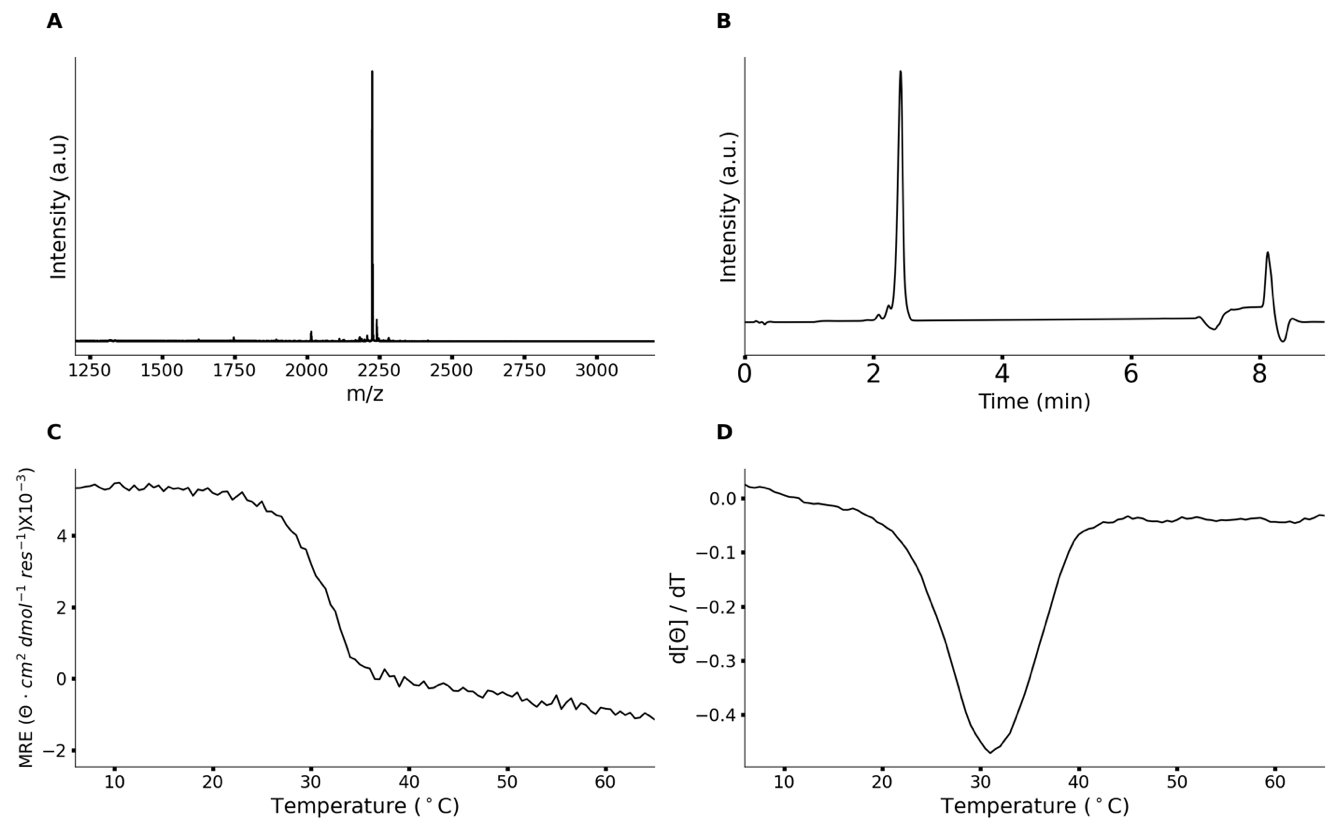


**Figure S7.** Characterization of peptide 487: (A) MALDI-ToF mass spectrum, (B) UPLC trace, CD C) Melting curve (D) First derivative of the melt


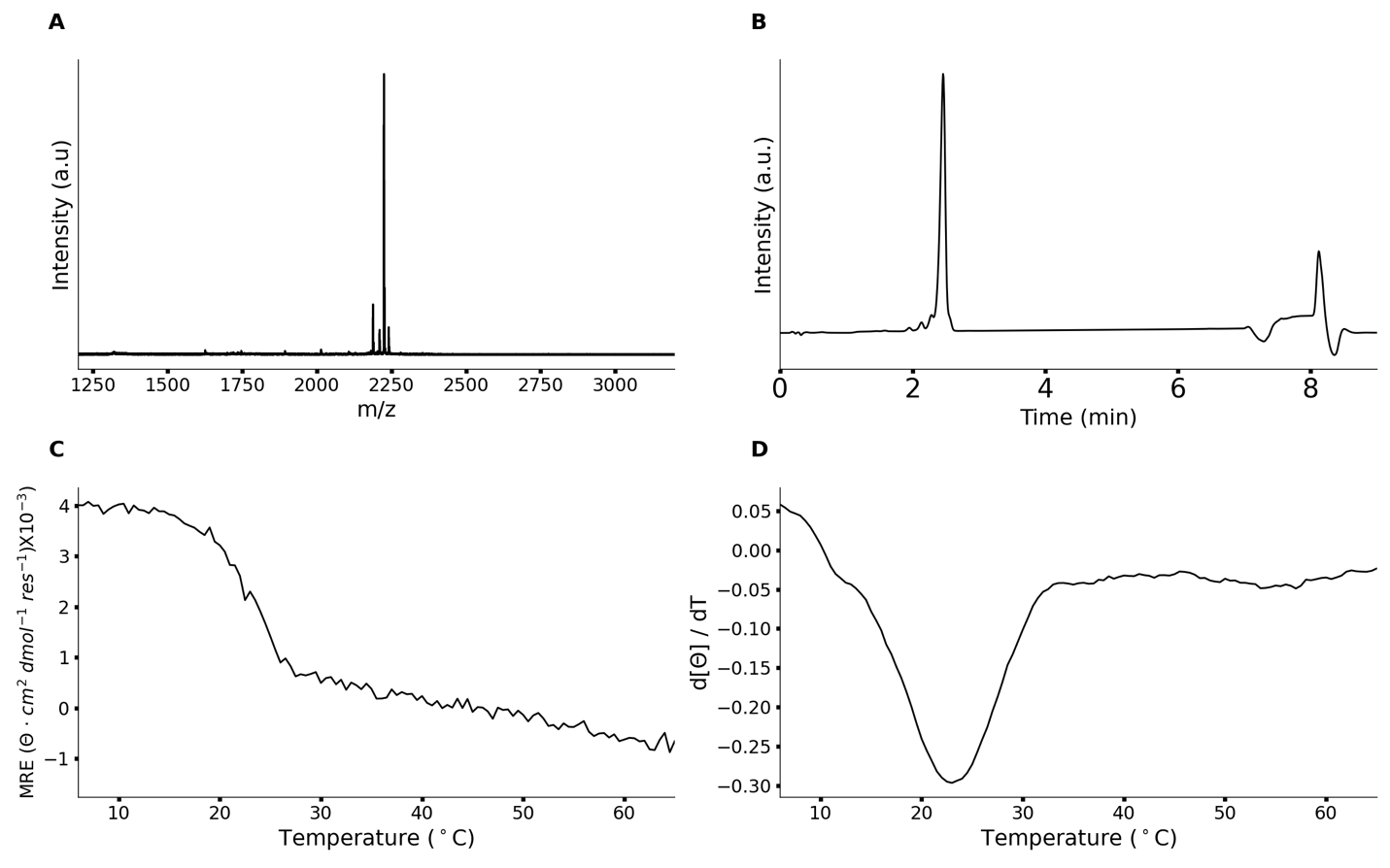


**Figure S8.** Characterization of peptide 488: (A) MALDI-ToF mass spectrum, (B) UPLC trace, CD C) Melting curve (D) First derivative of the melt


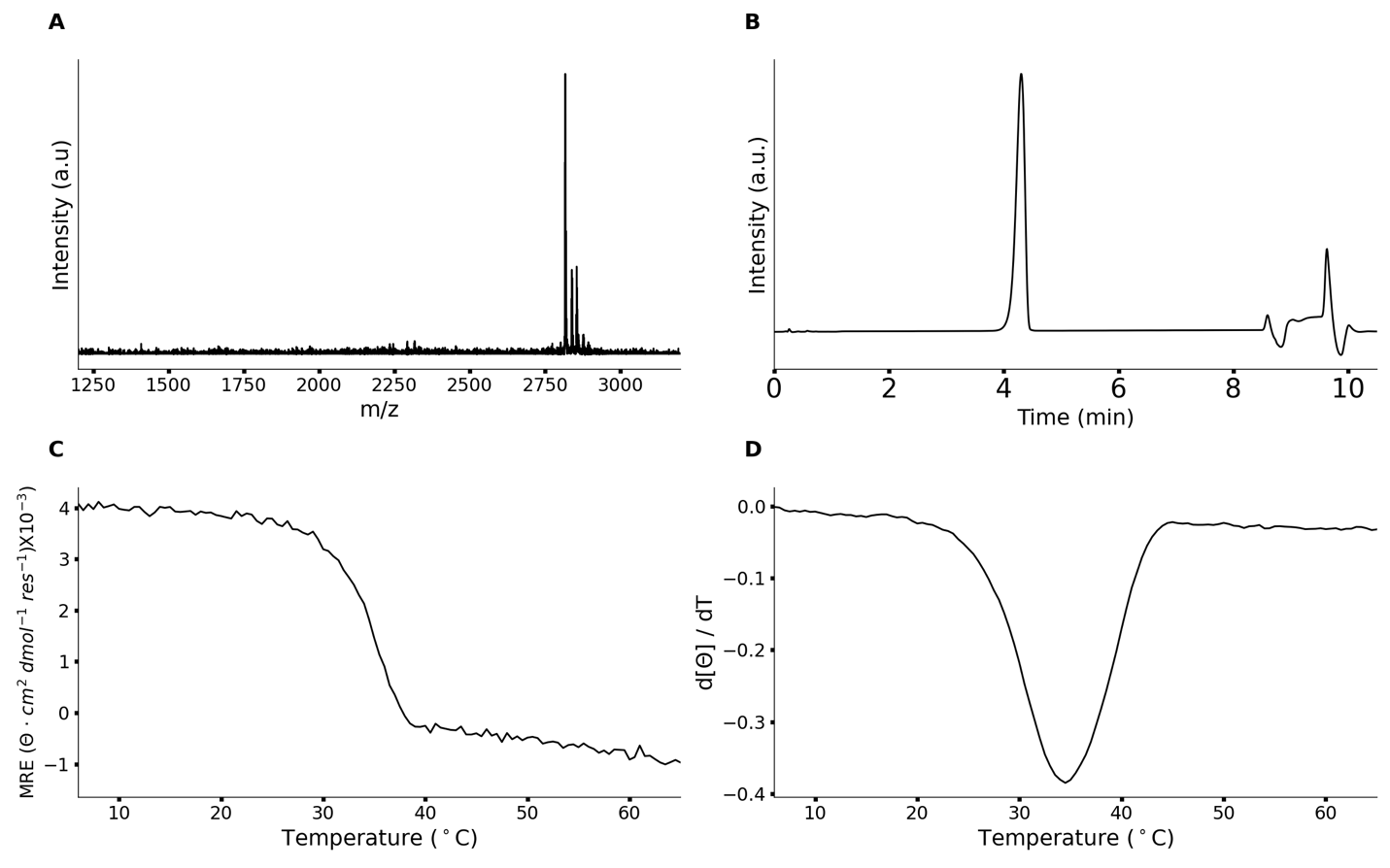


**Figure S9.** Characterization of peptide 493: (A) MALDI-ToF mass spectrum, (B) UPLC trace, CD C) Melting curve (D) First derivative of the melt

**
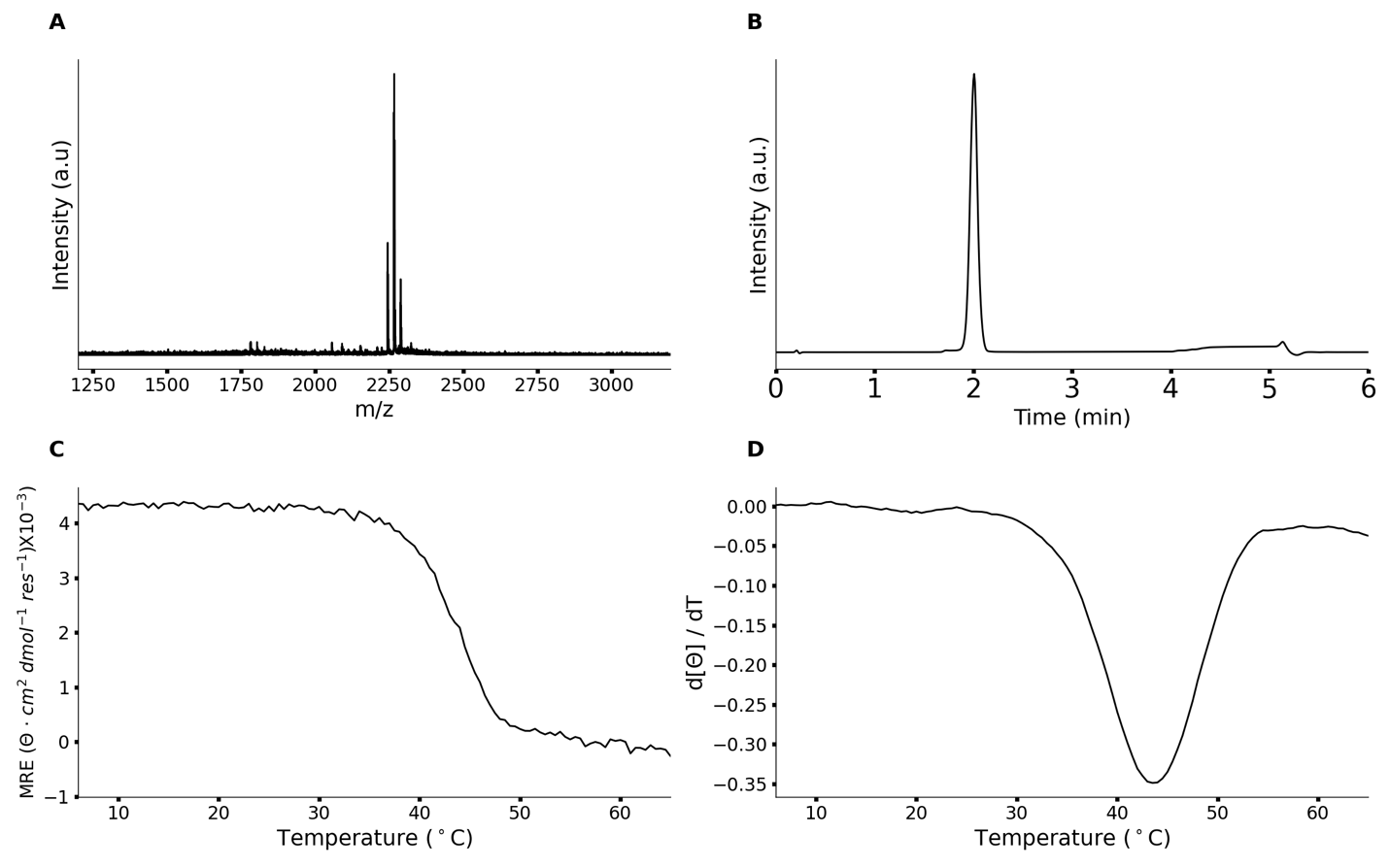
**

**Figure S10.** Characterization of peptide 494: (A) MALDI-ToF mass spectrum, (B) UPLC trace, CD C) Melting curve (D) First derivative of the melt


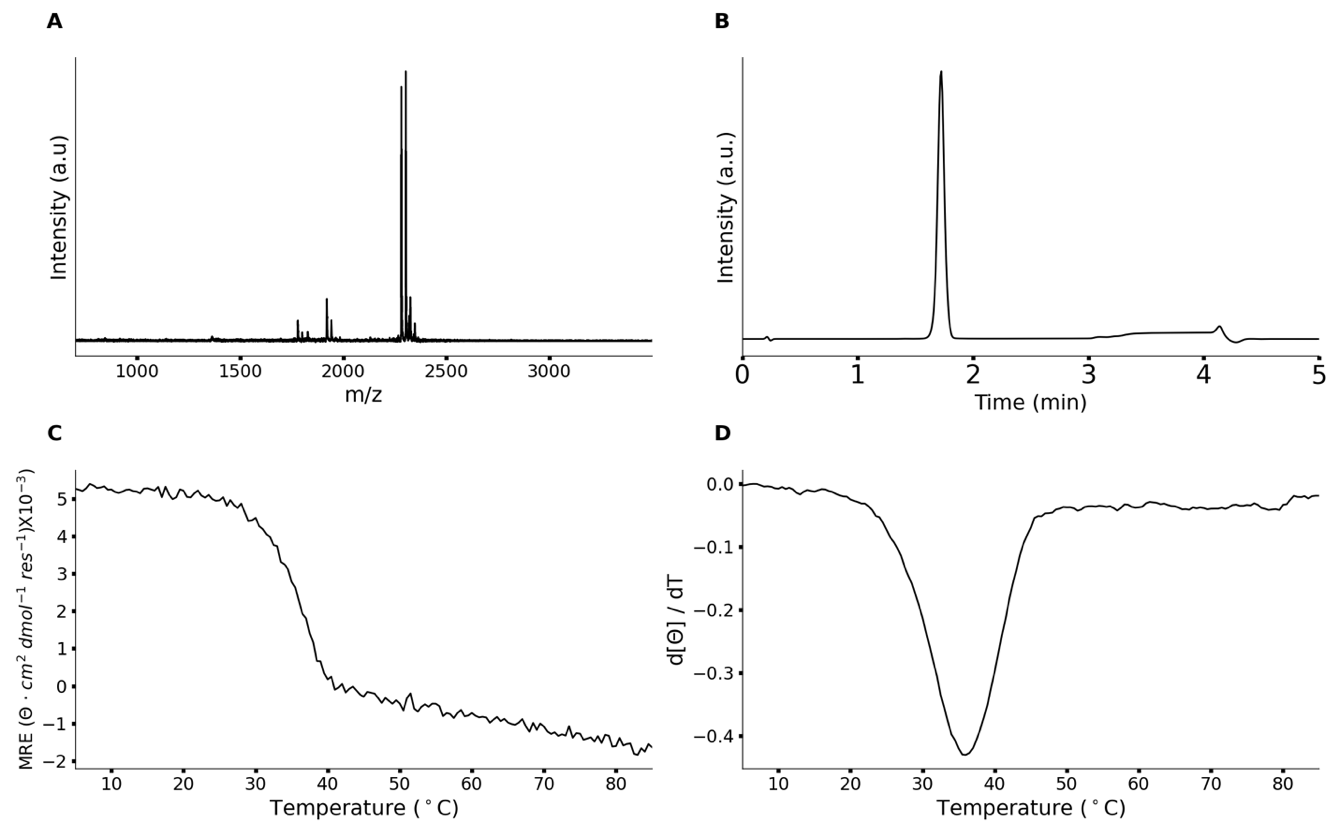


**Figure S11.** Characterization of peptide 495: (A) MALDI-ToF mass spectrum, (B) UPLC trace, CD C) Melting curve (D) First derivative of the melt


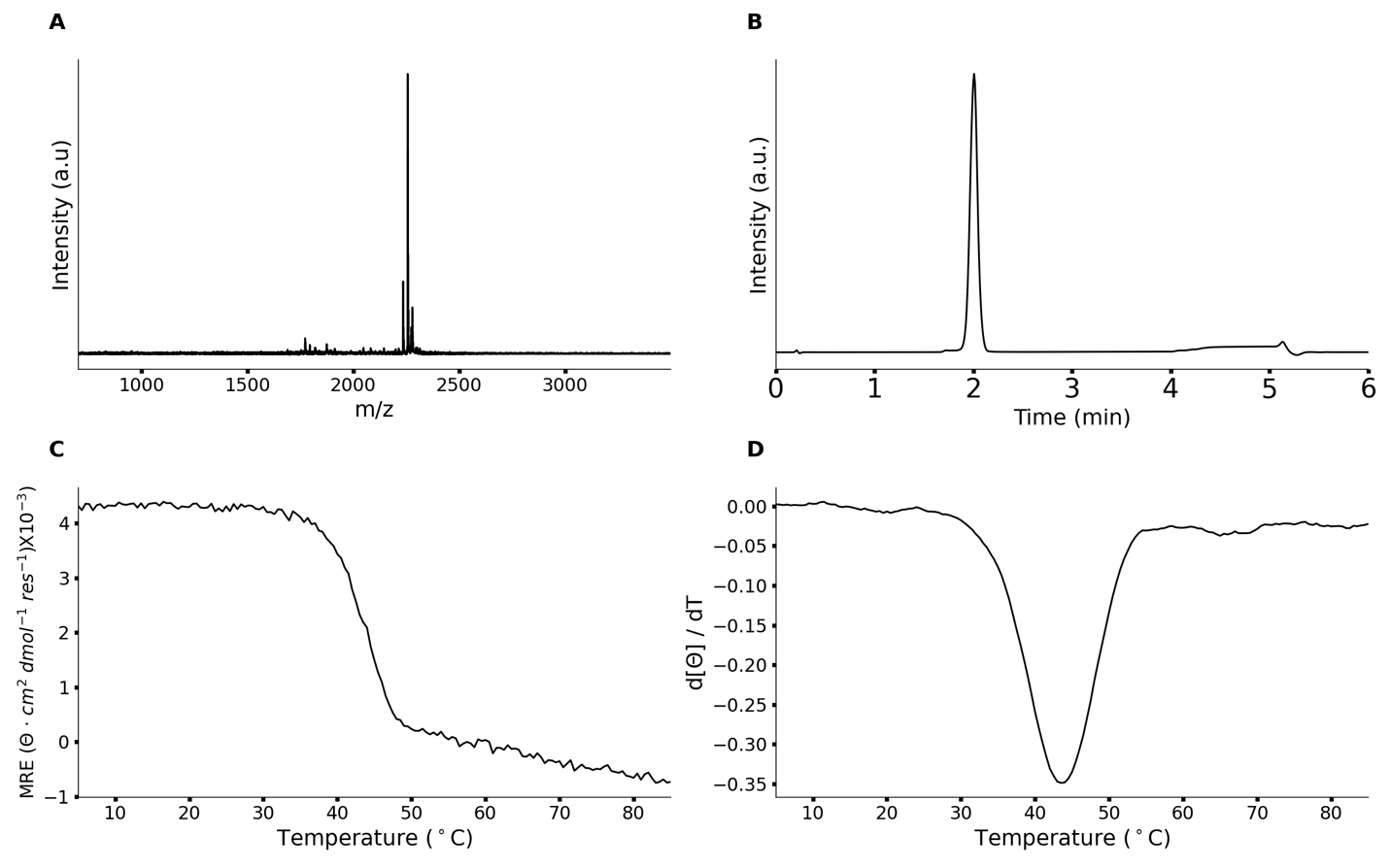


**Figure S12.** Characterization of peptide 496: (A) MALDI-ToF mass spectrum, (B) UPLC trace, CD C) Melting curve (D) First derivative of the melt


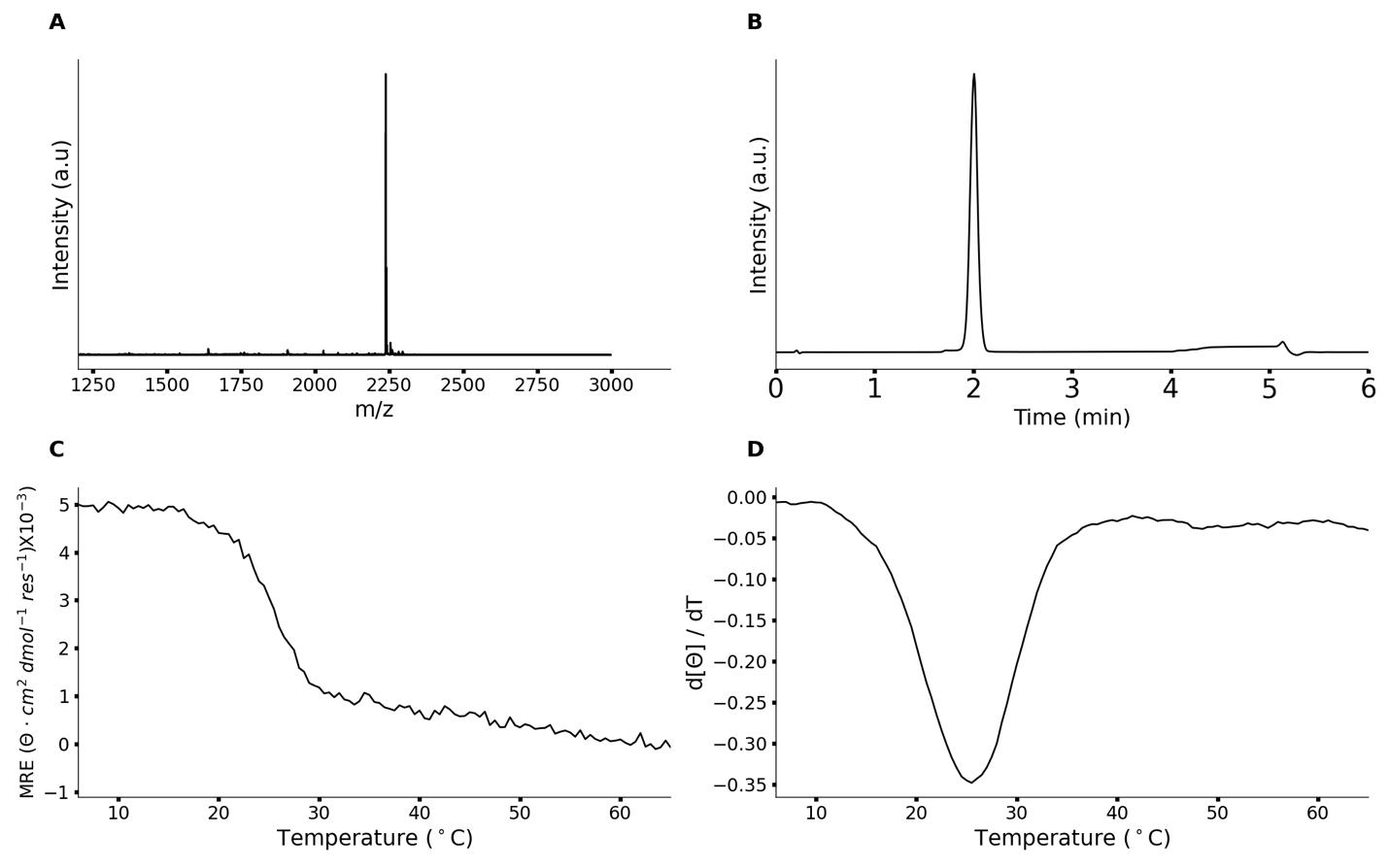


**Figure S13.** Characterization of peptide 542: (A) MALDI-ToF mass spectrum, (B) UPLC trace, CD C) Melting curve (D) First derivative of the melt


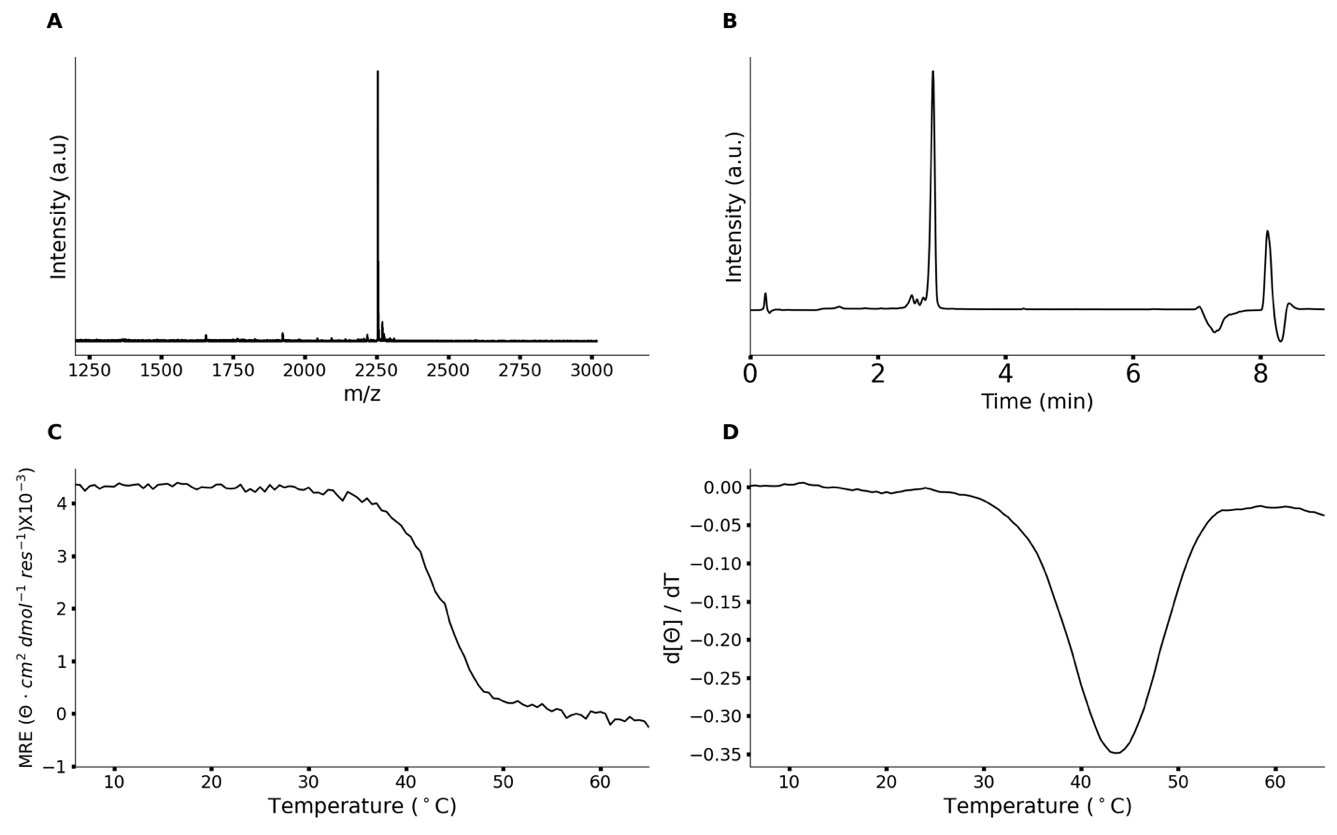


**Figure S14.** Characterization of peptide 543: (A) MALDI-ToF mass spectrum, (B) UPLC trace, CD C) Melting curve (D) First derivative of the melt


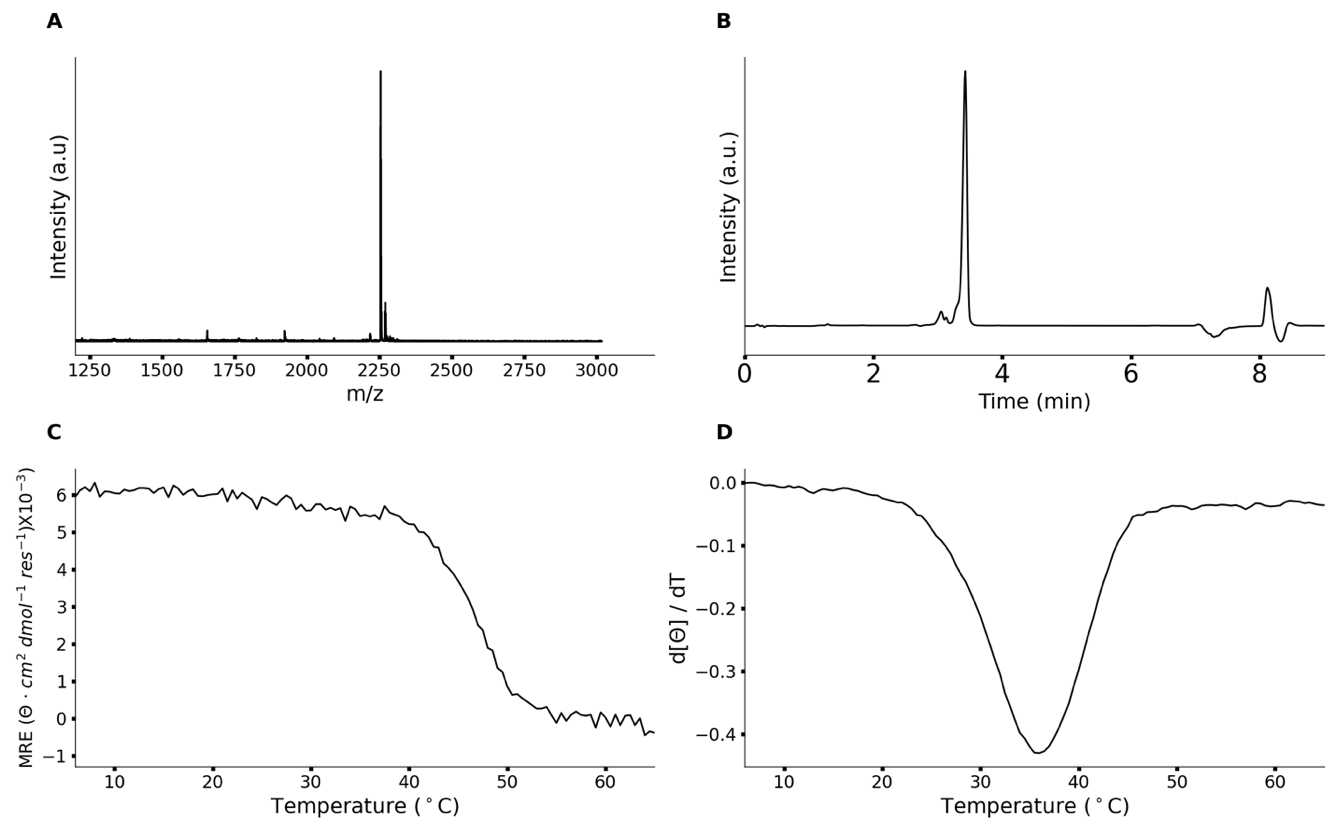


**Figure S15.** Characterization of peptide 544: (A) MALDI-ToF mass spectrum, (B) UPLC trace, CD C) Melting curve (D) First derivative of the melt


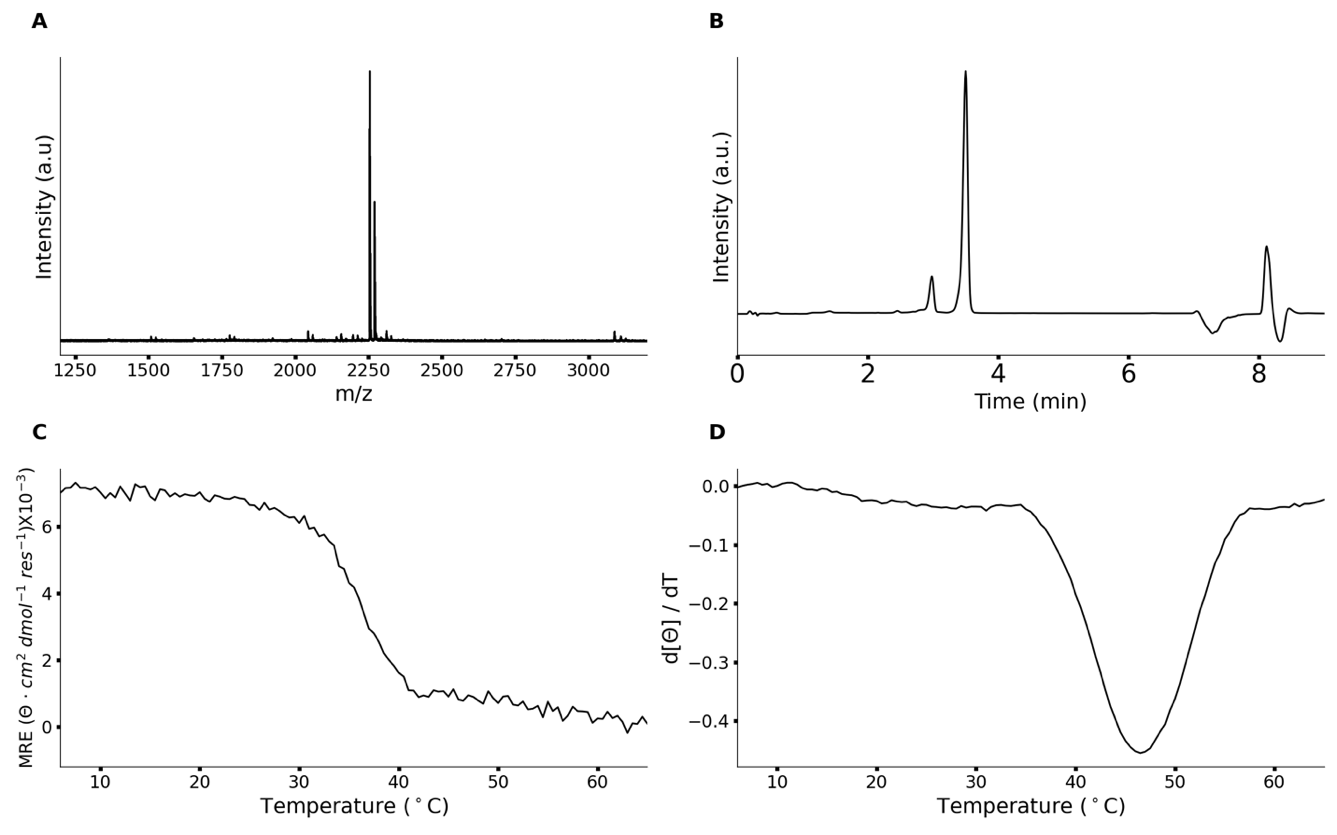


**Figure S16.** Characterization of peptide 545: (A) MALDI-ToF mass spectrum, (B) UPLC trace, CD C) Melting curve (D) First derivative of the melt


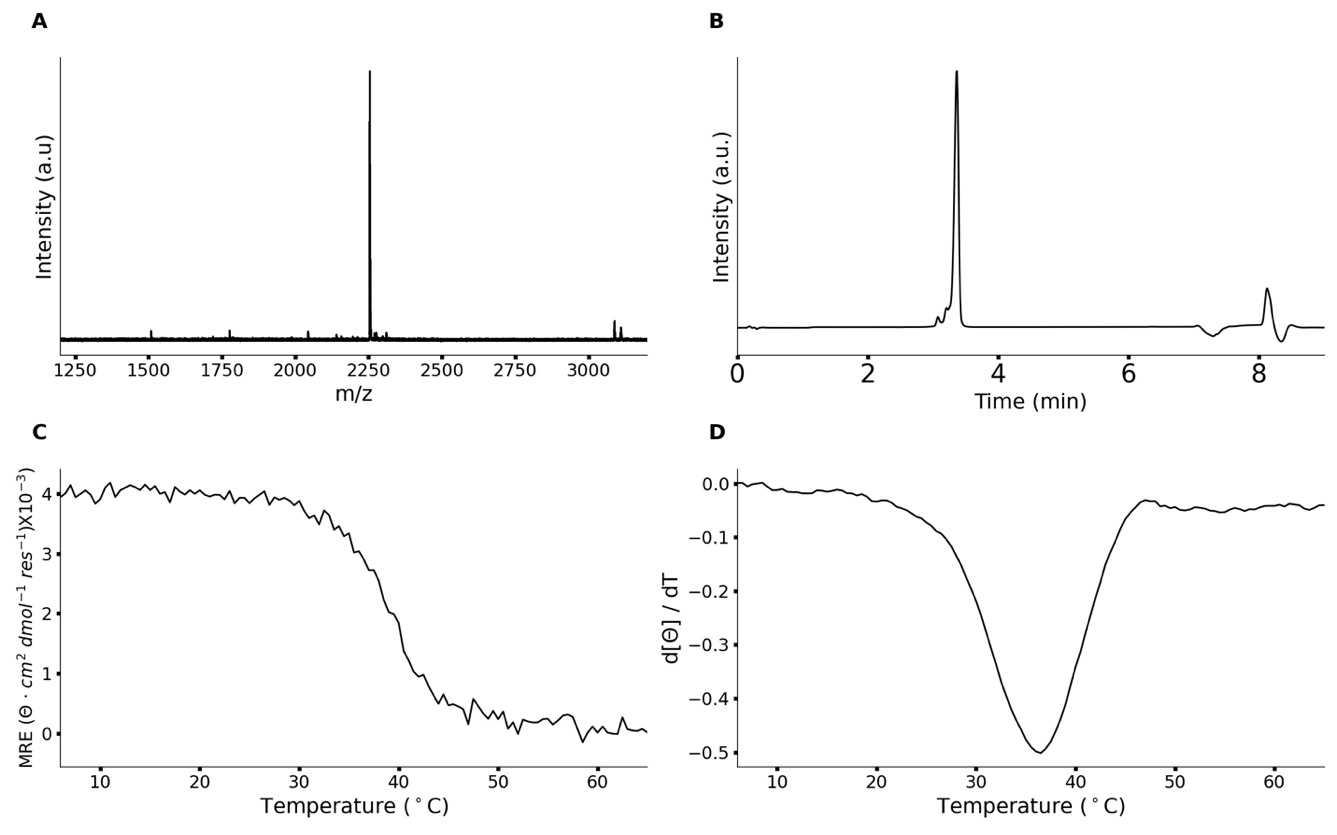


**Figure S17.** Characterization of peptide 546: (A) MALDI-ToF mass spectrum, (B) UPLC trace, CD C) Melting curve (D) First derivative of the melt


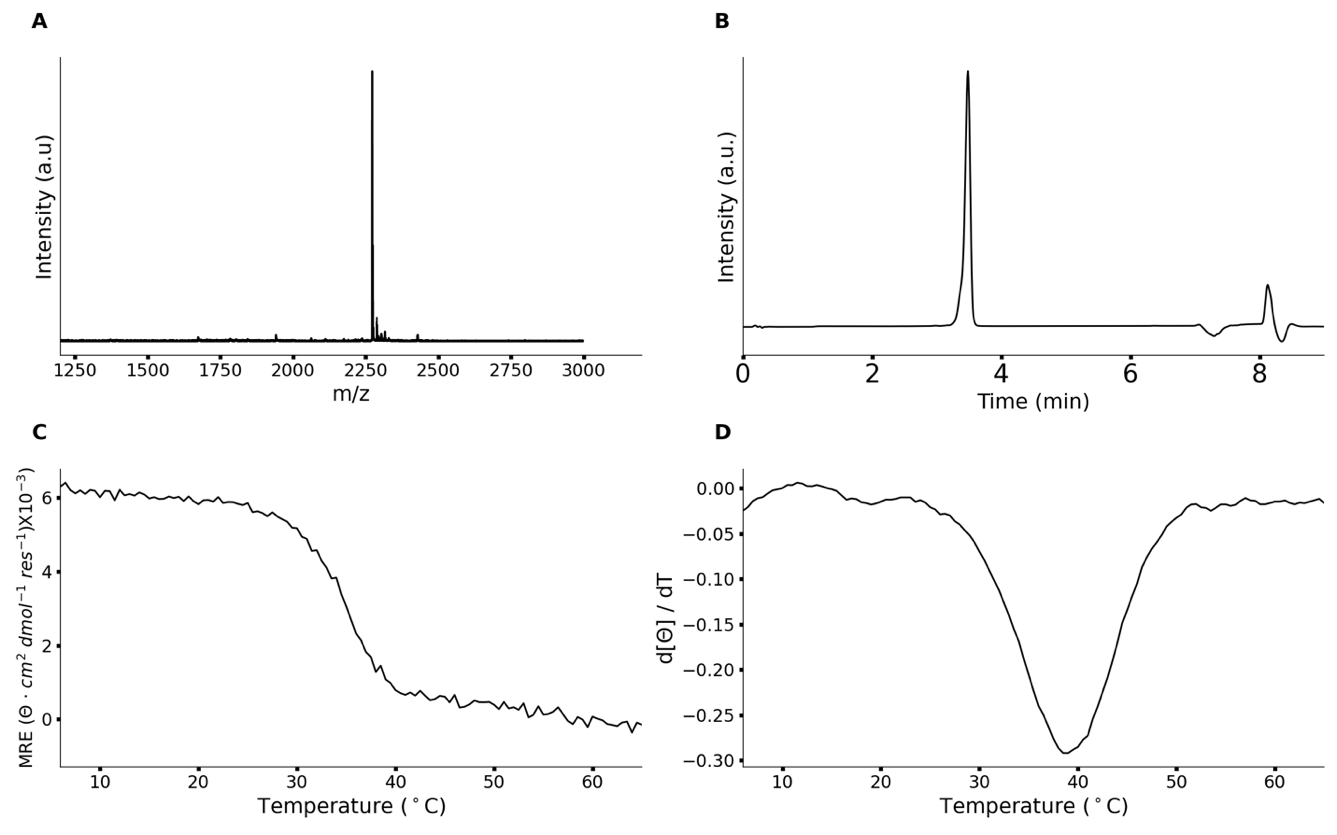


**Figure S18.** Characterization of peptide 547: (A) MALDI-ToF mass spectrum, (B) UPLC trace, CD C) Melting curve (D) First derivative of the melt


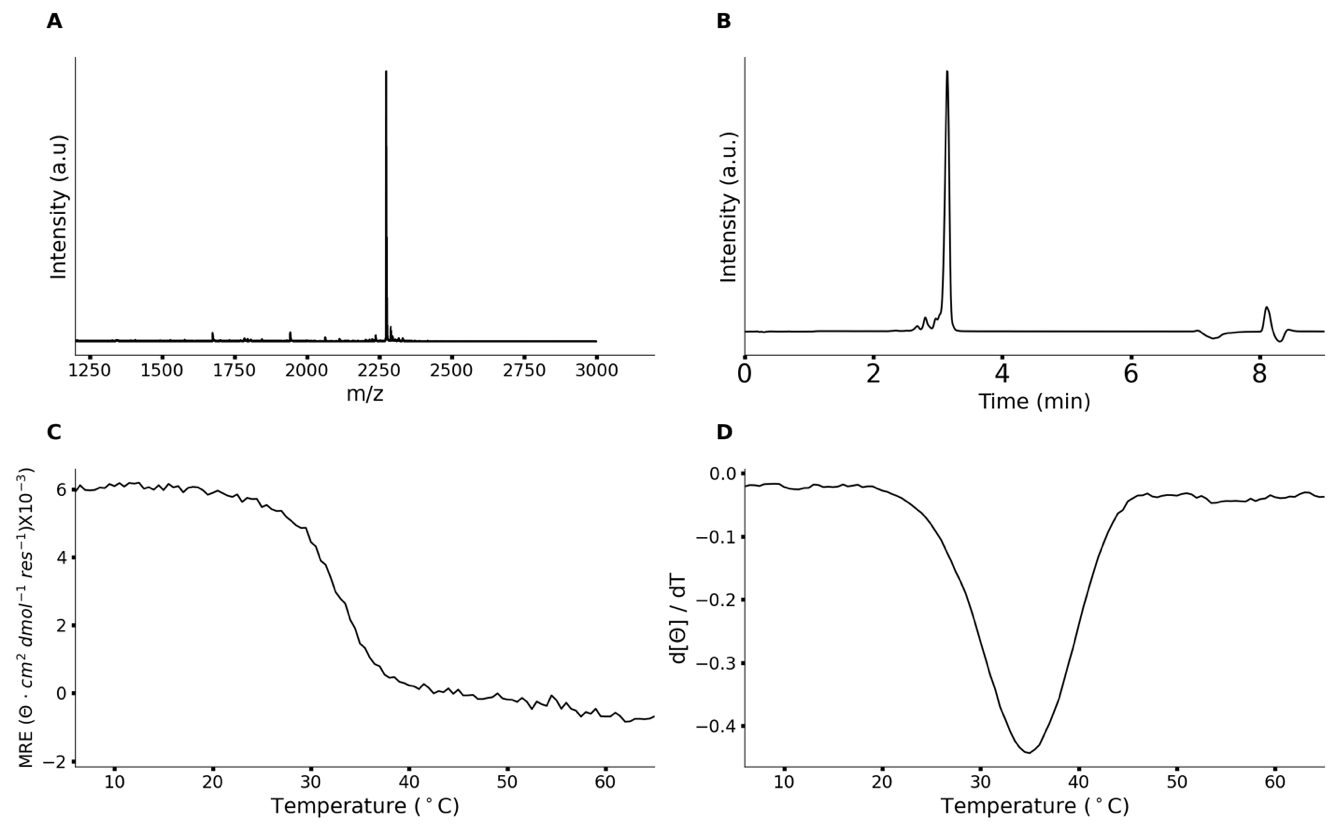


**Figure S19.** Characterization of peptide 548: (A) MALDI-ToF mass spectrum, (B) UPLC trace, CD C) Melting curve (D) First derivative of the melt


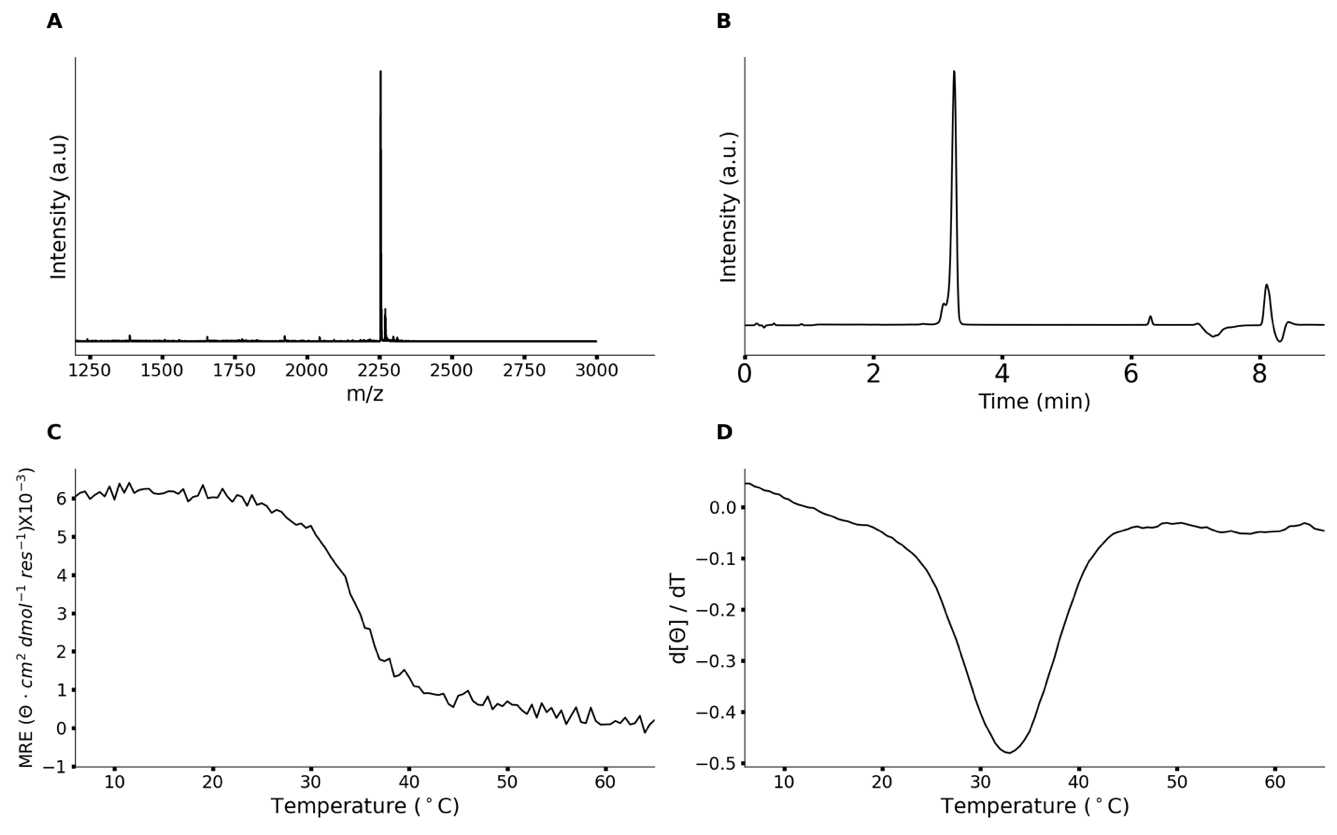


**Figure S20.** Characterization of peptide 549: (A) MALDI-ToF mass spectrum, (B) UPLC trace, CD C) Melting curve (D) First derivative of the melt


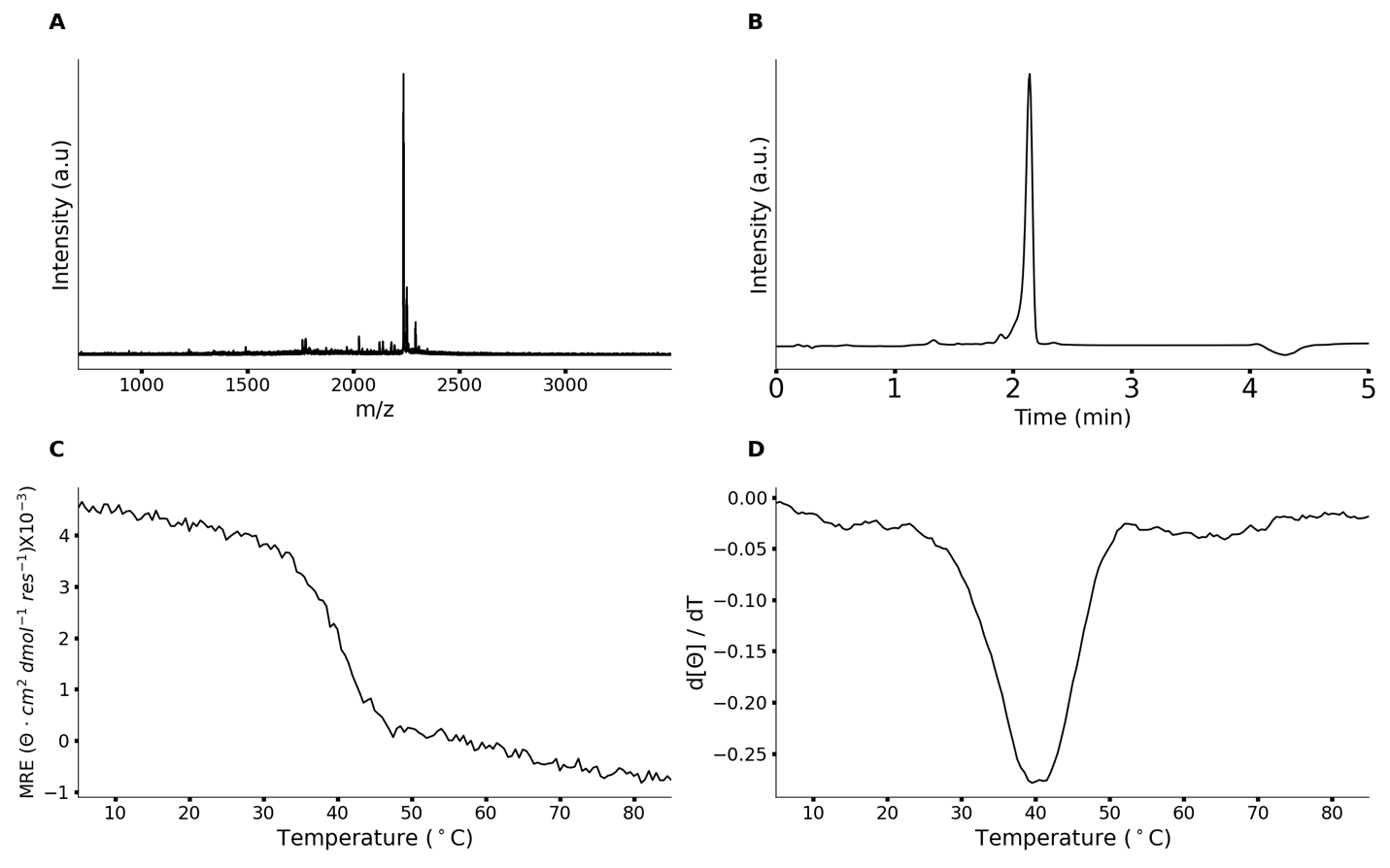


**Figure S21.** Characterization of peptide 551: (A) MALDI-ToF mass spectrum, (B) UPLC trace, CD C) Melting curve (D) First derivative of the melt


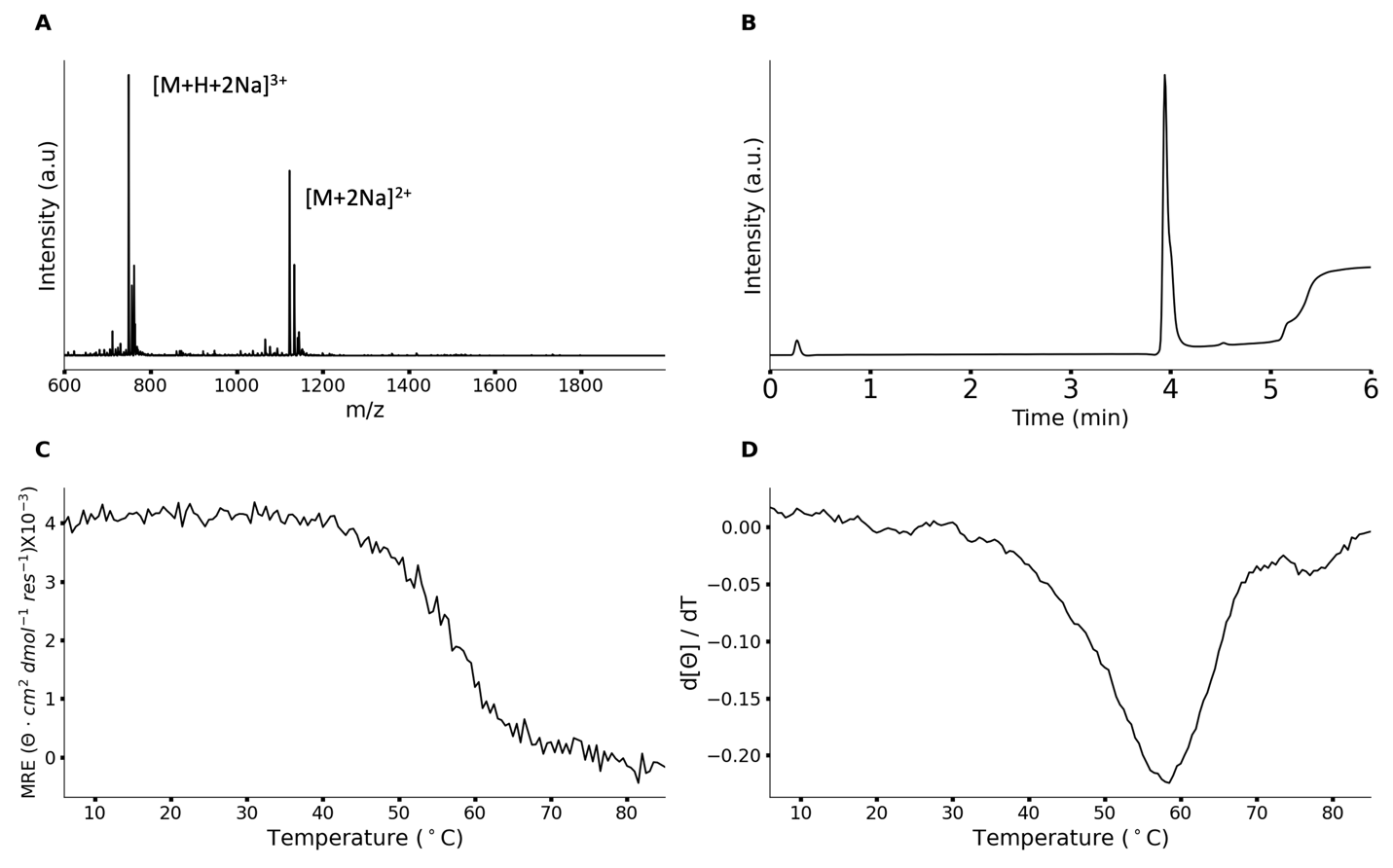


**Figure S22.** Characterization of peptide 572: (A) MALDI-ToF mass spectrum, (B) UPLC trace, CD C) Melting curve (D) First derivative of the melt

## **1.3. Incorporation of new parameters considering frameshifts and terminal amino acids**

The scoring of SCEPTTr 1.1 on the new peptides resulted in a relatively high average squared deviation (AvgSD) and low R^2^, suggesting that SCEPTTr 1.1 did not perform well on unseen data (Figure S23A). We refined the predictive power of SCEPTTr by incorporating new parameters that assess the effects of frameshifts and terminal amino acids. The refined version, SCEPTTr 1.2, was initially optimized using the same library as SCEPTTr 1.1 (Library 1.1) to maintain uniformity of training data when comparing two versions of the scoring function. This optimization resulted in AvgSD = 9.96 and R^2^ = 0.92 when evaluated on the training library, slightly worse compared to SCEPTTr 1.1 (Table S3). However, when evaluating the performance of both versions on new peptides, or unseen data, the scoring results demonstrated remarkable improvements of version 1.2 over its predecessor in terms of both accuracy and precision (Figure S23B, Table S3).

**Figure S23.** Scoring of new peptides by (a) SCEPTTr 1.1 (b) SCEPTTr 1.2 optimized with Library 1.1. Peptides featuring various frameshifts, capping conditions, and terminal residues are highlighted.

**Table S3.** Performance of different SCEPTTr versions on Library 1.1, Library 1.2, and new peptides

|  | Library 1.1 (AvgSD/R^2^) | Library 1.2 (AvgSD/R^2^) | New peptides (AvgSD/R^2^) |
| --- | --- | --- | --- |
| SCEPTTr 1.1 | 5.62/0.95 | 17.32/0.84 | 65.71/0.44 |
| SCEPTTr 1.2 optimized with Library 1.1 | 9.96/0.92 | 16.40/0.85 | 42.06/0.64 |
| SCEPTTr 1.2 optimized with Library 1.2 | 9.79/0.92 | 9.73/0.90 | 13.37/0.85 |

## **1.4. SCEPTTr 1.2 optimization by a genetic algorithm**

To broaden the predictive scope of SCEPTTr 1.2, the scoring function was subjected to optimization with the expanded library (Library 1.2). A genetic algorithm is an effective optimization method to ensure that all parameters that influence the net stability of a triple helix are considered in each round of optimization. Starting with experimentally deconvoluted parameter sets, each value of parameter has 20% chance of being adjusted by a random step size within (-0.2, 0.2) to create an initial population of 100 sets of parameters. These parameter sets were used to score the peptide library, and the 10 sets with the lowest AvgSD were selected as parents for the next generation. *C*(10, 2) combinations of parent sets were crossover, followed by mutation to produce new population. Parent sets were also retained in the new population. The new population of parameters set were then used to score peptide library, and AvgSD was ranked to choose best parents. This iterative process continued until no further improvement in AvgSD was observed.

SCEPTTr 1.2 re-optimization resulted in a fit of R^2^ = 0.90 with AvgSD = 9.73 (Figure S24A; Table S3). When scoring new peptides, significant improvements were observed in the performance of fully optimized SCEPTTr 1.2 compared to both SCEPTTr 1.1 and SCEPTTr 1.2 before full optimization (Figure S24C; Table S3). However, the parameter adjustments also resulted in a slight degradation in the estimation of peptides that were already present in the original SCEPTTr 1.1 library (Figure S24B; Table S3). This implies that SCEPTTr1.1 was overfitted to a certain extent. At the same time, during the optimization process, the scoring parameters were adjusted to better capture the characteristics of as many data points as possible from the expanded dataset. Consequently, the observed degradation could also be attributed to the fact that data adjustments made to accommodate new data can impact the reliability of predictions for existing peptides.


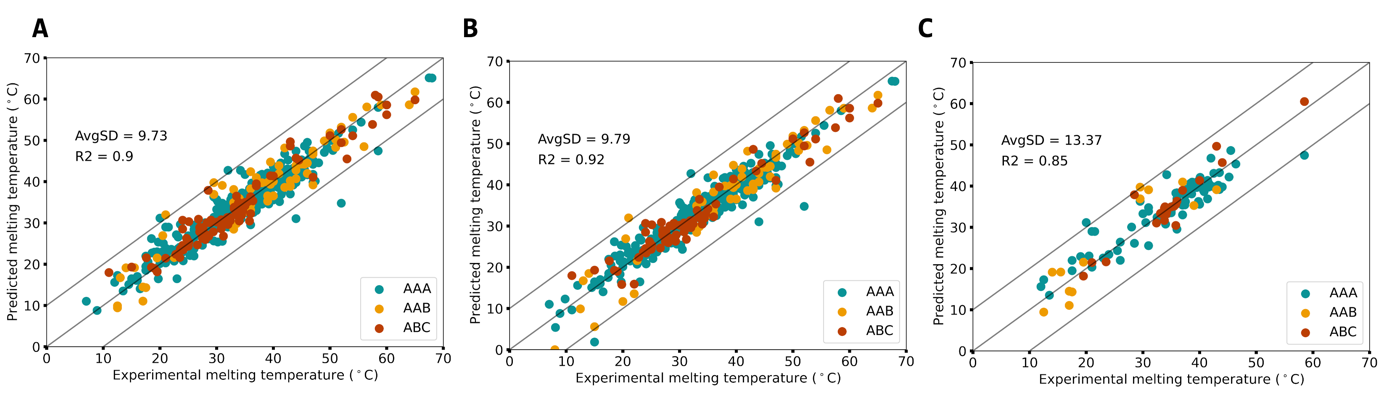


**Figure S24.** Performance of SCEPTTr 1.2 optimized with Library 1.2 on scoring (a) Library 1.2 (b) Library 1.1 and (c) new peptides only.

# **2. Algorithms preceding GRACE**

In 2005, Brodsky et al. introduced the Collagen Stability Calculator, the first physics-based model to relate CMP sequences to melting temperatures (Tm). ^[14]^ While establishing the foundation for CMP scoring, this model was limited to homotrimer assessment and only considered a selected set of stabilizing interactions. Recently, machine learning approaches have emerged to show advantages in collagen predicting domain. The Buehler group successfully developed two models, ColGen (2022) ^[21]^ and CollagenTransformer (2022) ^[22]^, leveraging deep learning and natural language processing (NLP), respectively, to predict Tm based solely on primary sequence data. These tools demonstrated notable predictive capabilities without relying on structural or physicochemical cues. Additionally, ColGen-GA, a tool developed by the same group in 2022, integrated a genetic algorithm for sequence optimization with the stability assessment framework of ColGen to generate de novo homotrimeric CMP sequences. ^[23]^ However, the utilities of all mentioned methods remained constrained to homotrimeric CMPs, thereby excluding the more prevalent heterotrimeric assemblies found in most natural collagens.

Early advancements in the computational design of heterotrimeric CMPs were achieved by Nanda and co-workers. In 2010, the Nanda group published first attempt of utilizing computational approach for de novo design of an ABC-type heterotrimer with arginine-glutamate interactions.^[24]^ This approach used Monte Carlo Simulated Annealing (MCSA) to concurrently optimize amino acid sequence for stabilization of target stability and specificity over competing states. Triple helical stability was computed using an energy function, and calculated energetic values were then correlated with experimental Tm via a line of best fit. This calibration line allowed the translation of computed energetic values into predicted Tm. Although the method was successful in identifying sequence candidates, the heterotrimer designed using this strategy did not achieve the target ABC-design. In fact, the most stabilizing composition experimentally identified was a B₂C helix. In a 2011 follow-up, the protocol was modified to focus on different charged amino acid pairs such as, lysine and aspartate pair.^[25]^ The revised protocol used MCSA for sequence sorting and the energy-function framework for stability assessment. This work successfully produced an ABC-type triple helix that demonstrated enhanced thermal stability (Tm = 29 °C) and specificity (ΔTm = 21 °C) compared to the 2010 design. In 2012, the Nanda group published a new iteration of scoring function that again focused on arginine-glutamate pair and could directly estimate a value for melting temperature. ^[29]^ The ABC-type heterotrimer design reported in that study achieved a thermal stability of 50 °C and specificity of 7 °C. In a 2018 iteration, Nanda and colleagues introduced further refinements of their scoring function by incorporating data from molecular dynamics (MD) simulations. ^[27]^ The flexibility of side chains was now estimated from MD trajectories, allowing the scoring function to incorporate weighting of interaction contributions. Concurrently, Fallas from our group introduced a genetic algorithm-based protocol utilizing lysine-aspartate charge pairs in 2012. ^[26]^ Though lack of the ability to assign a predicted values for melting temperatures, this approach successfully guided the sequence design of a highly stable and specific ABC-type triple helix. The designed heterotrimer had experimental stability of 58 °C and specificity of 15 °C. Despite promising progress, all algorithms discussed in this paragraph were limited in scope. Their capabilities of stability assessment and sequence optimization were only applicable to charge amino acid substitutions and interactions.

A more comprehensive and generalizable paradigm for CMP estimation was introduced by Walker from our group in 2021 as SCEPTTr. ^[30]^ This scoring function incorporated multiple factors influencing stability of a triple helix including peptide length, terminal capping, amino acid propensities, pairwise interactions, and helix register alignment. It is applicable to all 20 canonical amino acids and hydroxyproline, can evaluate homotrimers, A_2_B and ABC heterotrimer, and accounts for all helix compositions and registers, including non-canonical ones. SCEPTTr achieved a high correlation (R² = 0.95) between predicted and experimental Tm values across a dataset of 431 known sequences. SCEPTTr was shown to successfully guide the design of the first chemically diverse ABC- heterotrimer having Tm of 39.5 and specificity of 16 °C. The next iteration, SCEPTTr 1.1, incorporated novel charge-free amide-π interactions, expanding the toolbox for controlling triple-helical assemblies. ^[19]^ SCEPTTr 1.2, reported in our current work, further expands the toolbox by adding parameters that assesses cation-π interactions, interactions between reversed charged pairs, and the effect of terminal residues and frameshifts, while extending the training library to improve the predictive scope. Additionally SCEPTTr 1.2 reduces over-fitting problems observed in previous versions of SCEPTTr.

Although SCEPTTr has shown to significantly streamline the design process, defining initial sequence inputs remains a complex task. This includes evaluating competing species, amino acid propensities at specific positions, pairwise interactions, and other factors that influence CMP folding. The process becomes even more challenging when sequences must incorporate biologically relevant motifs, such as protein binding sites, which further constrain utility space for sequence design. To address this, we introduced GRACE, an integration of a new-generation genetic algorithm with SCEPTTr 1.2, as a robust method to discover chemically diverse, highly specific heterotrimeric triple helices. Notably, GRACE allows users to input biologically important sequences, such as binding motifs, and ensures these features are maintained throughout the optimization process. This enables GRACE to support not only de novo design but also the functional design of heterotrimeric CMPs.

**Table S4.** Summary of computational algorithms tailored to collagen prediction and design

| Model | Year | Sequence optimization method | Stability assessment method | Considering hetetrotrimer? | Amino acid consideration |
| --- | --- | --- | --- | --- | --- |
| Collagen Stability Calculator,  Brodsky group ^[14]^ | 2005 | N/a | Melting temperature | No | All amino acids |
| Nanda2010,  Nanda group ^[24]^ | 2010 | Monte Carlo Simulated Annealing | Energy calculation | Yes | Limited to arginine and glutamate |
| Nanda2011,  Nanda group ^[25]^ | 2011 | Monte Carlo Simulated Annealing | Energy calculation | Yes | Limited to lysine and aspartate |
| Nanda2013,  Nanda group ^[29]^ | 2013 | N/a | Melting temperature | Yes | Limited to arginine and glutamate |
| Nanda2018,  Nanda group ^[27]^ | 2018 | N/a | Melting temperature | Yes | Limited to lysine and aspartate |
| ColGen,  Buehler group ^[21]^ | 2022 | N/a | Melting temperature | No | All amino acids |
| CollagenTransformer, Buehler group ^[22]^ | 2022 | N/a | Melting temperature | No | All amino acids |
| ColGen-GA,  Buehler group ^[23]^ | 2022 | Genetic Algorithm | Melting temperature | No | All amino acids |
| Hargertink2012, Hartgerink group ^[26]^ | 2012 | Genetic Algorithm | N/a | Yes | Limited to lysine and aspartate |
| SCEPTTr 1.0,  Hartgerink group ^[30]^ | 2021 | N/a | Melting temperature | Yes | All amino acids |
| SCEPTTr 1.1,  Hartgerink group^[19]^ | 2021 | N/a | Melting temperature | Yes | All amino acids |
| SCEPTTr 1.2,  Hartgerink group | 2025 | N/a | Melting temperature | Yes | All amino acids |
| GRACE,  Hartgerink group | 2025 | Genetic Algorithm | Melting temperature | Yes | All amino acids |

# **3. GRACE performance at varying target conditions, mutation rate, crossover rate, and initial population size**

## **3.1. Comparison of GRACE performance at varying target melting temperatures and specificity**

**Table S5.** Comparison of GRACE runtime under different target conditions without any restriction in amino acid substitution, initial population of 500, mutation rate of 0.2, and crossover rate of 0.6

| Input | | Run time (sec) | Generation | Output | | |
| --- | --- | --- | --- | --- | --- | --- |
| Target Tm (°C) | Target ΔTm (°C) |  |  | Sequences | Predicted Tm (°C) | Predicted ΔTm (°C) |
| 37 | 25 | 420.0 | 1247 | A: RGNPGPRG**E**OGPOGSOG**D**RGOMG**FK**GP**Q**GR  B:  **K**G**QQ**GPOG**D**SGP**K**GPOGPPG**D**OG**K**PG**DK**G**F**  C:   IG**D**OGPSG**E**OGP**K**G**D**OGORGLOGPOG**DD**G**D** | 53.6 | 26.4 |
| 47 | 25 | 586.8 | 1753 | A: GPSGOSG**F**AGPRG**K**OGPOGPPG**FQ**GOOG**WQ**  B:  G**E**AG**EK**GO**K**GN**K**G**WQ**GPOGP**K**GPRG**FQ**GPM  C:   GO**Q**GNOG**D**PG**DD**G**D**RG**FQ**G**D**OG**E**RG**D**OG**F**R | 47.8 | 42.7 |
| 57 | 25 | 7648.9 | 19595 | A: GP**K**G**EK**GP**K**GPRG**F**OGPOGPSGPOGPOG**WK**  B:  G**DQ**G**D**OG**D**OG**E**OG**W**OGPOG**E**PGOSGPOG**D**O  C:   GISG**F**PGP**Q**GPOGPOGPOGPOG**E**RGPPGPO | 60.6 | 26.4 |
| 47 | 10 | 320.9 | 929 | A: RGIOG**D**OGOPGAOG**F**OG**F**SGPOG**Q**OGP**K**G**F**  B:  **K**G**WK**GOOGPSGOMGPRG**E**MGPPG**E**AG**D**PG**D**  C:   **Q**G**EQ**G**D**OG**EQ**GPMG**Q**OG**D**RGPPGR**Q**GNOG**D** | 47.4 | 22.7 |
| 47 | 15 | 333.5 | 967 | A: **K**GNPG**Q**OG**QQ**G**F**MGPOGPRGPPGPOG**D**RG**W**  B:  IG**Y**RGPOGOOG**F**OGPOGPPG**Q**AGPOGPRG**W**  C:   MG**Q**RGP**Q**GOMGPOGPOGPOG**EK**GN**K**GPOG**W** | 56.3 | 15.8 |
| 47 | 35 | 656.5 | 1890 | A: G**EQ**G**D**MG**D**OG**F**OG**DQ**G**D**OG**D**OGPMGP**Q**G**W**S  B:  GASG**F**PGORGP**Q**GPOG**FD**GOPGPRGAOG**E**O  C:   G**EK**GP**Q**GOOGO**K**G**FK**G**K**OGTOGPRG**W**OGOP | 54.1 | 35.1 |

**
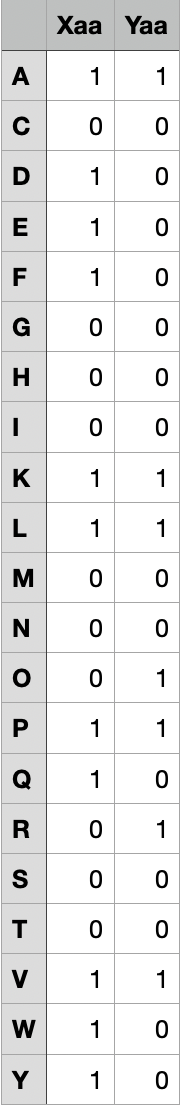
**

**Figure S25.** Tabular presentation of AminoAcids.csv file. The values in each cell can be either ‘1’ or ‘0’. These values can be turn to ‘0’ if the user wants to exclude certain amino acids at Xaa or Yaa positions. These values were used in the triple helices incorporating biological motifs, AAB-FOGER and ABC-FOGER. Triple helices generated without biological motifs (ABC-1, ABC-2) had all values set to “1”.

**Table S6.** Comparison of GRACE runtime under different target conditions with limited amino acid substitution (as in figure S24), initial population of 500, mutation rate of 0.2, and crossover rate of 0.6

| Input | | Run time (sec) | Generation | Output | | |
| --- | --- | --- | --- | --- | --- | --- |
| Target Tm (°C) | Target ΔTm (°C) |  |  | Sequences | Predicted Tm (°C) | Predicted ΔTm (°C) |
| 37 | 25 | 51.1 | 195 | A: GPRGPOG**Q**OG**K**RG**E**OG**DK**G**E**OG**QK**GAOG**F**A  B:  G**EK**G**W**VGPOGPPG**Q**OG**D**PG**D**RG**K**OG**D**OG**KK**  C:   G**D**RG**D**RGA**K**GPRGPOGPRG**E**RG**F**RGPOG**D**O | 46.0 | 25.3 |
| 47 | 25 | 56.5 | 216 | A: RGPRGPOG**WK**GPVGAOGP**K**G**Q**OGAOGPRG**Y**  B:  VG**YK**G**F**OG**D**RG**D**RGPOG**K**OG**DK**GPPG**EK**G**Y**  C:   OG**D**RG**D**PGPOG**Q**RG**D**RGLOG**D**OG**Y**RGPOG**Y** | 47.0 | 27.5 |
| 57 | 25 | 101.3 | 394 | A: GP**K**G**D**OG**DK**GPOG**Q**OG**D**OG**DK**GP**K**G**Q**OG**WK**  B:  G**DK**G**D**OG**DK**G**D**OGPOGPOG**K**RG**D**PG**D**RG**K**V  C:   G**DK**G**D**OG**D**RG**DK**GP**K**GPOGPRGPRGPOG**W**R | 64.5 | 25.4 |
| 47 | 10 | 34.2 | 151 | A: RG**D**OG**E**OG**F**RGPRGPPGP**K**G**Q**RGPOGVRGP  B:  OG**F**OGP**K**G**Q**RGP**K**GPOG**D**OG**E**OG**F**OGP**K**G**W**  C:   **K**GPRG**K**OG**E**RG**K**RG**D**RGPPGLAGPPG**D**OG**Y** | 47.4 | 28.2 |
| 47 | 15 | 58.5 | 231 | A: GA**K**GP**K**G**D**OG**D**OG**Q**OG**D**OG**E**OG**K**OGPOG**W**R  B:  G**K**PG**E**OG**DK**GPOGPRG**D**RGPOGP**K**GPOG**E**R  C:   GP**K**GP**K**G**D**RG**D**PGP**K**G**E**RGAOGPRG**D**PG**E**P | 51.5 | 16 |
| 47 | 35 | 11013.3 | 43493 | A: G**FK**G**K**OG**E**OG**F**OG**D**RG**D**OG**D**RG**D**OG**K**OG**D**R  B: G**DK**G**DK**GPRGLRGPOG**F**PGPOG**F**PGPRG**E**P  C: G**DK**G**D**RG**E**OG**QK**G**FK**GP**K**GPOGP**K**GPOG**W**O | 55.5 | 35.4 |

## **3.2. Comparison of GRACE performance at varying mutation rates**

**Table S7.** Comparison of GRACE average runtime at varying mutation rates. For selected amino acids, initial population size of 500, crossover rate of 0.0, target Tm of 47.0 °C, and target specificity of 25 °C, mutation rate of 0.2 resulted in the shortest average runtime across three trials.

| Mutation Rate | Runtime (sec) | | | |
| --- | --- | --- | --- | --- |
|  | Trial 1 | Trial 2 | Trial 3 | Average |
| 0.01 | 26.2 | 94.7 | 75.4 | 65.4 |
| 0.1 | 40.8 | 45.1 | 42.8 | 42.9 |
| **0.2** | **19.6** | **40.1** | **35.6** | **31.8** |
| 0.4 | 121.8 | 143.4 | 106.8 | 124 |
| 0.6 | 947.4 | 606.1 | 469.1 | 674.2 |
| 0.8 | 400.8 | 793.5 | 2484.7 | 1226.3 |
| 1.0 | 86337 | 2245.1 | 64457 | 51013.0 |

**Table S8.** Comparison of GRACE average generation at varying mutation rates. For selected amino acids, initial population size of 500, crossover rate of 0.0, target Tm of 47.0 °C, and target specificity of 25 °C, mutation rate of 0.2 resulted in the lowest generations across three trials.

| Mutation Rate | Generation | | | |
| --- | --- | --- | --- | --- |
|  | Trial 1 | Trial 2 | Trial 3 | Average |
| 0.01 | 2040 | 7507 | 4925 | 4824 |
| 0.1 | 732 | 752 | 618 | 701 |
| **0.2** | **211** | **365** | **395** | **324** |
| 0.4 | 1026 | 1202 | 731 | 986 |
| 0.6 | 7682 | 4897 | 3336 | 5305 |
| 0.8 | 3101 | 5968 | 19254 | 9441 |
| 1.0 | 16668 | 17551 | 19480 | 17900 |

**Table S9.** Runtime at varying mutation rates - Trial 1

| Mutation rate | Run time (sec) | Generation | Output | | |
| --- | --- | --- | --- | --- | --- |
|  |  |  | Sequences | Predicted Tm (°C) | Predicted ΔTm (°C) |
| 0.01 | 26.2 | 2040 | A: G**WK**GP**K**G**Q**OG**Q**RG**D**RG**Y**OGPOGPOG**F**RGPO  B:  G**DK**G**D**VG**D**RG**E**OG**Q**OG**F**OGP**K**GAOG**Q**OG**FK**  C:   G**D**RG**D**RGP**K**G**FK**GPPGPOGPRG**D**OGPOG**D**A | 47.1 | 25.6 |
| 0.1 | 40.8 | 732 | A: G**FK**GPOG**K**OG**D**RG**D**OGPOG**Q**PG**FK**GAOGLO  B:  G**D**RG**D**RGPOGPOG**F**RGPOGP**K**GPRG**D**PGLA  C:   G**Q**RG**QK**G**FK**GPOGPRG**D**RGPOG**E**PGPOG**E**P | 47.6 | 26.8 |
| 0.2 | 19.6 | 211 | A: GP**K**GPOGPOG**K**RG**D**RGPAGPRG**F**RG**D**RG**D**P  B:  G**D**PG**D**OGPOG**Q**OG**Q**RGP**K**GVOG**Q**OG**EK**G**Y**L  C:   GPPGPOGP**K**GPOG**E**PG**D**RG**D**OGL**K**G**K**OG**D**R | 47.5 | 25.3 |
| 0.4 | 121.8 | 1026 | A: G**DK**GPVG**K**OG**EK**GPRGPOGPAG**FK**GPRG**F**P  B:  G**D**RG**D**OGPOG**D**OG**EK**G**FK**G**K**RGPPG**D**RG**W**P  C:   G**E**PGP**K**GPOGPOG**D**PG**D**RG**D**OG**W**RGPOG**W**R | 48.3 | 28.6 |
| 0.6 | 947.4 | 7682 | A: G**DK**GPOGV**K**GPOG**F**PG**E**OG**F**OGPRGPOG**Y**R  B:  G**D**PG**D**RG**D**AG**D**RGPRGPOGPPGPOG**W**OG**EK**  C:   GPAGPOG**W**RGVOGPRG**F**OGPOGPRGPRG**D**O | 47.8 | 25.6 |
| 0.8 | 400.8 | 3101 | A: G**FK**G**E**OGVVG**D**OGPOGPOGP**K**GPOGPAG**Y**O  B:  G**DK**G**DK**GARGL**K**GP**K**GPOG**D**PG**E**OGP**K**GVV  C:   G**D**AG**EK**G**Y**OG**Q**OG**D**OG**D**PGPOGPRG**D**OG**D**R | 47.0 | 25.1 |
| 1.0 | 86337.0 | 16668 | A: GP**K**GPOG**W**OG**E**OG**F**RG**D**OG**D**OGAOGPRG**K**P  B:  G**K**PG**DK**GA**K**GPOGPRG**F**OGPOGPRGPOG**W**R  C:   GPRG**D**PG**D**RG**E**OGP**K**G**K**OGPOGPPG**F**PG**E**P | 50.1 | 25.8 |

**Table S10.** Runtime at varying mutation rates – Trial 2

| Mutation Rate | Run time (sec) | Generation | Output | | |
| --- | --- | --- | --- | --- | --- |
|  |  |  | Sequences | Predicted Tm (°C) | Predicted ΔTm (°C) |
| 0.01 | 94.7 | 7507 | A: GL**K**GPRGPOG**FK**GPOGP**K**GP**K**GPOGP**K**G**Y**R  B:  G**DK**G**E**OG**FK**G**DK**G**D**RG**KK**G**D**OG**D**OGPRG**E**O  C:   G**D**PG**D**RG**D**OG**D**PG**E**OG**D**OG**D**OGPRGPOG**W**R | 59.8 | 26.0 |
| 0.1 | 45.1 | 752 | A: G**F**PGPPG**D**RG**D**OGPOG**D**RG**D**RG**D**RGPOG**W**P  B:  G**QK**GPOG**E**RG**F**OGPOG**Q**OG**Q**RG**Q**OG**F**OGPR  C:   G**DK**G**DK**GPOG**F**OGP**K**GP**K**GPOG**W**RGPOG**E**P | 53.0 | 26.2 |
| 0.2 | 40.1 | 365 | A: GPVG**DK**G**D**OGLOG**K**RG**D**OGPRGPOG**QK**G**W**R  B:  G**F**RG**D**RG**D**PGPAGPRG**F**OGPOG**F**OG**K**RG**E**P  C:   G**EK**G**Q**OG**Q**OG**KK**GVOG**F**OGPRGPRGPOG**W**A | 47.6 | 26.3 |
| 0.4 | 143.4 | 1202 | A: GPPGPRGPOG**F**RG**D**OG**D**AG**D**OG**D**PG**EK**G**W**R  B:  GP**K**GP**K**G**QK**G**E**OG**W**PGPOGVRGPOG**KK**G**Y**O  C:   G**D**PG**D**RG**D**OG**DK**GP**K**GP**K**GP**K**G**F**RG**D**PG**W**R | 47.2 | 36.9 |
| 0.6 | 606.1 | 4897 | A: G**D**AGPOG**WK**GPOGPOG**Q**RG**D**RG**D**OGP**K**G**E**O  B:  G**E**OGPOG**D**RG**D**OGP**K**GPOG**Q**AG**F**OG**D**OG**YK**  C:   GPRGPOGPOG**F**RG**DK**G**DK**G**K**OGVOGPOG**D**P | 48.4 | 25.3 |
| 0.8 | 793.5 | 5968 | A: G**W**VG**DK**G**D**PG**Q**RG**D**OG**D**RG**E**OG**F**RGPRG**E**A  B:  G**Q**RG**D**RG**D**OGP**K**G**F**OGPOG**F**RGPOG**F**RG**W**O  C:   G**QK**G**Q**OG**F**RG**EK**G**DK**GPRGPOG**D**OGPRG**W**R | 42.9 | 26.1 |
| 1.0 | 2245.1 | 17551 | A: GL**K**G**D**OG**D**OG**D**RG**D**PG**D**PG**EK**G**W**OGPPG**WK**  B:  G**D**OG**Y**RGPOGPRG**WK**GAOG**D**PG**D**OGPRG**D**P  C:   G**DK**GP**K**G**FK**GP**K**GPRG**D**RGPPGPRGPOG**W**R | 48.3 | 32.8 |

**Table S11.** Runtime at varying mutation rates – Trial 3

| Mutation Rate | Run time (sec) | Generation | Output | | |
| --- | --- | --- | --- | --- | --- |
|  |  |  | Sequences | Predicted Tm (°C) | Predicted ΔTm (°C) |
| 0.01 | 75.4 | 4925 | A: GPOG**D**RG**DK**G**D**RG**E**OG**W**OGPOG**W**OGPRGVO  B:  GPRGPOG**D**RG**EK**G**FK**GL**K**GPOGPOG**EK**G**W**P  C:   G**EK**G**F**OGP**K**G**K**RG**D**PG**D**RG**D**OGPAG**D**OG**Y**R | 47.8 | 30.9 |
| 0.1 | 42.8 | 618 | A: G**W**RG**D**OG**D**OG**D**PGAOGPPGPOGPOGPRG**W**O  B:  G**E**RG**F**OGP**K**GPOGPRGPOGPOGP**K**G**QK**G**WK**  C:   G**QK**G**FK**G**D**OG**D**OG**E**OG**F**OGPOG**D**RG**D**OG**D**O | 57.0 | 25.5 |
| 0.2 | 35.6 | 395 | A: GARGPOGPRG**D**OGP**K**GP**K**G**E**OG**W**RGPRG**E**O  B: G**QK**G**F**OGPPG**F**RG**E**OG**E**PG**D**PGP**K**GP**K**G**F**O  C: G**K**OG**EK**GPOG**E**PG**F**PGPRGPPGP**K**G**D**OG**D**P | 48.5 | 27.0 |
| 0.4 | 106.8 | 731 | A: GP**K**GPPG**DK**G**D**OGP**K**GPRGLOGPPGL**K**G**WK**  B:  G**D**OG**E**OG**K**PG**D**OG**K**RG**EK**G**F**OGAOG**DK**G**D**O  C:   G**WK**GP**K**GPOGPOG**Q**OG**D**OG**D**OGLRG**D**PG**D**R | 47.0 | 26.1 |
| 0.6 | 469.1 | 3336 | A: G**K**PGPOG**D**RG**D**PGPPG**FK**GP**K**G**Q**OGP**K**G**W**O  B:  GPRGPOGPPG**W**RG**K**RG**K**OG**D**PG**D**PG**D**RG**D**R  C:   G**QK**G**FK**GPOGPRGPOG**W**RGPPGPRGPOG**Y**R | 49.1 | 31.8 |
| 0.8 | 2484.7 | 19254 | A: GPAGPRG**D**OGPOG**W**RGVOG**KK**G**D**RG**D**PG**D**R  B:  G**EK**GPOG**FK**GPOGPRG**W**OG**D**PG**E**OG**Q**RGLP  C:   G**KK**G**E**OG**E**RG**D**AGPPG**FK**GP**K**GP**K**GPOG**W**O | 47.3 | 32.3 |
| 1.0 | 64457 | 19480 | A: GP**K**G**Q**OGPOG**W**OG**K**OG**DK**GP**K**G**E**OG**K**RG**D**O  B:  G**D**AG**DK**GLRGPRGPOG**K**RG**D**RG**D**OG**E**OG**WK**  C:   G**W**PG**D**RG**E**OG**QK**G**F**OGP**K**GPOG**QK**GPOG**K**O | 47.1 | 26.4 |

## **3.3. Comparison of GRACE performance at varying crossover rates**

**Table S12.** Comparison of GRACE average runtime at varying crossover rates. For selected amino acids, initial population size of 500, mutation rate of 0.2, target Tm of 47.0 °C, and target specificity of 25 °C, crossover rate of 0.6 resulted in the shortest average runtime across three trials.

| Crossover Rate | Runtime (sec) | | | |
| --- | --- | --- | --- | --- |
|  | Trial 1 | Trial 2 | Trial 3 | Average |
| 0.2 | 142.6 | 24.2 | 63.1 | 76.6 |
| 0.4 | 75.8 | 70.3 | 128.5 | 91.5 |
| **0.6** | **33.8** | **94.4** | **48.2** | **58.8** |
| 0.8 | 75.4 | 41.6 | 701.9 | 273.0 |
| 1.0 | 344.3 | 101.7 | 214.2 | 220.0 |

**Table S13.** Comparison of GRACE average generation at varying crossover rates. For selected amino acids, initial population size of 500, mutation rate of 0.2, target Tm of 47.0 °C, and target specificity of 25 °C, crossover rate of 0.6 resulted in the lowest generation across three trials.

| Crossover Rate | Generation | | | |
| --- | --- | --- | --- | --- |
|  | Trial 1 | Trial 2 | Trial 3 | Average |
| 0.2 | 994 | 186 | 436 | 538.7 |
| 0.4 | 358 | 342 | 602 | 434 |
| **0.6** | **130** | **367** | **187** | **228** |
| 0.8 | 256 | 431 | 2216 | 967.7 |
| 1.0 | 955 | 279 | 560 | 598 |

**Table S14.** Runtime at varying crossover rates – Trial 1

| Crossover Rate | Run time (sec) | Generation | Output | | |
| --- | --- | --- | --- | --- | --- |
|  |  |  | Sequences | Predicted Tm (°C) | Predicted ΔTm (°C) |
| 0.2 | 142.6 | 994 | A: GPAG**D**OG**D**OG**EK**G**W**OGPOG**QK**GVOG**D**RG**D**O  B:  G**E**OGVRGPOG**D**PG**DK**GP**K**G**D**OG**D**OGP**K**G**W**R  C:   GA**K**GPVG**W**RGPPGPRG**D**AG**D**RGP**K**G**D**OG**E**R | 47.1 | 32 |
| 0.4 | 75.8 | 358 | A: RGAOG**K**RG**EK**G**Q**OG**QK**G**EK**G**F**OGPPGPRGP  B:  **K**G**F**PGPPG**KK**G**D**PG**D**PG**D**RG**EK**GPOGPRG**Y**  C:   OG**WK**GPRG**D**RG**E**OGPRG**E**OG**Q**OG**D**OG**E**OG**W** | 48.3 | 25 |
| 0.6 | 33.8 | 130 | A: RG**E**OG**D**RGPRG**D**OG**D**OGPOG**W**OGPOG**D**RGL  B:  RG**W**OG**E**RG**Q**OG**F**RGPOGP**K**GPRGPOGPPG**W**  C:   **K**G**W**OG**Q**OG**DK**G**E**OG**F**RG**D**OG**E**OG**W**OGPOGA | 54.2 | 25.1 |
| 0.8 | 75.4 | 256 | A: **K**GP**K**G**E**OG**W**RGP**K**GPOGPOG**F**OGPOGP**K**G**W**  B:  AG**D**PG**D**OGAAG**D**OG**EK**GPOGLPGPOGPOG**D**  C:   OGVRGPOG**K**RGVRG**D**RG**D**OGPPGPRGPPGL | 48.9 | 25.7 |
| 1.0 | 344.3 | 955 | A: RGLPGP**K**GP**K**GP**K**GPOGPRG**F**OG**E**OG**WK**GP  B:  PG**W**RG**K**RG**D**OG**DK**G**D**PG**Q**PG**F**PGPOG**D**RG**D**  C:   OG**E**PG**Q**OG**F**PG**D**OG**W**RGPOGPRGPPGPOG**W** | 47.0 | 35 |

**Table S15.** Runtime at varying crossover rates – Trial 2

| Crossover Rate | Run time (sec) | Generation | Output | | |
| --- | --- | --- | --- | --- | --- |
|  |  |  | Sequences | Predicted Tm (°C) | Predicted ΔTm (°C) |
| 0.2 | 24.2 | 186 | A: **K**G**DK**G**W**RGPOGPRGPRG**K**OG**E**OG**F**RGPOG**W**  B:  PG**D**OG**E**OG**FK**G**EK**GPOGP**K**GPOGP**K**G**W**OGL  C:   **K**G**F**PG**K**OG**D**PG**D**PG**DK**GPRG**D**OGPRG**D**PGP | 47.2 | 27.5 |
| 0.4 | 70.3 | 342 | A: **K**G**WK**GLOGP**K**GAOGPRGPOG**EK**GPRG**D**RG**W**  B:  AG**D**RG**D**OG**K**OG**DK**GPOG**W**RG**K**PG**E**OG**Q**RG**Y**  C:   AGVOGPPG**E**OGPOG**D**OG**E**RG**WK**GPRGPOG**W** | 47.9 | 26.2 |
| 0.6 | 94.4 | 367 | A: G**F**PG**K**RG**D**OGPRGPOG**D**RGPAGPOG**D**RG**YK**  B:  GP**K**G**Q**RG**FK**GPOG**F**OGP**K**GPRGPOG**Q**OG**D**O  C:   G**DK**G**E**OGPPG**DK**GPOG**D**OG**E**PG**WK**GPOGPP | 47.9 | 27.3 |
| 0.8 | 41.6 | 431 | A: KGKPGDKGDRGDRGKOGDKGPAGWOGPOGY  B:  **K**G**Y**RG**D**RG**E**OGPRG**F**OG**D**RG**E**RGLOGPRGA  C:   **K**G**EK**GP**K**G**Q**OG**EK**G**Q**OG**Q**RGPPG**F**RGPOG**F** | 48.4 | 25.2 |
| 1.0 | 101.7 | 279 | A: OG**D**RG**D**PG**EK**GVOG**W**OG**D**OG**D**OG**D**RG**E**RG**W**  B:  **K**G**Q**AG**Q**OG**D**RG**E**OGPRGPOGPRGPPG**Q**AG**W**  C:   **K**G**Y**OGLAGPRG**Q**OG**EK**GP**K**GP**K**G**K**RGPOGV | 47.1 | 29.1 |

**Table S16.** Runtime at varying crossover rates – Trial 3

| Crossover Rate | Run time (sec) | Generation | Output | | | |
| --- | --- | --- | --- | --- | --- | --- |
|  |  |  | Sequences | Predicted Tm (°C) | Predicted ΔTm (°C) |  |
| 0.2 | 63.1 | 436 | A: GPRG**D**PGPOG**WK**GV**K**G**D**RGPOGP**K**GP**K**GPO  B:  G**QK**G**W**OGP**K**G**D**RG**D**OG**E**OG**F**PG**D**OG**D**RG**D**P  C:   G**KK**G**D**RG**K**AG**EK**G**F**OGPPGPOGPOGPOG**W**R | 49.0 | 30.5 |  |
| 0.4 | 128.5 | 602 | A: GL**K**G**K**PGVOG**F**RGPOG**D**OG**D**OGP**K**GPRGPP  B:  G**D**PG**WK**GPOGPRG**D**OGPAGPRG**D**OG**EK**G**W**V  C:   GPAG**D**RG**D**OGPOG**FK**GPOGVOG**W**OG**D**OG**D**O | 47.3 | 30.8 |  |
| 0.6 | 48.2 | 187 | A: AGPRG**Q**OG**F**RGPOG**DK**G**E**OGV**K**GP**K**GP**K**G**Q**  B:  **K**GVOG**F**RGPOG**W**OG**D**PG**D**OG**D**AG**DK**G**DK**G**D**  C:   RG**D**RGPOG**FK**GPOGPAGPPG**E**PG**D**PG**D**RG**D** | 47.7 | 31.1 |  |
| 0.8 | 701.9 | 2216 | A: **K**G**K**OG**E**OG**FK**GPOG**Q**OGPOGPOGPOGP**K**GP  B:  RG**W**PGPPG**DK**G**DK**GPOGP**K**GPOGP**K**G**K**OG**Y**  C:   **K**G**W**RGPOG**K**RG**D**OG**D**OG**K**OG**D**OG**D**OG**D**OGP | 61.9 | 25.3 |  |
| 1.0 | 214.2 | 560 | A: GP**K**GPOGPRGPOGPOG**W**AGPOG**F**AGPPG**W**R  B:  G**D**RG**D**OGP**K**G**W**OGPOG**K**OGVOGP**K**GV**K**G**E**O  C:   G**E**PG**F**OG**D**RG**D**RGPAGPRGPOG**D**RG**D**PG**D**R | 47.6 | 30.8 |  |

## **2.4. Comparison of GRACE performance at varying initial population size**

**Table S17.** Comparison of GRACE average runtime at varying initial population size. For selected amino acids, mutation rate of 0.2, crossover rate of 0.6, target Tm of 47.0 °C, and target specificity of 25 °C, initial population size of 500 resulted in the shortest average runtime across three trials.

| Initial population | Runtime (sec) | | | |
| --- | --- | --- | --- | --- |
|  | Trial 1 | Trial 2 | Trial 3 | Average |
| 10 | 178.5 | 114.4 | 189.1 | 160.7 |
| 50 | 50.9 | 238.0 | 342.7 | 210.5 |
| 100 | 94.5 | 88.0 | 402.6 | 195.0 |
| 200 | 156.9 | 64.5 | 532.8 | 251.4 |
| 300 | 136.6 | 596.7 | 35.1 | 256.1 |
| 400 | 65.0 | 385.6 | 58.9 | 169.8 |
| **500** | **56.5** | **122.9** | **93.9** | **91.1** |

**Table S18.** Comparison of GRACE average generation at varying initial population size. For selected amino acids, mutation rate of 0.2, crossover rate of 0.6, target Tm of 47.0 °C, and target specificity of 25 °C, initial population size of 500 resulted in the lowest average generation across three trials.

| Initial population | Generation | | | |
| --- | --- | --- | --- | --- |
|  | Trial 1 | Trial 2 | Trial 3 | Average |
| 10 | 733 | 428 | 725 | 628.7 |
| 50 | 200 | 942 | 1306 | 816.0 |
| 100 | 306 | 340 | 1578 | 741.3 |
| 200 | 572 | 238 | 1994 | 934.7 |
| 300 | 526 | 2399 | 140 | 1021.7 |
| 400 | 257 | 1493 | 229 | 659.7 |
| **500** | **216** | **493** | **365** | **358** |

**Table S19.** Runtime at varying initial population size – Trial 1

| Initial population | Run time (sec) | Generation | Output | | |
| --- | --- | --- | --- | --- | --- |
|  |  |  | Sequences | Predicted Tm (°C) | Predicted ΔTm (°C) |
| 10 | 178.5 | 733 | A: G**WK**G**D**RG**D**OGPOGPRG**D**RGPPG**D**OG**D**PGLR  B:  G**D**AG**E**PG**FK**GPOGP**K**G**Q**OG**QK**GPOGPRG**E**P  C:   G**EK**GPOG**D**RG**DK**GPOG**D**PG**DK**G**D**OG**Q**OG**Y**P | 52.8 | 25.3 |
| 50 | 50.9 | 200 | A: G**FK**GPOG**D**OG**D**OG**K**RG**D**PG**E**OG**F**AGPRGAO  B:  G**D**PG**D**OGPOGPRG**Q**OGA**K**GPOG**K**PGVRG**F**A  C:   G**QK**GP**K**GPOGP**K**G**FK**G**K**RG**D**OGPVGPPG**W**P | 47.7 | 27.2 |
| 100 | 94.5 | 306 | A: RG**K**OG**D**RGLOGPOG**D**RG**E**PGPRGPRG**D**OG**E**  B:  RG**W**RGPPG**DK**GPOGP**K**G**KK**GPRGPOG**D**RGP  C:   **K**G**Q**OG**K**OG**DK**G**D**OG**K**RG**D**PG**EK**GPOGPRG**Y** | 53.2 | 25.4 |
| 200 | 156.9 | 572 | A: GP**K**G**EK**GVOGP**K**GPOGVRG**K**OG**E**OG**W**OG**Q**O  B:  G**D**PG**D**PG**D**RG**D**OG**D**PGPRG**F**RGPOGP**K**GPO  C:   GPAGPOGPOG**F**AGPOG**EK**GPRG**F**OG**D**RG**D**O | 47.1 | 29.5 |
| 300 | 136.6 | 526 | A: GP**K**GPRG**Q**OG**F**AGPOG**D**PGPPGPPGP**K**G**W**O  B:  G**DK**G**EK**G**F**OGPOGVOGPPGP**K**GPOG**D**RG**D**O  C:   G**D**PG**D**RG**D**OGP**K**GPOGPOG**D**PG**D**RGPPG**Y**R | 57.6 | 25.6 |
| 400 | 65.0 | 257 | A: **K**G**D**OG**E**PG**F**OGPOG**K**RGPOGPPG**W**RG**D**RG**D**  B:  OG**D**RGPPGPRG**E**PGPPG**FK**GPRGPOG**Q**RG**Y**  C:   **K**G**Q**RG**Q**OGPRG**Q**PG**K**RG**K**RG**E**PG**DK**GPOG**W** | 48.6 | 26.7 |

**Table S20.** Runtime at varying initial population size – Trial 2

| Initial population | Run time (sec) | Generation | Output | | |
| --- | --- | --- | --- | --- | --- |
|  |  |  | Sequences | Predicted Tm (°C) | Predicted ΔTm (°C) |
| 10 | 114.4 | 428 | A: **K**G**D**OG**D**RGP**K**GP**K**G**FK**GPOG**D**OG**DK**G**DK**G**D**  B:  **K**G**D**PG**Q**OG**Q**OG**D**OG**D**RG**D**OGPOG**D**PG**D**RG**Y**  C:   **K**G**Y**OGPPGLRGPOGP**K**G**FK**GPOGP**K**GPOG**D** | 60.0 | 25.8 |
| 50 | 238.0 | 942 | A: GP**K**G**K**OG**YK**GPOGPOG**DK**G**D**OGP**K**GPOG**W**O  B:  G**D**RG**D**PGPRG**D**OGPOGPPG**D**OG**K**RG**EK**GP**K**  C:   G**EK**G**Q**OGPOG**WK**GP**K**GPOGPOGPRGPOG**D**P | 56.0 | 25.1 |
| 100 | 88.0 | 340 | A: RG**D**PG**D**OGLOGP**K**G**Q**OG**W**OGPPGPOGPRG**W**  B:  **K**G**W**RGPOGPOG**DK**G**DK**GPRGAPGPOGP**K**G**F**  C:   **K**G**Y**OG**W**OGPRG**D**RG**E**OG**Y**OG**Y**OGPRG**D**OG**D** | 54.0 | 26.5 |
| 200 | 64.5 | 238 | A: RG**D**PG**D**OGLOGP**K**G**Q**OG**W**OGPPGPOGPRG**W**  B:  **K**G**W**RGPOGPOG**DK**G**DK**GPRGAPGPOGP**K**G**F**  C:   **K**G**Y**OG**W**OGPRG**D**RG**E**OG**Y**OG**Y**OGPRG**D**OG**D** | 48.8 | 26.8 |
| 300 | 596.7 | 2399 | A: RGPOG**Q**OG**F**RGPOGPOGPOG**D**OG**D**OGPOG**F**  B:  **K**G**W**OGPOG**EK**G**F**OGP**K**GPOGPOGPOGPRGP  C:   RG**D**RGPOG**D**PG**D**PG**D**OG**DK**GPOGPRGPOG**W** | 66.2 | 25.4 |
| 400 | 385.6 | 1493 | A: G**FK**G**E**PGVRGP**K**GPRG**W**OGPOG**D**OG**D**RG**Y**R  B:  G**DK**G**Y**RG**E**OG**D**OG**E**PG**W**RGPOGPOGP**K**G**Q**O  C:   G**D**AG**E**OG**W**OGPRGPOGP**K**G**FK**GP**K**G**D**OG**D**O | 47.4 | 32.7 |
| 500 | 122.9 | 493 | A: GLPGPRGPOG**D**RGPRGPOG**D**OG**D**RG**K**AGVR  B:  GP**K**GP**K**G**F**OGPOG**Q**OG**W**OGPPGPOGPRGVA  C:   G**D**OG**DK**G**D**OGPPGPOGA**K**GPOGPAGPOG**W**R | 52.1 | 25.3 |

**Table S21.** Runtime at varying initial population size – Trial 3

| Initial population | Run time (sec) | Generation | Output | | |
| --- | --- | --- | --- | --- | --- |
|  |  |  | Sequences | Predicted Tm (°C) | Predicted ΔTm (°C) |
| 10 | 189.1 | 725 | A: **K**G**K**OG**E**OG**Q**OG**D**OGP**K**GPOGP**K**G**K**OG**DK**GL  B:  RG**Y**OGP**K**GP**K**GP**K**G**D**OG**D**OG**D**PG**D**OG**D**RG**D**  C:   **K**G**F**RGP**K**G**D**OG**D**PG**D**PGPOGP**K**GPOGPOG**W** | 63.9 | 25.7 |
| 50 | 342.7 | 1306 | A: RG**D**OG**D**OGPOG**D**OGP**K**G**FK**GP**K**GPOG**F**OG**K**  B:  **K**G**WK**GPOGP**K**GPRG**D**OG**D**PG**DK**G**D**OGPRGL  C:   **K**G**D**OG**DK**G**D**OG**E**RG**W**OGPOG**D**RG**D**OGPOG**W** | 62.3 | 25.5 |
| 100 | 402.6 | 1578 | A: G**FK**GPOG**F**OG**D**OG**D**OGPOG**F**OGPOG**DK**G**Y**O  B:  G**D**AG**DK**GPRGP**K**GPOGP**K**GPOGP**K**GPOG**D**R  C:   G**K**RG**D**OG**EK**GPOG**D**RG**D**OG**D**OG**DK**G**D**PG**Q**P | 58.2 | 25.1 |
| 200 | 532.8 | 1994 | A: **K**GPRGPOG**WK**GPOG**FK**GP**K**G**W**OGPPG**Q**RGV  B:  AG**YK**G**F**OG**D**PG**D**AG**D**OG**D**OG**DK**GPOGP**K**G**W**  C:   PGVRG**D**PGPRGPOGVRGPPG**D**RG**D**AG**K**OG**Y** | 48.2 | 37.2 |
| 300 | 35.1 | 140 | A: RGVOG**E**OGPOG**E**PGPRG**D**RG**K**PG**D**RGP**K**GP  B:  **K**G**WK**GPOGPRGAOG**E**PG**Q**RG**Y**OGP**K**G**D**OG**D**  C:   VG**D**RG**DK**GPOG**FK**GPOGP**K**G**F**OG**K**OG**D**OGP | 49.1 | 25.2 |
| 400 | 58.9 | 229 | A: AG**D**PG**D**OGP**K**G**E**VG**F**PGPRG**D**OG**E**PGPRG**F**  B:  OGVRGPAG**D**PG**D**RGP**K**GP**K**G**F**RGPOG**E**RG**F**  C:   **K**G**E**OG**QK**GVRGPOG**D**PG**DK**G**D**RG**F**RGPOG**W** | 47.7 | 29.7 |
| 500 | 93.9 | 365 | A: GP**K**G**K**OG**KK**G**E**OG**WK**GPOGPOG**E**OG**DK**G**Y**O  B:  G**D**RG**D**RG**D**OG**D**RG**KK**G**DK**GPOG**K**OG**D**PG**D**O  C:   G**Q**AGP**K**G**F**RGPPG**D**RG**DK**G**D**RGP**K**GPOGPR | 48.5 | 29.3 |

##

**2.5. Summary of GA parameters used in GRACE**

**Table S22.** Summary of GA parameters

| Parameter | Value |
| --- | --- |
| Mutation rate | 0.2 |
| Crossover rate | 0.6 |
| Initial population size | 500 |
| Number of parents | 2 |
| Running population size | varying |

# **4. Comprehensive Tm predictions of all possible assemblies from GRACE-generated peptides**

**Table S23.** Comprehensive Tm predictions of all possible registers. The most stable registers are highlighted in blue; the second most stable registers are highlighted in red.

| Register | Melting temperature (°C) | | | |
| --- | --- | --- | --- | --- |
|  | ABC-1 | ABC-2 | AAB-FOGER | ABC-FOGER |
| AAA | < 10.0 | 14.3 | **28.4** | <10.0 |
| AAB | 23.7 | 33.7 | **45.8** | 20.9 |
| AAC | 14.0 | 32.6 | N/a | 13.9 |
| ABA | 14.8 | 27.8 | 17.3 | 17.3 |
| ABB | 23.6 | 31.0 | 26.4 | 22.8 |
| ABC | **39.7** | **63.9** | N/a | **47.5** |
| ACA | <10.0 | 12.3 | N/a | <10.0 |
| ACB | <10.0 | 13.5 | N/a | 16.0 |
| ACC | 13.6 | 28.3 | N/a | 22.9 |
| BAA | <10.0 | 13.1 | 13.2 | <10.0 |
| BAB | 14.9 | 33.9 | 23.2 | <10.0 |
| BAC | <10.0 | 20.6 | N/a | <10.0 |
| BBA | <10.0 | 10.5 | <10.0 | <10.0 |
| BBB | <10.0 | 15.1 | <10.0 | <10.0 |
| BBC | **24.0** | 35.7 | N/a | 14.6 |
| BCA | <10.0 | 28.8 | N/a | 15.2 |
| BCB | 15.2 | 31.5 | N/a | 18.9 |
| BCC | 23.7 | 34.0 | N/a | 21.6 |
| CAA | <10.0 | 24.5 | N/a | <10.0 |
| CAB | 11.3 | 35.0 | N/a | 14.6 |
| CAC | <10.0 | 24.4 | N/a | <10.0 |
| CBA | <10.0 | 19.9 | N/a | <10.0 |
| CBB | <10.0 | 14.3 | N/a | <10.0 |
| CBC | 10.6 | **37.6** | N/a | **24.9** |
| CCA | <10.0 | 20.1 | N/a | <10.0 |
| CCB | <10.0 | 12.6 | N/a | 12.5 |
| CCC | <10.0 | 17.7 | N/a | 14.0 |

# **5. Mass spectra and UPLC of GRACE-generated peptides**

**Table S24.** Sequences, average mass, and observed m/z of GRACE-generated peptides; G: ^15^N glycine; **G**: ^15^N-^13^C glycine.

| Peptide | Sequence | Expected | Observed |
| --- | --- | --- | --- |
| ABC-1  A  B  C | QGFOGIKGPOGDOGPOGPR**G**DOGNOGYKGP    QGFDGPOGDOGPQGPRGPOGYKGPOGPRGD    OGRQGNKGPOGIOGPKGYQ**G**PQGDOGPOGF | [M+H] ^+^  3008.7  [M+H] ^+^  3019.1  [M+H]^+^  3036.2 | [M+H] ^+^  3009.0  [M+H] ^+^  3020.2  [M+H] ^+^  3037.4 |
| ABC-2  A  B  C | RGLOGPOGPOGIKGPOGKKGPOGIDGPRGW  QGFQGPKGPOGPOGDK**G**POGDRGKOGPRGW  QGIOGPOGDQGPOGPDGDOGPQGFRGPOGW | [M+H] ^+^  3020.4  [M+H]^+^  3087.33  [M+H]^+^  3015.1 | [M+H] ^+^  3021.7  [M+H] ^+^  3088.6  [M+H] ^+^  3016.8 |
| AAB-FOGER  A  B | PRGPRGDOGPRGFOGERGDKGDOGPRGDRG  QKGPOGPRGQOGFKGPKGPOGDKGQOGFOG | [M+H] ^+^  3161.3  [M+H] ^+^  3049.3 | [M+H] ^+^  3161.4  [M+H] ^+^  3049.9 |
| ABC-FOGER  A  B  C | YKGKOGDKGPKGPOGWOGPOGDRGDOGEOG  DRGDRGKOGDRGDOGPKGPOGPPGYKGPOG  EKGPOGYOGPRGFOGERGDKGPKGPRGDPG | [M+5H]^5+^  619.7  [M+4H]^4+^  774.3  [M+3H]^3+^  1032.1  [M+6H]^6+^  508.7  [M+5H]^5+^  610.3  [M+4H]^4+^  762.6  [M+3H]^3+^  1016.4  [M+6H]^6+^  518.9  [M+5H]^5+^  622.5  [M+4H]^4+^  777.8  [M+3H]^3+^  1036.8 | [M+5H]^5+^  619.6  [M+4H]^4+^  774.2  [M+3H]^3+^  1032.1  [M+6H]^6+^  508.9  [M+5H]^5+^  610.4  [M+4H]^4+^  762.8  [M+3H]^3+^  1016.9  [M+6H]^6+^  518.9  [M+5H]^5+^  622.4  [M+4H]^4+^  777.8  [M+3H]^3+^  1037.0 |


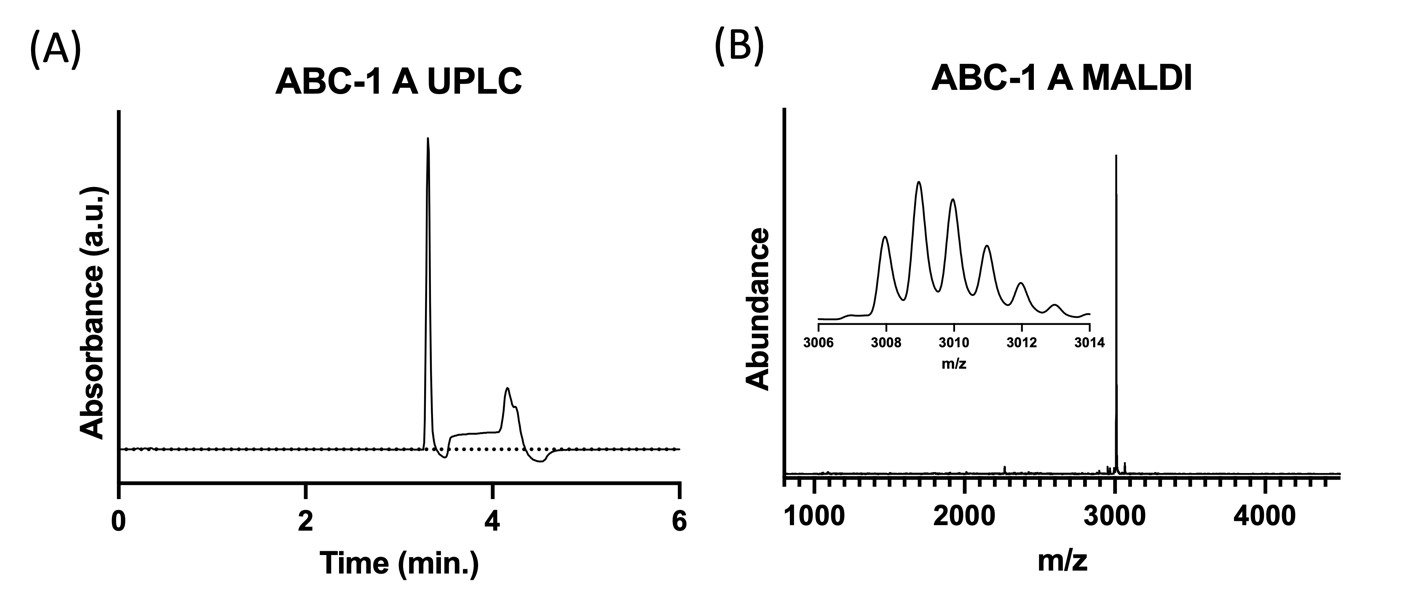


**Figure S26.** Characterization of ABC-1 peptide A. (A) UPLC trace. (B) MALDI-ToF mass spectrum


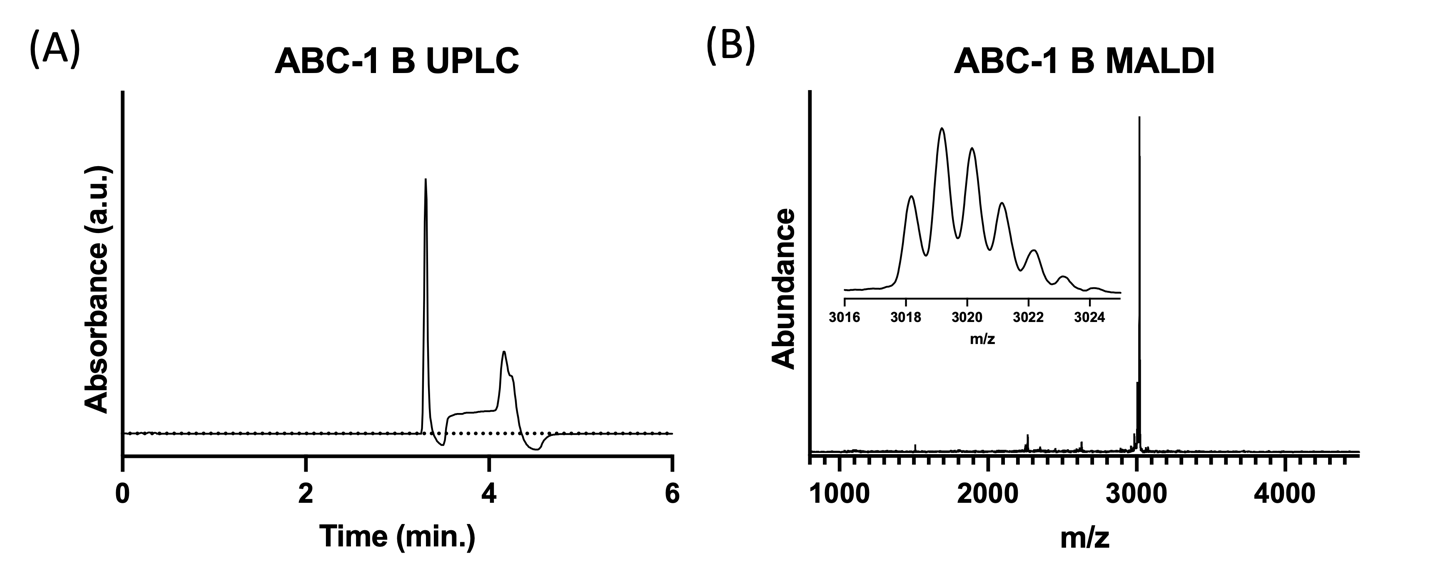


**Figure S27.** Characterization of ABC-1 peptide B. (A) UPLC trace. (B) MALDI-ToF mass spectrum


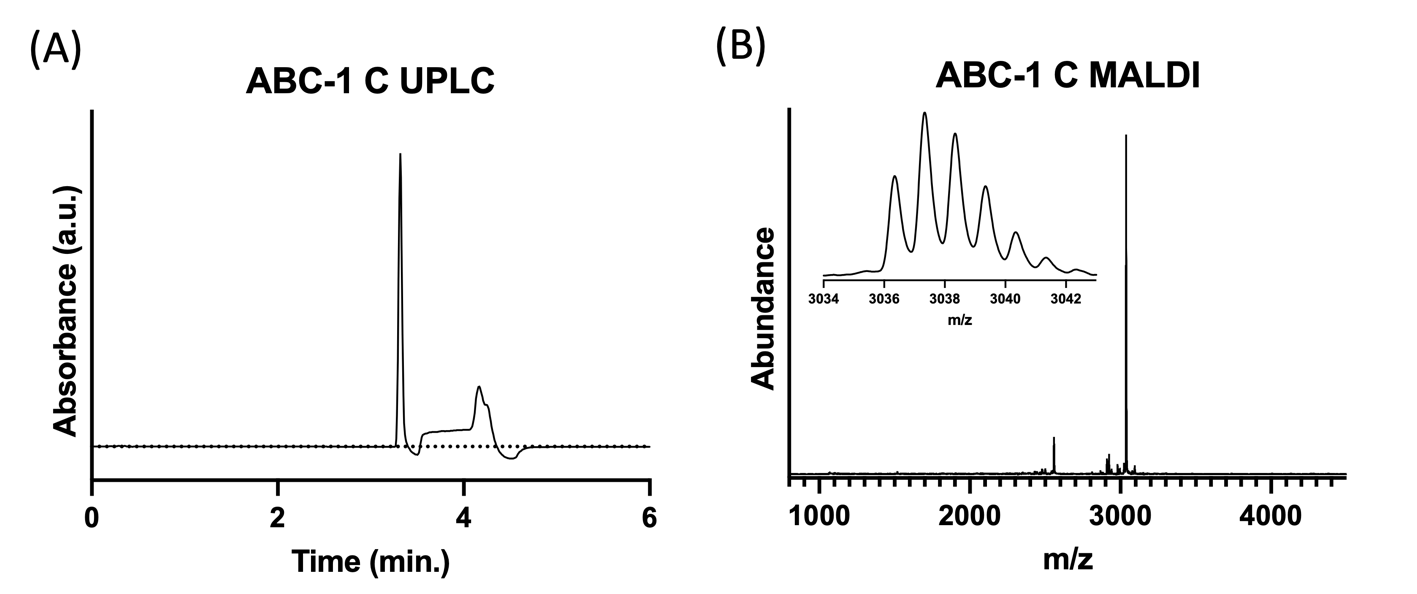


**Figure S28.** Characterization of ABC-1 peptide C. (A) UPLC trace. (B) MALDI-ToF mass spectrum


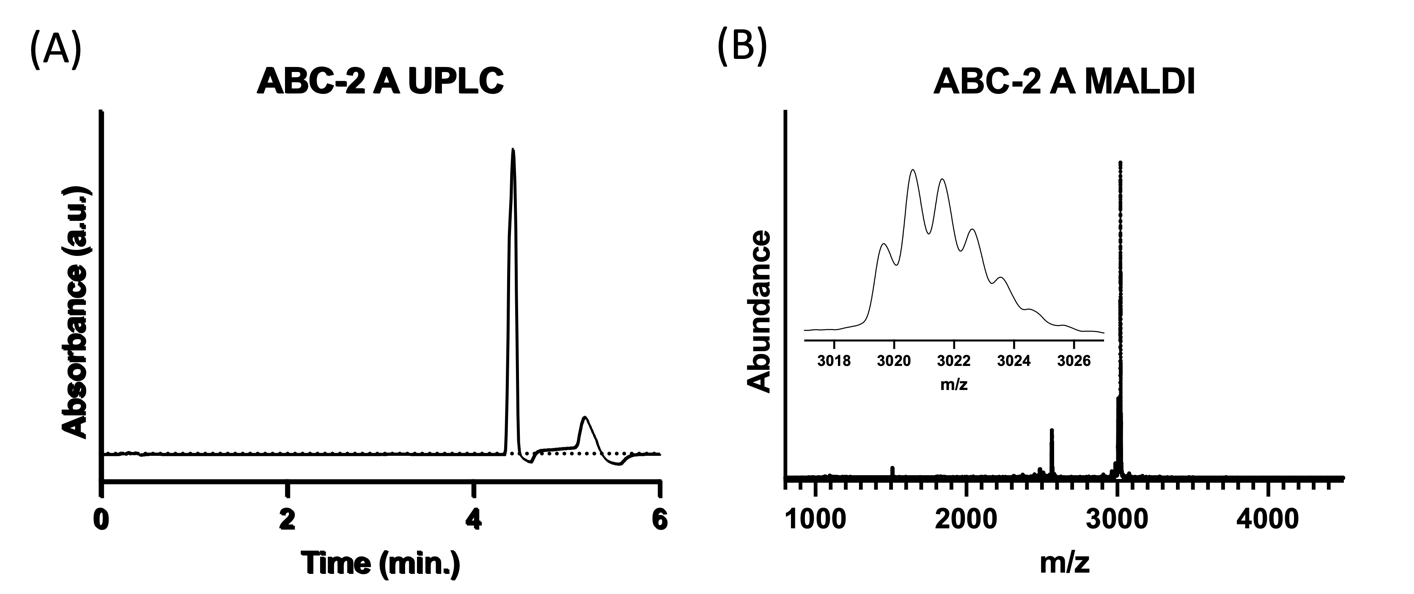


**Figure S29.** Characterization of ABC-2 peptide A. (A) UPLC trace. (B) MALDI-ToF mass spectrum


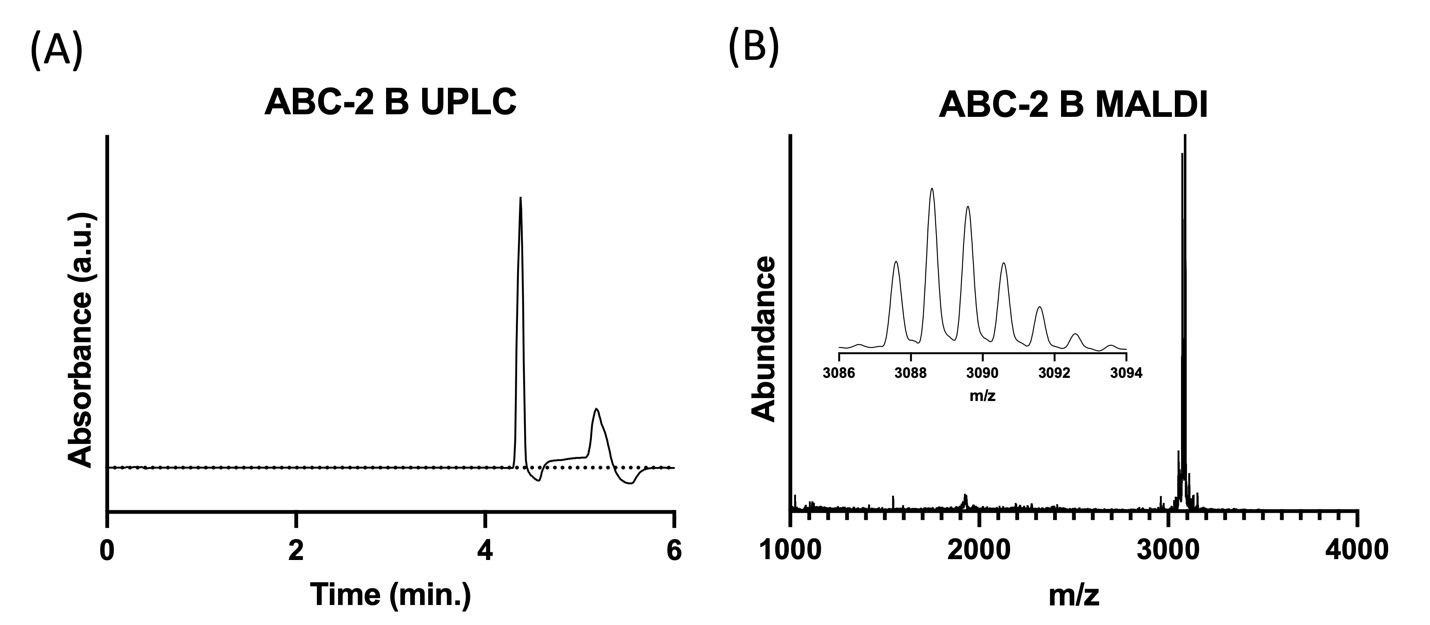


**Figure S30.** Characterization of ABC-2 peptide B. (A) UPLC trace. (B) MALDI-ToF mass spectrum


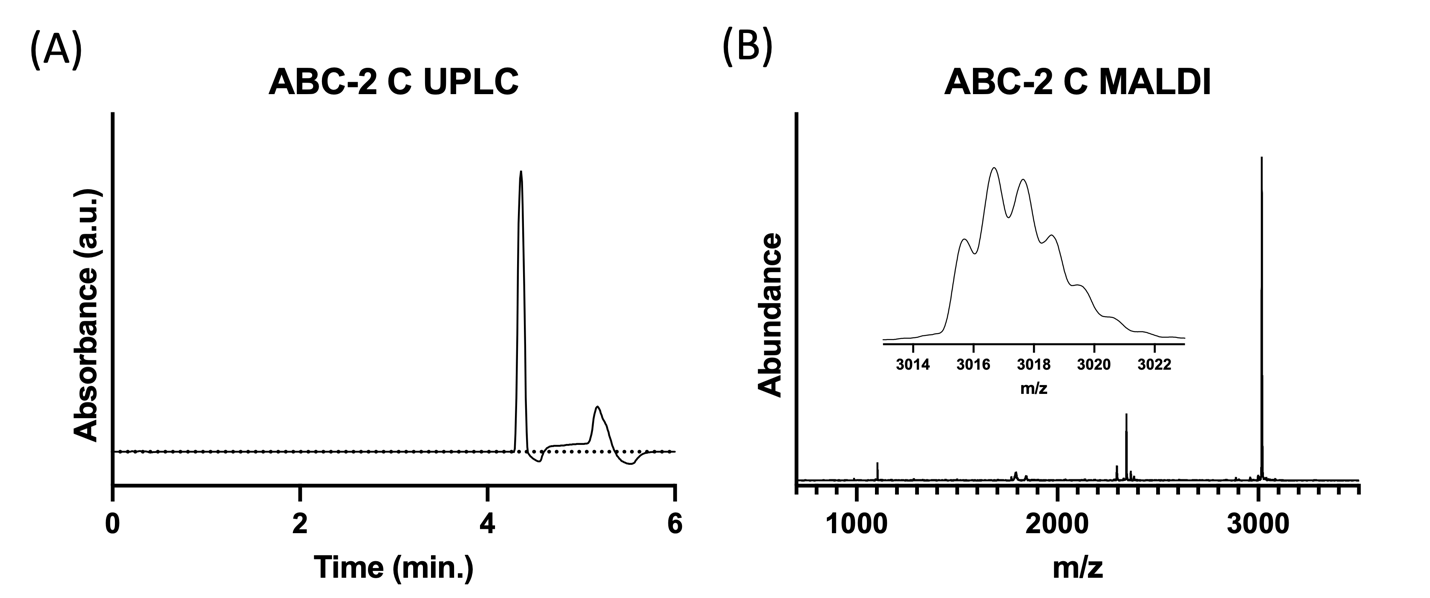


**Figure S31.** Characterization of ABC-2 peptide C. (A) UPLC trace. (B) MALDI-ToF mass spectrum


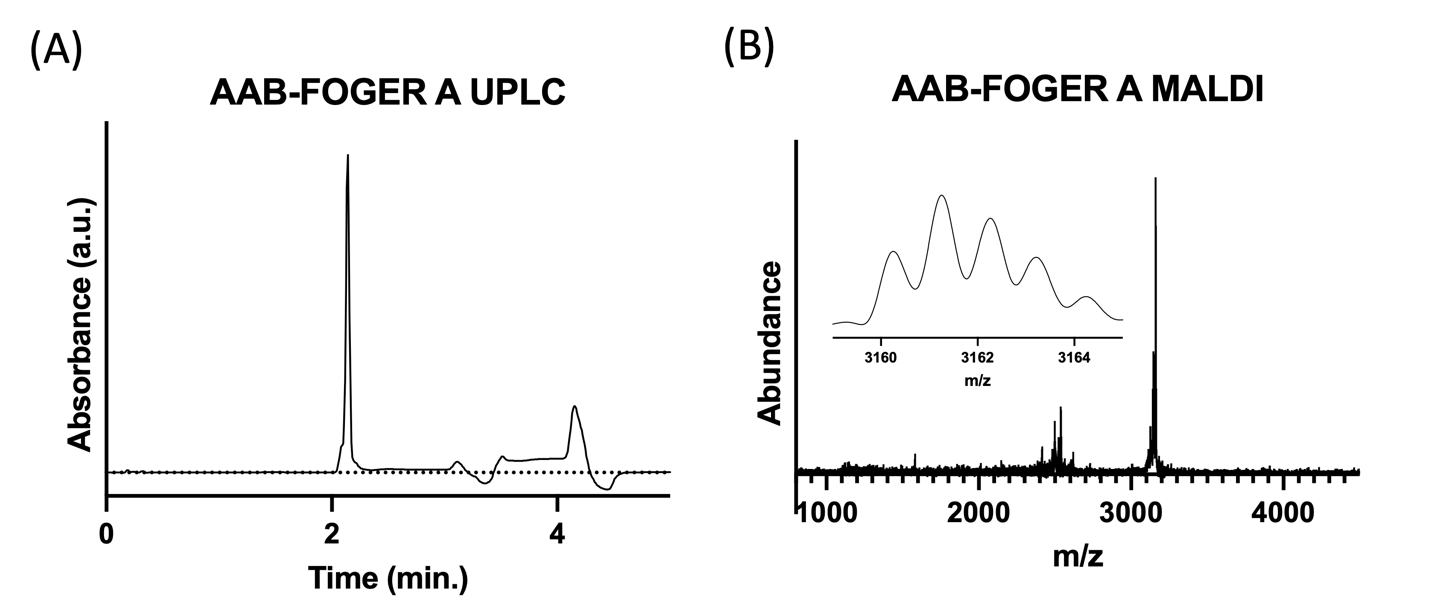


**Figure S32.** Characterization of AAB-FOGER peptide A. (A) UPLC trace. (B) MALDI-ToF mass spectrum


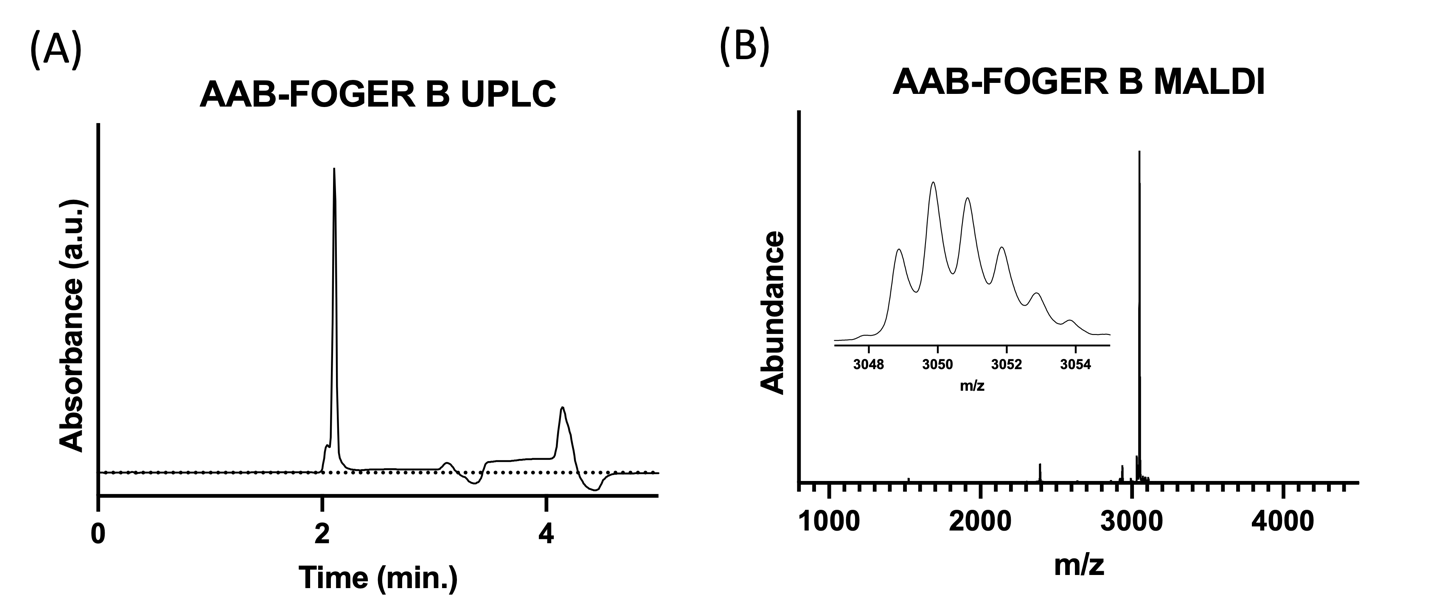


**Figure S33.** Characterization of AAB-FOGER peptide B. (A) UPLC trace. (B) MALDI-ToF mass spectrum


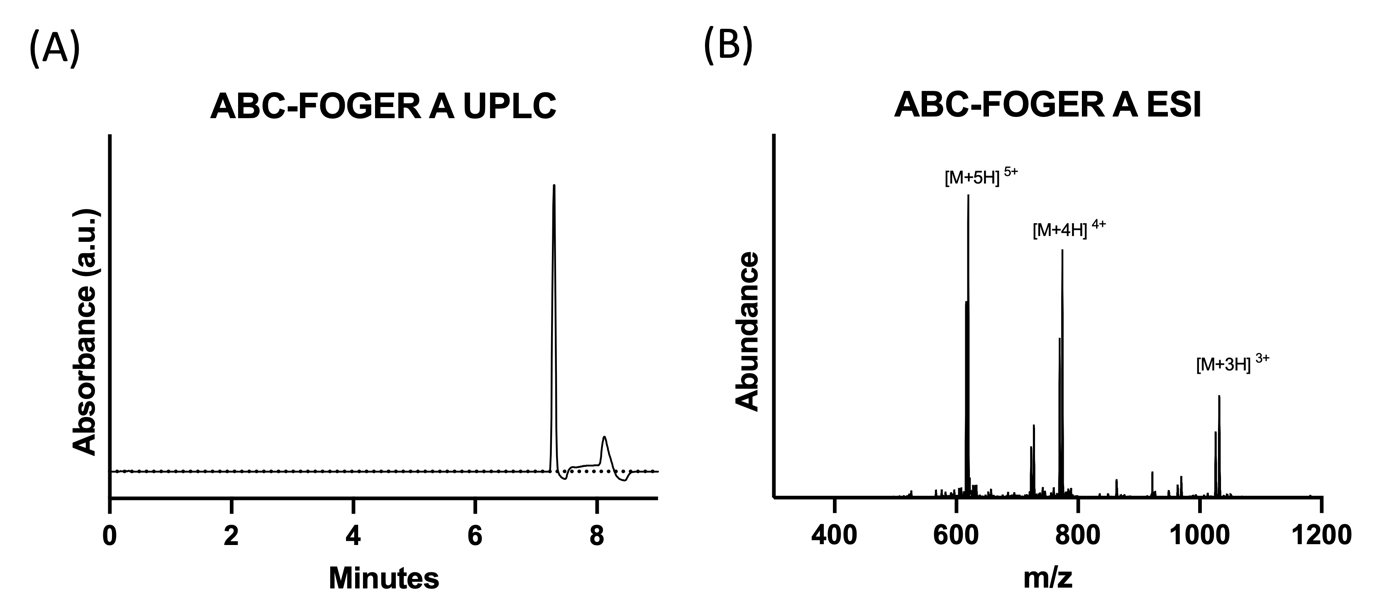


**Figure S34.** Characterization of ABC-FOGER peptide A. (A) UPLC trace. (B) ESI mass spectrum


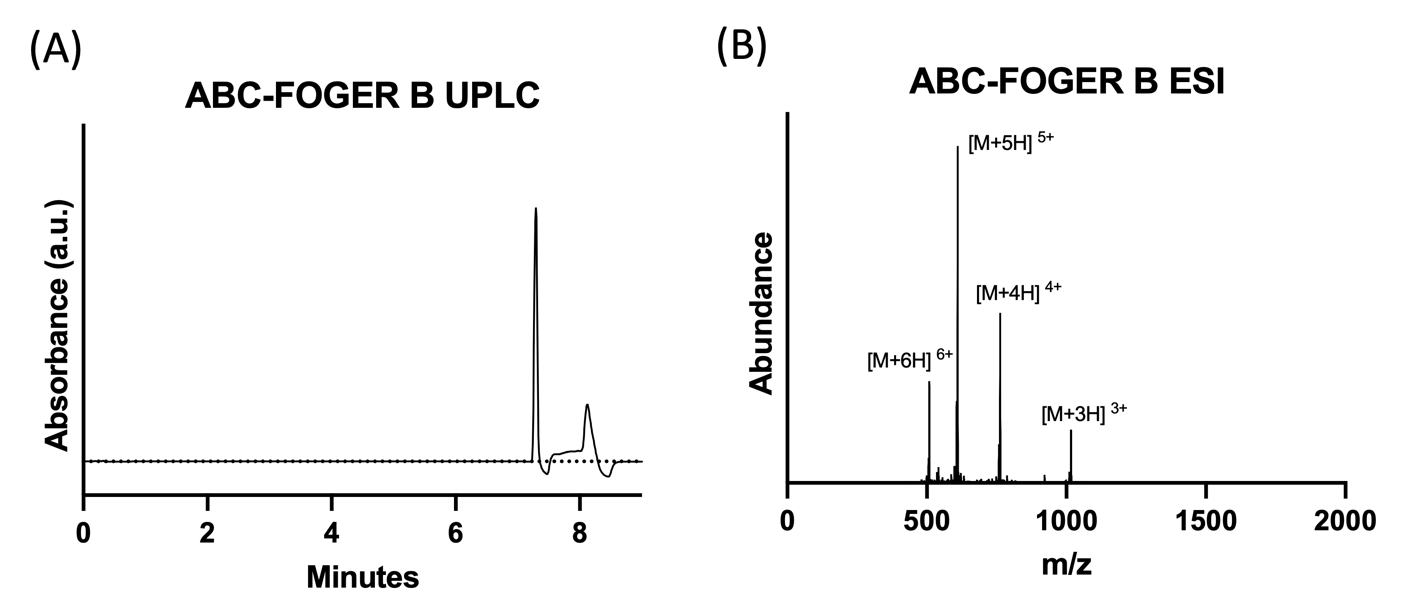


**Figure S35.** Characterization of ABC-FOGER peptide B. (A) UPLC trace. (B) ESI mass spectrum


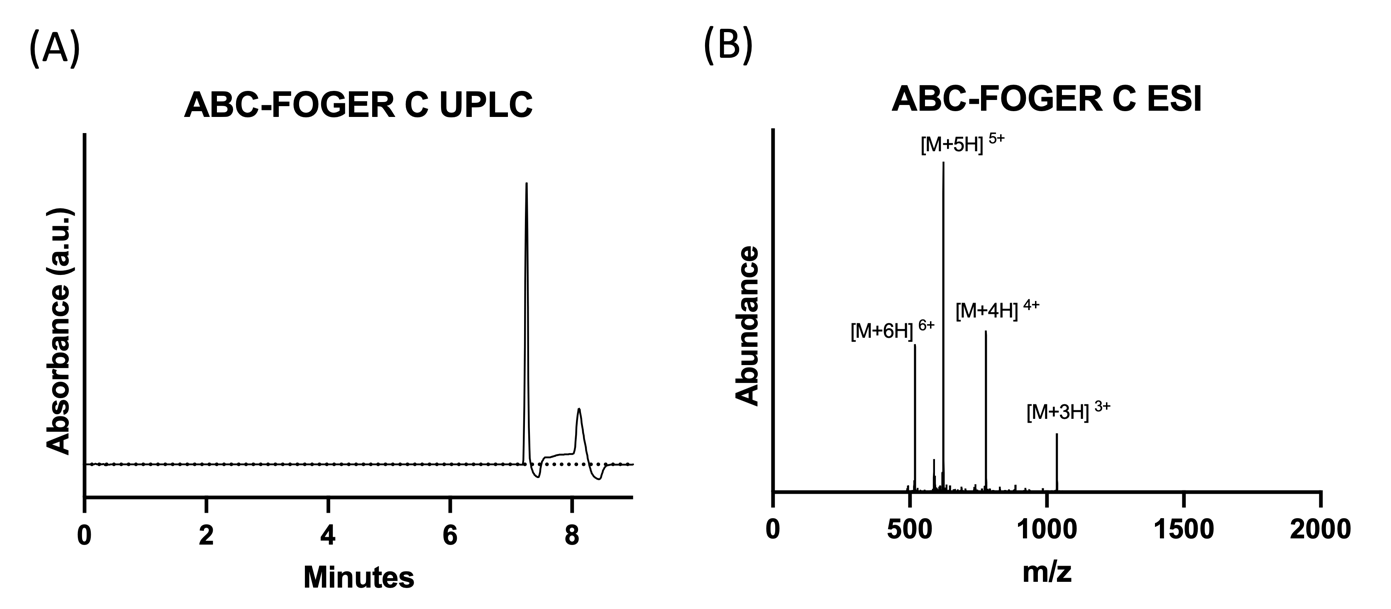


**Figure S36**. Characterization of ABC-FOGER peptide C. (A) UPLC trace. (B) ESI mass spectrum

# **6. CD characterization of GRACE-generated heterotrimers**


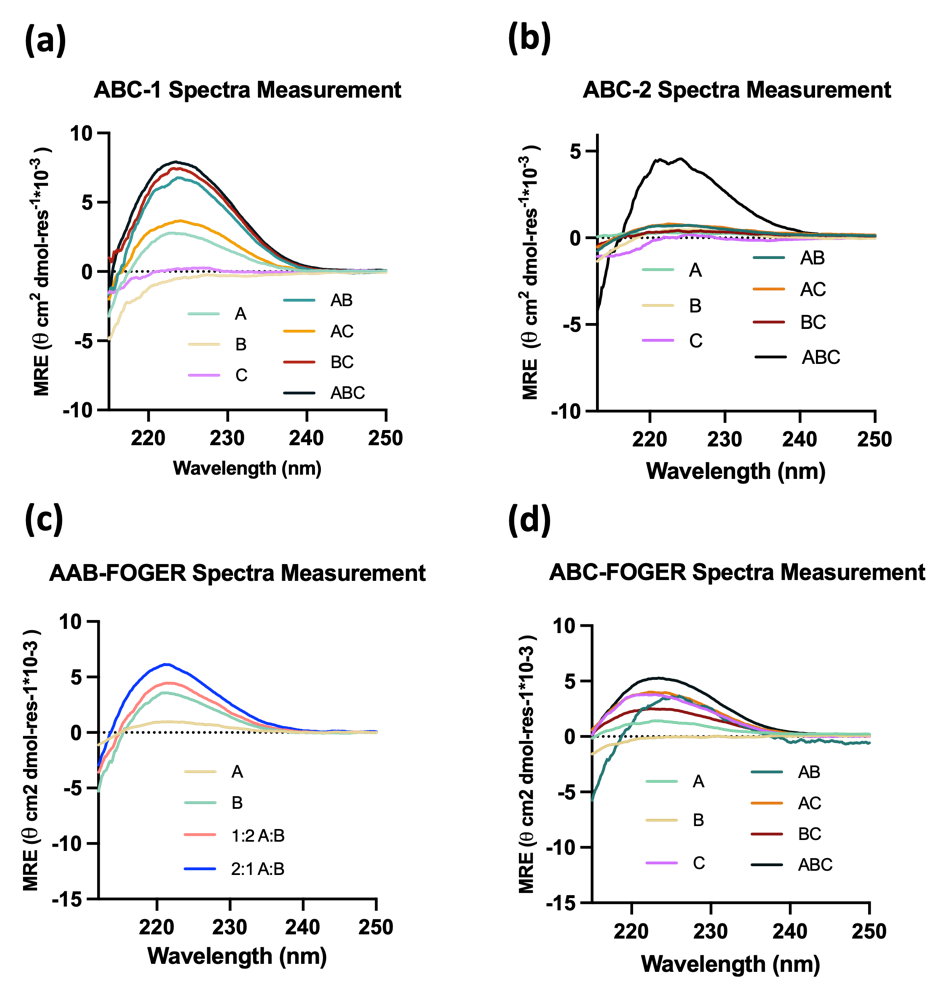


**Figure S37.** Spectra measurement of GRACE-generated heterotrimers collected at 5 °C. Maximum at around 225 nm is indicative of triple helical structure. (a) ABC-1, (b) ABC-2, (c) AAB-FOGER, (d) ABC-FOGER

**
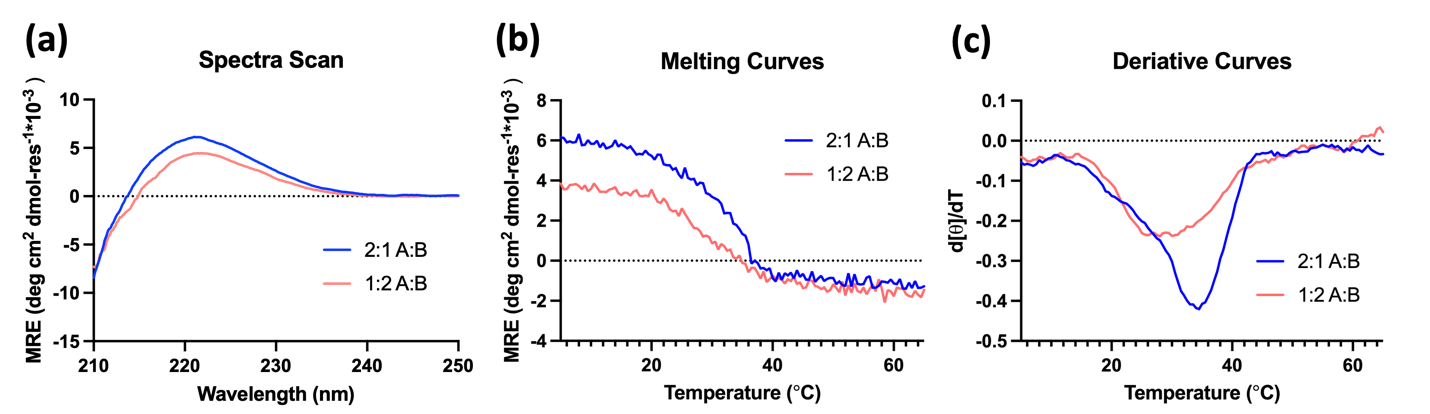
**

**Figure S38.** CD of AAB-FOGER peptide mixtures with different mixing ratio (a) Wavelength scans of AAB-FOGER 2: 1 A: B and 1: 2 A : B mixtures; (b) Melting curves; (c) Derivative curves

# **7. Structural characterization by NMR**

## **7.1. Structural analysis of competing assemblies at 10 °C**

Structural characterization of competing species in the ABC-1 and ABC-FOGER systems was performed using ^1^H–^15^N HSQC NMR at 10 °C. At this lower temperature, spectra are expected to reveal signals corresponding to target assemblies, competing assemblies and monomeric peptides as well as conformational heterogeneity among these species.

In the ABC-1 system, ^1^H–^15^N HSQC NMR spectrum of A unary solution at 10 °C exhibited five cross peaks (Figure S43). One peak matched the monomer A signal previously observed at 25 °C, while a nearby cross peak was assigned to an alternative conformation of monomer A. Three distinct cross peaks, marked with solid circles, indicates a folded homotrimer A, consistent with the experimental Tm of 11 °C. The B unary solution precipitated at 10 °C, and therefore NMR data of this sample was not collected. The CD spectrum (Figure S37a) and melting curve (Figure 3a and b) of peptide C indicate no triple helix formation. Therefore, the cross peaks observed at 10 °C (Figure S44), in addition to the monomer peak identified in the unary C spectra at 25 °C (Figure 3c), can be attributed to the conformational heterogeneity of monomer C.

In binary mixtures of ABC-1 at 10 °C, the A: B solution revealed six additional cross peaks beyond peaks identified from A and B unary solutions (Figure S45), suggesting the presence of at least two distinct triple helical registers. Based on GRACE predictions, the additional cross peaks can be assigned to the AAB and ABB registers, with predicted melting temperatures of 23.7 °C and 23.6 °C, respectively (Table S23). Although these predicted Tm values deviate from experimental data of 19.5 °C, the prediction of highly similar Tm for the two assemblies is consistent with the observation of co-existing registers. In A:C binary solution, in addition to peaks identified from A and C unary solutions, three distinct cross peaks were observed (Figure S46), consistent with the observed CD transition at 17.5 °C. This suggests the presence of additional helical registers, hypothetically assigned to AAC or ACC based on SCEPTTr predictions (Table S23). Similarly, the B: C binary mixture exhibited four different cross peaks, along with peaks that overlap with peaks identified from B and C unary solutions (Figure S47). This implies the presence of least two distinct heterotrimeric registrations, consistent with experimental Tm of 20.0. These observed cross peaks can be attributed to BBC and BCC registers based on the predicted values (Table S23).

In the ABC-FOGER system, the HSQC spectrum of the A unary solution at 10 °C showed one intense cross-peak corresponding to monomer A (Figure S56), matching the chemical shift observed at 25 °C (Figure 5f). As the CD data indicates minimal homotrimer A formation (Figure S37d and Figure 5d-e), the two additional cross-peaks observed at 10 °C are attributed to conformational heterogeneity of monomer A. In the B unary solution, one intense monomer peak was observed along with two additional peaks at nearby chemical shifts, likely corresponding to different conformations of monomer B (Figure S57). For the C unary solution, the 10 °C spectrum showed one intense peak matching the monomer C signal at 25 °C, along with seven additional cross-peaks (Figure S58). These additional peaks may arise from homotrimer C of different helical registers, corresponding to CD transition at 13 °C (Figure 5d-e), or from conformational heterogeneity of monomer C.

Among binary mixtures of ABC-FOGER, the A:C solution appeared to form the most thermally stable competing species, as indicated by the CD transition temperature of 26 °C (Figure 5d-e). HSQC NMR analysis at 10 °C revealed three additional cross peaks besides those observed in the A and C unary spectra, confirming the formation of a heterotrimeric triple helix, which is predicted to adopt the ACC register (Table S23). The A: B and B: C mixtures were less stable, with experimental Tm values of 20.0 °C and 21.5 °C, respectively, thereby showing lower-intensity peaks with signal overlap in HSQC spectra. In the A: B mixture, besides peaks identified from A and B unary spectra, three distinct cross-peaks were present (Figure S59), which can be assigned to ABB heterotrimers with higher predicted Tm, and four lower-intensity cross-peaks (Figure S59), potentially assigned to AAB heterotrimer due to its lower predicted Tm (Table S23). The B: C mixture exhibited four distinct cross-peaks in addition to peaks observed in B and C unary spectra, and one or two peaks overlapping with monomer signals, suggesting the presence of at least two registers, tentatively assigned to CBC and BCC, with the latter predicted to be less stable (Table S23).

While the registrations were not resolved for non-target assemblies, observations from ^1^H-^15^N HSQC were consistent with CD data and predictions by GRACE.

## **7.2. ABC-1**

**Table S25.** NOESY-HSQC peak assignments of heterotrimer ABC-1

| Plane at ^15^N chemical shift of 107.81 ppm – peptide A – leading strand | |
| --- | --- |
| 1.08 ppm | NOE to Arg19 Cγ-H |
| 1.38 ppm | NOEs to Lys16 C𝛿-H on peptide C |
| 1.80 and 1.57 ppm | NOEs to Arg19 Cβ-H |
| 3.06 ppm | NOE to Lys16 C𝜀-H on peptide C |
| 3.39 ppm | NOEs to labeled Gly20 C𝛼-H |
| 3.99 ppm | NOE to Arg19 C𝛼-H |
| 4.89 ppm | NOE to Hyp19 NH on peptide B |
| 7.79 ppm | NOE to labeled Gly20 NH |
| 8.11 ppm | NOE to labeled Gly20 NH on peptide B |
| 8.92 ppm | NOE to Gly17 NH on peptide C |
| Plane at ^15^N chemical shift of 104.53 ppm – peptide B – middle strand | |
| 1.93 and 2.15 ppm | NOE to Hyp19 Cβ-H |
| 3.38 and 3.76 ppm | NOEs to the labelled Gly20 Cα-H |
| 4.30 ppm | NOE to Gln19 Cα-H on peptide C |
| 4.89 ppm | NOE to Hyp19 Cα-H |
| 7.89 ppm | NOE to Gly20 NH on peptide A |
| 8.11 ppm | NOE to labeled Gly20 NH |
| Plane at ^15^N chemical shift of 110.16 ppm – peptide C – trailing strand | |
| 1.80 and 2.08 ppm | NOEs to Gln19 Cβ-H |
| 2.76 ppm | NOE to Gln19 Cγ-H |
| 3.39 and 3.76 ppm | NOEs to the labelled Gly20 Cα-H |
| 4.31 ppm | NOE to Gln19 C𝛼-H |
| 4.92 ppm | NOE to Hyp22 C𝛼-H on peptide A |
| 8.22 ppm | Overlap NOE to labeled Gly20 NH and NOE to Gly20 NH on peptide B |
| 8.43 ppm | NOE to Gly23 NH on peptide A |


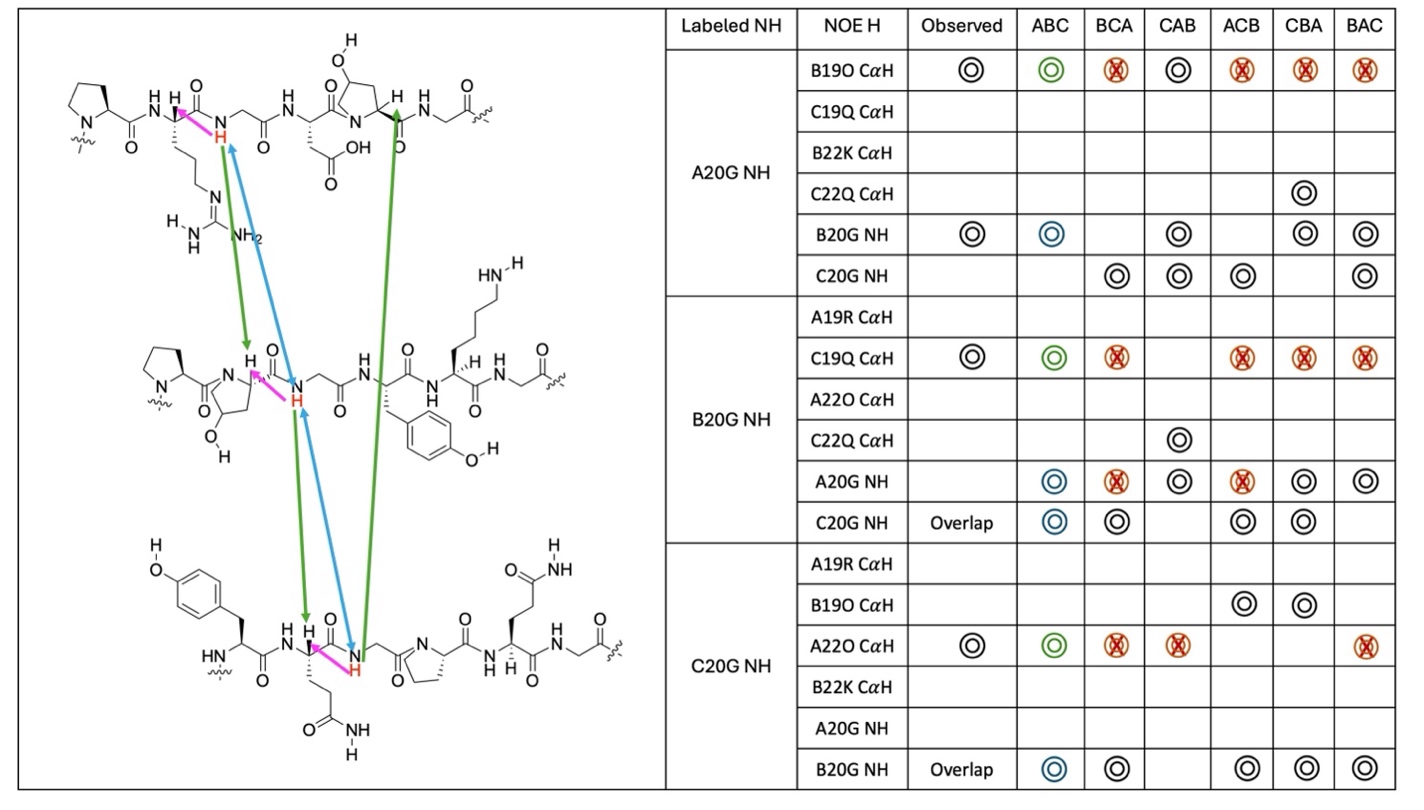


**Figure S39.** ChemDraw figure illustrating the predicted register of heterotrimer ABC-1 and the table of observed and expected inter-strand NOEs of each of six canonical registrations. Colors of arrows in figure correlate with color of peaks in the table. The red, crossed peaks imply a peak that is observed but not expected for the indicated register.


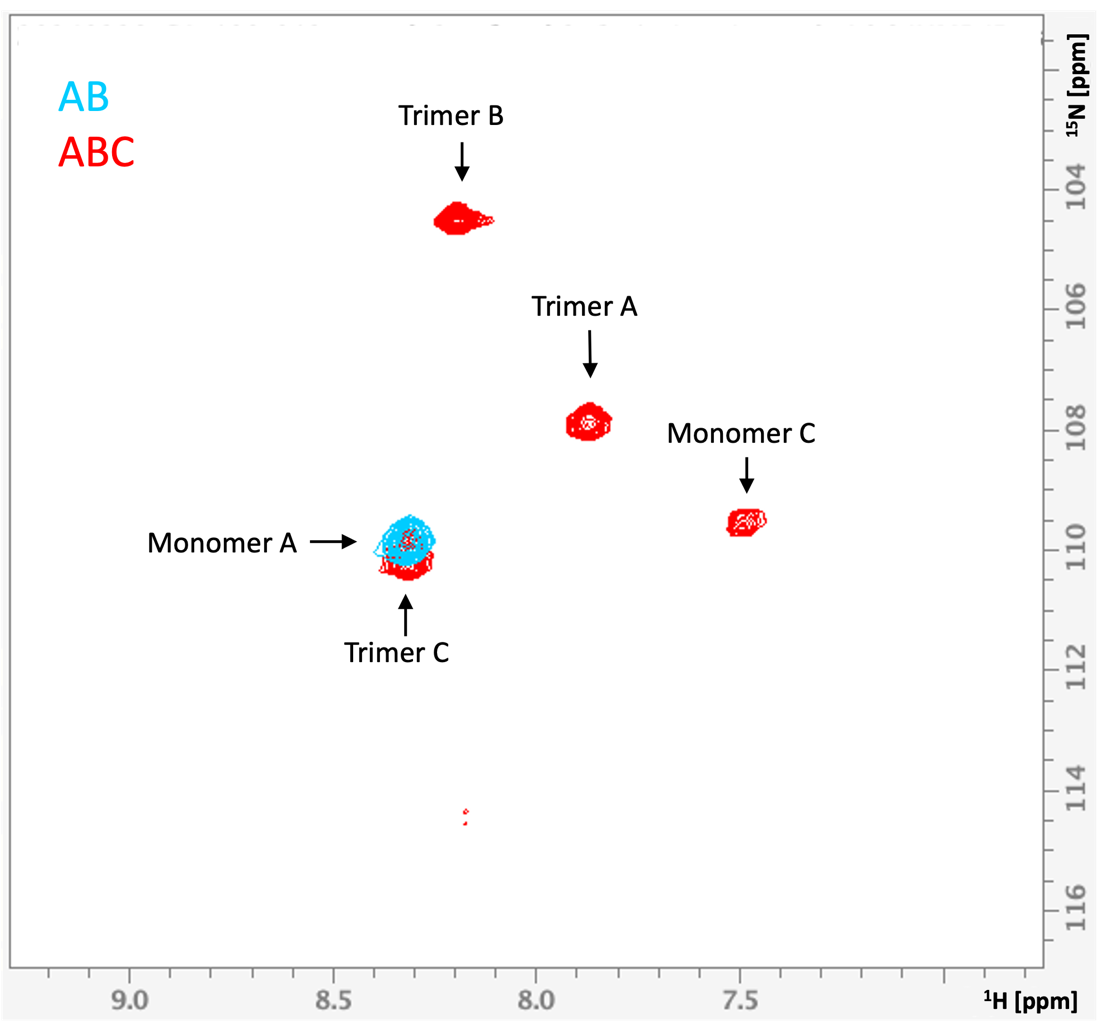


**Figure S40.** Overlaid ^1^H–^15^N HSQC spectra of ABC-1 A:B binary mixture and A:B:C ternary mixture at 25 °C


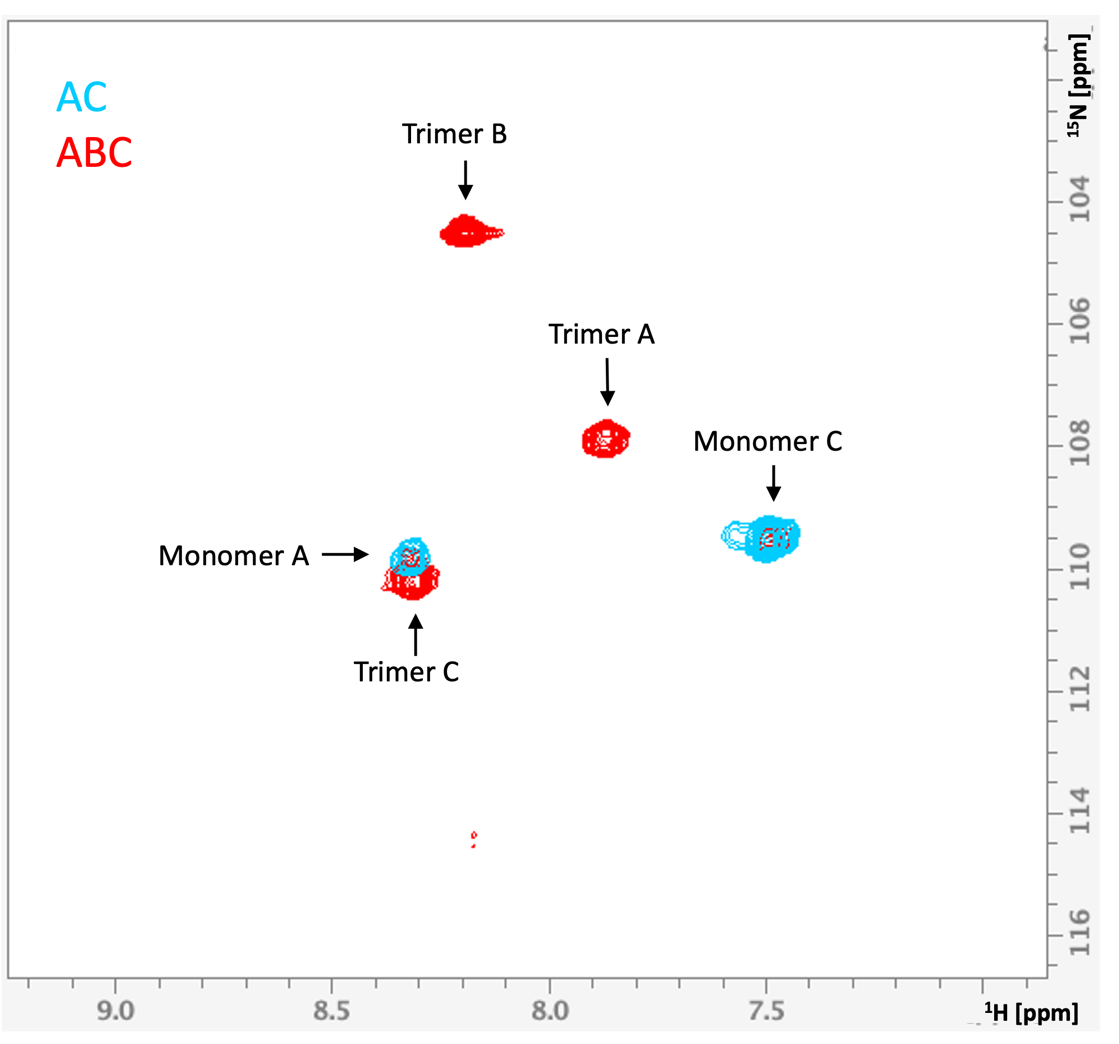


**Figure S41.** Overlaid ^1^H–^15^N HSQC spectra of ABC-1 A:C binary mixture and A: B: C ternary mixture at 25 °C


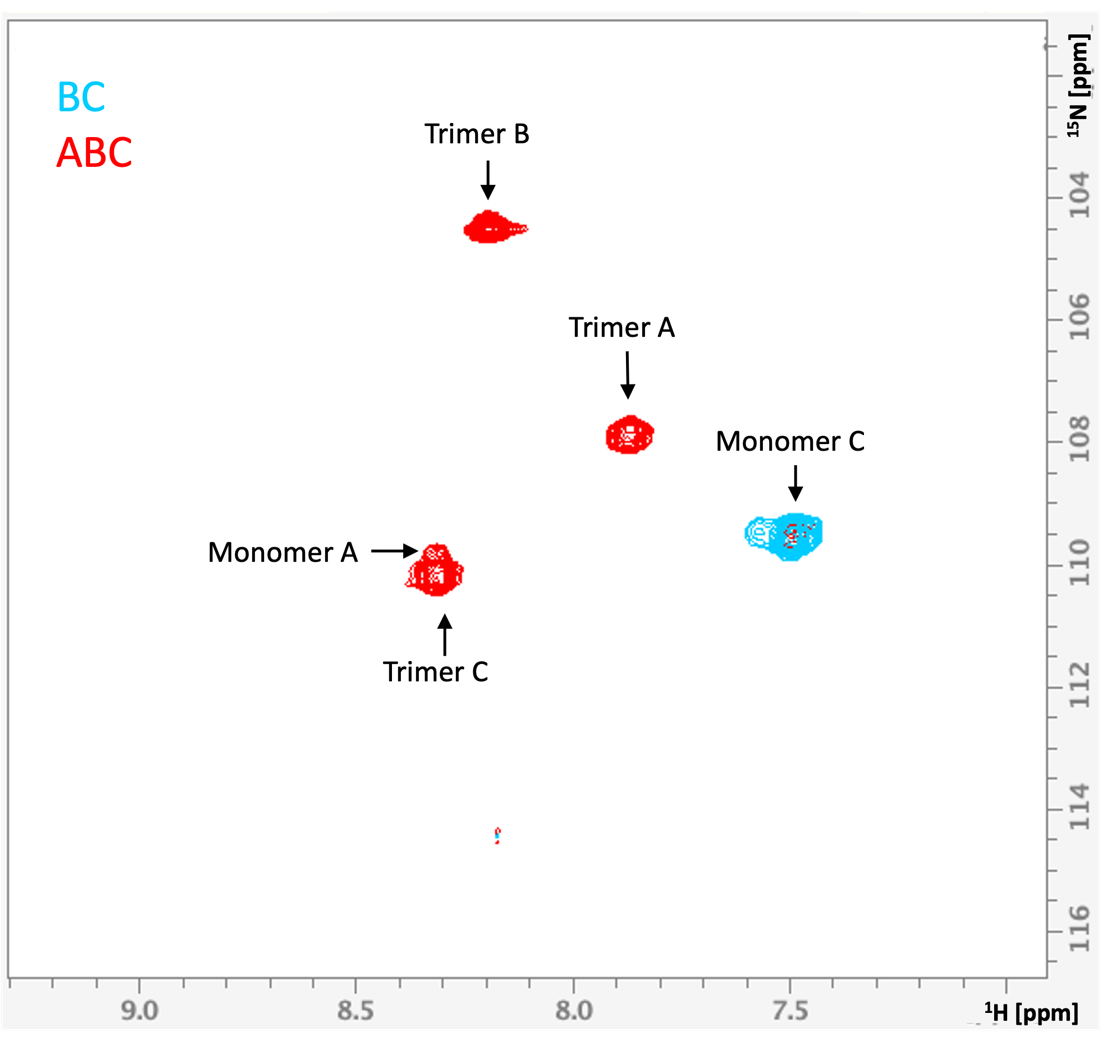


**Figure S42.** Overlaid ^1^H–^15^N HSQC spectra of ABC-1 B:C binary mixture and A: B: C ternary mixture at 25 °C


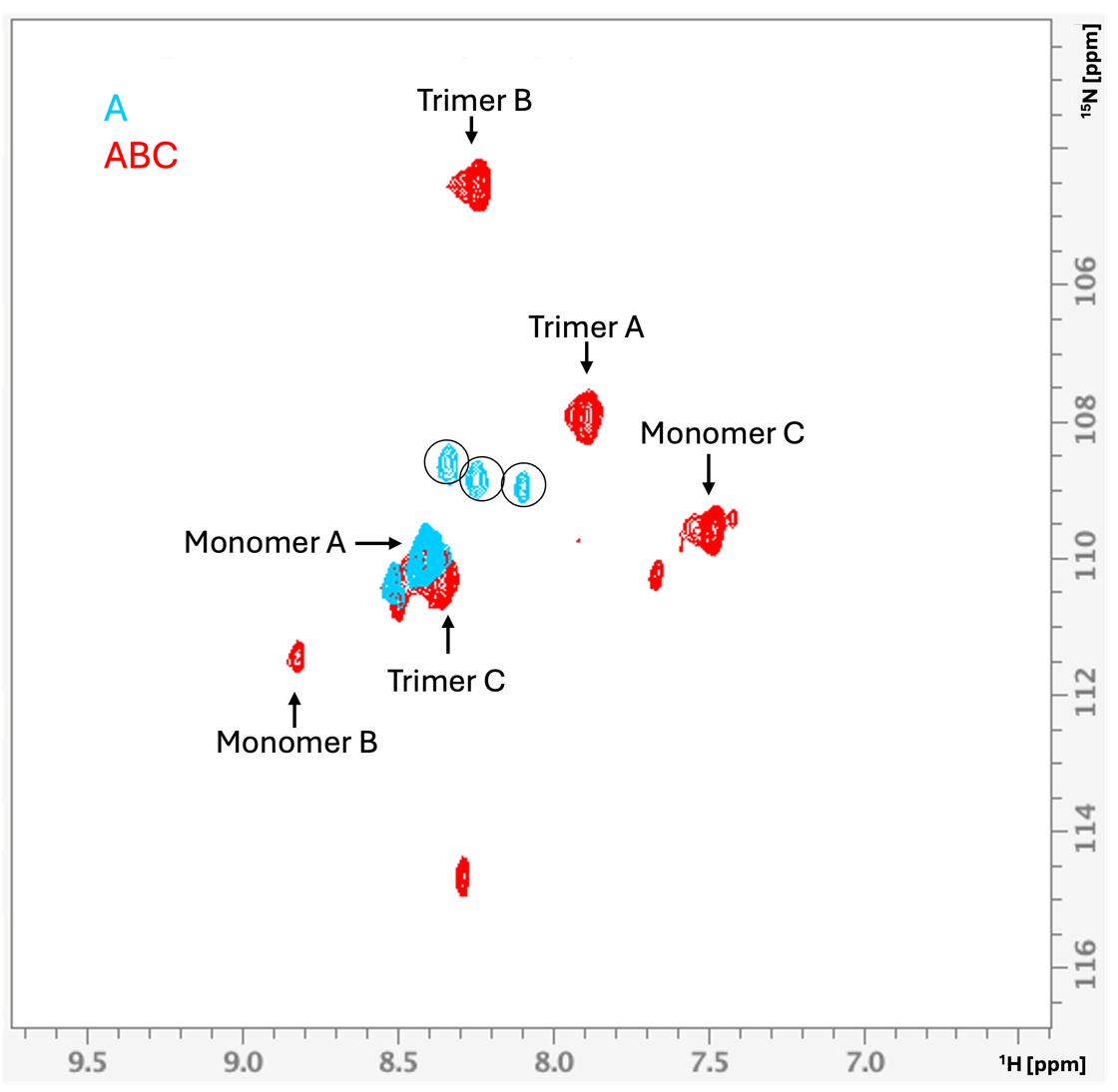


**Figure S43.** Overlaid ^1^H–^15^N HSQC spectra of ABC-1 A unary mixture and A: B: C ternary mixture at 10 °C. Cross peaks marked with solid circles suggest the presence of homotrimer A at 10 °C.


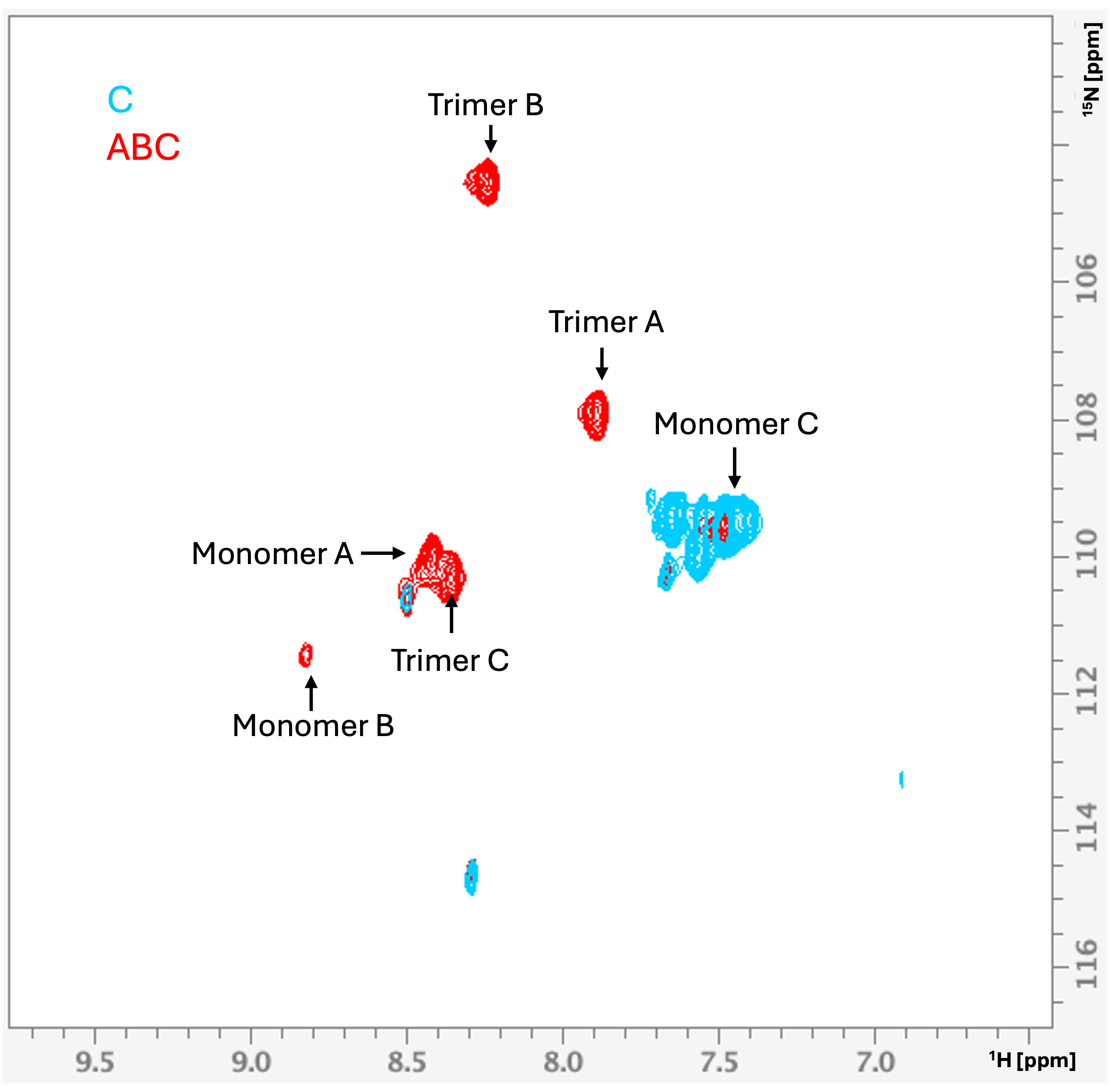


**Figure S44.** Overlaid ^1^H–^15^N HSQC spectra of ABC-1 C unary mixture and A: B: C ternary mixture at 10 °C.


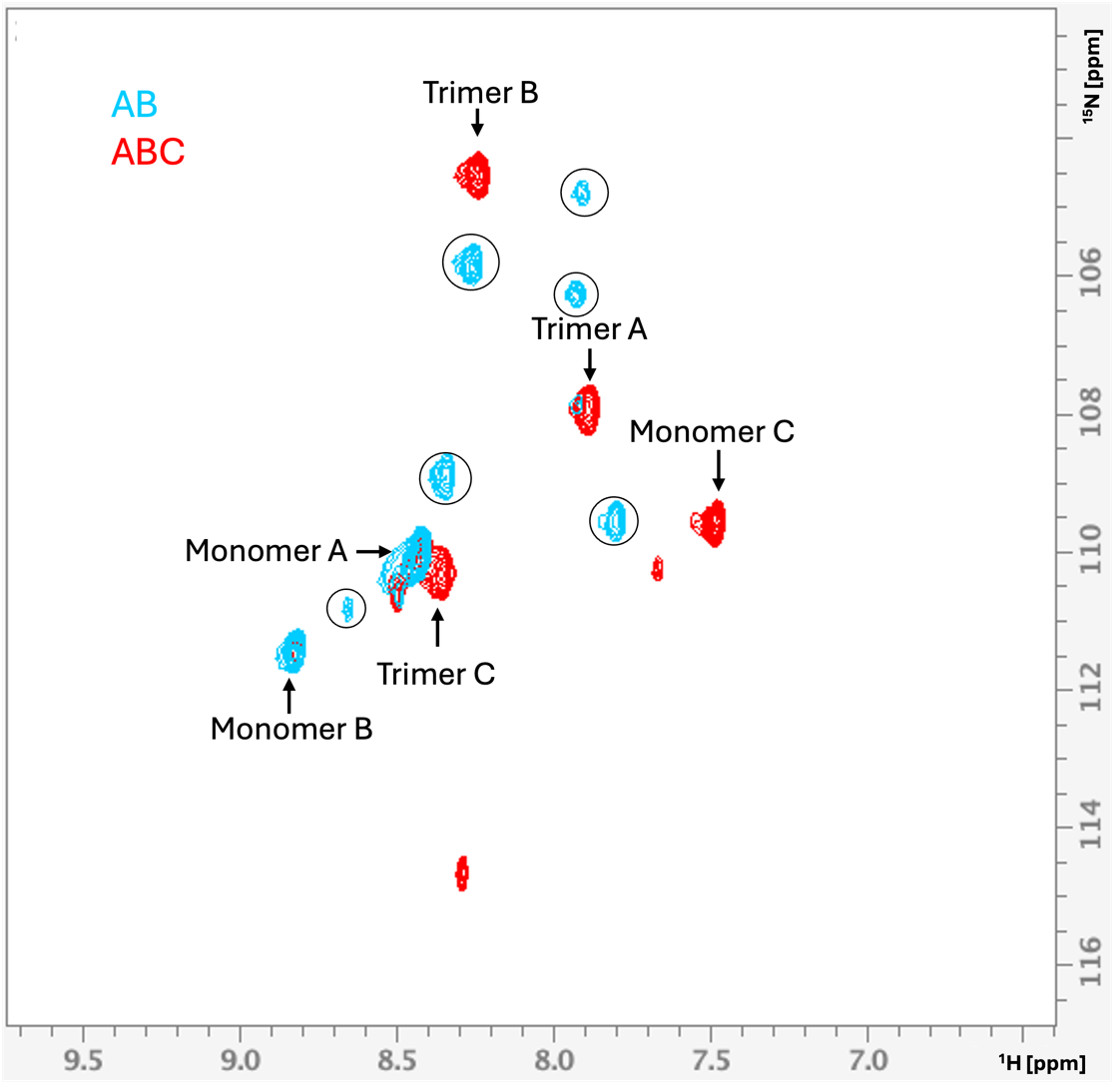


**Figure S45.** Overlaid ^1^H–^15^N HSQC spectra of ABC-1 A: B binary mixture and A: B: C ternary mixture at 10 °C. The circled cross peaks suggest the presence of at least two heterotrimeric registers.


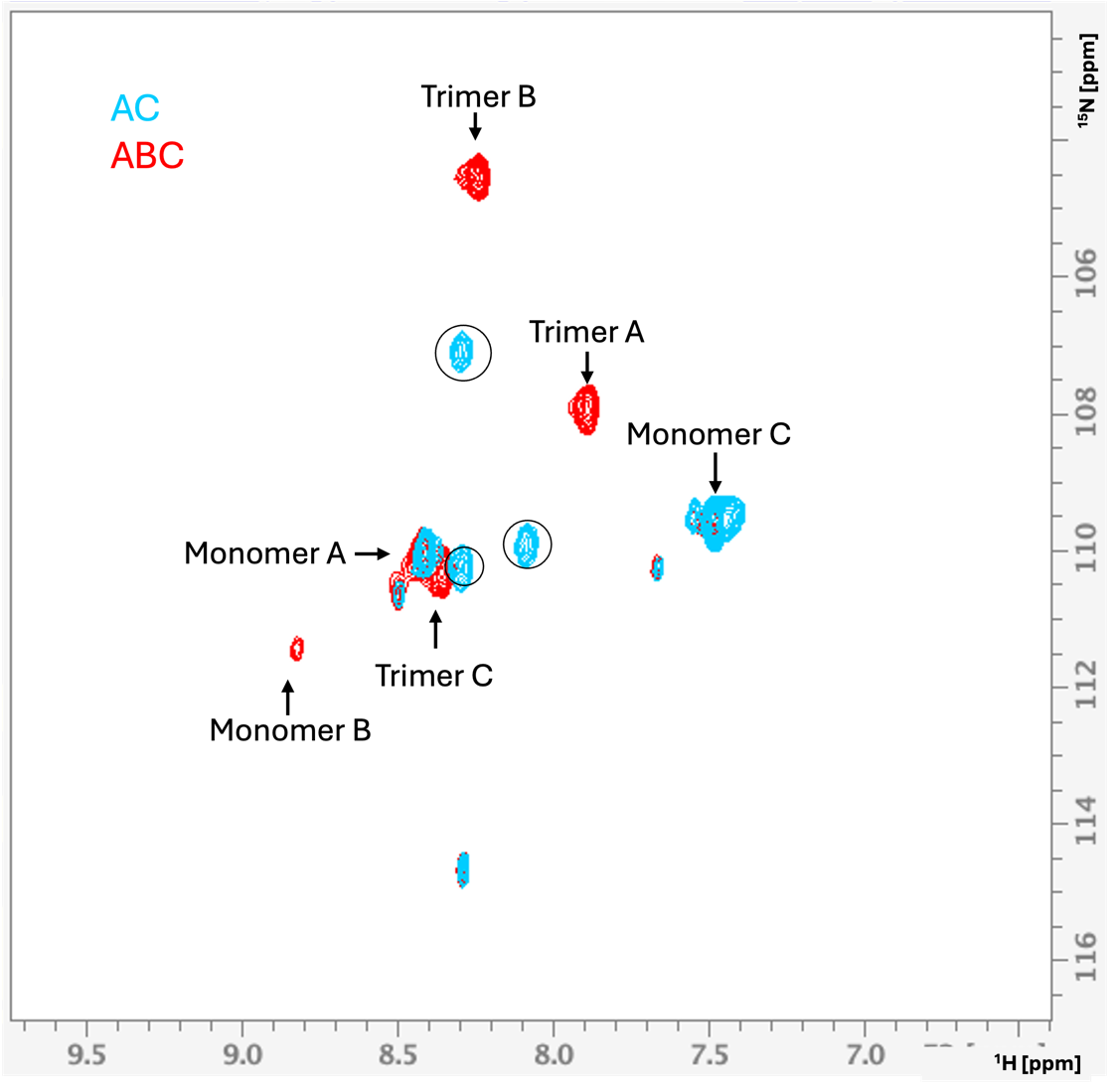


**Figure S46.** Overlaid ^1^H–^15^N HSQC spectra of ABC-1 A: C binary mixture and A: B: C ternary mixture at 10 °C. The circled cross peaks suggest the presence of a heterotrimer.


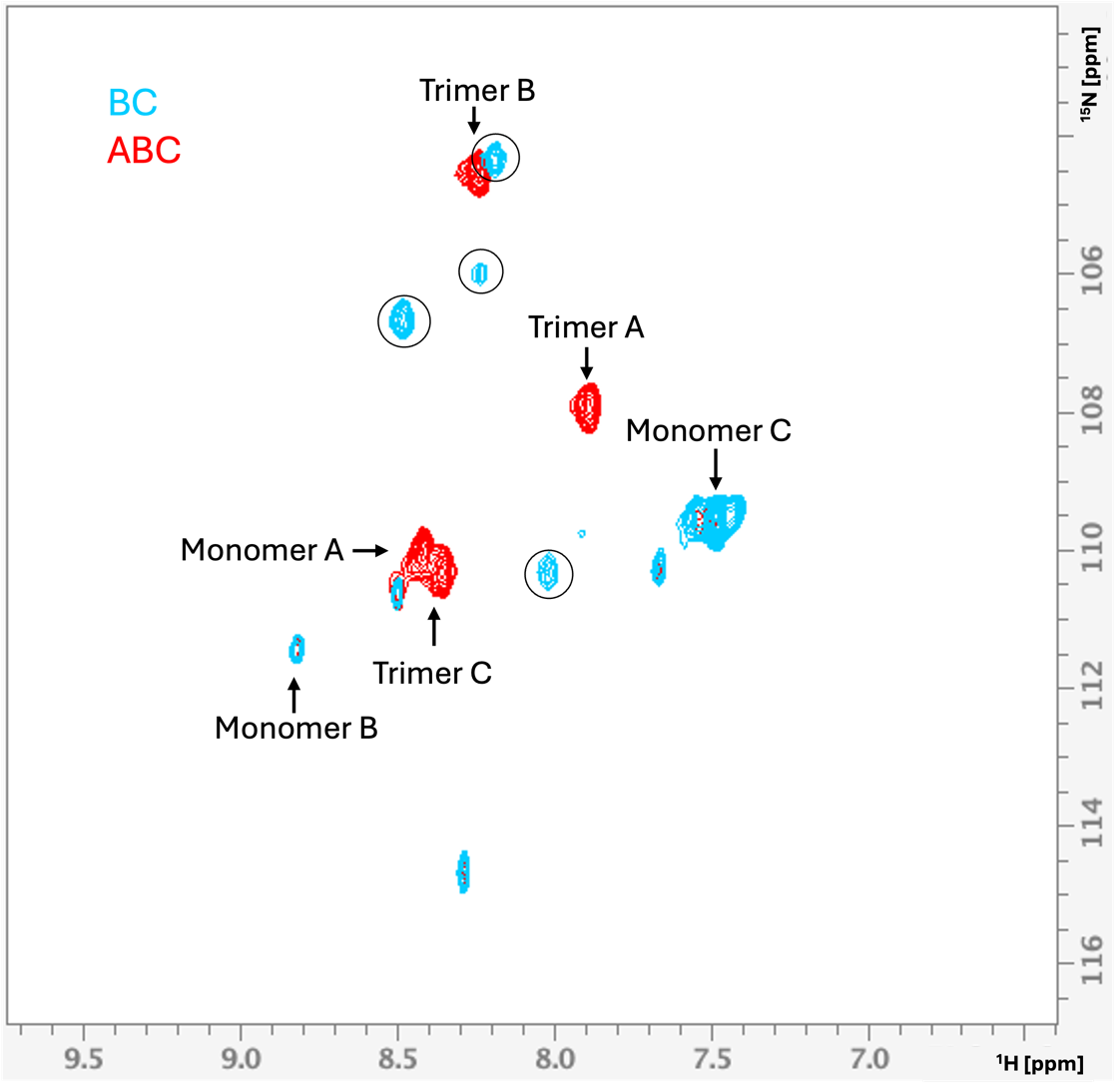


**Figure S47.** Overlaid ^1^H–^15^N HSQC spectra of ABC-1 B: C binary mixture and A: B: C ternary mixture at 10 °C. The circled cross peaks suggest the presence of heterotrimers of different registers.

## **7.3. ABC-2**

**Table S26.** NOESY-HSQC peak assignments of heterotrimer ABC-2

| Plane at ^15^N chemical shift of 106.25 ppm – peptide A – leading strand | |
| --- | --- |
| 1.60 ppm | NOE to Lys19 LCβ-H on peptide A |
| 2.17 and 1.97 ppm | NOEs to Hyp16 Cβ-H |
| 3.09 ppm | NOE to Hyp16 Cδ-H |
| 3.59 ppm | NOE to labeled Gly17 C𝛼-H |
| 4.16 ppm | NOE to Lys16 C𝛼-H on peptide B |
| 4.38 ppm | NOE to Asp15 C𝛼-H on peptide B |
| 4.81 ppm | NOE to Hyp16 C𝛼-H |
| 7.74 ppm | NOE to Gly14 NH on peptide C |
| 8.07 ppm | NOE to labeled Gly17 NH on peptide B |
| 8.28 ppm | NOE to labeled Gly17 NH |
| Plane at ^15^N chemical shift of 110.31 ppm – peptide B – middle strand | |
| 1.10 ppm | NOE to Lys16 Cγ-H |
| 1.60 ppm | NOE to Lys16 Cβ-H |
| 3.08 ppm | NOE to Lys16 Cε-H |
| 3.48 and 3.69 ppm | NOEs to the labeled Gly17 Cα-H |
| 4.16 ppm | NOE to the Lys16 Cα-H |
| 4.39 ppm | NOE to Asp16 Cα-H on peptide C |
| 8.07 ppm | NOE to labeled Gly17 NH |
| 8.27 ppm | NOE to labeled Gly17 NH on peptide A |
| 8.57 ppm | NOE to labeled Gly17 NH on peptide C |
| Plane at ^15^N chemical shift of 104.06 ppm – peptide C – trailing strand | |
| 1.60 ppm | NOE to Lys16 Cβ-H on peptide B |
| 2.49 ppm | NOE to Asp16 Cβ-H |
| 3.11 ppm | NOE to Lys16 Cε-H on peptide B |
| 3.50 and 3.70 ppm | NOEs to labeled Gly17 Cα-H |
| 4.16 ppm | NOE to Lys16 Cα-H on peptide B |
| 4.39 ppm | NOE to Asp16 Cα-H |
| 7.89 ppm | NOE to Gly20 NH on peptide A |
| 8.07 ppm | NOE to labeled Gly17 NH on peptide B |
| 8.28 ppm | NOE to labeled Gly17 NH on peptide A |
| 8.52 ppm | NOE to labeled Gly17 NH |


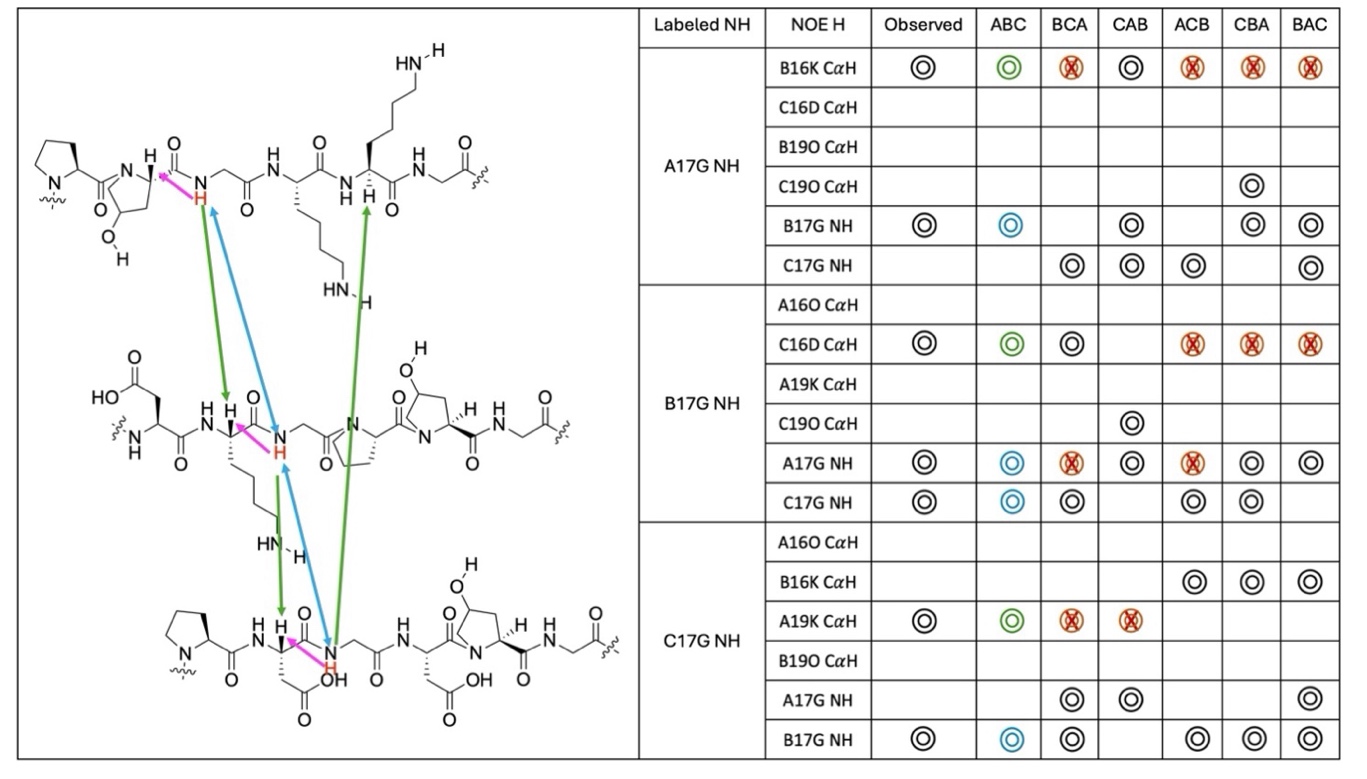


**Figure S48.** ChemDraw figure illustrating the predicted register of heterotrimer ABC-2 and the table of observed and expected inter-strand NOEs of each of six canonical registrations. Colors of arrows in figure correlate with color of peaks in the table. The red, crossed peaks imply a peak that is observed but not expected for the indicated register.


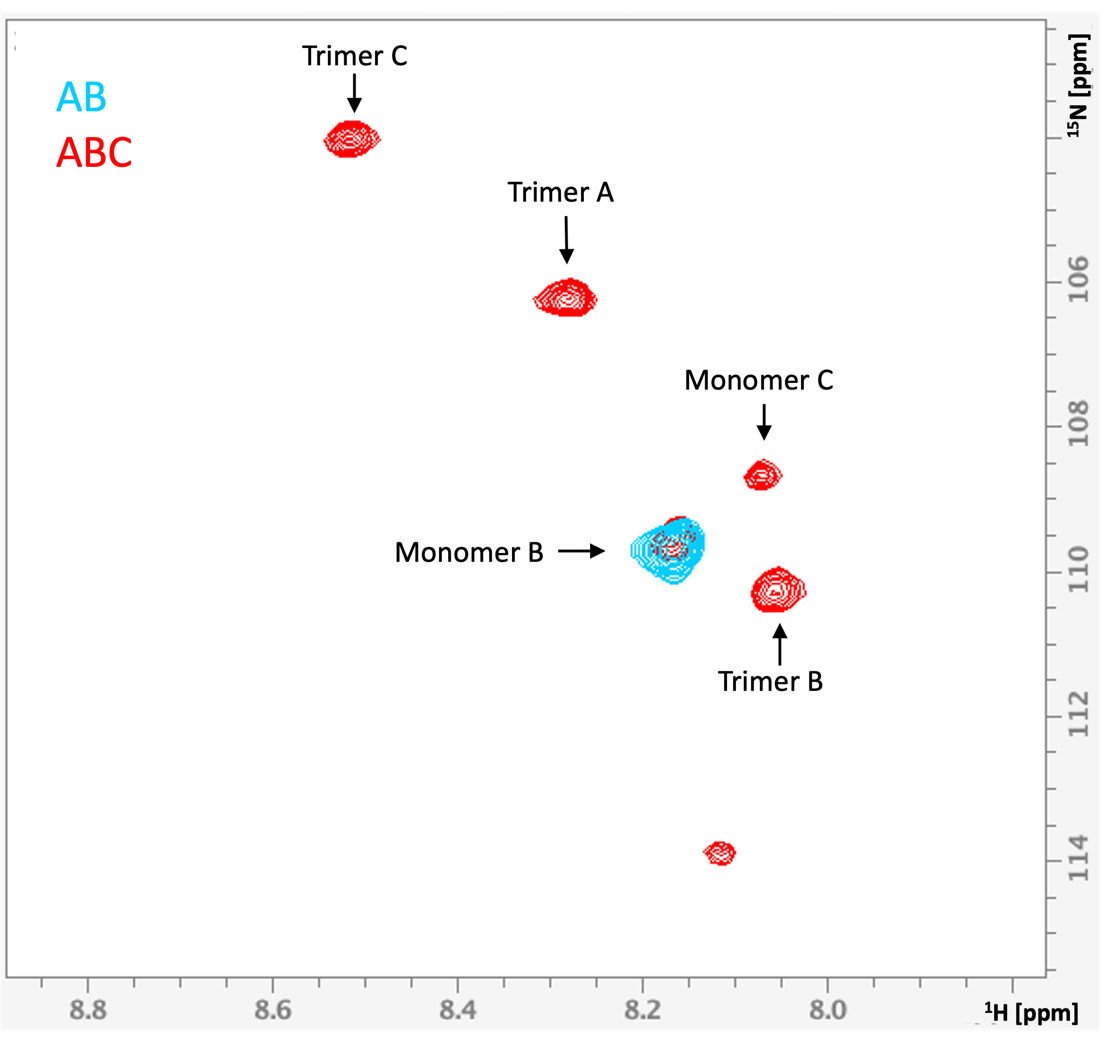


**Figure S49.** Overlaid ^1^H–^15^N HSQC spectra of ABC-2 A:B binary mixture and A:B:C ternary mixture at 25 °C.


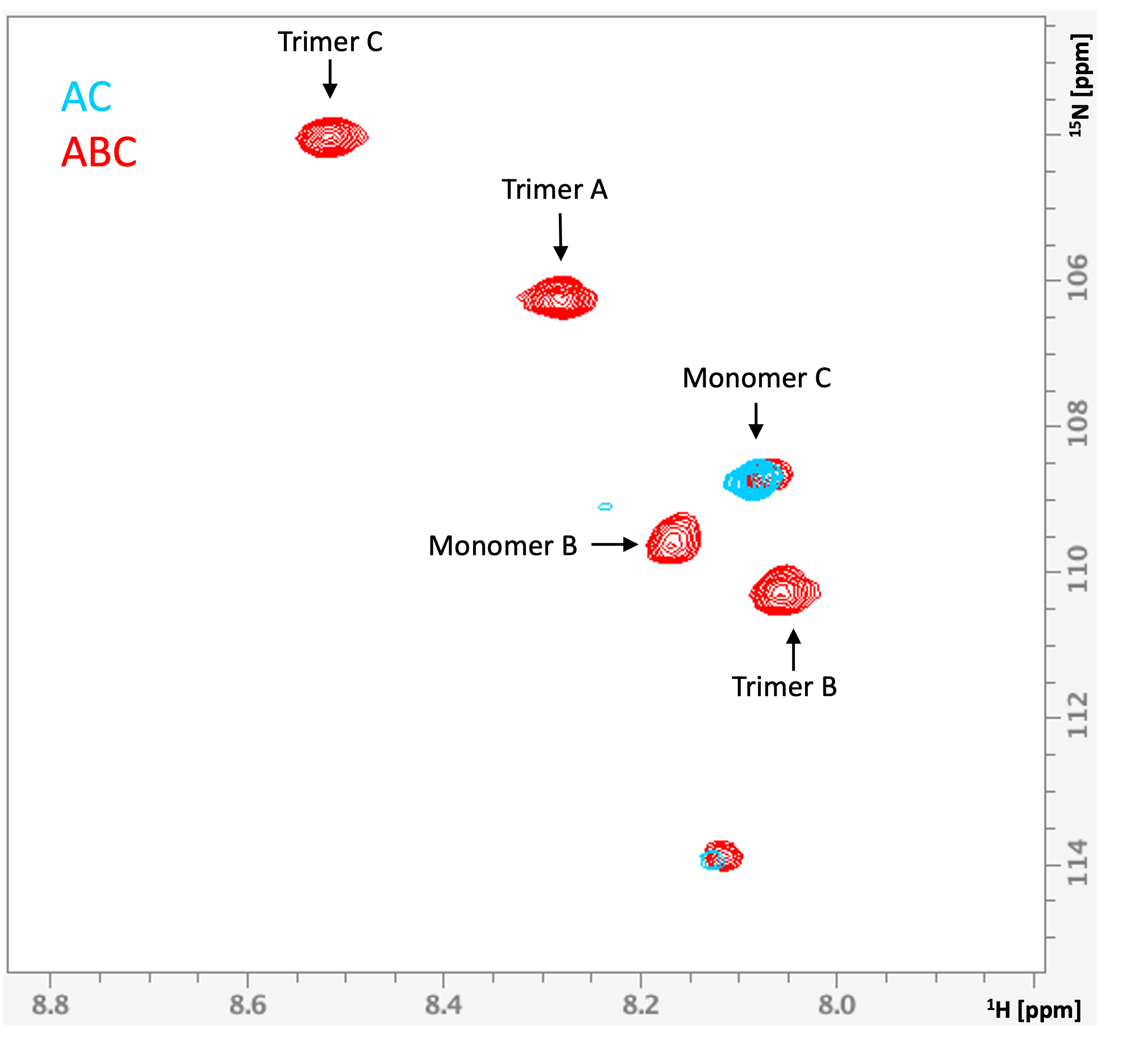


**Figure S50.** Overlaid ^1^H–^15^N HSQC spectra of ABC-2 A:C binary mixture and A: B: C ternary mixture at 25 °C.


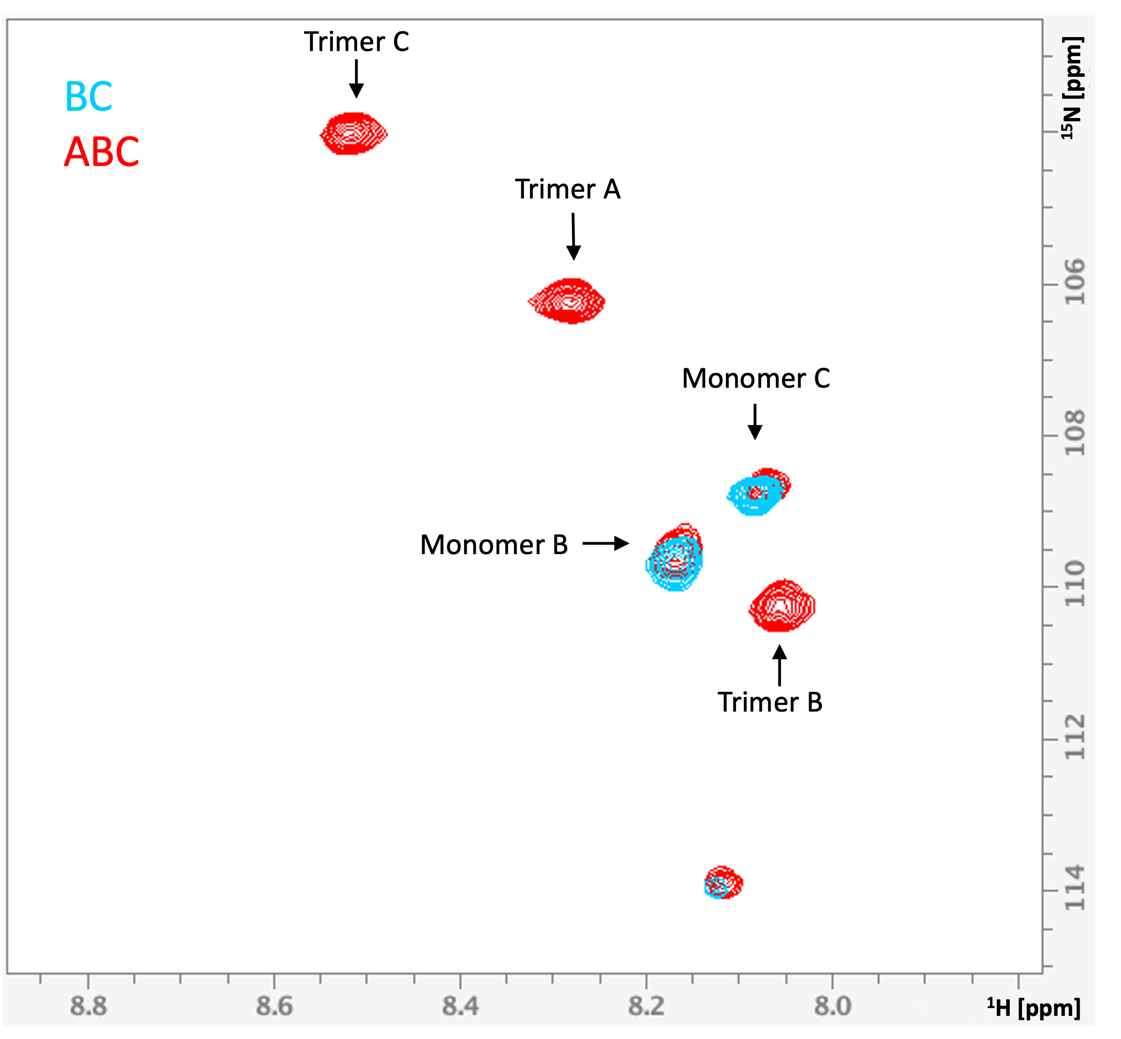


**Figure S51.** Overlaid ^1^H–^15^N HSQC spectra of ABC-2 B:C binary mixture and A: B: C ternary mixture at 25 °C.

## **7.4. AAB-FOGER**

**Table S27.** NOESY-HSQC peak assignments of heterotrimer AAB-FOGER

| Plane at ^15^N chemical shift of 108.67 ppm – peptide A – leading strand | |
| --- | --- |
| 1.19 ppm | NOE to Lys20 Cγ-H |
| 1.70 ppm | NOE to Lys20 Cβ-H |
| 2.31 ppm | NOE to Pro19 Cβ-H |
| 2.77 ppm | NOE to Asp19 Cβ-H on middle strand |
| 3.03 ppm | NOE to Lys20 Cε-H |
| 3.63 and 3.84 ppm | NOEs to labeled Gly21 Cα-H |
| 4.13 ppm | NOE to Lys20 Cα-H on middle strand |
| 4.92 ppm | NOE to Lys20 Cα-H |
| 8.02 ppm | NOE to labeled Gly21 NH on middle strand |
| 8.53 ppm | NOE to labeled Gly21 NH |
| Plane at ^15^N chemical shift of 108.2 ppm – peptide A – middle strand | |
| 1.21 ppm | NOE to Lys20 Cγ-H |
| 1.69 ppm | NOE to Lys20 Cβ-H |
| 2.27 ppm | NOE to Pro19 Cβ-H |
| 3.02 ppm | NOE to Lys20 Cε-H |
| 3.28 and 3.60 ppm | NOEs to Gly21 C𝛼-H |
| 4.14 ppm | NOE to Lys20 C𝛼-H |
| 4.94 ppm | NOE to Hyp20 C𝛼-H on peptide B |
| 8.02 ppm | NOE to labeled Gly21 NH |
| 8.49 ppm | NOE to labeled Gly21 NH on leading strand |
| 8.80 ppm | NOE to Asp22 NH on leading strand |
| Plane at ^15^N chemical shift of 104.45 ppm – peptide B – trailing strand | |
| 1.20 ppm | NOE to Lys20 C𝛾-H on middle strand |
| 1.68 ppm | NOE to Lys20 Cβ-H on middle strand |
| 2.07 and 2.25 ppm | NOEs to Hyp20 Cβ-H |
| 3.02 ppm | NOEs to Lys20 Cε-H on middle strand |
| 3.59 and 3.80 ppm | NOE to labeled Gly21 C𝛼-H |
| 4.93 ppm | NOE to Hyp20 C𝛼-H |
| 8.13 ppm | NOE to Gly21 NH overlap with NOE to Gly21 on middle strand |
| 8.46 ppm | NOE to Gly24 NH on leading strand |

## **7.5. ABC-FOGER**

**Table S28.** NOESY-HSQC peak assignments of heterotrimer ABC-FOGER

| Plane at ^15^N chemical shift of 107.97 ppm (left) – peptide A – leading strand | |
| --- | --- |
| 1.94 and 2.19 ppm | NOEs to Hyp17 Cβ-H |
| 3.18 ppm | NOEs to Lys17 Cε-H on peptide B |
| 3.51 and 3.81 ppm | NOE to labeled Gly17 C𝛼-H |
| 4.04 ppm | NOE to Arg17 C𝛼-H on peptide C |
| 4.33 ppm | NOE to Lys17 C𝛼-H on peptide B |
| 4.75 ppm | NOE to Hyp17 C𝛼-H |
| 8.07 ppm | NOE to Gly15 NH on peptide C |
| 8.28 ppm | NOE to labeled Gly18 NH overlap with Gly17 NH on peptide B |
| Plane at ^15^N chemical shift of 110.69 ppm – peptide B – middle strand | |
| 1.12 ppm | NOE to Lys17 Cγ-H |
| 1.57 ppm | NOE to Lys17 Cβ-H |
| 2.91 and 3.15 ppm | NOEs to Lys17 Cε-H |
| 3.51 and 3.80 ppm | NOEs to the labeled Gly17 |
| 4.08 ppm | C𝛼-H NOE to Arg17 C𝛼-H on peptide C |
| 4.31 ppm | NOE to Lys17 C𝛼-H |
| 8.04 ppm | NOE to labeled Gly17 NH on peptide C |
| 8.36 ppm | NOE to labeled Gly17 NH overlap with Gly17 NH on peptide A |
| Plane at ^15^N chemical shift of 107.97 ppm (right) – peptide C – trailing strand | |
| 1.19 ppm | NOE to Lys17 Cγ-H on peptide B |
| 1.39 ppm | NOE to Arg17 Cγ-H |
| 1.64 and 1.89 ppm | NOEs to Arg17 Cβ-H |
| 3.13 ppm | NOE to Arg17 Cδ-H |
| 3.54 and 3.79 ppm | NOE to labeled Gly18 C𝛼-H |
| 4.03 ppm | NOE to Arg17 C𝛼-H |
| 4.32 ppm | NOE to Lys17 C𝛼-H on peptide B |
| 4.70 ppm | NOE to Hyp20 C𝛼-H on peptide A |
| 7.80 ppm | NOE to Arg17 Nε-H |
| 8.03 ppm | NOE to labeled Gly17 NH |
| 8.43 ppm | NOE to Gly21 NH on peptide A |

**Figure S52.** ChemDraw figure illustrating the predicted register of heterotrimer ABC-FOGER and the table of observed and expected inter-strand NOEs of each of six canonical registrations. Colors of arrows in figure correlate with color of peaks in the table. The red, crossed peaks imply a peak that is observed but not expected for the indicated register.


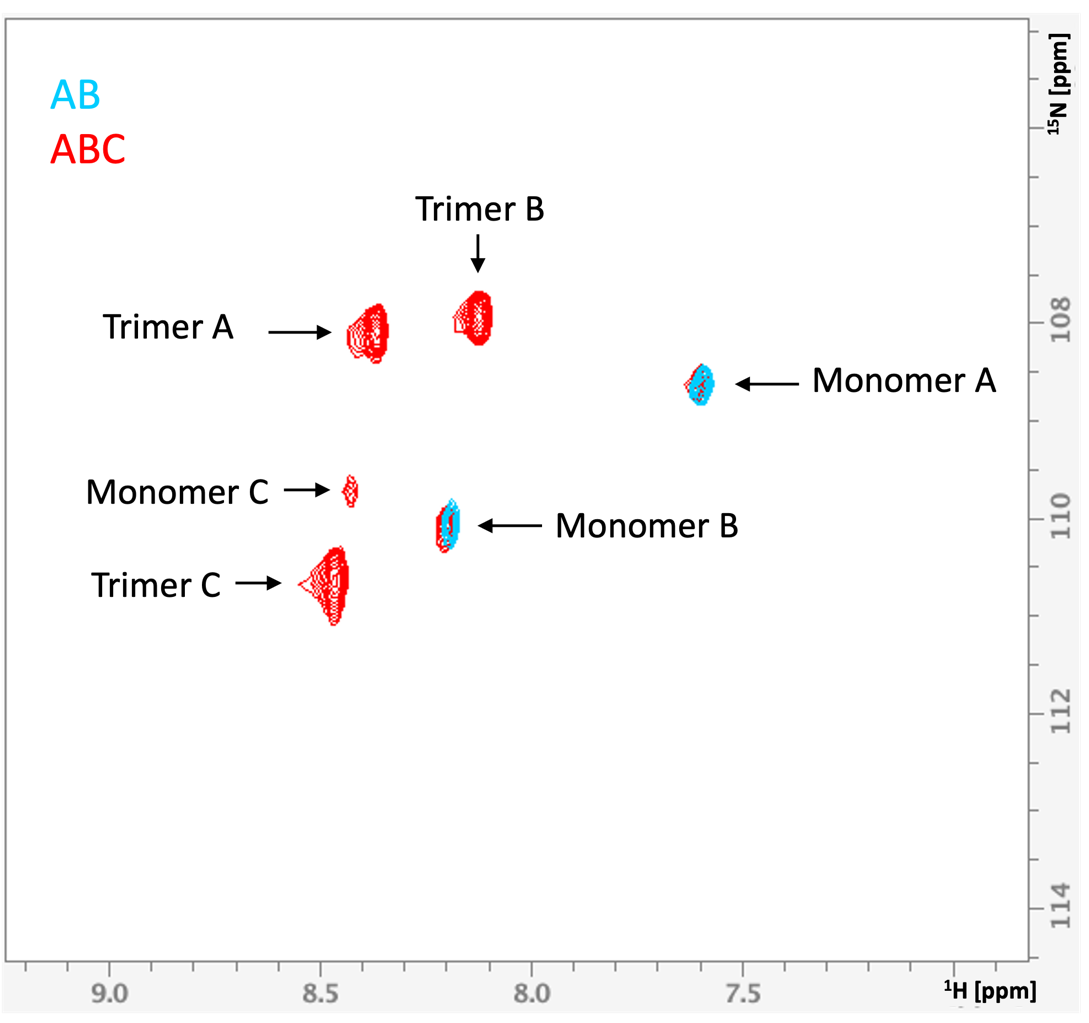


**Figure S53.** Overlaid ^1^H–^15^N HSQC spectra of ABC-FOGER A:B binary mixture and A:B:C ternary mixture at 25 °C.


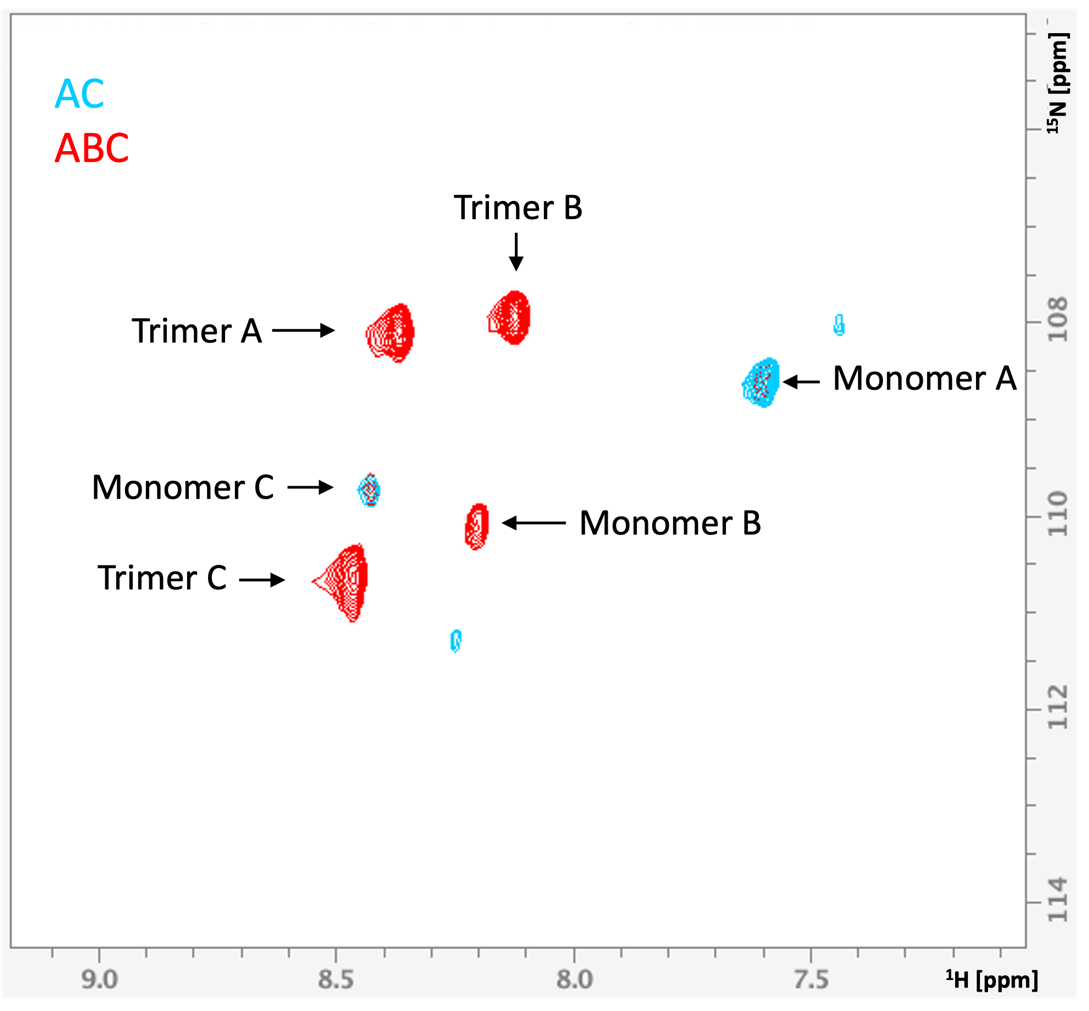


**Figure S54.** Overlaid ^1^H–^15^N HSQC spectra of ABC-FOGER A:C binary mixture and A:B:C ternary mixture at 25°C.


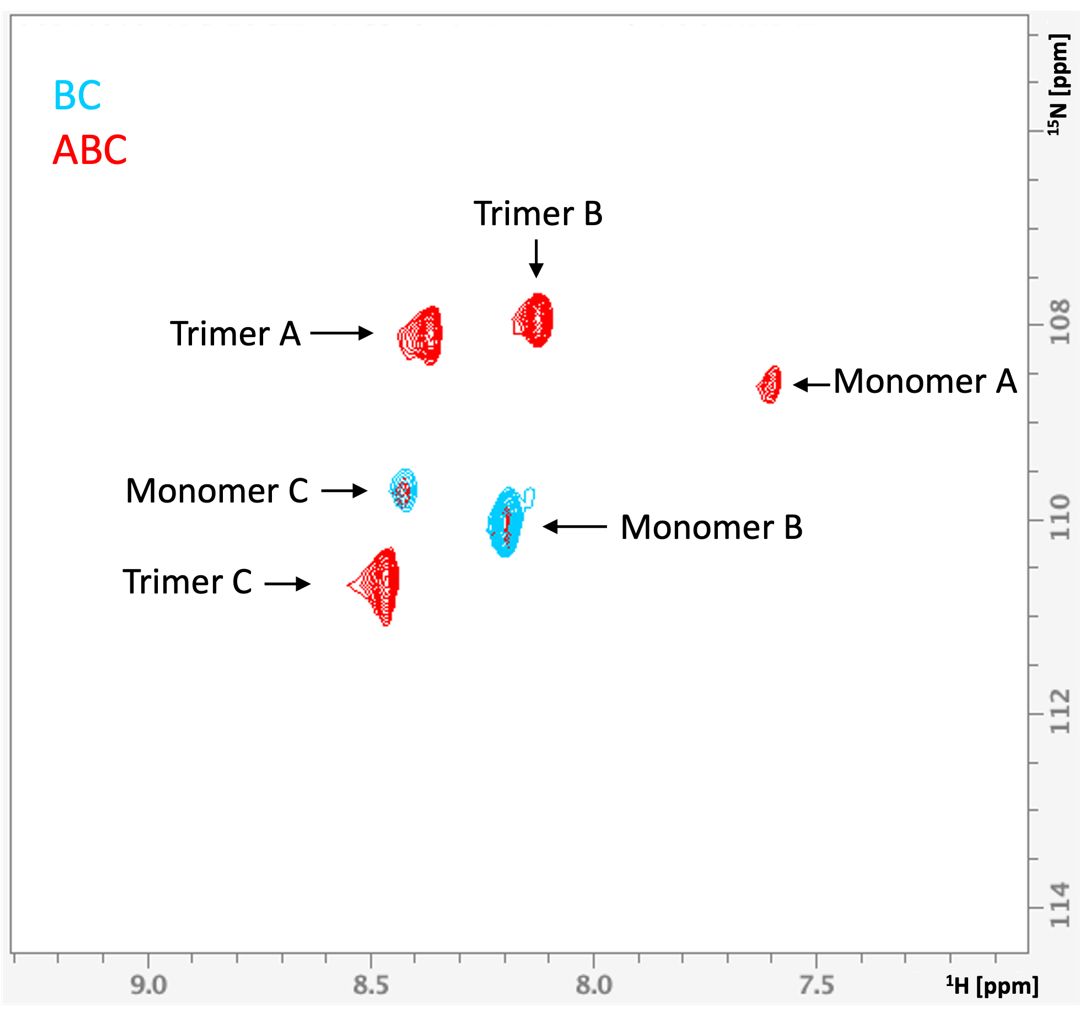


**Figure S55.** Overlaid ^1^H–^15^N HSQC spectra of ABC-FOGER B:C binary mixture and A:B:C ternary mixture at 25 °C.


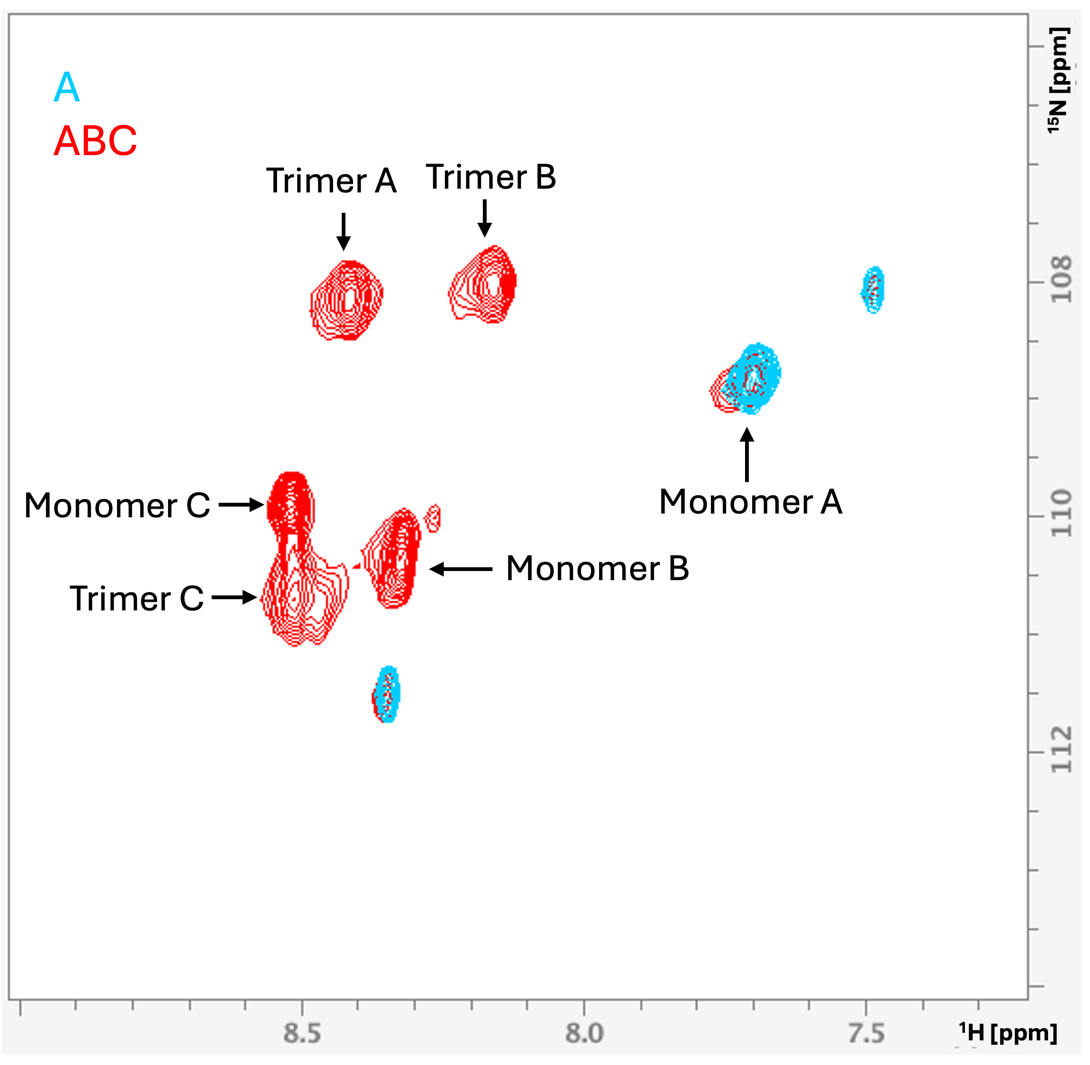


**Figure S56.** Overlaid ^1^H–^15^N HSQC spectra of ABC-FOGER A unary mixture and A:B:C ternary mixture at 10°C.


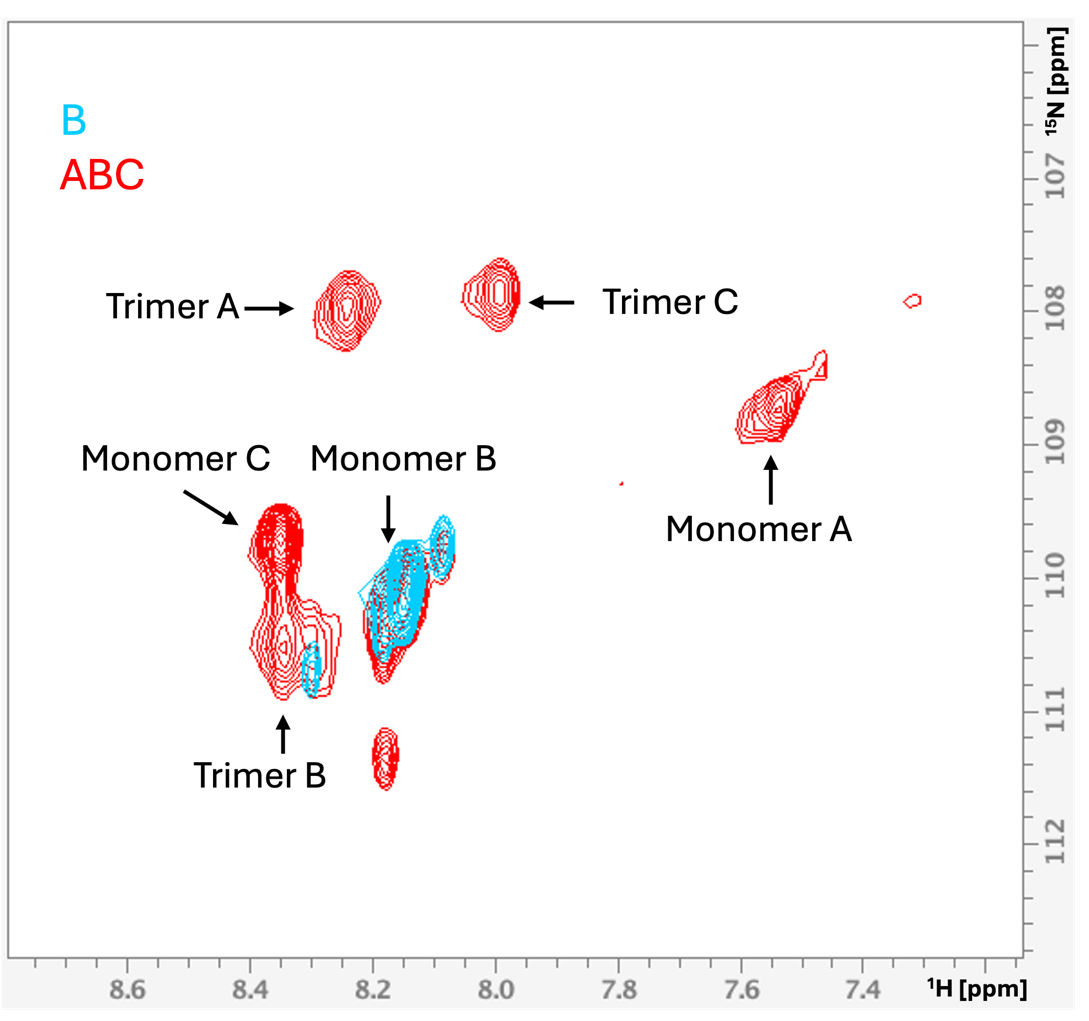


**Figure S57.** Overlaid ^1^H–^15^N HSQC spectra of ABC-FOGER B unary mixture and A:B:C ternary mixture at 10°C.


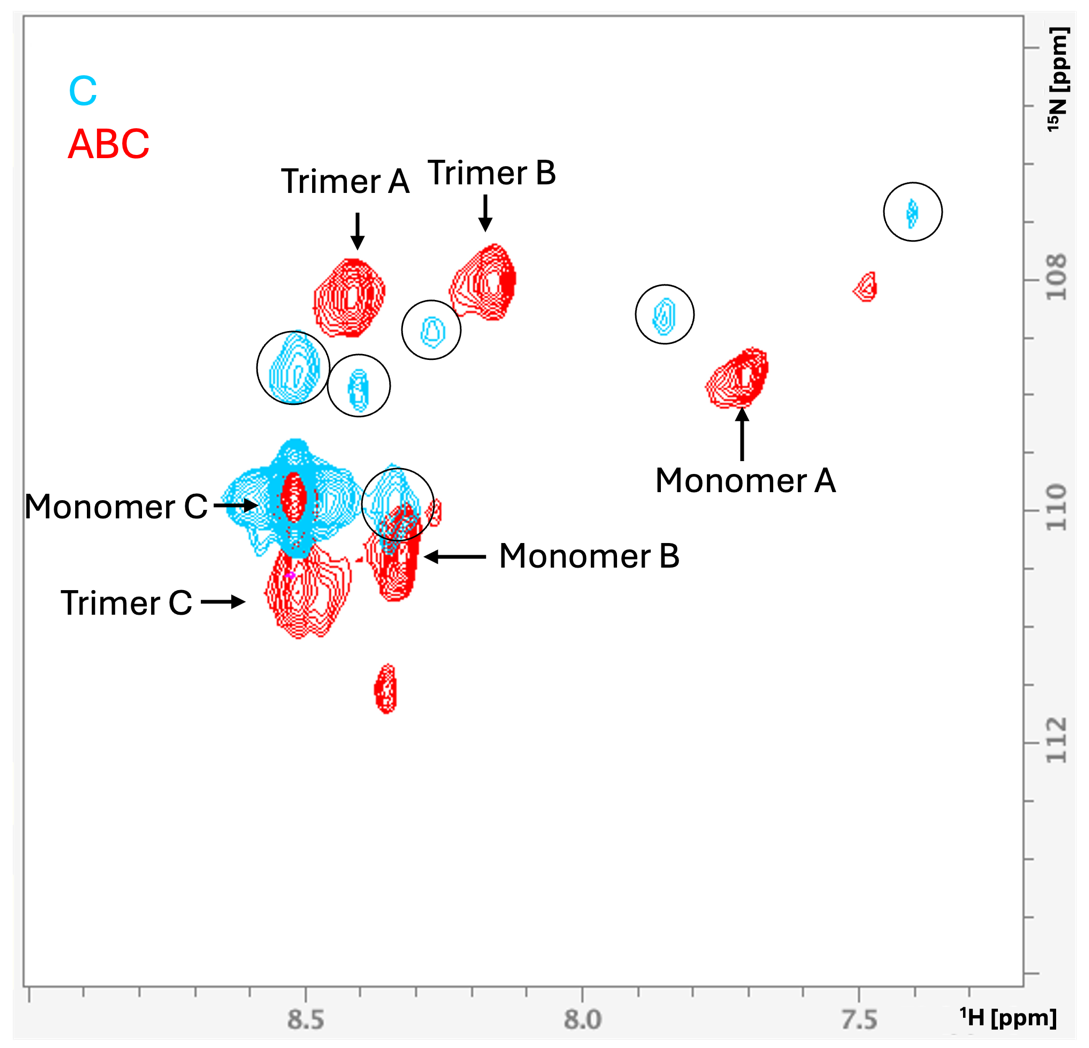


**Figure S58.** Overlaid ^1^H–^15^N HSQC spectra of ABC-FOGER C unary mixture and A:B:C ternary mixture at 10°C. Six circled cross peaks suggest the presence of additional homotrimers of different registers.


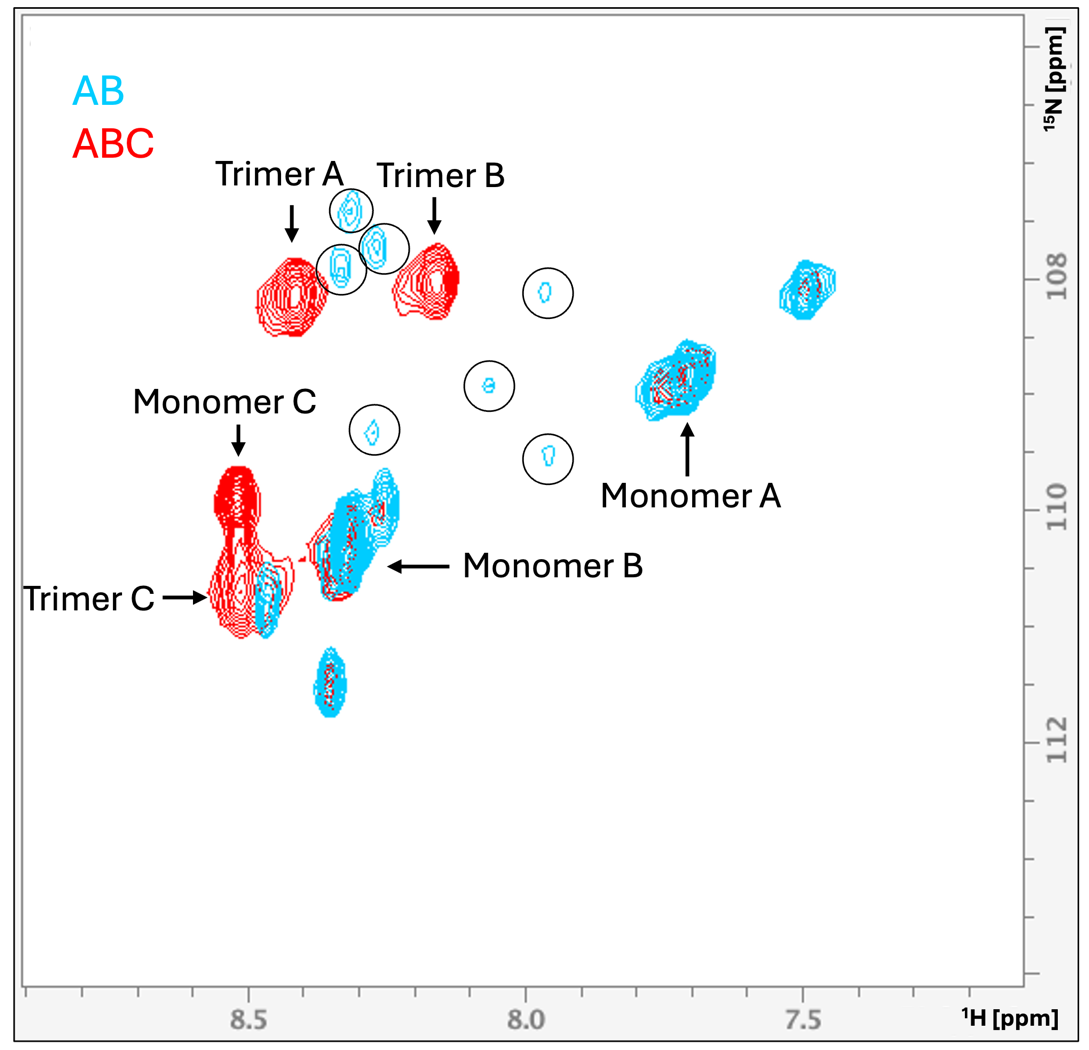


**Figure S59.** Overlaid ^1^H–^15^N HSQC spectra of ABC-FOGER A: B binary mixture and A:B:C ternary mixture at 10°C. Seven circled cross peaks suggest the presence of heterotrimers of different registers.


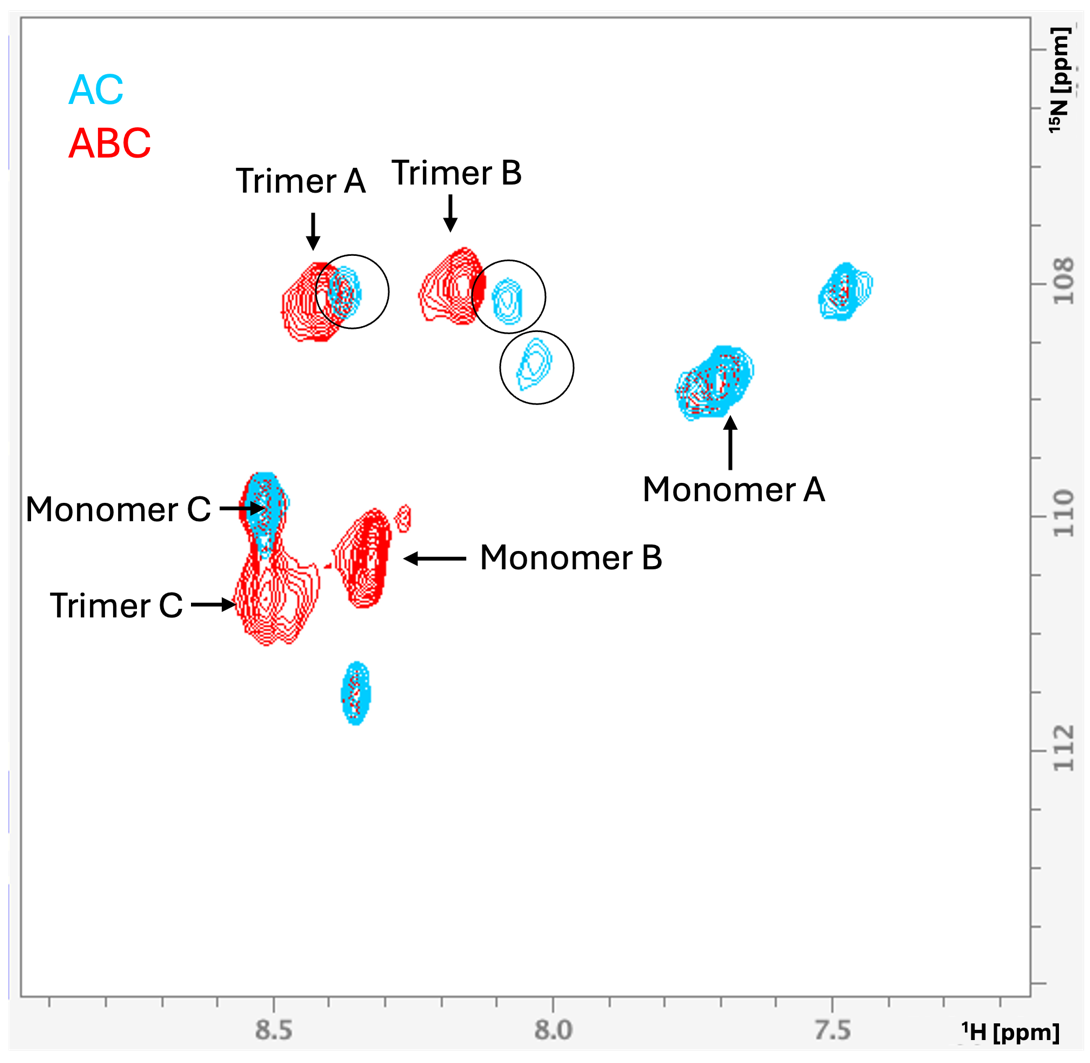


**Figure S60.** Overlaid ^1^H–^15^N HSQC spectra of ABC-FOGER A: C binary mixture and A:B:C ternary mixture at 10°C. Three circled cross peaks suggest the presence of an heterotrimer.


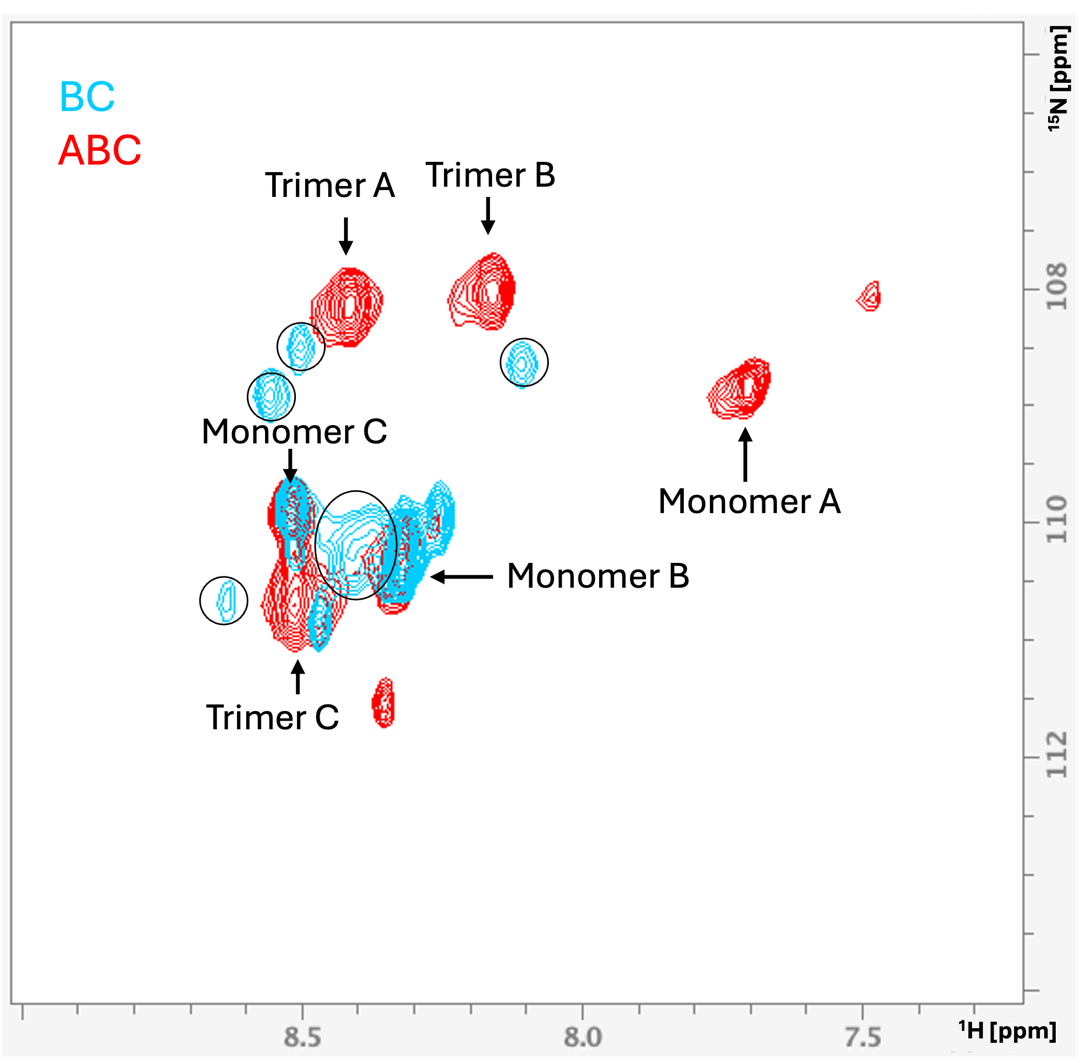


**Figure S61.** Overlaid ^1^H–^15^N HSQC spectra of ABC-FOGER B: C binary mixture and A:B:C ternary mixture at 10°C. Four circled cross peaks and circled overlaying peaks suggest the presence of heterotrimers of different registers.

# **8. Three-dimensional models of GRACE-generated heterotrimers by AlphaFold3**

**Table S29.** 3D structure predictions by AlphaFold3 with various input orders

^1^ - inter-chain predicted Template Modeling: the alignment confidence of predicted structures and interactions between multiple chains of a protein complexes, with scores closer to 1 indicating higher accuracy.

^2^ - predicted Template Modeling: the alignment confidence of 's predicted structure with its potential true form of a single protein chain, with scores closer to 1 indicating higher accuracy.

| Peptide | Trial | Run | Input | TripleHelix  (Y/N) | Predicted Register | ipTM^1^ | pTM^2^ |
| --- | --- | --- | --- | --- | --- | --- | --- |
| ABC-1 | 1 | 1 | ABC | Y | ABC | 0.9 | 0.9 |
| ABC-1 | 1 | 2 | ACB | Y | BAC | 0.89 | 0.88 |
| ABC-1 | 1 | 3 | BAC | Y | BCA | 0.9 | 0.89 |
| ABC-1 | 1 | 4 | BCA | Y | BAC | 0.89 | 0.89 |
| ABC-1 | 1 | 5 | CAB | Y | ABC | 0.9 | 0.9 |
| ABC-1 | 1 | 6 | CBA | Y | BAC | 0.89 | 0.89 |
| ABC-1 | 2 | 1 | ABC | Y | ABC | 0.9 | 0.9 |
| ABC-1 | 2 | 2 | ACB | Y | BCA | 0.9 | 0.89 |
| ABC-1 | 2 | 3 | BAC | Y | BCA | 0.9 | 0.89 |
| ABC-1 | 2 | 4 | BCA | Y | ABC | 0.9 | 0.9 |
| ABC-1 | 2 | 5 | CAB | Y | BCA | 0.9 | 0.89 |
| ABC-1 | 2 | 6 | CBA | Y | CBA | 0.9 | 0.89 |
| ABC-1 | 3 | 1 | ABC | Y | ABC | 0.9 | 0.9 |
| ABC-1 | 3 | 2 | ACB | Y | BCA | 0.9 | 0.89 |
| ABC-1 | 3 | 3 | BAC | Y | BAC | 0.88 | 0.88 |
| ABC-1 | 3 | 4 | BCA | Y | BAC | 0.89 | 0.89 |
| ABC-1 | 3 | 5 | CAB | Y | ABC | 0.9 | 0.9 |
| ABC-1 | 3 | 6 | CBA | Y | BCA | 0.89 | 0.89 |
| ABC-1 | 4 | 1 | ABC | Y | BAC | 0.89 | 0.89 |
| ABC-1 | 4 | 2 | ACB | Y | BAC | 0.89 | 0.89 |
| ABC-1 | 4 | 3 | BAC | Y | BCA | 0.89 | 0.89 |
| ABC-1 | 4 | 4 | BCA | Y | BAC | 0.89 | 0.88 |
| ABC-1 | 4 | 5 | CAB | Y | BCA | 0.9 | 0.89 |
| ABC-1 | 4 | 6 | CBA | Y | ABC | 0.9 | 0.9 |
| ABC-1 | 5 | 1 | ABC | Y | ABC | 0.9 | 0.9 |
| ABC-1 | 5 | 2 | ACB | Y | ABC | 0.9 | 0.9 |
| ABC-1 | 5 | 3 | BAC | Y | BAC | 0.89 | 0.89 |
| ABC-1 | 5 | 4 | BCA | Y | ABC | 0.9 | 0.9 |
| ABC-1 | 5 | 5 | CAB | Y | BCA | 0.9 | 0.89 |
| ABC-1 | 5 | 6 | CBA | Y | BAC | 0.89 | 0.89 |
| ABC-2 | 1 | 1 | ABC | Y | ABC | 0.88 | 0.89 |
| ABC-2 | 1 | 2 | ACB | Y | ACB | 0.87 | 0.88 |
| ABC-2 | 1 | 3 | BAC | Y | ABC | 0.88 | 0.88 |
| ABC-2 | 1 | 4 | BCA | Y | ABC | 0.87 | 0.88 |
| ABC-2 | 1 | 5 | CAB | Y | ABC | 0.87 | 0.88 |
| ABC-2 | 1 | 6 | CBA | Y | ABC | 0.87 | 0.88 |
| ABC-2 | 2 | 1 | ABC | Y | ABC | 0.87 | 0.88 |
| ABC-2 | 2 | 2 | ACB | Y | ABC | 0.87 | 0.88 |
| ABC-2 | 2 | 3 | BAC | Y | ABC | 0.87 | 0.88 |
| ABC-2 | 2 | 4 | BCA | Y | ABC | 0.87 | 0.88 |
| ABC-2 | 2 | 5 | CAB | Y | ABC | 0.88 | 0.89 |
| ABC-2 | 2 | 6 | CBA | Y | ABC | 0.87 | 0.88 |
| ABC-2 | 3 | 1 | ABC | Y | ABC | 0.88 | 0.88 |
| ABC-2 | 3 | 2 | ACB | Y | ABC | 0.88 | 0.88 |
| ABC-2 | 3 | 3 | BAC | Y | BCA | 0.86 | 0.88 |
| ABC-2 | 3 | 4 | BCA | Y | ABC | 0.87 | 0.88 |
| ABC-2 | 3 | 5 | CAB | Y | ABC | 0.87 | 0.88 |
| ABC-2 | 3 | 6 | CBA | Y | ABC | 0.88 | 0.88 |
| ABC-2 | 4 | 1 | ABC | Y | ABC | 0.88 | 0.88 |
| ABC-2 | 4 | 2 | ACB | Y | ABC | 0.88 | 0.88 |
| ABC-2 | 4 | 3 | BAC | Y | ABC | 0.87 | 0.88 |
| ABC-2 | 4 | 4 | BCA | Y | ABC | 0.88 | 0.88 |
| ABC-2 | 4 | 5 | CAB | Y | BCA | 0.87 | 0.88 |
| ABC-2 | 4 | 6 | CBA | Y | ABC | 0.87 | 0.88 |
| ABC-2 | 5 | 1 | ABC | Y | ABC | 0.88 | 0.88 |
| ABC-2 | 5 | 2 | ACB | Y | ABC | 0.88 | 0.88 |
| ABC-2 | 5 | 3 | BAC | Y | ABC | 0.88 | 0.89 |
| ABC-2 | 5 | 4 | BCA | Y | ABC | 0.88 | 0.88 |
| ABC-2 | 5 | 5 | CAB | Y | ABC | 0.88 | 0.88 |
| ABC-2 | 5 | 6 | CBA | Y | ABC | 0.87 | 0.88 |
| AAB-FOGER | 1 | 1 | AAB | Y | AAB | 0.87 | 0.86 |
| AAB-FOGER | 1 | 2 | ABB | Y | ABB | 0.87 | 0.87 |
| AAB-FOGER | 1 | 3 | BAA | Y | BAA | 0.86 | 0.86 |
| AAB-FOGER | 1 | 4 | BBA | Y | BBA | 0.87 | 0.87 |
| AAB-FOGER | 1 | 5 | AB | N | AB | 0.71 | 0.74 |
| AAB-FOGER | 1 | 6 | BA | N | BA | 0.55 | 0.63 |
| AAB-FOGER | 2 | 1 | AAB | Y | AAB | 0.87 | 0.86 |
| AAB-FOGER | 2 | 2 | ABB | Y | ABB | 0.87 | 0.87 |
| AAB-FOGER | 2 | 3 | BAA | Y | BAA | 0.86 | 0.87 |
| AAB-FOGER | 2 | 4 | BBA | Y | ABB | 0.87 | 0.87 |
| AAB-FOGER | 2 | 5 | AB | N | AB | 0.71 | 0.73 |
| AAB-FOGER | 2 | 6 | BA | N | AB | 0.71 | 0.74 |
| AAB-FOGER | 3 | 1 | AAB | Y | AAB | 0.87 | 0.86 |
| AAB-FOGER | 3 | 2 | ABB | Y | ABB | 0.87 | 0.87 |
| AAB-FOGER | 3 | 3 | BAA | Y | AAB | 0.87 | 0.86 |
| AAB-FOGER | 3 | 4 | BBA | Y | BBA | 0.87 | 0.87 |
| AAB-FOGER | 3 | 5 | AB | N | BA | 0.55 | 0.63 |
| AAB-FOGER | 3 | 6 | BA | N | AB | 0.71 | 0.74 |
| AAB-FOGER | 4 | 1 | AAB | Y | BAA | 0.86 | 0.87 |
| AAB-FOGER | 4 | 2 | ABB | Y | BBA | 0.87 | 0.87 |
| AAB-FOGER | 4 | 3 | BAA | Y | BAA | 0.86 | 0.87 |
| AAB-FOGER | 4 | 4 | BBA | Y | BBA | 0.87 | 0.87 |
| AAB-FOGER | 4 | 5 | AB | N | AB | 0.71 | 0.74 |
| AAB-FOGER | 4 | 6 | BA | N | AB | 0.71 | 0.74 |
| AAB-FOGER | 5 | 1 | AAB | Y | AAB | 0.87 | 0.86 |
| AAB-FOGER | 5 | 2 | ABB | Y | ABB | 0.87 | 0.87 |
| AAB-FOGER | 5 | 3 | BAA | Y | AAB | 0.87 | 0.87 |
| AAB-FOGER | 5 | 4 | BBA | Y | ABB | 0.87 | 0.87 |
| AAB-FOGER | 5 | 5 | AB | N | BA | 0.59 | 0.66 |
| AAB-FOGER | 5 | 6 | BA | N | AB | 0.7 | 0.73 |
| ABC-FOGER | 1 | 1 | ABC | Y | ABC | 0.87 | 0.88 |
| ABC-FOGER | 1 | 2 | ACB | Y | ABC | 0.87 | 0.88 |
| ABC-FOGER | 1 | 3 | BAC | Y | BCA | 0.89 | 0.89 |
| ABC-FOGER | 1 | 4 | BCA | Y | ABC | 0.87 | 0.88 |
| ABC-FOGER | 1 | 5 | CAB | Y | ABC | 0.88 | 0.88 |
| ABC-FOGER | 1 | 6 | CBA | Y | ABC | 0.87 | 0.88 |
| ABC-FOGER | 2 | 1 | ABC | Y | ABC | 0.87 | 0.88 |
| ABC-FOGER | 2 | 2 | ACB | Y | ABC | 0.88 | 0.88 |
| ABC-FOGER | 2 | 3 | BAC | Y | ABC | 0.88 | 0.88 |
| ABC-FOGER | 2 | 4 | BCA | Y | ABC | 0.88 | 0.88 |
| ABC-FOGER | 2 | 5 | CAB | Y | ABC | 0.88 | 0.88 |
| ABC-FOGER | 2 | 6 | CBA | Y | ABC | 0.88 | 0.88 |
| ABC-FOGER | 3 | 1 | ABC | Y | ABC | 0.87 | 0.88 |
| ABC-FOGER | 3 | 2 | ACB | Y | BCA | 0.88 | 0.88 |
| ABC-FOGER | 3 | 3 | BAC | Y | ABC | 0.88 | 0.88 |
| ABC-FOGER | 3 | 4 | BCA | Y | ABC | 0.87 | 0.87 |
| ABC-FOGER | 3 | 5 | CAB | Y | ABC | 0.88 | 0.88 |
| ABC-FOGER | 3 | 6 | CBA | Y | ABC | 0.88 | 0.88 |
| ABC-FOGER | 4 | 1 | ABC | Y | CAB | 0.88 | 0.88 |
| ABC-FOGER | 4 | 2 | ACB | Y | ABC | 0.87 | 0.88 |
| ABC-FOGER | 4 | 3 | BAC | Y | ABC | 0.88 | 0.88 |
| ABC-FOGER | 4 | 4 | BCA | Y | BCA | 0.88 | 0.88 |
| ABC-FOGER | 4 | 5 | CAB | Y | ABC | 0.88 | 0.88 |
| ABC-FOGER | 4 | 6 | CBA | Y | ABC | 0.87 | 0.87 |
| ABC-FOGER | 5 | 1 | ABC | Y | ABC | 0.88 | 0.88 |
| ABC-FOGER | 5 | 2 | ACB | Y | ABC | 0.87 | 0.88 |
| ABC-FOGER | 5 | 3 | BAC | Y | CAB | 0.88 | 0.88 |
| ABC-FOGER | 5 | 4 | BCA | Y | ABC | 0.88 | 0.88 |
| ABC-FOGER | 5 | 5 | CAB | Y | ABC | 0.88 | 0.88 |
| ABC-FOGER | 5 | 6 | CBA | Y | ABC | 0.88 | 0.88 |


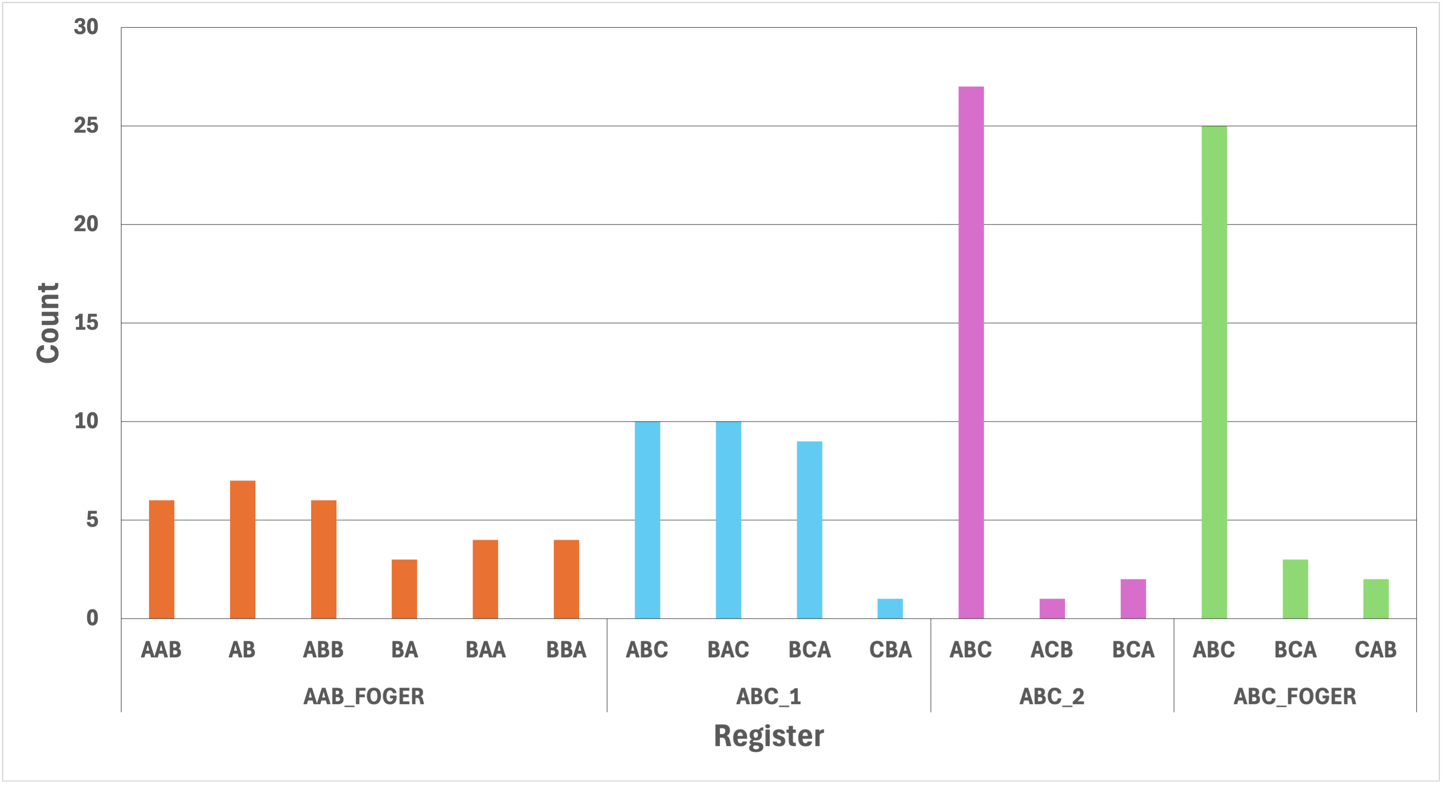


**Figure S62.** Distribution of predictions on heterotrimer registers by AlphaFold3


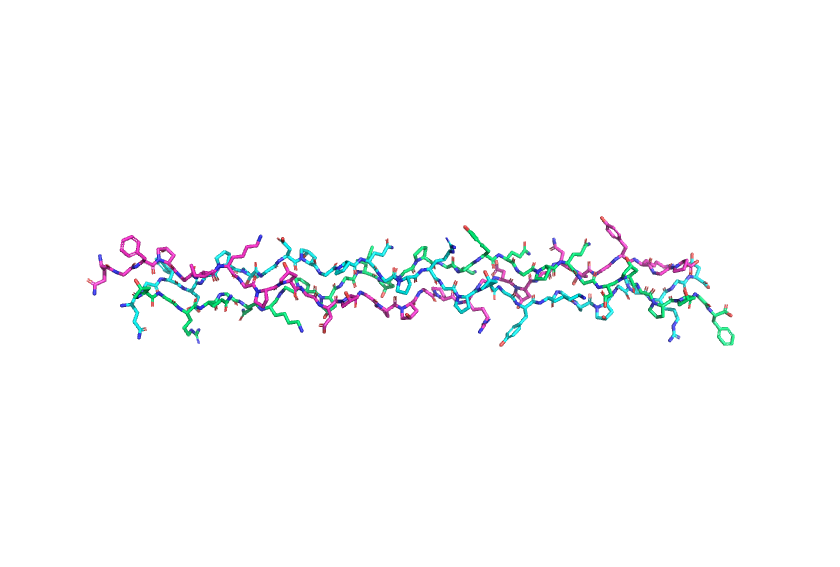


**Figure S63.** 3D structure of ABC-1 by AlphaFold3. Trial 1; input: ABC; predicted: ABC. Peptide A is pink; peptide B is cyan; peptide C is green.


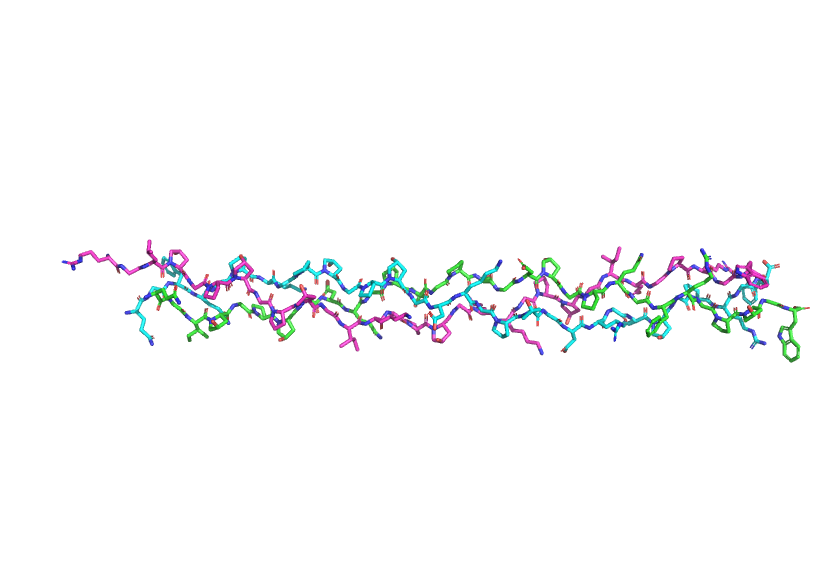


**Figure S64.** 3D structure of ABC-2 by AlphaFold3. Trial 1; input: ABC; predicted: ABC. Peptide A is pink; peptide B is cyan; peptide C is green.


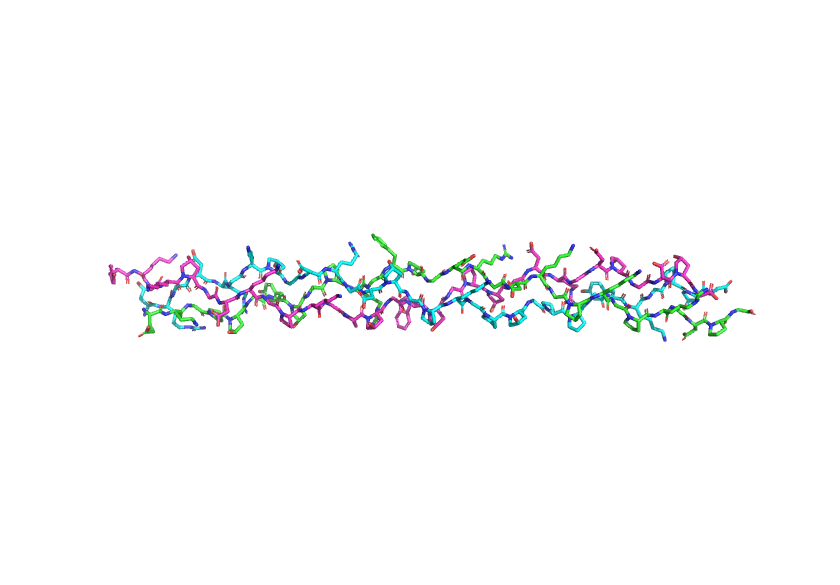


**Figure S65.** 3D structure of ABC-FOGER by AlphaFold3. Trial 1; input: ABC; predicted: ABC. Peptide A is pink; peptide B is cyan; peptide C is green.


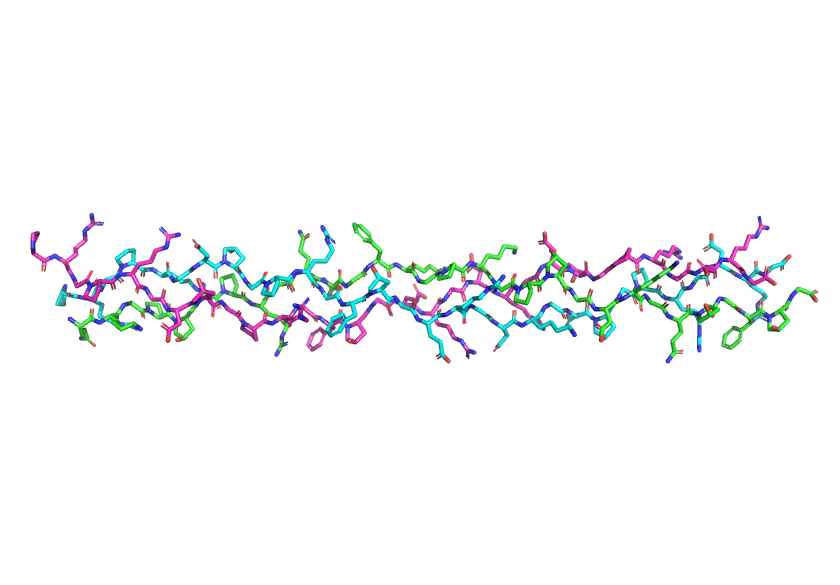


**Figure S66.** 3D structure of AAB-FOGER by AlphaFold3. Trial 1; input: AAB; predicted: AAB. Peptide A (leading chain) is pink; peptide A (middle chain) is cyan; peptide B is green.


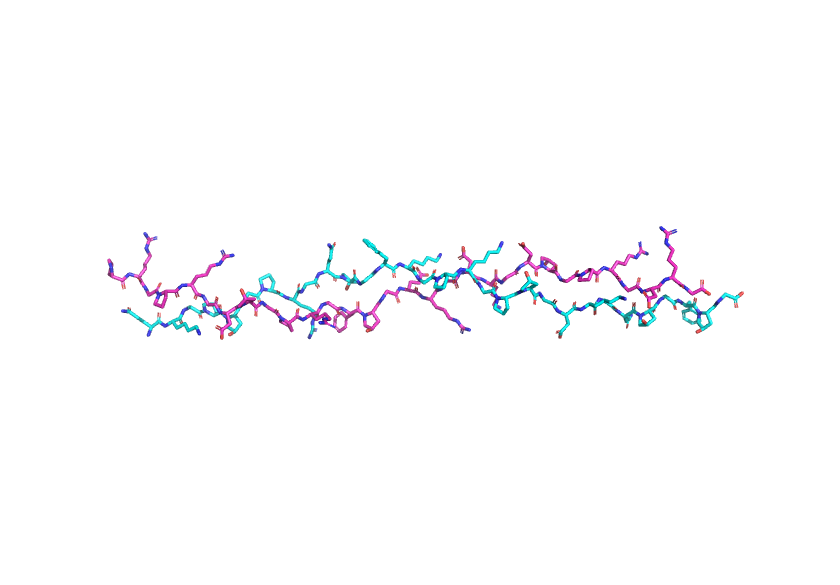


**Figure S67**. 3D structure of AAB-FOGER by AlphaFold3 as a dimer when inputting only two peptide chains. Trial 1; input: AB; predicted: AB. Peptide A is pink; peptide B is green.

# **9. GRACE example runs**

**
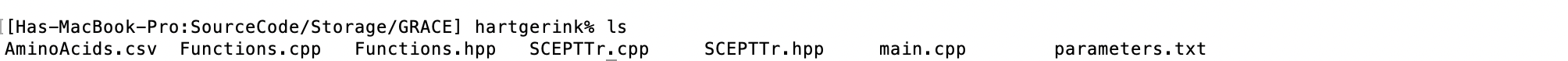
**

**Figure S68.** Navigate to the directory containing all of the source files.


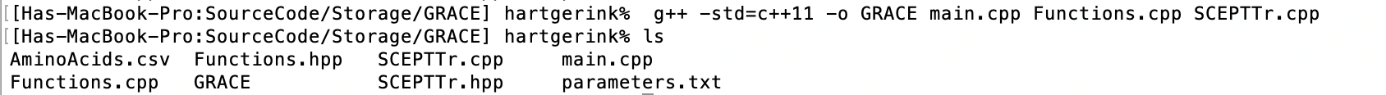


**Figure S69.** Compile using g++ -std=c++11 -o GRACE main.cpp Functions.cpp SCEPTTr.cpp This will generate an executable named GRACE.


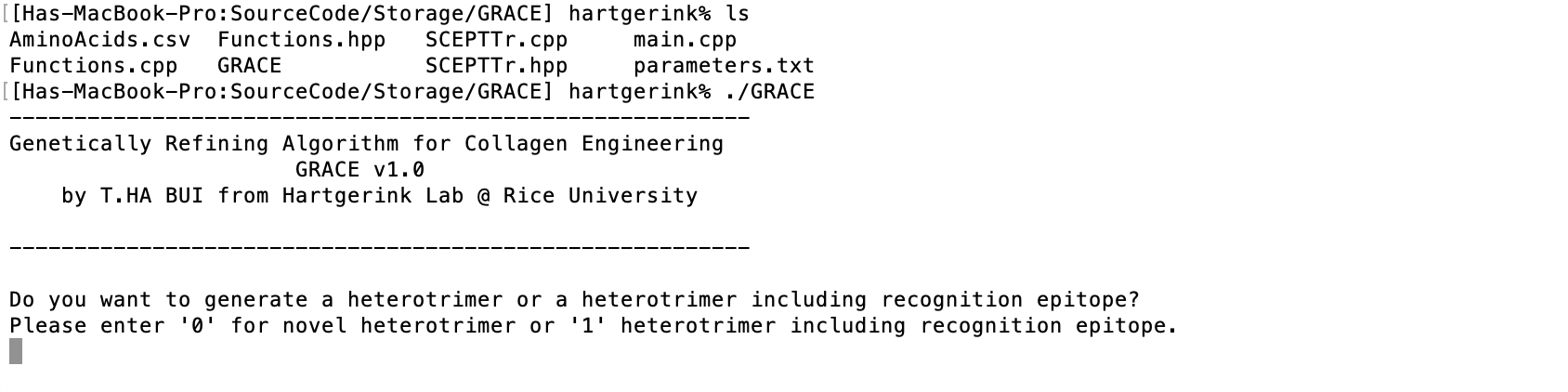


**Figure S70.** Ensure **AminoAcids.csv** and **parameters.txt** are in the same directory as the compiled executable **GRACE**. To execute, type: ./GRACE and follow the on-screen prompts to input search conditions.


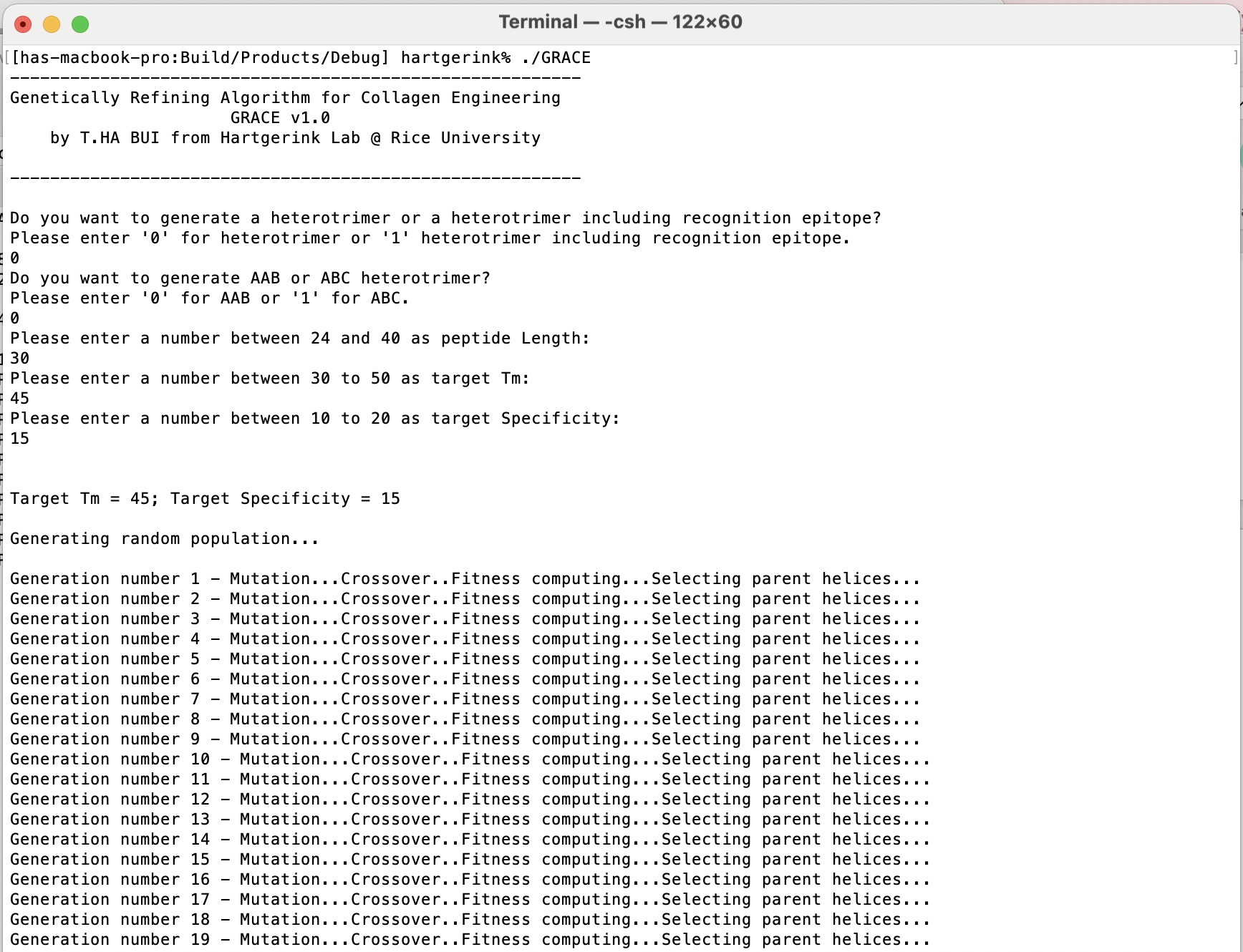


**Figure S71.** Execution of GRACE to generate an AAB-type heterotrimer with 30 residues in each strand. Melting temperature and specificity are targeted to be at least 45 and 15, respectively.


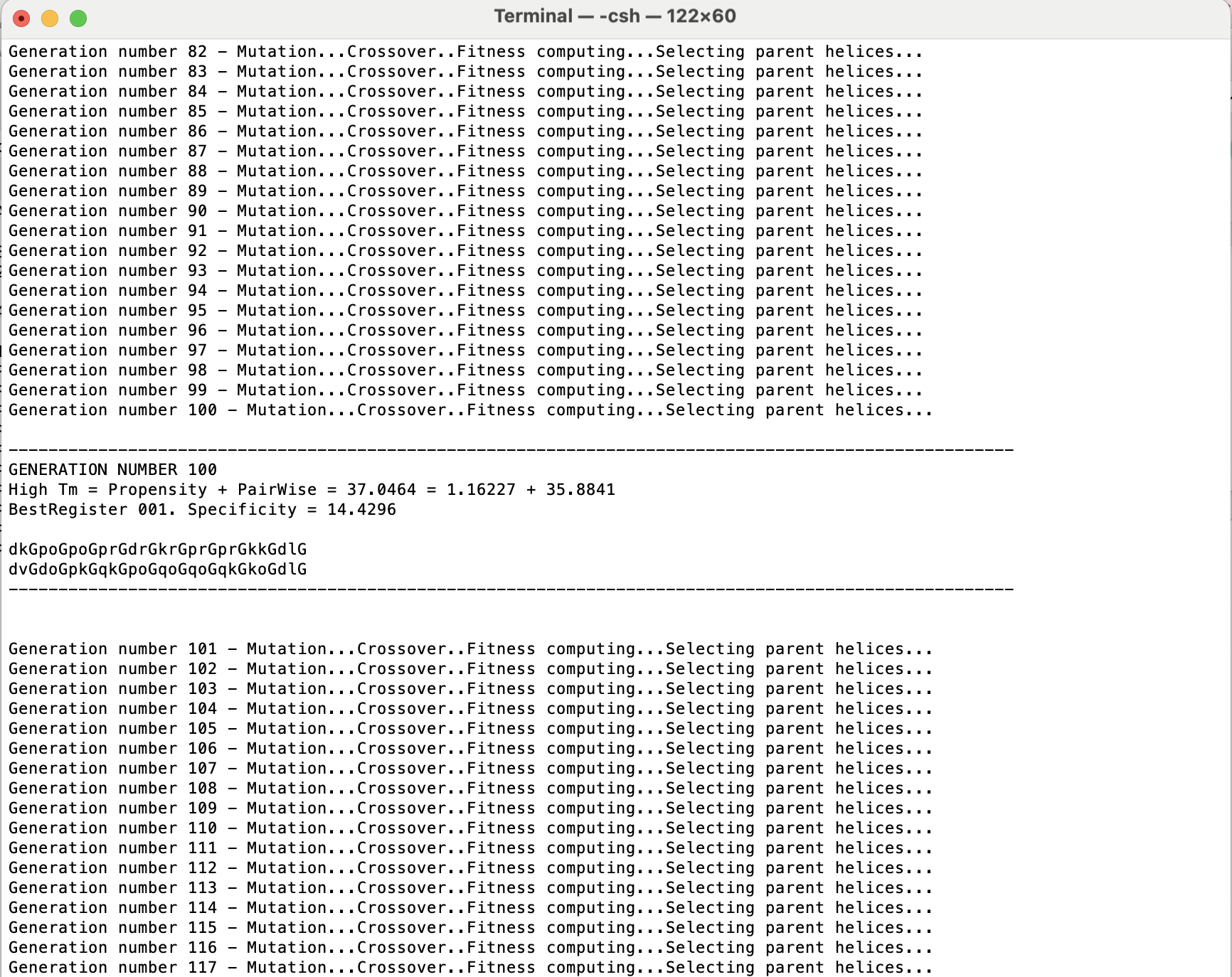


**Figure S72.** The algorithm generates sequences and outputs predicted register, melting temperature, and specificity every 100 generations, enabling monitoring of improvements.


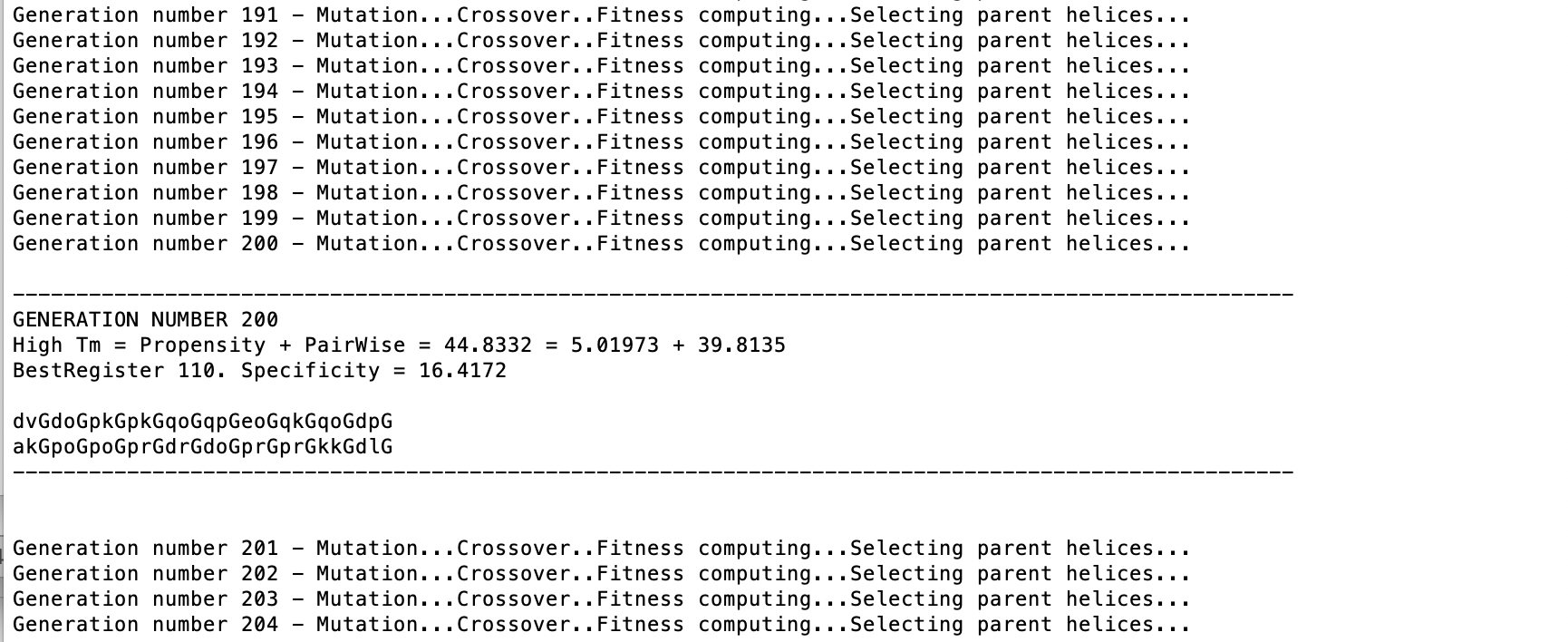


**Figure S73.** Output after 200 generations


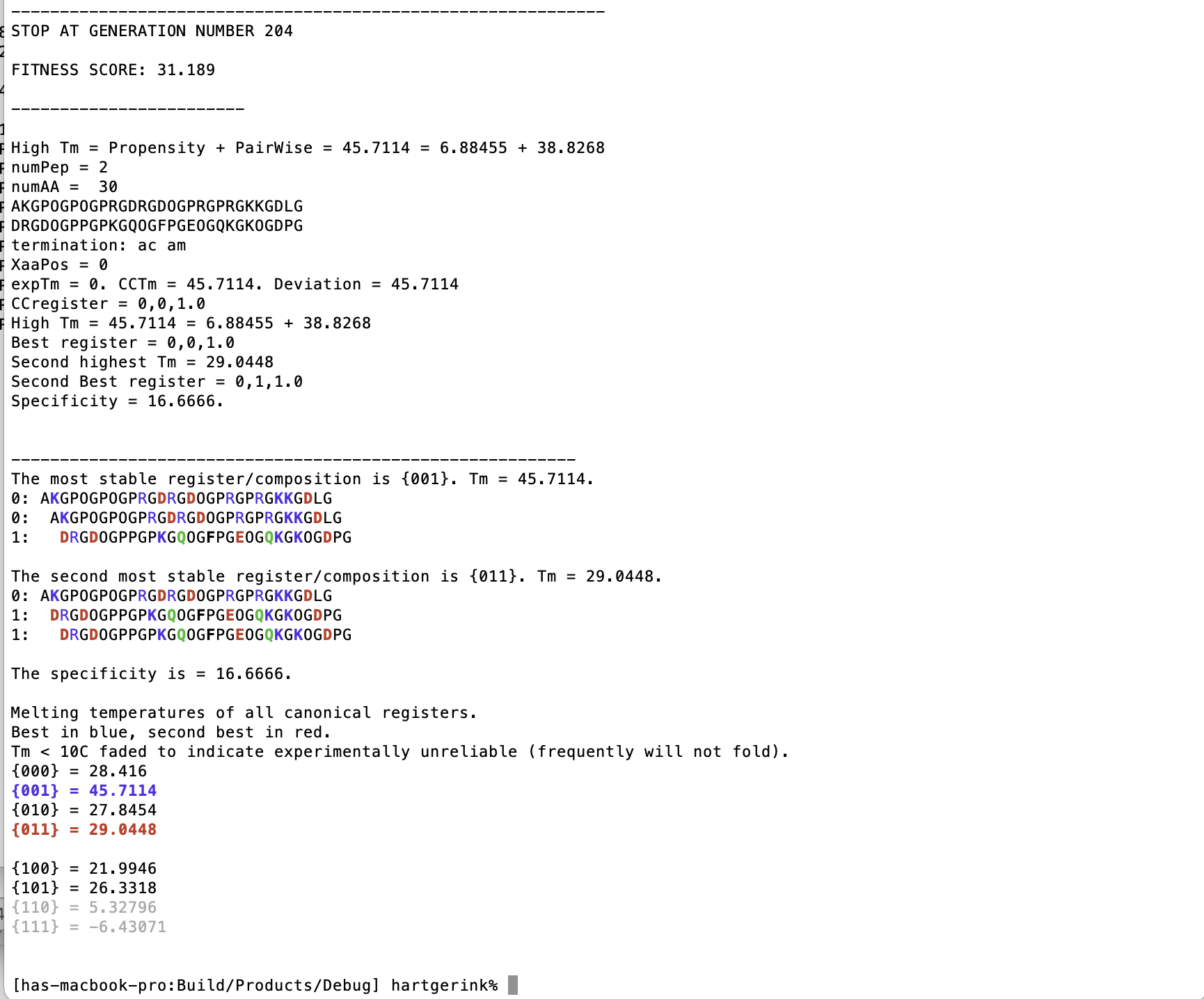


**Figure S74.** Final output showing a set of sequences satisfying the target melting temperature and specificity. Melting temperature of all registers were also estimated.


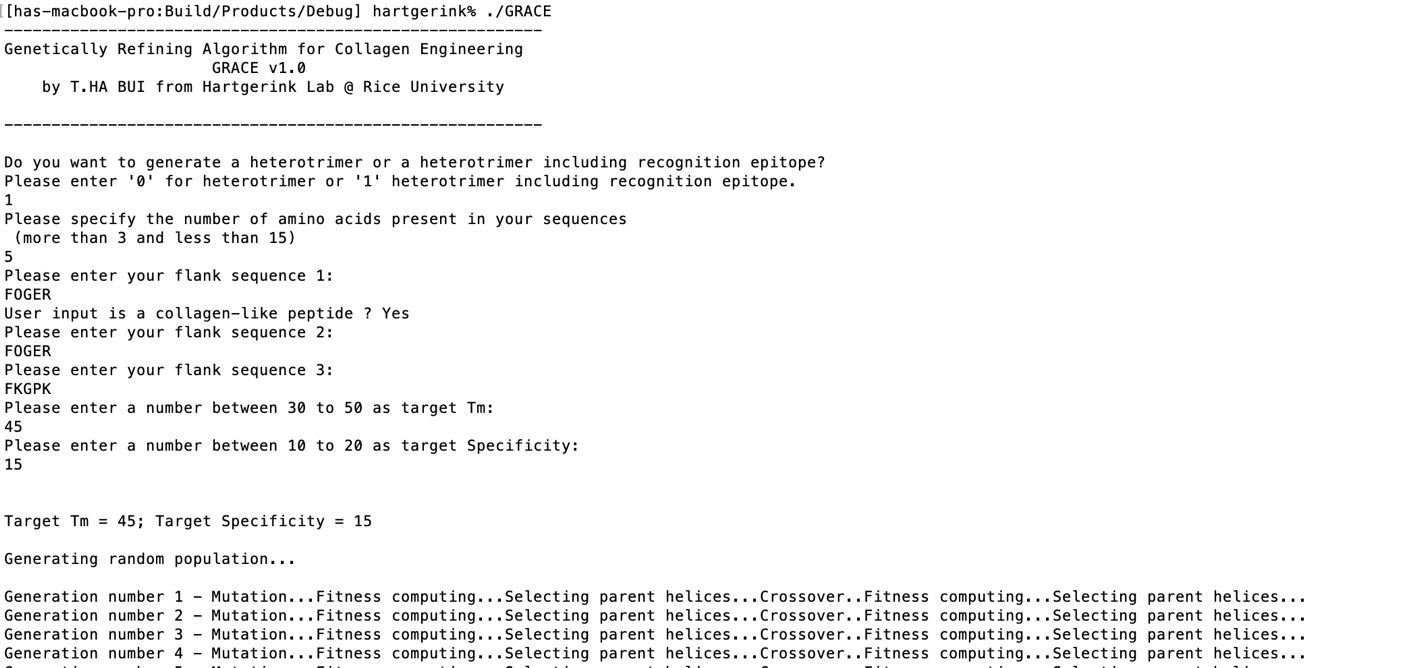


**Figure S75.** Execution of GRACE to generate a heterotrimer with user sequences. Each user sequence has 5 residues. Sequence on leading strand is ‘FOGER’, on middle strand is ‘FOGER’, and on trailing strand is ‘FKGPK’.

**Figure S76**. Final output. The most stable assembly has user sequence 1 on leading strand, user sequence 2 on middle strand, and user sequence 3 on trailing strand.
